# Factors associated with low school readiness, a linked health and education data study in Wales, UK

**DOI:** 10.1371/journal.pone.0273596

**Published:** 2023-12-11

**Authors:** Amrita Bandyopadhyay, Emily Marchant, Hope Jones, Michael Parker, Julie Evans, Sinead Brophy

**Affiliations:** 1 National Centre for Population Health and Wellbeing Research, Swansea University Medical School, Wales, United Kingdom; 2 Public Health Wales, Keir Hardie University Health Park, Wales, United Kingdom; University of Oxford, UNITED KINGDOM

## Abstract

**Background:**

School readiness is a measure of a child’s cognitive, social, and emotional readiness to begin formal schooling. Children with low school readiness need additional support from schools for learning, developing required social and academic skills, and catching-up with their school-ready peers. This study aims to identify the most significant risk factors associated with low school readiness using linked routine data for children in Wales.

**Method:**

This was a longitudinal cohort study using linked data. The cohort comprises of children who completed the Foundation Phase assessment between 2012 and 2018. Individuals were identified by linking Welsh Demographic Service and Pre16 Education Attainment datasets. School readiness was assessed via the binary outcome of the Foundation Phase assessment (achieved/not achieved). This study used multivariable logistic regression model and a decision tree to identify and weight the most important risk factors associated with low school readiness.

**Results:**

In order of importance, logistic regression identified maternal learning difficulties (adjusted odds ratio 5.35(95% confidence interval 3.97–7.22)), childhood epilepsy (2.95(2.39–3.66)), very low birth weight (2.24(1.86–2.70), being a boy (2.11(2.04–2.19)), being on free school meals (1.85(1.78–1.93)), living in the most deprived areas (1.67(1.57–1.77)), maternal death (1.47(1.09–1.98)), and maternal diabetes (1.46(1.23–1.78)) as factors associated with low school readiness. Using a decision tree, eligibility for free school meals, being a boy, absence/low attendance at school, being born late in the academic year, being a low birthweight child, and not being breastfed were factors which were associated with low school readiness.

**Conclusion:**

This work suggests that public health interventions focusing on children who are: boys, living in deprived areas, have poor early years attendance, have parents with learning difficulties, have parents with an illness or have illnesses themselves, would make the most difference to school readiness in the population.

## Introduction

### Background

Early childhood education shapes the direction of a child’s development, enhances their ability to learn in the school environment and strengthens their foundation for lifelong learning [1,2]. School readiness encompasses cognitive, social, and emotional aspects and indicates if a child can achieve at an appropriate level in formal school. School readiness is also a determinant of health and wellbeing over the life course [3,4]. It is strongly linked to the pre-school environment, and it indicates the acquisition of the necessary social skills, emotional skills, knowledge, and attitude to effectively engage and learn in school. School readiness is defined by a child’s physical well-being and motor development (e.g., co-ordination, fine motor-skills), social and emotional development (co-operation, empathy, and the ability to express their emotion), approaches towards learning (enthusiasm, curiosity, temperament), language and communication (listening and speaking), basic knowledge (essential vocabulary and numbers) and cognitive skills (problem solving) [4].

A review of published literature on the risk factors associated with school readiness indicates that area-level characteristics, parental demography, and parental and child health conditions play a significant role in school readiness. Factors associated with higher school readiness include higher levels of child care provision in the area where the child is brought up [5,6], living in private housing [7], the mother’s age (between late twenties or thirties) [7,8], breastfeeding (higher rates and longer duration) [7,9], dual parent households, a nurturing parenting style [7,10] and parents with good physical [10,11] and mental health [7,9,10]. Similarly, good physical health of the child (being born at term and a healthy birth weight) [12,13] is also associated with higher school readiness. Conversely, low access to childcare, higher levels of unemployment (area and family level), living in social housing, exposure to poor environment such as damp, maternal heavy drinking behaviours [14], mother who smoked during pregnancy [5,12], teenage mothers or older mothers (35+ years) and parents with poor physical health (hypertension, diabetes), poor mental health, single parent or step-parent families, low expectations by the parent for the child, preterm or low birth weight child, and poor health of the child are also associated with low school readiness [6,7]. An Australian data linkage study conducted by Chittleborough et al, identified a group of predictors (such as maternal age, smoking during pregnancy, parity, marital status, and both parents’ occupation and gender) which were capable to identify the children at risk of developmental vulnerability at school entry [15].

Since being school ready is associated with many positive outcomes, improving school readiness is a necessary strategy for economic development and social mobility [16]. If children are not school ready, it can take many years for them to catch up with their peers, if ever, [17,18] and therefore contribute to widening inequalities. School readiness has been identified as a key public health concern in a recent review of UK public health systems and policy approaches to early child development [19]. It is very challenging to identify the right individuals (children and families) who are at risk in order to provide the necessary support [20]. Therefore, identifying the most significant risk factors is a priority in closing the gap in children’s school readiness and improving outcomes for children. Studies have shown that routine data obtained during a child’s birth can help to identify the children and the families at risk of poor development [21,22]. A framework using routinely collected administrative data can inform the appropriate supporting agencies to provide adequate help and support to the most vulnerable of the society.

### Objective

The aim of the study is to identify and weight the most significant risk factors of low school readiness using linked routine data for children in Wales. This work also examined the risk factors which were clustered together and build a vulnerability profile of the children who are at risk of low school readiness. The factors which are associated with school readiness are examined using; a) traditional statistical methods (multivariable logistic regression model, to observe the highly associated risk factors) and b) data driven supervised machine learning classification algorithm (decision tree, to measure the commonly observed and prevalent risk factors at the population level). In a logistic regression model, the log-odds for low school readiness as a linear combination of explanatory variables and confounders have been investigated. On the other hand, the decision tree model, based on recursive partitioning, highlights the statistically significant hierarchically clustered features for low school readiness and captures complex relationship between the risk factors and low school readiness. The hierarchically clustered features from the decision tree and the risk factors identified from the logistic model are important to cross-validate the set of overlapping risk factors and serves to strengthen the importance of the findings. These risk factors will inform the development of a profiling model by identifying the socio-economic and physical and mental health barriers that the child and/or their families face, which may impact the child’s ability to meet the developmental milestones necessary to progress effectively through the early years. The primary focus of the model is to build a holistic understanding of the most significant risk factors of the low school readiness which can inform the necessary support system required for the individuals and families at highest need and make more efficient use of the resources.

## Methods

### Sample selection and data linkage

**Fig 1:**
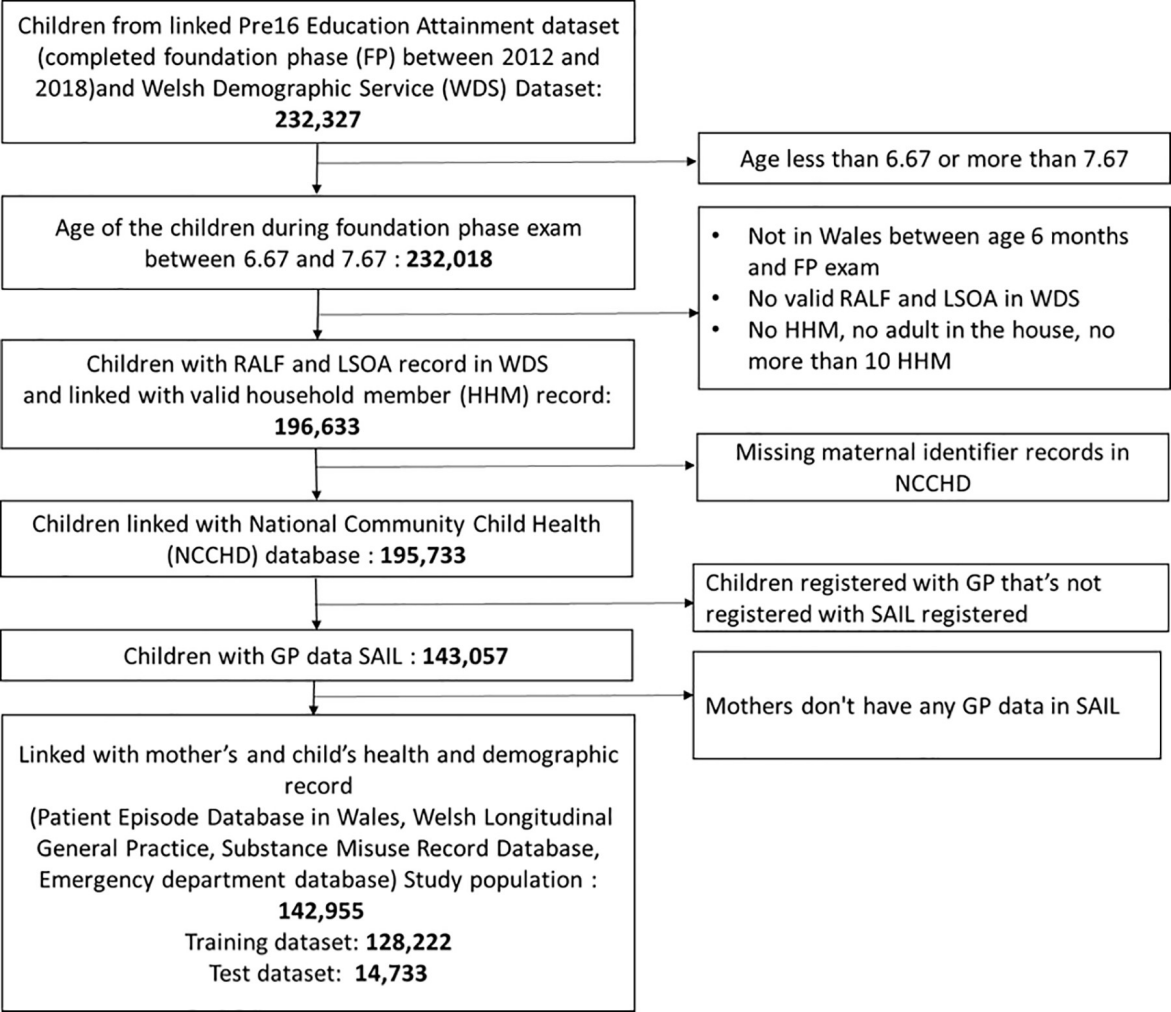
Flow diagram of the study population.

In this cohort study, the study population was derived by linking the Welsh Demographic Service (WDS) dataset (administrative dataset about individuals in Wales that use NHS services) and the Pre16 Education Attainment dataset (individual-level administrative data relating to the education system in Wales). The study population consists of children who completed Foundation Phase (a statutory curriculum for children aged 3–7 years) [23] between 2012 and 2018. The data linkage was done using an encrypted key known as Anonymised Linking Fields (ALF) in the Secure Anonymised Information Linkage (SAIL) Databank [24,25]. Residential Anonymised Linking Fields (RALFs) are an encrypted residential address available in WDS dataset, which is also linked with a smaller geographical unit known as lower super output area (LSOA). Using ALFs, RALFs and LSOA the study population were anonymously linked with the individuals living with the child in the same household during the child’s Foundation Phase [26]. Children without valid and continuous RALFs and primary care records in Welsh Longitudinal General Practice (WLGP) dataset in SAIL until their completion of Foundation Phase were not included in the study to ensure the complete coverage of exposure and outcome data during the study period. The study population was linked with the National Community Child Health Database (NCCHD) to obtain birth and maternal records during childbirth. Records with missing maternal identifiers and mothers with no primary care record in WLGP dataset were not included in the study. The flow diagram of the selection of the study population is presented in Fig 1.

### Risk factors from routine data

The selection of risk factors associated with low school readiness has been informed by the literature review undertaken at the inception of the study. The risk factors had been selected from the routinely collected electronic administrative and health datasets and this provided the framework upon which the current study was developed. The literature review focused on observational studies including case controls, cohort studies and studies using linked routine data with the primary or secondary outcomes examining school readiness. Depending on the strength of association (Odds Ratio) between the risk factors and low school readiness a list of risk factors were prepared, and their analogous variables were created or selected from linked routine data. The literature review to select the risk factors and how these were mapped with routine data, have been described in a Supplementary document (Appendix 1 in S1 File and Appendix 2 in S1 File). General demography and birth-related variables including gender, gestational age, birth weight, breastfeeding, mode of delivery (caesarean section/assisted delivery/natural delivery) and maternal age at childbirth were obtained from NCCHD. The multiple birth (singleton/non-singleton) flag was derived using week of birth of the child, encrypted maternal identifier and the birth order of the children. To identify the children who lost their mother before the Foundation Phase, a binary variable was derived. Maternal physical health (diabetes, cancer, anaemia, hypertension, learning difficulty) and mental health (depression, anxiety, serious mental illness, medication related to anxiety/depression) related primary and secondary care records during and after pregnancy until Foundation Phase were obtained from WLGP and hospital admission dataset—Patient Episode database in Wales (PEDW). The Substance Misuse Database (SMD) was used to populate information on the mothers’ alcohol or other substance abuse related record during the study period. Any coded READ and ICD10 codes related to substance misuse on WLGP and PEDW dataset were also considered in this study. Mothers’ alcohol related hospital admission records were obtained from PEDW. Maternal smoking during and after pregnancy were obtained from WLGP, smoking related READ code mentioned on the dataset during the study period were considered to build the variable. The record of physical assault related hospital admissions of mothers during or post pregnancy was obtained from PEDW. The hospital admission and GP records of the children for epilepsy, asthma, diabetes, ear infections, and eye infections were considered as a measure of child health conditions. Any emergency hospital admission and any accident and emergency (A&E) attendance of the study population between birth and Foundation Phase were obtained from PEDW and Emergency Department dataset (EDDS). READ code version 2 and ICD10 codes have been used to identify the health records from WLGP and PEDW dataset (see Appendix 3 in S1 File). Any coded diagnosis of the above-mentioned physical and mental health conditions for mother and child during their GP visit (obtained from WLGP) or hospital admission (obtained from PEDW) were considered in this study. The children’s age at the completion of their Foundation Phase and the total number of days they were absent in the school in early years (e.g., nursery) were obtained from Pre16 Education Attainment dataset. Household characteristics such as living in a single adult household, total number of adults, and total number of other children in the household were derived from the WDS dataset. In this study the eligibility for free school meals (FSM) during Foundation Phase was used to measure the family-level deprivation of the study population. The area-level deprivation was measured by the Welsh Index of Multiple Deprivation (WIMD) 2014 which provides a measure of the relative deprivation in Wales linked to LSOA [27]. The local authorities and the type of local area (urban/rural) where the children were brought up during Foundation Phase were included in the study.

### School readiness from routine data

The binary Foundation Phase Indicator variable was obtained from the Pre16 Education Attainment dataset and was used as a measure of school readiness from the routine data in the current study. The National Curriculum assess school readiness using the Foundation Phase Indicator at the end of early year foundation stage where the child would be at the age of 6 or 7. The Foundation Phase Indicator represents whether the child has achieved at least the expected level 5 or above in the early stage learning goals in the following areas;—i) personal and social development, well-being and cultural diversity, ii) language, literacy, and communication skills–English/Welsh and iii) mathematical development [28]. In this study a binary variable has been derived based on the Foundation Phase Indicator record as a measure of school readiness from routine data.

- Low school readiness = Not achieved Foundation Phase
- School readiness = Achieved Foundation Phase

### Statistical analysis

A multivariable logistic regression model was first developed to identify and weight the most important risk factors associated with school readiness. Next, we built a data driven machined learning classifier model using decision tree to investigate the most commonly observed risk factors at the population level. Since the children with learning difficulties or special educational needs tend to have a much higher risk of low school readiness, they were removed from the models. Data preparation including data linkage was performed on DB2 SQL platform and the statistical analysis was done in R version 4.0.3.

#### Logistic regression

To identify the most important risk factors associated with low school readiness we used multivariable logistic regression. Variables included gender, gestational age, birthweight, breastfeeding, caesarean section, multiple birth, maternal age, maternal death before Foundation Phase, maternal physical and mental health, child physical (epilepsy, asthma, diabetes, ear, and eye) and mental health conditions (depression, anxiety), free school meal uptake, local area status and number of adults and children living in the same household. The significant risk factors of low school readiness are presented with their adjusted Odds Ratio (aOR) and 95% confidence interval (CI).

#### Decision tree

A classification tree–decision tree algorithms were developed using RPART (Recursive Partitioning And Regression Trees) packages in R [29,30]. The algorithm repeatedly partitions the data into multiple sub-spaces to reach the homogeneous end sub-space, hence it is called recursive partitioning. For decision trees, the data for one representative local authority was removed from the dataset and used as the testing dataset to validate the model performance and examine generalisability within areas of Wales.

## Results

### Overall sample characteristics

The study population consisted of 142,955 children (training dataset: 128,222, testing dataset: 14,733) who completed Foundation Phase between 2012 and 2018 in Wales (see Table 1). 14.32% (Training dataset: 14.15%, Testing dataset: 15.75%) children did not achieve in Foundation Phase. The study population consisted of 51.24% boys, 42.87% were not breastfed and 24.83% were born via caesarean section. 8.33% were born to mothers aged below 19, 0.14% of mothers had learning difficulties and 0.23% lost their mother before their Foundation Phase assessment. There were 0.1% mothers who had an alcohol related hospital admission, 0.36% with substance abuse and 14.63**%** had a smoking record in WLGP during pregnancy. 0.64% of children had an epilepsy related GP visit, 0.46% had a hospital admission record for epilepsy. 3.30% and 4.54% children were admitted to hospital for asthma and ear infection respectively before they completed Foundation Phase. 56.37% of children had at least one emergency hospital admission and 66.4% had A&E records anytime between birth and Foundation Phase. 0.90% of children (Training dataset: 0.88%, Testing dataset 1.07%) were diagnosed with a learning difficulty. 22.05% of children were in single adult households, 19.57% were eligible for FSM and 25.66% lived in most deprived area measured by WIMD. Overall characteristics of the study population have been described in Table 1.

**Table 1:** Characteristics of the study population.

Variables	Overall (n = 142,955)		Training dataset (n = 128,222)		Testing dataset (n = 14,733)	
**Gender**						
**Girl**	69,703	48.76%	62,420	48.68%	7,283	49.43%
**Boy**	73,252	51.24%	65,802	51.32%	7,450	50.57%
**Gestational age**						
**Extremely pre-term: <28 weeks**	358	0.25%	319	0.25%	39	0.26%
**Very pre-term: 28–31**	1,173	0.82%	1,017	0.79%	156	1.06%
**Pre-term: 32–36**	8,434	5.90%	7,524	5.87%	910	6.18%
**Term: 37–42**	131,249	91.81%	117,708	91.80%	13,541	91.91%
**Late term: 43–45**	899	0.63%	849	0.66%	50	0.34%
**Unknown/NULL**	842	0.59%	805	0.63%	37	0.25%
**Birth weight**						
**Very low: <1500 g**	1,454	1.02%	1,295	1.0%	159	1.08%
**Low: 1500-<2500**	8,185	5.73%	7,207	5.6%	978	6.64%
**Normal: 2500-<4000g**	115,844	81.04%	103,767	80.9%	12,077	81.97%
**High: 4000-5000g**	16,802	11.75%	15,320	11.9%	1,482	10.06%
**Unknown**	670	0.47%	633	0.5%	37	0.25%
**Breastfeeding**						
**No**	61,287	42.87%	54,838	42.77%	6,449	43.77%
**Yes**	73,988	51.76%	66,037	51.50%	7,951	53.97%
**Unknown**	7,680	5.37%	7,347	5.73%	333	2.26%
**C-section birth**						
	35,489	24.83%	31,275	24.39%	4,214	28.60%
**Multiple birth**						
**Non-singleton**	3,922	2.74%	3,546	2.77%	376	2.55%
**Maternal age**						
**Less than 19**	11,910	8.33%	10,416	8.12%	1,494	10.14%
**20–24**	32,384	22.65%	28,598	22.30%	3,786	25.70%
**25–29**	39,356	27.53%	35,093	27.37%	4,263	28.94%
**30–34**	35,840	25.07%	32,534	25.37%	3,306	22.44%
**35 and above**	23,458	16.41%	21,574	16.83%	1,884	12.79%
**Unknown**	7	0.00%	7	0.01%		
**Death of mother before Foundation Phase**						
	327	0.23%	286	0.22%	41	0.28%
**Diabetes PEDW (mother)**						
	1,462	1.02%	1,308	1.02%	154	1.05%
**Diabetes GP (mother)**						
	1,367	0.96%	1231	0.96%	136	0.92%
**Cancer PEDW (mother)**						
	1,192	0.83%	1,097	0.86%	95	0.64%
**Cancer GP (mother)**						
	1,037	0.73%	947	0.74%	90	0.61%
**Anaemia PEDW (mother)**						
	7,317	5.12%	6,799	5.30%	518	3.52%
**Anaemia GP (mother)**						
	15,680	10.97%	14,340	11.18%	1,340	9.10%
**Hypertension GP (mother)**						
	2,599	1.82%	2,330	1.82%	269	1.83%
**Learning Difficulty GP (mother)**						
	205	0.14%	182	0.14%	23	0.16%
**Depression PEDW (mother)**						
	5,179	3.62%	4,648	3.62%	531	3.60%
**Depression GP (mother)**						
	29,332	20.52%	26,060	20.32%	3,272	22.21%
**Anxiety PEDW (mother)**						
	2,913	2.04%	2,626	2.05%	287	1.95%
**Anxiety GP (mother)**						
	30,278	21.18%	26,916	20.99%	3,362	22.82%
**Anti-Depression/anxiety medication (mother)**						
	396	0.28%	359	0.28%	37	0.25%
**Serious Mental Illness PEDW (mother)**						
	710	0.50%	624	0.49%	86	0.58%
**Serious Mental Illness GP (mother)**						
	776	0.54%	676	0.53%	100	0.68%
**Alcohol PEDW (mother)**						
**During pregnancy**	137	0.10%	129	0.10%	8	0.05%
**After pregnancy**	1,362	0.95%	1,232	0.96%	130	0.88%
**Smoking GP (mother)**						
**During pregnancy**	20,913	14.63%	18,720	14.60%	2,193	14.88%
**After pregnancy**	37,142	25.98%	33,477	26.11%	3,665	24.88%
**Substance misuse (any) SMD (mother)**						
**During pregnancy**	167	0.12%	149	0.12%	18	0.12%
**After pregnancy**	2,084	1.46%	1,823	1.42%	261	1.77%
**Substance misuse (other drug) PEDW (mother)**						
**During pregnancy**	272	0.19%	249	0.19%	23	0.16%
**After pregnancy**	1,355	0.95%	1,233	0.96%	122	0.83%
**Substance misuse (other drug) GP (mother)**						
**During pregnancy**	511	0.36%	459	0.36%	52	0.35%
**After pregnancy**	1,917	1.34%	1,705	1.33%	212	1.44%
**Assault PEDW (mother)**						
	572	0.40%	514	0.40%	58	0.39%
**Diabetes PEDW (child)**						
	212	0.15%	184	0.14%	28	0.19%
**Diabetes GP (child)**						
	199	0.14%	178	0.14%	21	0.14%
**Epilepsy PEDW (child)**						
	652	0.46%	554	0.43%	98	0.67%
**Epilepsy GP (child)**						
	916	0.64%	816	0.64%	100	0.68%
**Asthma PEDW (child)**						
	4,719	3.30%	4249	3.31%	470	3.19%
**Asthma GP (child)**						
	55,001	38.47%	49,468	38.58%	5,533	37.56%
**Ear PEDW (child)**						
	6,493	4.54%	5870	4.58%	623	4.23%
**Eye PEDW (child)**						
	3,836	2.68%	3432	2.68%	404	2.74%
**Any emergency hospital admission (child)**						
	80,588	56.37%	71282	55.59%	9306	63.16%
**Any A&E attendance (child)**						
	94,924	66.40%	84818	66.15%	10106	68.59%
**Learning Difficulty (child)**						
	1,290	0.90%	1132	0.88%	158	1.07%
**Low School readiness**						
**Did not achieve Foundation Phase**	20,468	14.32%	18,148	14.15%	2,320	15.75%
**Free school meal**						
	27,971	19.57%	24,587	19.18%	3,384	22.97%
**WIMD 2014—overall**						
**1 (most deprived)**	36,682	25.66%	32,237	25.14%	4,445	30.17%
**2**	30,647	21.44%	25,862	20.17%	4,785	32.48%
**3**	26,486	18.53%	24,531	19.13%	1,955	13.27%
**4**	22,283	15.59%	21,020	16.39%	1,263	8.57%
**5 (least deprived)**	26,857	18.79%	24,572	19.16%	2,285	15.51%
**Local area—urban/rural**						
**Rural town**	22,578	15.79%	18,592	14.50%	3,986	27.05%
**Rural village**	13,494	9.44%	13,418	10.46%	76	0.52%
**Urban city and town**	106,883	74.77%	96,212	75.04%	10,671	72.43%
**No of adult in the household**						
**1**	31,524	22.05%	27809	21.69%	3,715	25.22%
**2**	83,698	58.55%	75271	58.70%	8,427	57.20%
**3**	17,360	12.14%	15656	12.21%	1,704	11.57%
**4 or above**	10,373	7.26%	9486	7.40%	887	6.02%
**No of children in the household (excluding the cohort member)**						
**0**	23,706	16.58%	21,005	16.38%	2,701	18.33%
**1**	67,693	47.35%	60,491	47.18%	7,202	48.88%
**2**	33,989	23.78%	30,720	23.96%	3,269	22.19%
**3**	11,714	8.19%	10,614	8.28%	1,100	7.47%
**4 or above**	5,853	4.09%	5,392	4.21%	461	3.13%

Descriptive statistics of the study population stratified by their school readiness has been included as a supplementary file (please see Appendix 4 in S1 File).

#### Logistic regression results

Significant risk factors associated with low school readiness included: maternal learning difficulty (aOR (95% CI): 5.35 (3.97–7.22)), child epilepsy (2.95 (2.39–3.66)), having a very low birthweight (2.24 (1.86–2.70)), boys (2.11 (2.04–2.19)), being eligible for FSM (1.85 (1.78–1.93)), being extremely preterm (1.41 (1.04–1.91)), living in the most deprived area (1.67 (1.57–1.77)), not being breastfed (1.25 (1.21–1.30)), maternal death (1.47 (1.09–1.98)), maternal diabetes (1.46 (1.23–1.78)), smoking in pregnancy (1.36 (1.30–1.43)), child hospital admissions/illness for asthma (1.12 (1.03–1.22)), ear (1.36 (1.26–1.45)) and eye problems (1.30 (1.18–1.42)), single adult household (1.08 (1.04–1.12)), living with more than 3 children (1.63 (1.52–1.75)) in the household. The risk factors with their OR and upper and lower CI are presented in Table 2.

**Table 2:** Logistic regression model to identify the risk factors associated with low school readiness.

Variable name	OR	Lower CI	Upper CI	P value
**Gender**				
**Boy**	2.11	2.04	2.19	0.00000
**Gestational age (between 22 and 45)**				
**Extremely pre-term: <28 weeks**	1.41	1.04	1.91	0.02527
**Very pre-term: 28–31**	0.98	0.81	1.19	0.87621
**Pre-term: 32–36**	1.03	0.96	1.11	0.40224
**Late term: 43–45**	1.18	0.98	1.43	0.08157
**Unknown/NULL**	0.95	0.75	1.20	0.65316
**Birth weight (BW) (max 5000)**				
**Very low: <1500 g**	2.24	1.86	2.70	0.00000
**Low: 1500–<2500g**	1.55	1.44	1.67	0.00000
**High: 4000–5000g**	0.85	0.80	0.90	0.00000
**Unknown**	1.01	0.77	1.33	0.93332
**Breastfeeding**				
**No**	1.25	1.21	1.30	0.00000
**Unknown**	1.29	1.19	1.39	0.00000
**C-section birth**				
	1.00	0.96	1.04	0.97489
**Multiple birth**				
**Non-singleton**	0.94	0.84	1.04	0.20643
**Maternal age (between 10 and 65)**				
**Less than 19**	1.22	1.14	1.30	0.00000
**20–24**	1.14	1.09	1.20	0.00000
**25–29**	1.05	1.00	1.10	0.04689
**35 and above**	1.11	1.05	1.17	0.00035
**Unknown**	1.64	0.26	10.14	0.59509
**Death of mother**				
	1.47	1.09	1.98	0.01154
**Diabetes PEDW (mother)**				
	1.00	0.84	1.19	0.99550
**Diabetes GP (mother)**				
	1.46	1.23	1.74	0.00002
**Cancer PEDW (mother)**				
	0.99	0.74	1.33	0.95178
**Cancer GP (mother)**				
	0.81	0.59	1.12	0.20313
**Anaemia PEDW (mother)**				
	0.94	0.87	1.02	0.12312
**Anaemia GP (mother)**				
	1.00	0.95	1.05	0.87589
**Hypertension GP (mother)**				
	1.02	0.91	1.16	0.69703
**Learning Difficulty GP (mother)**				
	5.35	3.97	7.22	0.00000
**Depression PEDW (mother)**				
	1.06	0.98	1.15	0.13704
**Depression GP (mother)**				
	1.13	1.09	1.18	0.00000
**Anxiety PEDW (mother)**				
	1.06	0.95	1.18	0.28780
**Anxiety GP (mother)**				
	0.98	0.94	1.02	0.30464
**Anti Dep medication (mother)**				
	0.89	0.67	1.18	0.41647
**Serious Mental Illness PEDW (mother)**				
	1.00	0.81	1.24	0.97260
**Serious Mental Illness GP (mother)**				
	1.00	0.82	1.23	0.97759
**Alcohol PEDW (mother)**				
**During pregnancy**	1.44	0.97	2.13	0.07261
**After pregnancy**	0.98	0.84	1.13	0.74478
**Smoking GP (mother)**				
**During pregnancy**	1.36	1.30	1.43	0.00000
**After pregnancy**	1.29	1.24	1.34	0.00000
**Substance misuse (any) SMD (mother)**				
**During pregnancy**	1.00	0.67	1.49	0.99571
**After pregnancy**	1.17	1.03	1.32	0.01335
**Substance misuse (other drug) PEDW (mother)**				
**During pregnancy**	1.35	1.00	1.81	0.04916
**After pregnancy**	1.05	0.90	1.22	0.52958
**Substance misuse (other drug) GP (mother)**				
**During pregnancy**	1.31	1.04	1.64	0.02336
**After pregnancy**	1.05	0.93	1.20	0.42067
**Assault PEDW (mother)**				
	1.07	0.87	1.32	0.53618
**Diabetes PEDW (child)**				
	1.43	0.54	3.79	0.46615
**Diabetes GP (child)**				
	0.58	0.21	1.60	0.29455
**Epilepsy PEDW (child)**				
	2.09	1.62	2.71	0.00000
**Epilepsy GP (child)**				
	2.95	2.39	3.66	0.00000
**Asthma PEDW (child)**				
	1.12	1.03	1.22	0.00837
**Asthma GP (child)**				
	0.91	0.88	0.95	0.00000
**Ear PEDW (child)**				
	1.36	1.26	1.45	0.00000
**Eye PEDW (child)**				
	1.30	1.18	1.42	0.00000
**Any emergency hospital admission (child)**				
	1.09	1.05	1.13	1.09
**Any A&E attendance (child)**				
	1.02	0.98	1.06	1.02
**Free school meal**				
	1.85	1.78	1.93	0.00000
**Local authority**				
**Blaenau Gwent**	1.00	0.87	1.14	0.95948
**Bridgend**	0.86	0.75	0.98	0.02302
**Caerphilly**	1.14	1.01	1.28	0.04063
**Cardiff**	1			
**Carmarthenshire**	1.28	1.12	1.47	0.00032
**Ceredigion**	1.13	0.94	1.35	0.19523
**Conwy**	1.70	1.47	1.97	0.00000
**Denbighshire**	1.10	0.93	1.28	0.25936
**Flintshire**	1.27	1.10	1.46	0.00095
**Gwynedd**	1.26	1.09	1.46	0.00162
**Isle of Anglesey**	1.13	0.97	1.31	0.12881
**Merthyr Tydfil**	1.09	0.94	1.27	0.26314
**Monmouthshire**	1.08	0.89	1.30	0.44402
**Neath Port Talbot**	1.62	1.43	1.84	0.00000
**Newport**	0.79	0.69	0.91	0.00118
**Pembrokeshire**	1.12	0.95	1.33	0.17595
**Powys**	1.05	0.88	1.25	0.59866
**Rhondda Cynon Taff**	1.23	1.09	1.39	0.00078
**Swansea**	1.49	1.32	1.68	0.00000
**Torfaen**	0.96	0.83	1.12	0.62127
**Vale of Glamorgan**	1.02	0.88	1.17	0.81997
**Wrexham**	1.22	1.06	1.39	0.00419
**WIMD 2014—overallf**				
**1 (most deprived)**	1.67	1.57	1.77	0.00000
**2**	1.52	1.43	1.62	0.00000
**3**	1.37	1.29	1.46	0.00000
**4**	1.28	1.19	1.37	0.00000
**Local area—urban/rural**				
**Rural town**	1.08	1.03	1.13	0.00277
**Rural village**	1.20	1.12	1.29	0.00000
**No of adult in the household**				
**1**	1.08	1.04	1.12	0.00028
**3**	1.16	1.10	1.22	0.00000
**4 or above**	1.17	1.10	1.24	0.00000
**No of children in the household (excluding the cohort member)**				
**0**	1.08	1.03	1.13	0.00220
**2**	1.21	1.16	1.26	0.00000
**3**	1.43	1.35	1.52	0.00000
**4 or above**	1.63	1.52	1.75	0.00000
**Child’s age in the academic year**				
	0.31	0.30	0.33	0.00000
**School session absences**				
	1.02	1.02	1.02	0.00000
**Unauthorised absences**				
	1.00	0.99	1.00	0.00064
				

### Result from decision tree

**Fig 2:**
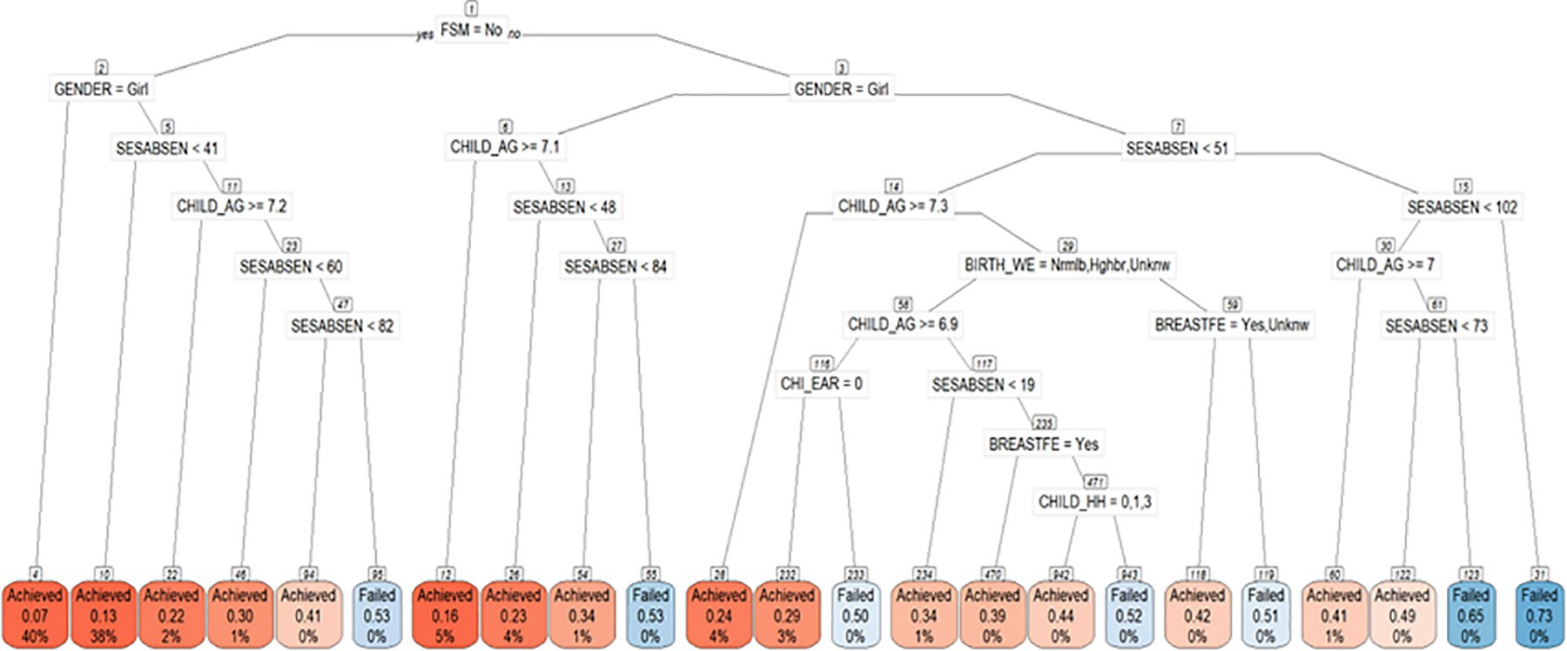
Decision tree for the children without learning difficulty.

The training model consisted of 127,090 individuals who lived in Wales (excluding testing dataset). The most important variables in the model were: FSM, gender (boy), number of school absences, child’s age while completing Foundation Phase, children with any emergency hospital admission, children with any A&E attendance, children with asthma, low birth weight, maternal substance misuse related GP record, maternal substance misuse related hospital admission, not being breastfed, children with ear problems and number of children in the household (higher number). The final decision tree model has been shown in Fig 2. Here are some case studies of the branches described in the decision tree model.

1. IF children are eligible for FSM (higher family level deprivation) -> Gender- Boys -> Total number of absent sessions more than 102 THEN the probability of Failed is 73% (terminal node 31)
2. IF children are not eligible for FSM (lower family level deprivation) -> Gender- Boys -> Younger in academic year ->Total number of absent sessions more than 82 THEN they are more likely to be Failed (terminal node 95).
3. IF children are eligible for FSM (higher family level deprivation) -> Gender- Girls -> Total number of absent sessions more than 84 THEN they are more likely to be Failed (terminal node 55).
4. IF children are eligible for FSM (higher family level deprivation) -> Gender- Boys -> Younger in academic year -> Low birth weight baby -> Not breastfed THEN they are more likely to be Failed (terminal node 119).
5. IF children are not eligible for FSM (lower family level deprivation) -> Gender- Girls THEN they are more likely to be Achieved (terminal node 4).
6. IF children are not eligible for FSM (lower family level deprivation) -> Gender- Girls -> Total number of absent sessions more than 41 THEN they are more likely to be Achieved (terminal node 10).

There were 14,575 children in the testing dataset. The model performance has been explained with the help of a confusion matrix. The model achieves 85.21% accuracy, 4.94% sensitivity, 99.37% specificity, 58.06% positive predictive values and 85.56% negative predictive value and 15% prevalence (see Tables 3 and 4).

**Table 3:** Confusion matrix/two by two table of the DT model.

Prediction	Reference (Children without learning difficulty) n = 14,575	
	Did not achieve (P)	Achieved (N)
Did not achieve(P)	108 (TP)	78 (FP)
Achieved (N)	2,078 (FN)	12,311 (TN)

**Table 4:** Prediction model performance (n = 14,575 children from Rhondda Cynon Taff).

	Accuracy	Sensitivity	Specificity	Positive Predictive Value	Negative Predictive Value	Prevalence
**Decision Tree**	85.21%	4.94%	99.37%	58.06%	85.56%	15%

## Discussion

This study investigated the risk factors associated with low school readiness and developed two holistic models on a national level routine data framework. Here the multivariable regression model helped to identify the risk factors with the highest association/Odds Ratio but might not be common or frequently observed on a population level, the decision tree on the other hand contributed to identify the most important and common/frequent risk factors. Infrequent but highly associated events/factors which affect a child’s school readiness include if the mother has a learning disability (0.14%), the child has epilepsy (0.64%) or is born extremely low birth weight (1%). However, there were also factors which were both highly associated and common such as being a boy (51%), where the odds of not being school ready is 2.11 than a girl (aOR more than twice that of girls), family level deprivation (eligible for FSM) which includes 19.5% of children, doubles the risk that they will not be school ready (aOR: 1.85). Low school attendance in early years (e.g., nursery) is associated with being 2% less likely to be school ready for every day missed in nursery.

The findings from our study suggest that rising poverty and the cost-of-living crisis are likely to result in lower school readiness and lower educational attainment. This will put a strain on school resources as children enter school [31]. Children in family and area-level deprivation are at higher risk of not being school ready. This finding is consistent with the existing literature [7,15]. Boys being disadvantaged compared to girls has been noted in other research [32]. In fact, it is suggested that family instability (separation, divorce, second families) affects boys more than girls, with a lack of a male influence impacting on behavioural difficulties [32] and that recent population increases in family instability can help explain a trend in lower attainment for boys at all levels. In addition, existing research clearly demonstrates that deprivation is a strong predictor of low school readiness [9,33]. Various indicators of deprivation such as parental employment, lower parental educational attainment, lower income, less time with the child, poorer play/local area facilities have been identified as significantly linked with low school readiness [7]. Our findings such as the significant association between living in family level (eligibility for FSM) and area level (most deprived WIMD) deprivation and higher chance not to be school ready are along the similar lines reported in the literature [9,34,35]. Hence, it is suggested that pre-school investment [35] and free childcare can overcome some of the risk factors associated with deprivation.

In this study, the decision tree model highlighted the risk factors which are clustered together e.g., boys living in household level deprivation and higher absences in school are at high risk of low school readiness. Similarly, girls who are often missing school are at risk of not being school ready. It also showed that children who are not breastfed, having ear infection and younger in academic year than their peers will more likely be not school ready. The branches of clustered risk factors are used to examine the determinants of low school readiness. These most significant risk factors can contribute to understand the profile of the vulnerable children and their families and help to improve the decision making at policy level which will support children to overcome the odds and have a better start in life.

This work has been developed as part of the Early Years Vulnerability Profiling Pilot. This will enable the Health Board and local authority to plan how the Early Years Vulnerability Profile can be used to inform better targeting of prevention and early intervention to children and their families up to the age of seven years to enable better outcomes for health, well-being, education, and social skills. The clustered risk factors can be used to understand what is associated with as determinants of low school readiness at population level. This can contribute to informed decision making at policy level that supports the children to have best start in life.

### Strengths and limitations

This study is based on linked data for an entire country over a 6-year period. This provides a wider range of risk factors from routine administrative data at a national level which can be addressed to improve outcomes for children who are exposed to inequalities and disadvantages from early life. It can contribute to breaking a cycle of disadvantage for children by helping to identify where and how to target early years interventions designed to improve school readiness. There is evidence that routinely collected data observed during perinatal period can contribute to improve child’s development at early years [15,21]. A linked population level database can facilitate a holistic investigation of the complex factors associated with the low school readiness. Longitudinal data linkage allows the capturing of the developmental trajectory of the individual child from school foundation phase and health visitor records. Combing this with maternal physical and mental health records can only strengthen the power of the analysis, as it is proved that maternal health and wellbeing is one of the biggest predictors of child’s development and wellbeing (*Improving school readiness Creating a better start for London*). If all these information can be available at an early stage to the policy makers from school and health visitors report, this can directly contribute to identify the most vulnerable children and their families at a very early stage and can help to build necessary intervention and support plans for them when it’s most needed.

However, it can only examine factors which are recorded using routine data. Important factors such as parenting style, time spent with the child reading, playing, and interacting cannot be captured with this data but would be important factors associated with school readiness. Another limitation of the study is that it only included the children who were in Wales during the entire study period and were removed if the children moved out of Wales as we were unable to capture their exposure records. However, this would not lead to any selection issues (please see Appendix 5 in S1 File) since these were arbitrary and independent events and does not lead to or is not linked with low school readiness. The study has identified a cluster of socioeconomic, health and household level risk factors leading to low school readiness and establishing a direct causal pathway of the modifiable risk factors is beyond the scope of the study.

A major strength of the study is that it incorporated data from birth till they enter their formal school to build the model to identify the risk factors of low school readiness, hence these findings can be helpful to identify the children at risk of low school readiness before they start their schooling as many of these factors are present in the first years of life (gender, deprivation, gestational age, parental health) and so those at risk can be supported through access to childcare, parenting support and supporting breastfeeding. In addition, the school readiness for local children coming to a school can be predicted and this means schools can have the necessary resources in place to help the specific catchment of children coming to their school.

### Conclusion

This study highlighted a vulnerability profile of the children who are at higher risk of low school readiness by identifying the group of risk factors which are clustered together. The findings suggest that earlier intervention (access to childcare, mother/baby groups, community activities, parenting interventions) could help to improve the outcomes for children who are at a high risk of low school readiness. This is especially true in deprived areas with low access to childcare and where there are child or adult health problems. It has been observed that intervention programmes like Flying Start has positive effects on the children living in deprivation including improved school attendance and better educational outcomes than their peers who are in similar condition but not under Flying Start programme [36]. This work suggests that interventions which focused on boys in deprived areas, encourage or facilitated attendance in nursery in the early years, investment in early years childcare and promoting breastfeeding would have a significant impact on school readiness. Interventions such as parenting programmes which supported families with parental learning difficulties, support when there is parental or child illness (e.g., community tutoring volunteer programmes) especially for epilepsy would make a significant difference for the child’s readiness for school. This could positively influence a child’s life trajectory by strengthening foundations for lifelong learning, improving health and wellbeing outcomes throughout the life-course, and reducing education and developmental inequalities that persist.

## Supporting information

S1 File (ZIP)
