# Supplementary material for: Factors associated with low school readiness, a linked health and education data study in Wales, UK
Source: PLoS One. 2023 Dec 11;18(12):e0273596. doi: 10.1371/journal.pone.0273596 (PMC10712842; doi:10.1371/journal.pone.0273596)
Supplement: S1 File — (ZIP) [file pone.0273596.s001.zip › Appendix1_Supplementary_SRNFactors_LitReview.docx]

# Appendix 1: Literature review on weighting of risk factors for low school readiness

# Aim

School readiness is a term to reflect if a child has the competency and capacity to achieve at the appropriate level in formal school. It is strongly linked to the child’s pre-school environment and includes language and communication skills, personal and social development, physical development, cognitive skills and basic knowledge (vocabulary). The main factors associated with school readiness relate to the family environment and include maternal education, income, maternal mental health, mother age, facilities in the local area and birth weight of infant. School readiness has been identified as a key public health concern in a recent review of UK public health systems and policy approaches to early child development ([Black et al 2019](https://academic.oup.com/jpubhealth/article/42/2/224/5364180)).

This study examines:

1. the risk factors associated with poor school readiness and
2. the relative weighting of importance of the risk factors in determining school readiness.

This includes:

1. The strength of the association and
2. The number/prevalence of infants exposed to each risk factor at birth, in RCT flying start and non-flying start areas and in Wales

# Method

## Literature review

*Search criteria:* The following databases will be searched, The Cochrane library, Web of Knowledge, Pubmed, EMBASE, CINAHL and Dissertation Abstracts International. Further references will be obtained by citation tracking, and by correspondence with appropriate experts. Data provided in published reports will be supplemented by information obtained by correspondence with authors. The search will be restricted to publications in English and in EU countries and publications between Jan 2000-April 2020.

*Selection criteria:* Observational studies including case controls, cohort studies and routine data linked studies with the primary or secondary outcomes examining school readiness.

*Data extracted:* The variables examined both those associated and not associated with school readiness will be extracted. The strength of association (odd ratio or coefficient) will be compared across studies.

| **Risk factor** | **Selected Risk size (OR or RR)** | **Paper and outcome** | **Interpretation and conclusion** | **Country, study and sample** |
| --- | --- | --- | --- | --- |
| **Child care** | Bayesian estimation multilevel modelling  **Literacy score:**  **County-level child care supply – the number of licensed child care slots per 100 children**  Slope: 0.480* (0.218)  *p<0.05 | [Lipscomb *et al* 2019](https://link.springer.com/article/10.1007/s11121-019-01002-8)  Direct measures of early literacy (easyCBM letter names and easyCBM letter sounds) and maths (easyCBM Numbers and Operations) test scores at Kindergarten entry. | A child’s literacy score improved significantly by half a point (0.48) for each additional county child care slot available per 100 children. | USA  Oregon Kindergarten Assessment  N=40,652 |
|  | Logistic regression - Odds ratio coefficients  **Probability of low vocabulary, reading and maths performance:**  **Low vocabulary (48 months):**  Child in child care centre ( excluding Head Start) >10 h/week: 0.46***  Child in Head Start >10 h/week: 0.52***  **Low reading performance (60 months)**  Child in child care centre (excluding Head Start) >10 h/week: 0.34***  Child in Head Start >10 h/week: 0.40***  **Low maths performance (60 months)**  Child in child care centre (excluding Head Start) >10 h/week: 0.46***  Child in Head Start >10 h/week: 0.40***  ***p<0.001 | [Scheffner Hammer *et al* 2016](https://www.ncbi.nlm.nih.gov/pmc/articles/PMC5962923/)    Low vocabulary (4 years) – Peabody Picture Vocabulary Test – bottom 10%  Low reading performance (5 years) – 74 item reading test assessing basic skills - – bottom 10%  Low maths performance (5 years) – 42 item mathematics test – bottom 10% | A child attending child care had significantly lower odds of low vocabulary performance (child care 0.46, Head Start 0.52), low reading performance (child care 0.34, Head Start 0.40) and low maths performance (child care 0.46, Head Start 0.40) than a child not enrolled in child care. | USA  Early Childhood Longitudinal Study (ECLS-B)  N=6,050 |
|  | Linear probability model (OLS)  Coefficient (std error)  Probability of child being fully school ready compared to child in centre-based care: **Family child care:**  Language/literacy: -0.063*** (0.014)  Maths: -0.060*** (0.013)  **Informal child care:**  Language/literacy: -0.049*** (0.018)  Maths: -0.072*** (0.018)  Probability of child being fully school ready compared to child not in pre-kindergarten:  **Enrolled in pre-kindergarten:**  Language/literacy: 0.132*** (0.012)  Maths: 0.127*** (0.012)  ***p<0.001 | [Forry *et al* 2013](https://www.sciencedirect.com/science/article/pii/S0885200613000367)  Language and literacy.  Mathematics.  Binary indicator – probability of child being fully school ready compared to child not enrolled in child care. | Compared to children in centre-based care, those in family child care and informal child care were less likely to be assessed as fully ready in language/literacy and maths.  Children enrolled in pre-kindergarten were more likely to be assessed as fully ready in language/literacy and maths than those not enrolled in pre-kindergarten. | USA  Maryland Model for School Readiness (MMSR) – teacher assessment of seven domains.  N=6,772 |
| **Unemployment** | Bayesian estimation multilevel modelling  **County-level unemployment. % of adults (16+) not employed:**  Early maths score slope: -0.213*  Early literacy score slope: -1.49*  *p<0.05 | [Lipscomb *et al* 2019](https://link.springer.com/article/10.1007/s11121-019-01002-8)  Direct measures of early literacy (easyCBM letter names and easyCBM letter sounds) and maths (easyCBM Numbers and Operations) test scores at Kindergarten entry. | For each percentage increase in unemployment, a child’s maths score decreased by -0.213 points and literacy score decreased by -1.49 points. | USA  Oregon Kindergarten Assessment  N=40,652 |
|  | Predictive Risk Model (PRM). Dominance analysis to rank and weight predictor variables.  **Odds of being not school ready (both parents in work reference group:**  **Unadjusted:**  **One parent in work:** 1.79 (1.49 to 2.14)  **Neither parents in work:** 5.39 (4.36 to 6.67)  **Adjusted:**  **One parent in work:** 0.82 (0.67 to 1.00)  **Neither parents in work:** 1.21 (0.87 to 1.68)  Weighting (rank): 6.9 (7) | [Camacho *et al* 2019](https://bmjopen.bmj.com/content/9/6/e024851)  School readiness measured using the Bracken School Readiness Assessment (BSRA) at age 3.  School ready binary indicator.  Children classified as not school ready (delayed or very delayed BSRA score) or school ready (average, advanced, very advanced BSRA score). | Using a PRM, parental employment status was included as one of 13 final predictor variables of a child being assessed as not school ready at age 3. Lower parental employment status was negatively associated with not being school ready. | UK  Millennium Cohort Study  N=9,487 |
| **Housing** | Predictive Risk Model (PRM). Dominance analysis to rank and weight predictor variables.  **Odd of being not school ready (owner occupied reference group):**  **Unadjusted:**  Private rented: 2.68 (2.16 to 3.33)  Social housing: 3.89 (3.34 to 4.53)  Other**:** 2.65 (2.10 to 3.35)  **Adjusted:**  Private rented: 1.21 (0.87 to 1.67)  Social housing: 1.45 (1.16 to 1.81)  Other**:** 0.9 (0.62 to 1.30)  Weighting (rank): 5.7 (8) | [Camacho *et al* 2019](https://bmjopen.bmj.com/content/9/6/e024851)  School readiness measured using the Bracken School Readiness Assessment (BSRA) at age 3.  School ready binary indicator.  Children classified as not school ready (delayed or very delayed BSRA score) or school ready (average, advanced, very advanced BSRA score).  Predictor variables collected at age 9 months. | Using a PRM, housing tenure was included as one of 13 final predictor variables of a child being assessed as not school ready at age 3.  Living in rented or social housing was negatively  associated with not being school ready. | UK  Millennium Cohort Study  N=9,487 |
|  | Prediction model of odds of low academic scores at kindergarten entry (age 5-6)  **Parental rating of housing quality for raising children – good, fair or poor (excellent or very good rating reference group):**  Unadjusted 2.2 (1.8–2.7)  Adjusted 1.4 (1.1–1.8)  Risk Index Weight: 6.7  Risk Index Weight (= β/5.3 × 100): represent a range from 0 (lowest risk) to 100 (highest risk) | [Nelson *et al* 2016](https://pediatrics.aappublications.org/content/138/2/e20154477)  Direct measures of early literacy and maths – adapted from Peabody picture vocabulary and PreLAS 2000.  Poor academic school readiness defined as ≥1 SD below mean in early literacy and mathematics tests.  Predictor variables measured at age 2. | A low parent rating of quality of housing for raising children was associated with a higher odds of poor academic school readiness. | USA  Early Childhood Longitudinal Study  ECLS-B  N=6,350 |
| **Drinking alcohol during pregnancy** | Z-score regression coefficients (95% CI)  **British Ability Scale**  **Boys**  Fully adjusted model:  None – reference group  Light: 0.07 (-0.01 to 0.15)  Moderate: 0.14 (0.02 to 0.25)  Heavy/binge: 0.02 (-0.19 to 0.24)  **Girls**  Fully adjusted model:  None – reference group  Light: -0.04 (-0.11 to 0.03)  Moderate: 0.01 (-0.14 to 0.16)  Heavy/binge: -0.20 (-0.41 to 0.01)  **Bracken School Readiness Assessment:**  **Boys**  Fully adjusted model  None – reference group  Light: 0.11 (0.03 to 0.19)  Moderate: 0.08 (-0.05 to 0.22)  Heavy/binge: 0.11 (-0.12 to 0.34)  **Girls**  Fully adjusted model  None – reference group  Light: -0.04 (-0.11 to 0.03)  Moderate: 0.00 (-0.15 to 0.15)  Heavy/binge: -0.25 (-0.49 to -0.01) | [Kelly *et al* 2009](https://pubmed.ncbi.nlm.nih.gov/18974425/)  British Ability Scale (BAS) and the Bracken School Readiness Assessment (BSRA) at age 3.  Z-scores  Fully adjusted models – mother and infant factors, socio-economic markers, family psycho-social environment |  | UK  Millennium Cohort Study  N=9,154 (BAS)  N=8,775 (BSRA) |
| **Mother’s age** | Predictive Risk Model (PRM). Dominance analysis to rank and weight predictor variables.  **Odds of not being school ready - Mother’s age at birth of first child (30-39 reference group)**  Unadjusted OR (95% CI):  **40+:** 2.83 (2.29 to 3.49)  **20-29:** 5.57 (4.20 to 7.37)  **14-19:** 6.02 (4.84 to 7.48)    Adjusted OR (95% CI):  **40+:** 1.05 (0.68 to 1.63)  **20-29:** 1.28 (0.98 to 1.66)  **14-19:** 1.32 (0.95 to 1.83)  Weighting (rank): 2.9 (11) | [Camacho *et al* 2019](https://bmjopen.bmj.com/content/9/6/e024851)  School readiness measured using the Bracken School Readiness Assessment (BSRA) at age 3.  School ready binary indicator.  Children classified as not school ready (delayed or very delayed BSRA score) or school ready (average, advanced, very advanced BSRA score).  Predictor variables collected at age 9 months. | Using a PRM, mother’s age at birth of first child was included as one of 13 final predictor variables of a child being assessed as not school ready at age 3.  Younger parents at the birth of their first child were more likely to have children classified as not school ready. | UK  Millennium Cohort Study  N=9,487 |
|  | Logistic regression  **Teen mother at first birth**  .69*** (positive comprehensive development profile)  ***p<0.001 | [Hair *et al*. 2006](https://www.sciencedirect.com/science/article/pii/S0885200606000640)  Positive comprehensive development profile – score above mean in health, social/emotional, language, cognitive domains | Teenage mothers during their first birth were less likely to have children with a positive comprehensive development profile | USA  Early Childhood Longitudinal Study (ECLS-K)  N=17,219 |
|  | Logistic regression - Odds ratio coefficients  **Odds of low vocabulary - Mother >35 years at birth**: 1.48*  *p<0.05 | [Scheffner Hammer *et al* 2016](https://www.ncbi.nlm.nih.gov/pmc/articles/PMC5962923/)  Low vocabulary (4 years) – Peabody Picture Vocabulary Test – bottom 10% | Older maternal age at birth was significantly associated with a higher risk of low vocabulary at age 4. | USA  Early Childhood Longitudinal Study (ECLS-B)  N=6,050 |
|  | Logistic regression – Odds ratio coefficients  **Odds of child learning delay – Mother’s age first birth (<20 reference group):**  20-24: 0.84*  25-29: 0.65***  30-34: 0.41***  35+: 0.59**  *p<0.05, **p<0.01, ***p<0.001 | [Kiernan and Mensah (2009)](https://www.cambridge.org/core/journals/journal-of-social-policy/article/poverty-maternal-depression-family-status-and-childrens-cognitive-and-behavioural-development-in-early-childhood-a-longitudinal-study/246EDA5324B918B737369A7B01E358BA)  Bracken Basic Concept Scale (BBCS)  Raw scores grouped into 5 categories  (very delayed, delayed, average, advanced, very advanced)  ‘Learning delay’ binary indicator – delayed/very delayed | Mother’s age at first birth was significantly associated with cognitive learning delay. | UK  Millennium Cohort Study  N=13,233 |
|  | Predictive risk model  Relative risk (95% CI) of developmental vulnerability:  **Mother’s age:**  Males: 0.98 (0.96-0.99)  Females: 0.99 (0.97-1.01) | [Chittleborough *et al* 2016](https://www.sciencedirect.com/science/article/pii/S0885200615300119)  Australian Early Development Index (AEDI) [(more info)](https://pubmed.ncbi.nlm.nih.gov/24771275/)  AEDI domains – physical health and wellbeing, social competence, emotional maturity, language and cognitive skills, communication skills and general knowledge  Outcome – children vulnerable in ≥2 developmental domains | Mother’s age (<20) included as one of six final predictive variables of children’s poor development. | Australia  Australian Early Development Consensus  N=13,827 |
| **Breastfed** | Predictive Risk Model (PRM). Dominance analysis to rank and weight predictor variables.  **Odds of not being school ready – breastfeeding duration (6+ months reference group)**  Adjusted:  **6 months – 6 weeks:** 1.05 (1.02 to 1.53)  **1 week or less:** 1.19 (1.34 to 2.09)  **1-6 weeks:** 1.25 (0.96 to 1.65)  **Never:** 1.49 (1.19 to 1.87)  Unadjusted:  **6 months – 6 weeks:** 1.05 (0.81 to 1.36)  **1 week or less:** 1.19 (0.89 to 1.59)  **1-6 weeks:** 1.68 (1.36 to 2.07)  **Never:** 2.74 (2.29 to 3.27)  Weighting (rank): 3.9 (10) | [Camacho *et al* 2019](https://bmjopen.bmj.com/content/9/6/e024851)  School readiness measured using the Bracken School Readiness Assessment (BSRA) at age 3.  School ready binary indicator.  Children classified as not school ready (delayed or very delayed BSRA score) or school ready (average, advanced, very advanced BSRA score).  Predictor variables collected at age 9 months. | Using a PRM, breastfeeding duration was included as one of 13 final predictor variables of a child being assessed as not school ready at age 3.  Never breastfeeding was associated with not being school ready | UK  Millennium Cohort Study  N=9,487 |
|  | Logistic regression – Odds ratio coefficients  **Odds of learning delay (never breastfed reference group):**  <6 months: 0.75***  >6 months: 0.56***  ***p<0.001 | [Kiernan and Mensah (2009)](https://www.cambridge.org/core/journals/journal-of-social-policy/article/poverty-maternal-depression-family-status-and-childrens-cognitive-and-behavioural-development-in-early-childhood-a-longitudinal-study/246EDA5324B918B737369A7B01E358BA)  Bracken Basic Concept Scale (BBCS)  Raw scores grouped into 5 categories  (very delayed, delayed, average, advanced, very advanced)  ‘Learning delay’ binary indicator – delayed/very delayed | Never breastfeeding was significantly associated with cognitive learning delay. | UK  Millennium Cohort Study  N=13,233 |
| **Parental physical health** | Prediction model of odds of low academic scores at kindergarten entry (age 5-6)  **Fair or poor parental health (good to excellent reference group):**  Unadjusted 3.0 (2.3–4.0)  Adjusted 1.5 (1.1–2.1)  Risk Index Weight: 8.2  Risk Index Weight (= β/5.3 × 100): represent a range from 0 (lowest risk) to 100 (highest risk) | [Nelson *et al* 2016](https://pediatrics.aappublications.org/content/138/2/e20154477)  Direct measures of early literacy and maths – adapted from Peabody picture vocabulary and PreLAS 2000.  Poor academic school readiness defined as ≥1 SD below mean in early literacy and mathematics tests.  Predictor variables measured at age 2. | A low rating of parental health or raising children was associated with a higher odds of poor academic school readiness. | USA  Early Childhood Longitudinal Study  ECLS-B  N=6,350 |
|  | Logistic regression – Odds ratio coefficients  **Odds of FSP lowest decile - maternal health status (excellent/good both surveys reference group)**  Fair/poor 9 month survey only: 2.7***  Fair/poor age 3 survey only: 3.7***  Fair/poor at both surveys: 4.4***  ***p=0.000 | [Hobcraft and Kiernan 2010](https://www.york.ac.uk/media/spsw/documents/research-and-publications/HobcraftKiernan2010PredictiveFactorsChildrensDevelopmentMillenniumCohort.pdf)  Learning and development assessed through ‘Foundation Stage Profile’ during child’s first year of primary school  Outcome – FSP lowest decile | Lower parental reported health status was statistically associated with a higher odds of their child being in the lowest decile of the FSP. | UK  Millennium Cohort Study  N=8430 |
|  | **Relative risk (95% CI)**  **Mother had anaemia:**  Males: 1.20 (1.01–1.44) Females: 1.27 (0.95–1.69)  **Mother had hypertension:**  Males: 1.30 (1.09–1.56)  Females: 0.86 (0.59–1.25)  **Mother had diabetes:** Males: 1.18 (0.92–1.51)  Females: 1.19 (0.79–1.79)  **Antepartum haemorrhage:**  Males: 1.35 (1.09–1.68)  Females: 1.11 (0.69–1.78)  **Procedures in pregnancy:**  Males: 0.92 (0.73–1.16)  Females: 1.14 (0.79–1.66)  **Emergency caesarean:**  Males: 1.13 (0.98–1.30)  Females: 0.89 (0.67–1.19) | [Chittleborough *et al* 2016](https://www.sciencedirect.com/science/article/pii/S0885200615300119)  Australian Early Development Index (AEDI) [(more info)](https://pubmed.ncbi.nlm.nih.gov/24771275/)  AEDI domains – physical health and wellbeing, social competence, emotional maturity, language and cognitive skills, communication skills and general knowledge  Outcome – children vulnerable in ≥2 developmental domains | Perinatal variables included in initial multivariate regression models but not included in final predictive risk model. | Australia  Australian Early Development Consensus  N=13,827 |
|  | Linear regression predicting child reading and maths ability - b (SE)  **Mother’s pre-pregnancy BMI**  Reading: -0.19 (0.08)*  Maths: -0.13 (0.06)*  *p<0.05 | [Larson *et al* 2015](https://pediatrics.aappublications.org/content/135/2/e440)  Direct cognitive assessment of early reading and maths scores using Peabody Picture Vocabulary Test and PreLAS 2000 at kindergarten entry (age 5-6) | Mother’s reported pre-pregnancy BMI had a negative association with children’s reading and maths ability at 5-6 years. | USA  Early Childhood Longitudinal Study (ECLS-B)  N=6,600 |
| **Parental mental health** | Predictive Risk Model (PRM). Dominance analysis to rank and weight predictor variables.  **Odds of not being school ready - Diagnosed maternal depression/anxiety (no depression/anxiety reference group):**  Unadjusted 1.33 (1.16 to 1.53)  Adjusted 1.28 (1.07 to 1.53)  Weighting (rank): 0.4 (13) | [Camacho *et al* 2019](https://bmjopen.bmj.com/content/9/6/e024851)  School readiness measured using the Bracken School Readiness Assessment (BSRA) at age 3.  School ready binary indicator.  Children classified as not school ready (delayed or very delayed BSRA score) or school ready (average, advanced, very advanced BSRA score).  Predictor variables collected at age 9 months. | Using a PRM, diagnosed maternal depression/anxiety was included as one of 13 final predictor variables of a child being assessed as not school ready at age 3.  Younger parents at the birth of their first child were more likely to have children classified as not school ready. | UK  Millennium Cohort Study  N=9,487 |
|  | Logistic regression – Odds ratio coefficients  **Odds of FSP lowest decile - maternal depression (no depression reference group):**  Depression at 9 month survey only: 1.9**  Depression at age 3 survey only: 1.9***  Both surveys: 3.0***  **p=0.001  ***p=0.000 | [Hobcraft and Kiernan 2010](https://www.york.ac.uk/media/spsw/documents/research-and-publications/HobcraftKiernan2010PredictiveFactorsChildrensDevelopmentMillenniumCohort.pdf)  Learning and development assessed through ‘Foundation Stage Profile’ during child’s first year of primary school  Outcome – FSP lowest decile | Children of mothers with depression had statistically higher odds of being in the lowest decile of the FSP. | UK  Millennium Cohort Study  N=8430 |
|  | Logistic regression – Odds ratio coefficients  **Maternal depression (no depression reference group):**  Unadjusted:  Depression at 9 month survey only: 1.38**  Depression at age 3 survey only: 1.73***  Both surveys: 2.06***  Adjusted for poverty, family status and background factors:  Depression at 9 month survey only: 1.13  Depression at age 3 survey only: 1.27*  Both surveys: 1.21  *p<0.05  **p<0.01  ***p<0.001 | [Kiernan and Mensah (2009)](https://www.cambridge.org/core/journals/journal-of-social-policy/article/poverty-maternal-depression-family-status-and-childrens-cognitive-and-behavioural-development-in-early-childhood-a-longitudinal-study/246EDA5324B918B737369A7B01E358BA)  Bracken Basic Concept Scale (BBCS)  Raw scores grouped into 5 categories  (very delayed, delayed, average, advanced, very advanced)  ‘Learning delay’ binary indicator – delayed/very delayed | There was a weak association between maternal depression and cognitive learning delay. Adjusting for family background and poverty attenuates this association.  The measures of depression at both surveys were not identical. | UK  Millennium Cohort Study  N=13,233 |
|  | Linear regression predicting child reading and maths ability (b SE)  **Maternal depression:**  Reading: -0.11 (0.11)  Maths: -0.26 (0.09)*  *p<0.05 | [Larson *et al* 2015](https://pediatrics.aappublications.org/content/135/2/e440)  Direct cognitive assessment of early reading and maths scores using Peabody Picture Vocabulary Test and PreLAS 2000 at kindergarten entry (age 5-6) | Maternal depression had a significant association with maths scores but not reading. | USA  Early Childhood Longitudinal Study (ECLS-B)  N=6,600 |
| **Family structure** | Predictive Risk Model (PRM). Dominance analysis to rank and weight predictor variables.  **Odds of not being school ready – number of children in family (one child reference group):**  **Unadjusted:**  **2-3 children:** 1.44 (1.27 to 1.63)  **4+ children:** 3.71 (3.04 to 4.54)  **Adjusted:**  **2-3 children:** 1.38 (1.15 to 1.66)  **4+ children:** 2.67 (1.94 to 3.68)  Weighting (rank): 7.8 (6) | [Camacho *et al* 2019](https://bmjopen.bmj.com/content/9/6/e024851)  School readiness measured using the Bracken School Readiness Assessment (BSRA) at age 3.  School ready binary indicator.  Children classified as not school ready (delayed or very delayed BSRA score) or school ready (average, advanced, very advanced BSRA score).  Predictor variables collected at age 9 months. | Using a PRM, the number of children in the family was included as one of 13 final predictor variables of a child being assessed as not school ready at age 3.  Having more children in the family was associated with higher odds of not being school ready. | UK  Millennium Cohort Study  N=9,487 |
|  | Logistic regression  **Family type (Two biological/adoptive parents reference group):**  1 biological, 1 step parent: .74***  1 biological or other parent: .77***  ***p<0.001 | [Hair *et al*. 2006](https://www.sciencedirect.com/science/article/pii/S0885200606000640)  Positive comprehensive development profile – score above mean in health, social/emotional, language, cognitive domains | Family type was significantly associated with children’s school readiness. Compared to children from two biological/adoptive parents, those from single parent families were less likely to have a positive comprehensive development profile. | USA  Early Childhood Longitudinal Study (ECLS-K)  N=17,219 |
|  | Logistic regression OR (95% CI)  **Odds of child being vulnerable in school readiness – Nonintact family (intact family reference group)**  1.834 (1.385 – 2.427)  P<0.001    Intact family - married or common-law couples in which all children are the natural and/or adopted offspring of both members of the couple | [Janus and Duku 2007](https://www.tandfonline.com/doi/abs/10.1080/10409280701610796a)  Early Development Indicator (EDI) – teacher assessment of physical health and well-being, social competence, emotional maturity, language and cognitive development, communication skills and general knowledge  Developmental vulnerability binary indicator – scoring in the lowest 10^th^ percentile in ≥ one domain. |  | USA  Longitudinal Survey of Children and Youths  N=2,196 |
|  | Logistic regression – Odds ratio coefficients  **Odds of FSP lowest decile – parental separation occurring between 9 months and age 3:**  2.41***  ***p=0.000 | [Hobcraft and Kiernan 2010](https://www.york.ac.uk/media/spsw/documents/research-and-publications/HobcraftKiernan2010PredictiveFactorsChildrensDevelopmentMillenniumCohort.pdf)  Learning and development assessed through ‘Foundation Stage Profile’ during child’s first year of primary school  Outcome – FSP lowest decile | Children whose parents separated between the ages of 9 months and 3 years had significantly higher odds of being in the lowest decile of the FSP. | UK  Millennium Cohort Study  N=8430 |
|  | Logistic regression – Odds ratio coefficients  **Odds of child learning delay by family status (married – reference group)**  (Adjusted for poverty, maternal depression and background factors)  **Family status at 3 years:**  Cohabiting: 1.16  Lone parent: 1.10  Step family: 1.54*  *p<0.05 | [Kiernan and Mensah (2009)](https://www.cambridge.org/core/journals/journal-of-social-policy/article/poverty-maternal-depression-family-status-and-childrens-cognitive-and-behavioural-development-in-early-childhood-a-longitudinal-study/246EDA5324B918B737369A7B01E358BA)  Bracken Basic Concept Scale (BBCS)  Raw scores grouped into 5 categories  (very delayed, delayed, average, advanced, very advanced)  ‘Learning delay’ binary indicator – delayed/very delayed | The odds of cognitive learning delay were significantly higher in children from step parent families. | UK  Millennium Cohort Study  N=13,233 |
|  | Logistic regression – Odds ratio coefficients  **Odds of child learning delay by number of siblings (one child reference group)**  (Adjusted for poverty, maternal depression and background factors)  One sibling: 1.33***  Two siblings: 1.77***  3+ siblings: 2.00***  ***p<0.001 | [Kiernan and Mensah (2009)](https://www.cambridge.org/core/journals/journal-of-social-policy/article/poverty-maternal-depression-family-status-and-childrens-cognitive-and-behavioural-development-in-early-childhood-a-longitudinal-study/246EDA5324B918B737369A7B01E358BA)  Bracken Basic Concept Scale (BBCS)  Raw scores grouped into 5 categories  (very delayed, delayed, average, advanced, very advanced)  ‘Learning delay’ binary indicator – delayed/very delayed | Children from families with more siblings had significantly higher odds of having cognitive learning delay. | UK  Millennium Cohort Study  N=13,233 |
|  | Predictive risk model  **Relative risk (95% CI) of developmental vulnerability - Never married, widowed, divorced or separated (mother married reference group):**  Males: 1.28 (1.12–1.47)  Females: 1.32 (1.03–1.68) | [Chittleborough *et al* 2016](https://www.sciencedirect.com/science/article/pii/S0885200615300119)  Australian Early Development Index (AEDI) [(more info)](https://pubmed.ncbi.nlm.nih.gov/24771275/)  AEDI domains – physical health and wellbeing, social competence, emotional maturity, language and cognitive skills, communication skills and general knowledge  Outcome – children vulnerable in ≥2 developmental domains | Mother’s marital status included as one of six final risk factors for poor childhood development | Australia  Australian Early Development Consensus  N=13,827 |
|  | Predictive risk model  **Relative risk (95% CI) of developmental vulnerability - Number of previous pregnancies (≥20 weeks) (none – reference group):**  **One:**  Males: 1.07 (0.93–1.24)  Females: 0.96 (0.75–1.24)  **Two:**  Males: 1.27 (1.06–1.51)  Females: 1.30 (0.96–1.75)  **Three or more:** Males: 1.45 (1.17–1.80)  Females: 1.54 (1.08–2.20) | [Chittleborough *et al* 2016](https://www.sciencedirect.com/science/article/pii/S0885200615300119)  Australian Early Development Index (AEDI) [(more info)](https://pubmed.ncbi.nlm.nih.gov/24771275/)  AEDI domains – physical health and wellbeing, social competence, emotional maturity, language and cognitive skills, communication skills and general knowledge  Outcome – children vulnerable in ≥2 developmental domains | Number of previous pregnancies (≥20 weeks) included as one of six final risk factors for poor childhood development. | Australia  Australian Early Development Consensus  N=13,827 |
| **Family history of learning disability** | Prediction model of odds of low academic scores at kindergarten entry (age 5-6)  **Family history of learning disability:**  Unadjusted 1.6 (1.3–2.1)  Adjusted 1.6 (1.2–2.1)  Risk Index Weight: 9.2  Risk Index Weight (= β/5.3 × 100): represent a range from 0 (lowest risk) to 100 (highest risk) | [Nelson *et al* 2016](https://pediatrics.aappublications.org/content/138/2/e20154477)  Direct measures of early literacy and maths – adapted from Peabody picture vocabulary and PreLAS 2000.  Poor academic school readiness defined as ≥1 SD below mean in early literacy and mathematics tests.  Predictor variables measured at age 2. | A low rating of parental health or raising children was associated with a higher odds of poor academic school readiness. | USA  Early Childhood Longitudinal Study  ECLS-B  N=6,350 |
|  | Logistic regression – odds ratio coefficient  **Odds of low reading and maths performance - Family member with learning disability:**  1.78** (Low reading performance 5 years)  1.80*** (Low maths performance 5 years)  **p<0.01  ***p<0.001 | [Scheffner Hammer *et al* 2016](https://www.ncbi.nlm.nih.gov/pmc/articles/PMC5962923/)    Low reading performance (5 years) – 74 item reading test assessing basic skills - – bottom 10%  Low maths performance (5 years) – 42 item mathematics test – bottom 10% | A with a family member with a learning disability had significantly higher odds of low reading and maths performance at age 5. | USA  Early Childhood Longitudinal Study (ECLS-B)  N=6,050 |
| **Parent smoking** | -.024 (early maths)  -.191 (early literacy) | [Lipscomb *et al* 2019](https://link.springer.com/article/10.1007/s11121-019-01002-8) | Early literacy and maths tests – area level factors  Bayesian estimation multi level modelling | Oregon Kindergarten Assessment, USA |
|  | 1.292 (0.987 – 1.690) | [Janus and Duku 2007](https://www.tandfonline.com/doi/abs/10.1080/10409280701610796a) | Early Development Indicator – physical health and well-being, social competence, emotional maturity, language and cognitive development, communication skills and general knowledge | Canada - Community Component of the National Longitudinal Survey of Children and Youth |
|  | Smoked in first 3 months of pregnancy  1.29 (1.07–1.56) | [Chittleborough *et al* 2011](https://pubmed.ncbi.nlm.nih.gov/21536608/) | School Entry Assessment (language, reading, writing, maths) – total SEA score.  Poor child development – scoring in lowest 10% of SEA | UK - The Avon Longitudinal Study of Parents and Children (ALSPAC) |
|  | Smoked in second half of pregnancy  Males: 1.43 (1.25–1.63)  Females: 1.27 (1.01–1.60)  Smoked in second half of pregnancy included as one of six final risk factors for poor child development outcomes | [Chittleborough *et al* 2016](https://www.sciencedirect.com/science/article/pii/S0885200615300119) | Australian Early Development Index (AEDI) [(more info)](https://pubmed.ncbi.nlm.nih.gov/24771275/)  AEDI domains – physical health and wellbeing, social competence, emotional maturity, language and cognitive skills, communication skills and general knowledge  Outcome – children vulnerable in ≥2 developmental domains | Australia – Australian Early Development Consensus |
| **Parenting quality/style** | Logistic regression – odds ratio coefficient  **Odds of having low vocabulary, reading or maths performance – parenting score:**  0.74*** (Low vocabulary 4 years)  0.76 (Low reading performance 5 years)  0.76* (low maths performance 5 years)  *p<0.05  ***p<0.001 | [Scheffner Hammer *et al* 2016](https://www.ncbi.nlm.nih.gov/pmc/articles/PMC5962923/)    Low vocabulary (4 years) – Peabody Picture Vocabulary Test – bottom 10%  Low reading performance (5 years) – 74 item reading test assessing basic skills - – bottom 10%  Low maths performance (5 years) – 42 item mathematics test – bottom 10% | A higher parenting quality (average score of two parenting assessments at 24 months) was significantly associated with a lower odds of low vocabulary and maths performance. Low reading was statistically significant. | USA  Early Childhood Longitudinal Study (ECLS-B)  N=6,050 |
|  | Linear regression predicting child reading and maths ability (b SE)  **Positive interactions**  Reading: 0.25 (0.10)*  Maths: 0.22 (0.09)*  **Parent supportiveness**  Reading: 1.04 (0.60) Maths: 1.62 (0.60)*  **Rules about food**  Reading: 2.95 (1.13)*  Maths: 3.93 (1.14)*  ≥**2 classes/activities at age 4**  Reading: 2.52 (1.03)*  Maths: 3.44 (1.19)*  *p<0.05 | [Larson *et al* 2015](https://pediatrics.aappublications.org/content/135/2/e440)  Direct cognitive assessment of early reading and maths scores using Peabody Picture Vocabulary Test and PreLAS 2000 at kindergarten entry (age 5-6) | A number of measures of parenting style and beliefs (positive interactions, parent supportiveness, rules about food, were significantly associated with children’s reading and maths performance. | USA  Early Childhood Longitudinal Study (ECLS-B)  N=6,600 |
| **Parental expectations** | Prediction model of odds of low academic scores at kindergarten entry (age 5-6)  **Lower parental expectations of child’s education**  **Logistic Regression**  Unadjusted 2.2 (1.8–2.8)  Adjusted 1.3 (1.0–1.7)  Risk Index Weight: 5.1  Risk Index Weight (= β/5.3 × 100): represent a range from 0 (lowest risk) to 100 (highest risk) | [Nelson *et al* 2016](https://pediatrics.aappublications.org/content/138/2/e20154477)  Direct measures of early literacy and maths – adapted from Peabody picture vocabulary and PreLAS 2000.  Poor academic school readiness defined as ≥1 SD below mean in early literacy and mathematics tests.  Predictor variables measured at age 2. | was associated with a higher odds of poor academic school readiness. | USA  Early Childhood Longitudinal Study  ECLS-B  N=6,350 |
|  | **Expects child to earn college degree**  Linear regression  B (SE)  Reading: 5.27 (1.11)* Maths: 3.87 (1.02)*  *p<0.05 | [Larson *et al* 2015](https://pediatrics.aappublications.org/content/135/2/e440) | Direct cognitive assessment.  Early reading and maths scores | Early Childhood Longitudinal Birth Cohort Study |
| **Home learning environment** | Logistic Regression  Prediction model of odds of low academic scores at kindergarten entry (age 5-6)  Frequency of shared reading (every day – reference group)  **3-6 times/week:**  Unadjusted 1.7 (1.3–2.2)  Adjusted 1.3 (1.0–1.8)  **1-2 times/week:**  Unadjusted 2.8 (2.2–3.6)  Adjusted 1.6 (1.2–2.1)  **Not at all:**  Unadjusted 4.6 (2.9–7.1)  Adjusted 2.1 (1.3–3.5)  Risk Index Weight:  3-6 times/week: 5.4  1-2 times/week: 8.4  Not at all: 14.1  Risk Index Weight (= β/5.3 × 100): represent a range from 0 (lowest risk) to 100 (highest risk) | [Nelson *et al* 2016](https://pediatrics.aappublications.org/content/138/2/e20154477) – poor school readiness | Poor school readiness >=1 SD below mean in early literacy and mathematics tests | USA - Early Childhood Longitudinal Study |
|  | Multivariate analysis of variance (MANOVA) & Logistic Regression  Reading/looking at books/magazines  1.345 (1.029 – 1.758) | [Janus and Duku 2007](https://www.tandfonline.com/doi/abs/10.1080/10409280701610796a) | Early Development Indicator – physical health and well-being, social competence, emotional maturity, language and cognitive development, communication skills and general knowledge | Canada - Community Component of the National Longitudinal Survey of Children and Youth |
|  | **Parent reading**  Reading: 3.97 (0.84)*  Maths: 3.41 (0.77)*  **Home computer**  Reading: 3.83 (0.94)*  Maths: 4.26 (0.74)* | [Larson *et al* 2015](https://pediatrics.aappublications.org/content/135/2/e440) | Direct cognitive assessment.  Early reading and maths scores | Early Childhood Longitudinal Birth Cohort Study |
| **Child does not combine words** | Logistic Regression  Unadjusted 2.0 (1.5–2.7)  Adjusted 1.7 (1.2–2.3)  Risk Index Weight: 9.8  Risk Index Weight (= β/5.3 × 100): represent a range from 0 (lowest risk) to 100 (highest risk) | [Nelson *et al* 2016](https://pediatrics.aappublications.org/content/138/2/e20154477) – poor school readiness | Poor school readiness >=1 SD below mean in early literacy and mathematics tests | USA - Early Childhood Longitudinal Study |
| **Child health** | Multivariate analysis of variance (MANOVA)  Health Utilities Index (individual’s overall functional health based on eight attributes: vision, hearing, speech, mobility (ability to get around), dexterity (use of hands and fingers), cognition (memory and thinking), emotion (feelings), and pain and discomfort)  2.346 (1.831 – 3.007) | [Janus and Duku 2007](https://www.tandfonline.com/doi/abs/10.1080/10409280701610796a) | Early Development Indicator – physical health and well-being, social competence, emotional maturity, language and cognitive development, communication skills and general knowledge | Canada - Community Component of the National Longitudinal Survey of Children and Youth |
|  | Good/fair/poor child health  Reading: -3.49 (1.25)*  Maths: -4.33 (1.15)* | [Larson *et al* 2015](https://pediatrics.aappublications.org/content/135/2/e440) | Direct cognitive assessment.  Early reading and maths scores | Early Childhood Longitudinal Birth Cohort Study |
| **Gestational age** | Logistic Regression  39 – 41 weeks reference group  **Poor school readiness reading:**  Very preterm (<32 weeks): .58 [1.29-5.15]  Moderate /Late Preterm (32-36 weeks): 1.44 [0.96-2.16]  Early Term (37-38 weeks): 1.25 [0.87-1.78]  **Poor school readiness maths:**  Very preterm (<32 weeks): 3.38 [1.66-6.91]  Moderate /Late Preterm (32-36 weeks): 1.44 [0.91-2.26]  Early Term (37-38 weeks): 1.55 [1.07-2.24] | [Shah *et al* 2016](https://pubmed.ncbi.nlm.nih.gov/27470694/) (poor school readiness reading, maths) | Poor school readiness (reading, maths) | USA - Early Childhood Longitudinal Study |
|  | **Child gestational age**  Logistic Regression  40 weeks – reference group  **<37 weeks:**  Males: 0.77 (0.59–1.00)  Females: 1.43 (0.90–2.26)  **37 weeks:**  Males: 1.06 (0.85–1.33)  Females: 1.45 (0.98–2.15)  **38 weeks**: Males: 0.97 (0.83–1.14)  Females: 1.48 (1.13–1.95)  **39 weeks:**  Males: 1.00 (0.86–1.17)  Females: 1.37 (1.06–1.78)  **41 or more weeks**  Males: 1.03 (0.86–1.24)  Females: 0.99 (0.69–1.40) | [Chittleborough *et al* 2016](https://www.sciencedirect.com/science/article/pii/S0885200615300119) | Australian Early Development Index (AEDI) [(more info)](https://pubmed.ncbi.nlm.nih.gov/24771275/)  AEDI domains – physical health and wellbeing, social competence, emotional maturity, language and cognitive skills, communication skills and general knowledge  Outcome – children vulnerable in >=2 developmental domains | Australia – Australian Early Development Consensus |
| **Low birth weight** | Logistic Regression  .67 (positive comprehensive development profile) | [Hair *et al*. 2006](https://www.sciencedirect.com/science/article/pii/S0885200606000640) | Positive comprehensive development profile – score above mean in health, social/emotional, language, cognitive domains | USA - Early Childhood Longitudinal Study |
|  | Logistic Regression  1.47 (low vocabulary 4 years) | [Scheffner Hammer *et al* 2016](https://www.ncbi.nlm.nih.gov/pmc/articles/PMC5962923/) | Low vocabulary (4 years)  Low reading performance (5 years)  Low maths performance (5 years | USA - Early Childhood Longitudinal Study |
|  | Logistic Regression  Unadjusted 1.7 (1.34 to 2.16)  Adjusted 1.26 (0.92 to 1.72)  Weighting (rank): 1.4 (12) | [Camacho *et al* 2019](https://bmjopen.bmj.com/content/9/6/e024851) | School readiness – Bracken School Readiness Assessment – not school ready | UK – Millennium Cohort Study (MCS) |
|  | Logistic Regression  Not low reference group  1.08**  **p<0.01 | [Kiernan and Mensah (2009)](https://www.cambridge.org/core/journals/journal-of-social-policy/article/poverty-maternal-depression-family-status-and-childrens-cognitive-and-behavioural-development-in-early-childhood-a-longitudinal-study/246EDA5324B918B737369A7B01E358BA) | Bracken Basic Concept Scale (BBCS) – stage of cognitive development - Learning delay | UK – Millennium Cohort Study (MCS) |
|  | Linear Regression  Reading: -3.44 (1.14)*  Maths: -6.43 (1.03)* | [Larson *et al* 2015](https://pediatrics.aappublications.org/content/135/2/e440) | Direct cognitive assessment.  Early reading and maths scores | Early Childhood Longitudinal Birth Cohort Study |
| **Late talker (24 months)** | Logistic Regression  1:72 (Low reading performance 5 years)  2.92 (Low vocabulary 4 years) | [Scheffner Hammer *et al* 2016](https://www.ncbi.nlm.nih.gov/pmc/articles/PMC5962923/) | Low vocabulary (4 years)  Low reading performance (5 years)  Low maths performance (5 years | USA - Early Childhood Longitudinal Study |
| **Low receptive vocabulary (48 months)** | Logistic Regression  3.65 (Low reading performance 60 months)  3.42 (Low maths performance) | [Scheffner Hammer *et al* 2016](https://www.ncbi.nlm.nih.gov/pmc/articles/PMC5962923/) | Low vocabulary (4 years)  Low reading performance (5 years)  Low maths performance (5 years | USA - Early Childhood Longitudinal Study |
| **Nonsingleton pregnancy** | Logistic Regression  0.68 (Low vocabulary 4 years)  1.59 (Low reading performance 5 years) | [Scheffner Hammer *et al* 2016](https://www.ncbi.nlm.nih.gov/pmc/articles/PMC5962923/) | Low vocabulary (4 years)  Low reading performance (5 years)  Low maths performance (5 years | USA - Early Childhood Longitudinal Study |
| **Child in Head Start programme (USA)** | Logistic Regression  0.52 (low vocabulary 4 years)  0.40 (low reading performance 5 years)  0.40 (low maths performance) | [Scheffner Hammer *et al* 2016](https://www.ncbi.nlm.nih.gov/pmc/articles/PMC5962923/) | Low vocabulary (4 years)  Low reading performance (5 years)  Low maths performance (5 years | USA - Early Childhood Longitudinal Study |
| **Internalizing problems** | Logistic Regression  1.55 (Low vocabulary 48 months) | [Scheffner Hammer *et al* 2016](https://www.ncbi.nlm.nih.gov/pmc/articles/PMC5962923/) | Low vocabulary (4 years)  Low reading performance (5 years)  Low maths performance (5 years | USA - Early Childhood Longitudinal Study |
| **Air quality** | Multilevel Regression  Coeff. (SE) [95% CI]  Nitrogen dioxide (NO2) – air pollutant  *p< 0.05; **p< 0.01; ***p< 0.001.  **Verbal ability (BAS):**  NO2 high at age 9 months and 3 years: −0.787*(0.385) [−1.542,−0.032]  Damp or condensation at age 9 months and 3 years: −1.501**(0.637) [−2.750,−0.252]  Damp or condensation at age 3 years: 1.487**(0.566) [−2.597,−0.377]  Second hand smoke exposure at age 9 months and 3 years: −2.179***(0.424) [−3.011,−1.348]  Second hand smoke exposure at age 9 months: −1.224**(0.521)[−2.245,−0.204  *Second hand smoke exposure at age 3 years:* −0.972**(0.376)[−1.710,−0.235]  **School readiness (BSRA):**  *Damp or condensation at age 9 months and 3 years:* 3.172***(0.904)[−4.944,−1.400]  *Damp or condensation at age 9 months:* −1.014*(0.496)[−1.986,−0.042]  *Damp or condensation at age 3 years:* 2.459***(0.804)[−4.035,−0.883]  *Second hand smoke exposure at age 9 months and 3 years:* 4.957***(0.603)[−6.138,−3.776]  *Second hand smoke exposure at age 9 months:* −3.109***(0.739)[−4.558,−1.660]  *Second hand smoke exposure at age 3 years:* -2.991***(0.534)[−4.039,−1.943 | [Midouhas *et al* 2018](https://www.sciencedirect.com/science/article/pii/S0013935117317024) | Cognitive ability - British Ability Scales Naming Vocabulary subscale and the Bracken School Readiness Assessment | UK – Millennium Cohort Study (MCS) |

# Summary of factors associated with school readiness

## Child Care

[Lipscomb *et al* 2019](https://link.springer.com/article/10.1007/s11121-019-01002-8) examined community-level social determinants of children’s school readiness. This study demonstrated an association between area-level child care supply (the number of licensed child care slots per 100 children) and higher early literacy scores (easyCBM). The authors examined child care as an indicator of social and family resources. A child’s literacy score improved by half a point (0.48) for each additional county child care slot available per 100 children. The authors stated that this finding appeared consistently in earlier alternative analyses models but that results should be interpreted with caution due to a small sample size.

[Scheffner Hammer *et al* 2016](https://www.ncbi.nlm.nih.gov/pmc/articles/PMC5962923/): Evidence from the Early Childhood Longitudinal Study (USA) showed that children who spent >10 hours per week in centre based child care or Head Start programmes were less likely to display low vocabulary at 48 months or low reading or maths performance at 60 months.

[Forry](https://www.sciencedirect.com/science/article/pii/S0885200613000367) *[et al](https://www.sciencedirect.com/science/article/pii/S0885200613000367)* [2013](https://www.sciencedirect.com/science/article/pii/S0885200613000367): Findings from Forry *et al* examining children from low income families and attendance to pre-school programmes demonstrated a positive association between subsidized centre-based child care, public pre-kindergarten and children’s academic school readiness. Those attending subsidized centre-based care had a higher likelihood of being categorized as fully ready (language/literacy and mathematical skills) than children in subsidized home-based care arrangements.

Investing in quality child care appears to be one potential avenue in providing children with opportunities to develop a variety of skills. In particular, child care appears to minimise the effects of stress that are experienced by low income parents ([Hall *et al* 2009](https://www.tandfonline.com/doi/abs/10.1080/03054980902934613)). The quality of child care also suggests that the attendance to high quality universal pre-school programmes (UK) has the potential to mitigate the impact of cognitive developmental risks and can partially address the effects of social inequalities ([Hall *et al* 2012](https://www.tandfonline.com/doi/abs/10.1080/09243453.2012.749793)). However, attendance in child care has been found to increase children’s risk for behavioural problems ([National Institute of Child Health and Human Development Early Child Care Research Network, 2003](https://pubmed.ncbi.nlm.nih.gov/12938694/)). In general, these studies suggest the benefit in facilitating low income families access and use of pre-school programmes that contribute towards children’s development and their likelihood of being ‘school ready’.

## Unemployment

[Lipscomb *et al* 2019](https://link.springer.com/article/10.1007/s11121-019-01002-8): Other area-level factors identified by Lipscomb *et al* included community unemployment rates. Within this study, county wide unemployment rates were associated with lower literacy and maths scores. For example, a child’s literacy score decreased by 1.49 points for each percentage increase in county unemployment. These community level social determinants of children’s school readiness are likely to also operate through the mechanism of social disadvantage.

[Camacho *et al* 2019](https://bmjopen.bmj.com/content/9/6/e024851): Evidence from the UK through the Millennium Cohort Study (MCS) has also highlighted unemployment as a risk factor for lower school readiness as measured by the Bracken School Readiness Assessment (BSRA). Camacho *et al* used data from the MCS to develop a predictive risk model for school readiness at age 3. Results from this study demonstrate varying rates of children classified as not being school ready (dichotomous variable for delayed/very delayed BSRA scores) of children who had both parents in work (8.4% not school ready), one parent in work (16.6% not school ready) and neither parent in work (29.9% not school ready). The predictive risk model developed in this study is able to predict 31% of children deemed ‘at risk’ of delayed school readiness with an accuracy of 74%. However, the authors highlight the ethical issues of stigmatisation by labelling young children as being at risk of poor development. They suggest the use of such predictive risk models should include support and counselling for families and access to interventions.

## Housing

[Camacho *et al* 2019](https://bmjopen.bmj.com/content/9/6/e024851): The predictive risk model developed by Camacho *et al* using data from the MCS also identified housing type as a factor associated with children’s school model. Within this study, children deemed ‘not school ready’ differed by housing type including owner occupied housing (9.3% not school ready), private rented housing (19.5% not school ready) and social housing (25.2% not school ready). Compared to owner occupied housing, children in social housing were 1.45 (1.16 – 1.67) times more likely to not be school ready compared to those living in owner occupied housing.

[Nelson *et al* 2016](https://pediatrics.aappublications.org/content/138/2/e20154477): Research from the Early Childhood Longitudinal Study in the USA using a nationally representative sample developed and validated prediction models for poor developmental and behavioural outcomes of children. The study by Nelson *et al* identified parent-reported housing quality to be associated with low academic scores. Children of parents that rated the quality of their housing as good, fair or poor compared to those that rated good/excellent were 2.2 (1.8 – 2.7) times more likely to display poor academic school readiness (>=1 SD below the mean of early reading and maths scores). The authors from this study suggest the inclusion of parent reported housing and area safety questions within routine health check visits and developmental screening, allowing the identification of children that could benefit from additional support.

## Drinking alcohol during pregnancy

[Kelly *et al* 2009](https://pubmed.ncbi.nlm.nih.gov/18974425/): UK study, using the Millennium Cohort Study, finds a J-shaped relationship between mothers reported drinking and high Strength Difficulties Questionnaire (SDQ) total difficulties score – using British Ability Scale (BAS) and the Bracken School Readiness Assessment (BSRA). Children born to mothers classified as light drinkers were less likely to have high scores, and children born to mothers classified as heavy/binge drinkers were more likely to have high scores compared with children born to abstainers. Similar patterns were seen for conduct problems, hyperactivity and emotional symptoms. Children born to light drinkers were less likely to score above the cut-offs compared with children of abstinent mothers. Children born to heavy drinkers were more likely to score above the cut-offs compared with children of abstinent mothers. Boys born to mothers who had up to 1–2 drinks per week or per occasion were less likely to have conduct problems (OR 0.59, 95%CI 0.45–0.77) and hyperactivity (OR 0.71, 95% CI 0.54–0.94). These effects remained in fully adjusted models. Girls were less likely to have emotional symptoms (OR 0.72, 95% CI 0.51–1.01) and peer problems (OR 0.68, 95% CI 0.52–0.92) compared with those born to abstainers. These effects were attenuated in fully adjusted models. Boys born to light drinkers had higher cognitive ability test scores [standard deviations, (95% CI)] BAS 0.15 (0.08–0.23) BSRA 0.24 (0.16–0.32) compared with boys born to abstainers. The difference for BAS was attenuated on adjustment for socio-economic factors, whilst the difference for BSRA remained statistically significant.

## Mother’s age at birth

[Camacho *et al* 2019](https://bmjopen.bmj.com/content/9/6/e024851): Research from the MCS by Camacho *et al* highlighted the mother’s age at birth as an important variable within their predictive risk model. Descriptive statistics on school readiness reflect this; age 14-19 (23.6% not school ready), 20-29 (13.9% not school ready), 30-39 (5.6% not school ready), 40+ (24%). Analysis indicated that women aged between 14 and 19 were 1.32 (0.95 – 1.83) more likely to have a child deemed as not school ready compared to women aged 30-39.

[Hair *et al* 2006](https://www.sciencedirect.com/science/article/pii/S0885200606000640): Research from the Early Childhood Longitudinal Study examined factors associated with children displaying a ‘comprehensive positive development profile’, those scoring above the mean in four domains of health, social/emotional, language and cognitive development. Women who were teenagers at their first birth were more likely to have children at risk in some developmental domains at school entry. The authors suggest targeted interventions aimed at risk factors such as teenage mothers may be effective in helping children to develop the necessary school readiness skills. Furthermore aside from early and targeted intervention, given that the findings demonstrate that children at risk in at least two domains of development are more likely to be children born to women who were teenagers at their first birth suggest the importance of preventative teenage pregnancy programmes.

[Scheffner Hammer *et al* 2016](https://www.ncbi.nlm.nih.gov/pmc/articles/PMC5962923/): In comparison, later research conducted through the Early Childhood Longitudinal Study by Scheffner Hammer *et al* identified older mothers (>35 years at child’s birth) to be more likely to have children with low vocabulary at 48 months at the 5% significance level. Thus, both studies suggest the risk of mothers on the lower and upper ends of the age spectrum at birth.

[Kiernan and Mensah (2009)](https://www.cambridge.org/core/journals/journal-of-social-policy/article/poverty-maternal-depression-family-status-and-childrens-cognitive-and-behavioural-development-in-early-childhood-a-longitudinal-study/246EDA5324B918B737369A7B01E358BA): Evidence from the Millennium Cohort Study suggests variance exists in the likelihood of a child having a learning delay, that is a child whose Bracken Basic Concept Scale tests are categorised as very delayed or delayed (compared to average, advanced and very advanced). Compared to mothers who were aged less than 20 at their first birth, variation existed between mothers of other age groups in the likelihood of their child having a learning delay; 20-24 (0.84), 25-29 (0.65), 30-34 (0.41), 35+ (0.59). Indeed, the authors describe that children of older mothers scored more highly in cognitive tests than children from younger mothers.

[Chittleborough *et al* 2016](https://www.sciencedirect.com/science/article/pii/S0885200615300119): A study based in Australia examined associations between early life characteristics and children’s development through the Australian Early Development Census (AEDC). The aim of the AECD is to enable communities to understand areas for improvement in supporting children and families. Data collected through the AECD was used to develop a predictive risk model using routinely collected perinatal characteristics in predicting children at risk of poor health and development. This was assessed by the Australian Early Development Index (AEDI) and classified children at risk as being vulnerable in two or more AECI domains. Mother’s age <20 was included as one of six risk factors that can discriminate between children with and without poor development at age five. These six predictors are able to identify 28% of girls and 25% of boys vulnerable in two or more AEDC domains. The authors in this study suggest that this could be used as a screening tool for families with three or more of the six risk factors at birth and offered intensive family support programme The study concludes that should such model be employed within the family support setting, it would be able to prevent about 25% of cases of poor development within the first year of schooling. Thus, mother’s age at birth is an important indicator and risk factor for poorer development outcomes of the child and screening at birth should be considered. However, the balance of specificity and sensitivity of the model and agreeing a cut off of risk factors for eligibility must also be considered. For example, lowering the number of risk factors to at least one would result in approximately 50% of births being deemed as ‘at risk’, a number deemed too high for necessary public health support and intervention.

## Breastfed

Another UK study, by [Camacho *et al* 2019](https://bmjopen.bmj.com/content/9/6/e024851) examines the impact of school readiness linked to breastfed babies, comparing those that are breastfed for more than six months against four groups, namely those that have never breastfed, breastfed for one week or less, between one and six weeks, six weeks to six months, and six months plus. In addition to the grouping by breastfeeding length, other key variables included parents’ socioeconomic classification, child’s ethnicity, maternal education, income band, sex, household number of children, mother’s age, low birth weight, mother’s mental health, infant developmental milestones, parents’ employment, and housing type.

[Kiernan and Mensah (2009)](https://www.cambridge.org/core/journals/journal-of-social-policy/article/poverty-maternal-depression-family-status-and-childrens-cognitive-and-behavioural-development-in-early-childhood-a-longitudinal-study/246EDA5324B918B737369A7B01E358BA) also use the Millennium Cohort Study, but use the Bracken Basic Concept Scape (BBCS) to examine learning delay, and compare children who were breastfed for up to six months and over six months against those that have never been breastfed. Their findings suggest that, relative to those children who have not been breastfed, that up to six months decreases the likelihood for learning delay, and for those over six months almost halves the likelihood (with a statistically significant co-efficient of 0.56).

## Parental Physical Health

[Nelson *et al* 2016](https://pediatrics.aappublications.org/content/138/2/e20154477): This study using data from the Early Childhood Longitudinal Study identified self-reported parental health status as a predictor of children’s school readiness. Parents that reported their health status to be either fair or poor were 3 (2.3 – 4) times more likely to have children with lower literacy and mathematics scores, representing a measure of school readiness at school entry. The authors commented on the novelty of this variable within their study and recommended future routine parental visits to include the measurement of such self-report information to improve the prediction of developmental risk.

[Hobcraft and Kiernan 2010](https://www.york.ac.uk/media/spsw/documents/research-and-publications/HobcraftKiernan2010PredictiveFactorsChildrensDevelopmentMillenniumCohort.pdf) reported similar findings using data from the MCS. This report builds on an earlier research study conducted by Kiernan and Mensah (2009) that examined factors shortly after pregnancy and following birth with child outcomes at age 5. Within the 2010 study, the authors aimed to identify the family and individual level factors. This report combines assessments taken at age 9 months, 3 years and 5 years with the outcome of children’s Foundation Stage Profile (FSP). This is a teacher assessment of children’s developmental achievement within six areas of learning over their first year of primary school. Results from this study identified the association between maternal self-reported health (at child age 9 months and 3 years timepoints) and child outcomes at age 5. Compared to those that reported their health to be good/excellent at both timepoints, the likelihood of having a child whose FSP was in the lowest decile for those that reported their health as fair/poor were age 9 months=2.7, 3 years=3.7 and 5 years=4.4 times greater. Thus, children with mothers with persistent physical health difficulties were more likely to be doing less well in school during their first year.

[Chittleborough *et al* 2016](https://www.sciencedirect.com/science/article/pii/S0885200615300119) using the AEDC developed an initial model of 22 perinatal variables to predict children at risk of poor development outcomes. This model included a range of measures of maternal health including mother had anaemia, hypertension, diabetes and pregnancy conditions and risky procedures (e.g. caesarean). For example, if a mother had diabetes the likelihood of their child displaying vulnerability in two or more of the AEDI domains was 1.18 (0.92-1.51) for males and 1.19 (0.79-1.79) for females.

[Larson *et al* 2015](https://pediatrics.aappublications.org/content/135/2/e440): Research from the USA’s Early Childhood Longitudinal Study found an association between a mother’s pre-pregnancy BMI and children’s cognitive development. The authors suggest that the steep socioeconomic gradient in children’s outcomes is mediated through a number of mechanisms including that of parental health. The study concludes that policies targeting socioeconomic inequality are required, in addition to a range of multifaceted early childhood interventions.

## Parental mental health

[Camacho *et al* 2019](https://bmjopen.bmj.com/content/9/6/e024851): Within Camacho *et al*.’s predictive risk model of children’s school readiness, a diagnosed maternal mental health condition (depression/anxiety) was included as a risk factor. Of those with a diagnosed condition, the likelihood of having a child being categorised as having delayed/very delayed development (not school ready) as assessed using the BSRA was 1.33 (1.16-1.53) times greater than those without a diagnosed mental health condition.

[Hobcraft and Kiernan 2010](https://www.semanticscholar.org/paper/Predictive-factors-from-age-3-and-infancy-for-poor-Hobcraft-Kiernan/39aaebd35800ba34ddec4d79b1bf6f24af327c13): This study used data from the MCS also identified maternal mental health as holding predictive power of poor outcomes for children’s development. Mothers that experienced depression during both timepoints of the survey (child age 9 months and 3 years) were 3 times more likely to have a child in the lowest decile of the Foundation Stage Profile

[Kiernan and Mensah (2009)](https://www.cambridge.org/core/journals/journal-of-social-policy/article/poverty-maternal-depression-family-status-and-childrens-cognitive-and-behavioural-development-in-early-childhood-a-longitudinal-study/246EDA5324B918B737369A7B01E358BA): A previous study using MCS data also identified maternal depression as predictive of children’s learning delay. However, the authors observed a weaker association between maternal depression and cognitive development. This was statistically significant of mothers who experienced depression when the child was 3 years (OR=1.27). However, a stronger association was observed with poor behavioural outcomes. Indeed, taking into account poverty attenuates this association, with more persistent poverty and maternal depression increasing the chances of a child performing less well in cognitive tests and displaying poorer behavioural development. Without adjusting for background variables, children living in persistently poor families with mothers that reported depression have higher odds (7-8x higher) of exhibiting learning delay in cognitive tests that those from non-deprived, non-depressed mothers. It is important to note that the measures of depression were different for the two timepoints.

[Larson *et al* 2015](https://pediatrics.aappublications.org/content/135/2/e440): Similar findings by Larson *et al* from the Early Childhood Longitudinal Study were found in relation to maternal depression.

## Family structure

It has been suggested that the presence of both parents within a family system is likely to benefit children through the increased level of parental engagement, economic resources and social capital.

[Camacho *et al* 2019](https://bmjopen.bmj.com/content/9/6/e024851): The importance and influence of family structure has been highlighted consistently in the literature. For example, research by Camacho *et al* highlighted this in relation to family size. This research study displayed increasing odds of having a child being categorised as not being school ready as the number of siblings increases compared to having one child; 2-3 children: 1.38 (1.15 to 1.66), 4+ children: 2.67 (1.94 -3.68).

[Hair *et al*. 2006](https://www.sciencedirect.com/science/article/pii/S0885200606000640): Family structure has also been highlighted in the research with regards to parental structure. A study by Hair *et al* demonstrated that compared to having two biological or adoptive parents, children from one biological/one step parent (0.74) or one biological or other parent type were less likely to display a comprehensive positive development profile. This profile was recognised as scoring above the mean in health, social/emotional, language, cognitive domains. Again, the authors suggest targeting prevention and early intervention programmes to those with risk factors including living in a single parent household.

[Janus and Duku 2007](https://www.tandfonline.com/doi/abs/10.1080/10409280701610796a): Similar findings were reported by Janus and Duku in relation to intact families, referring to a couple in which all children are natural and/or adopted offspring of both members of the couple. Children from intact families displayed higher scores in language, cognitive development, communication skills and general knowledge. In addition, they had improved social and emotional competency. An interesting finding from this study was that effect sizes of differences in children’s development indicators due to family structure (intact or not intact) were larger than those due to low income. Being from an intact family was the strongest contributor to children’s lack of vulnerability in development. Thus, being a child from an intact family provides a strong protective factor against vulnerability at the start of formal schooling.

[Hobcraft and Kiernan 2010](https://www.semanticscholar.org/paper/Predictive-factors-from-age-3-and-infancy-for-poor-Hobcraft-Kiernan/39aaebd35800ba34ddec4d79b1bf6f24af327c13) examined changes in family structure and its association with children’s school readiness in their research from the MCS. The authors examined the extent to which parental separation was associated with children’s developmental outcomes. Results are in agreement with other studies regarding differences in outcomes for parental separation and intact families, with those in the parental separation group displaying a 2.41 greater risk of being in the lowest decile of the Foundation Stage Profile.

[Kiernan and Mensah (2009)](https://www.cambridge.org/core/journals/journal-of-social-policy/article/poverty-maternal-depression-family-status-and-childrens-cognitive-and-behavioural-development-in-early-childhood-a-longitudinal-study/246EDA5324B918B737369A7B01E358BA): Previous research from the MCS explored family status in relation to children’s cognitive delay, measured using the Bracken Basic Concept Scale. Findings demonstrate that compared to children from married families, those from single parent or step parent families had higher odds of being categorised as having a cognitive learning delay. However, the authors commented on the significantly higher odds at the 5% level for children from step parent families and that this association continues with the adjustment for background factors.

This study also explored family size as assessed by the number of children, finding similar results to those by Camacho *et al* in which the odds for displaying a learning delay increase with the number of children. Compared to one child families, one sibling (1.33), two siblings (1.77) and three or more siblings (2.00) were all statistically significant.

[Chittleborough *et al* 2016](https://www.sciencedirect.com/science/article/pii/S0885200615300119): This population based study by Chittleborough *et al* using data linkage identified single relationship status as one of six final predictive risk factors for children’s poor school readiness. As discussed earlier, the authors suggest the importance of screening measures to include factors such as parental relationship status to be included in routine health visits. This research study also included the number of previous pregnancies (>=20 weeks) as another of six risk factors. The study highlights that the majority of home visiting programmes consider first time mothers as being an ‘at risk’ group and target resources accordingly. However, parents with multiple children may lack the resources, support or parental attention that contribute towards improved child development.

## Family history of learning disability

[Nelson *et al* 2016](https://pediatrics.aappublications.org/content/138/2/e20154477) and [Scheffner Hammer *et al* 2016](https://www.ncbi.nlm.nih.gov/pmc/articles/PMC5962923/): Both studies using the Early Childhood Longitudinal Study in the USA included family history of learning disability in relation to understanding the risk and protective factors that predict low academic scores. For example, family members with delayed language is likely to impact a child’s language development. Indeed Nelson *et al*’s findings indicate that those with a learning disability within the family present higher odds (1.6, 1.2-2.1) of displaying lower academic scores, measured through early reading and maths tests. This variable was retained in the final risk model for low academic scores contributing towards poor school readiness. The authors state that the final risk prediction model shows reasonable sensitivity and specificity that could help to identify children that would benefit from developmental support.

## Parent smoking

[Lipscomb *et al* 2019](https://link.springer.com/article/10.1007/s11121-019-01002-8): A US study, in Oregon, finds that the percent of births for which mothers smoked during pregnancy ranged from 0 to 27.89 between 2007 and 2009 *(Oregon Public Health Assessment Tool 2015*). The average number of births for which mothers smoked during pregnancy between 2007 and 2009 nationally was 9.7% (*U.S. Department of Health and Human Services 2018*). The impact on educational attainment, specifically early literacy and mathematics examinations, is found to be negative with a -0.24 coefficient for early mathematics and -0.191 for early literacy.

[Janus and Duku 2007](https://www.tandfonline.com/doi/abs/10.1080/10409280701610796a): A Canadian study, highlights the impact of maternal smoking on educational attainment as well as other impacts on development. They also note the work *of Richter & Richter (2001)* and *Wakschlag et al. (2006)* on how the exposure to environmental tobacco smoking not only has an impact on children’s emotional and cognitive development but also their general health status, especially exacerbating risks for respiratory infections. The paper focuses on Early Development Indicator – physical health and well-being, social competence, emotional maturity, language and cognitive development, communication skills and general knowledge.

[Chittleborough *et al* 2011](https://pubmed.ncbi.nlm.nih.gov/21536608/) focuses on smoking within the first trimester of pregnancy in the UK. The find that School Entry Assessment (language, reading, writing, maths) – total SEA score. Poor child development – scoring in lowest 10% of SEA in the Avon Longitudinal Study of Parents and Children (ALSPAC). Using Australian data, **Chittleborough *et al*. (2016)**, find the impact of smoking in the second half of pregnancy on physical health and well-being, social competence, emotional maturity, language and cognitive skills, communication skills and general knowledge. The study finds the impact of smoking in the second half of pregnancy impacts males and females differential and is therefore a factor to be aware of in any analysis.

## Parenting quality/style

[Scheffner Hammer *et al* 2016](https://www.ncbi.nlm.nih.gov/pmc/articles/PMC5962923/) and [Larson *et al* 2015](https://pediatrics.aappublications.org/content/135/2/e440): Within the Early Childhood Longitudinal Study, a number of factors relating to parenting behaviours were found to be associated with children’s vocabulary, reading and maths performance at age 5. These included parenting quality, positive interactions with their child, parent supportiveness and rules. In addition, proxy measures of parental engagement such as the child attending at least two activities or classes at age 4 were also associated with early reading and maths. Within Larson *et al*’s study, parenting style and beliefs accounted for 14% of the gap in children’s reading and maths scores. It is possible that parents providing nurturing relationships and stimulating environments benefit children’s cognitive development. Furthermore, the socioeconomic gradient in children’s development is likely to be mediated through factors including parenting behaviours. The authors advocated for a broader range of early interventions that target parenting factors and behaviour.

## Lower parental expectations of child’s education & Home learning environment

[Nelson *et al*’s 2016](https://pediatrics.aappublications.org/content/138/2/e20154477) paper focusing on the US and examines predictors of poor school readiness in children without development delay at the age of 2. The paper highlights the self-reported expectations of parents on a child’s education being less than a 4 year degree have an unadjusted odds ratio of 2.2 and adjusted odds ratio of 1.3 (at 95% CI), suggesting that the likelihood is higher for low academic scores at kindergarten entry (aged 2). Their paper also includes a variable on home learning environment as a predictor of poor school readiness, unsurprisingly it also highlights that if the home learning environment (using frequency of shared reading at home) has an odds ratio as high as 2.7 (unadjusted) when no shared reading takes place at home, compared with reading every day. The overarching findings in the paper show that parental factors are major contributors to a child’s school readiness – such as parental education, parental physical and mental health, levels of food insecurity, self-reported neighbourhood safety, and the child is brought up in a single parent household. Another key predictor used in Nelson *et al*. (2016) is that the child does not combine words, with the paper showing an odds ratio of 2.0 and 1.7 for the adjusted and unadjusted ORs respectively – suggesting a relatively high likelihood of a child’s school readiness being poor when compared with a child able to combine words. [Janus and Duku 2007](https://www.tandfonline.com/doi/abs/10.1080/10409280701610796a) investigates home learning environment and the impact this has on a child’s school readiness, and much like [Nelson *et al*’s 2016](https://pediatrics.aappublications.org/content/138/2/e20154477) that there is significant importance on home learning in order to better prepare a child for school.

## Child health

[Janus and Duku 2007](https://www.tandfonline.com/doi/abs/10.1080/10409280701610796a) also makes use of the Health Utilities Index (individual’s overall functional health based on eight attributes: vision, hearing, speech, mobility (ability to get around), dexterity (use of hands and fingers), cognition (memory and thinking), emotion (feelings), and pain and discomfort) as a predictor of school readiness. The odds ratio for the Health Utilities Index is reported as 2.373

## Gestational age

In addition to birth weight, the link between gestational age and poor school readiness for reading and math with suggestion of a threshold effect in children born ≥ 32 weeks gestation is explored in [Shah *et al*’s 2016](https://pubmed.ncbi.nlm.nih.gov/27470694/) US study using the Early Childhood Longitudinal Study. In adjusted models, the odds of poor school readiness in very pre-term children (less than or equal to 32 weeks) with regards to reading and mathematics were 2.58 and 3.38, respectively. However, for infants born moderate to late pre-term and early term, the odds of poor school readiness in reading did not differ from children born full-term (39-41 weeks). Using Australian linked data, [Chittleborough *et al* 2016](https://www.sciencedirect.com/science/article/pii/S0885200615300119) find that, when comparing with a 40-week full term birth, that there are differences in school readiness not only by gestational age, but also by gender. For children born between 37 and 40 weeks the researchers use multivariable associations are between the perinatal risk factors and vulnerability on two or more measure of children’s health and development domains. The findings present the risks that are included in the development of prediction models. The impact on males is negligible with all but a gestational age of less than 37 weeks has a coefficient of 0.77 which is a significant lower outcome for physical health and wellbeing, social competence, emotional maturity, language and cognitive skills, communication skills and general knowledge.

## Month of Birth

Although there a no relevant papers to discuss a direct link between month of birth of a child and school readiness, there is a link between several of the key factors discussed above, and therefore is imperative to be aware of when looking at factors that can help predict school readiness. An example of the link would be the identification of special educational needs is more probable over time – so a child born in September of a school year will have had up to 12 months more to have a diagnosis and have a greater likelihood of support in place. Equally, the effects of being a late talker (24 months) are also going to be more likely to more prevalent for a child born in August – and is therefore young in the school year – than a September child in the same academic year who has between 11-12 months relative time to catch up.

## Low birth weight

There have been many studies into the impact of low birth weight on educational attainment, however as a predictor of school readiness. [Hair *et al*. 2006](https://www.sciencedirect.com/science/article/pii/S0885200606000640) examines this using US data, finding that the odds ratio predicting membership in the school readiness proﬁles from background characteristics (weighted) is 0.67 for comprehensive positive development profile, suggesting…. The study also finds statistically significant odds ratios of 1.25 and 1.34 for social/emotional risk profile and health risk profile respectively.

[Scheffner Hammer *et al* 2016](https://www.ncbi.nlm.nih.gov/pmc/articles/PMC5962923/), [Camacho *et al* 2019](https://bmjopen.bmj.com/content/9/6/e024851) and [Kiernan and Mensah (2009)](https://www.cambridge.org/core/journals/journal-of-social-policy/article/poverty-maternal-depression-family-status-and-childrens-cognitive-and-behavioural-development-in-early-childhood-a-longitudinal-study/246EDA5324B918B737369A7B01E358BA)find similar negative impacts on child that experience a low birth weight on their educational attainment. [Scheffner Hammer *et al* 2016](https://www.ncbi.nlm.nih.gov/pmc/articles/PMC5962923/), for example finds a statistical significance result with regards to low birth weight using logistic regression as having an odds ratio of 1.47 for low vocabulary at 48 months. When looking at UK studies using the Millennium Cohort Study, Kiernan and Mensah (2009) and Camacho *et al*. (2019) find odds ratios in excess of 1 regarding low birth weight. Specifically, Camacho *et al*. (2019) find an odds ratio for low birth weight as 1.7 (unadjusted) and 1.26 (adjusted) as a predictor of school readiness.

## Late talker (24 months) & Non-singleton pregnancy & Child in Head Start programme (USA) & Internalizing problems

[Scheffner Hammer *et al* 2016](https://www.ncbi.nlm.nih.gov/pmc/articles/PMC5962923/), highlight several other key variables worth noting for school readiness including a late talker (24 months), non-singleton pregnancy, gestational age, and ethnicity. Using the odds ratio from a logistic regression, they find that for late talker’s (24 months) holds an odd ratio of 0.69 (at a p-value of 0.001). The models utilized in this study use gender-specific cutoffs for a late talker – and boys were found to be more likely to be a late talker, when taking into consideration sociodemographic, birth, and family variables. They find that there are three potentially malleable related to being a late talker, such as parenting quality, childcare, and approaches to learning. With regards to non-singleton pregnancy (twin or greater order) increase the odds of being a late talker in the second model used – however, these are not statistically significant at the 5% level when health issues are included in the third model with gender splits, yet is statistically significant when the sample is not split by gender (1.48 with a p-value of 5%). The use of the US’s Head Start Programme has a statistically significant at the 0.1% level.

## Air quality & environmental factors

[Midouhas *et al* 2018](https://www.sciencedirect.com/science/article/pii/S0013935117317024) use UK Millennium Cohort Study data to examine the effects of NO_2_ (deciles) and indoor air quality at age 9 months on verbal ability and school readiness at age 3 years. The study finds a statistically significant negative impact (-0.787 at the 5% confidence level) when a child experiences high levels of nitrogen dioxide at the age of 9 months on their verbal ability at the age of 3. In addition, the study also finds that an exposure to damp or condensation at 9 months has a more statistically significant negative impact (-1.501 at the 1% confidence level) on verbal reasoning. The most statistically significant variable related to verbal reasoning and environment factors is the exposure to second hand smoke at 9 months, having a -2.179 coefficient at the 0.1% significance level.

## Absenteeism

[Ansari & Purtell (2018](https://pubmed.ncbi.nlm.nih.gov/28508590/)) analyse US data relating to the Head Start policy – comparable to the Flying Start policy in the UK to support low-income parents with early parenthood. The findings of the multivariate analysis found there is no disadvantage to language development, however children who were more frequently absent demonstrated smaller gains in literacy (-0.05 at 1% p-value) and mathematics (-0.06 at a p-value of 1%). Their study followed up using propensity score matching which supported the findings of the multivariate analysis.

## English as a second language (ESL)

Another study relating to the Head Start programme in the US is a study by [Park *et al* (2015),](https://journals.sagepub.com/doi/abs/10.1177/1476718x13507445) native English speakers did not perform any better than second language students who attended HS on the other three literacy skills, recognising beginning sounds of words, writing their first name, and reading story books on their own. These findings might be a result of effective Head Start programs, or they might result from relationships between children’s native language and their second language.

## Subsidized Childcare

[Johnson *et al.* (2013)](https://www.ncbi.nlm.nih.gov/pmc/articles/PMC3745537/) observed a subsample of the Early Childhood Longitudinal Study – Birth Cohort cases by data collectors using the Early Childhood Environment Rating Scale. Findings suggest that subsidy receipt in preschool is not directly linked to subsequent reading or social-emotional skills. However, subsidy receipt predicted lower math scores among children attending community-based centres. Supplementary analyses revealed that subsidies predicted greater use of community-based centre care, but this association did not appear to affect school readiness.
