# Supplementary material for: Factors associated with low school readiness, a linked health and education data study in Wales, UK
Source: PLoS One. 2023 Dec 11;18(12):e0273596. doi: 10.1371/journal.pone.0273596 (PMC10712842; doi:10.1371/journal.pone.0273596)
Supplement: S1 File — (ZIP) [file pone.0273596.s001.zip › Appendix2_Supplementary_SRNFactors_MappedRD.docx]

# Appendix 2: Information on mapping risk factors of low school readiness

# Risk factors for low school readiness and mapping them to the routine data (RD)

| Risk factors from literature | Analogous variable from RD | Source | Comments |  |
| --- | --- | --- | --- | --- |
| Childcare supply |  |  | Not available in RD |  |
| Unemployment | Area level deprivation - Employment/Income deprivation score  Family level deprivation- Free School Meals (FSM) | Welsh Index of Multiple Deprivation (WIMD) 2014/19 reference table  FSM from education data |  | d |
| Housing | Housing deprivation | WIMD 2014/19 reference table |  | d |
| Drinking alcohol during pregnancy | Mother's alcohol-related condition during pregnancy from RD | TECC/NCCHD,  WLGP, PEDW |  | d |
| Mother’s age | Maternal age at birth | TECC/NCCHD |  | d |
| Breastfed | Breastfeeding record from RD | TECC/NCCHD |  | d |
| Emergency caesarean | Mode of delivery | TECC/NCCHD |  | d |
| Parental physical health | Mother and Father had anaemia, hypertension, diabetes, Antepartum haemorrhage, cancer,  ***CHR score***  Mother’s pre-pregnancy BMI, | TECC/NCCHD, PEDW,  WLGP |  | d |
| Parental mental health | Mother and Father had  Depression, Serious Mental Illness, Anti-depressant medication | TECC/NCCHD, PEDW,  WLGP | Anytime between their birth child’s school readiness was measured/ just during pregnancy for mother? | d |
| Family structure | Living with single adult/  Number of adults in the household  Number of children in the household  Number of previous pregnancies | WDSD, TECC/NCCHD |  | d |
| Family history of learning disability | Mother had learning difficulty | TECC/NCCHD, WLGP |  | d |
| Parent smoking | Maternal smoking record during pregnancy | TECC/NCCHD, WLGP |  | d |
| Parenting quality/style  Parental expectations  Home learning environment  Child does not combine words |  |  | Not available |  |
| Child physical health | Epilepsy, Diabetes, Asthma,  Number of ear and eye infection/ medication - Ask MSB  Number of emergency hospital admission  Number of A&E attendance | PEDW, WLGP, A&E |  | d |
| Gestational age | Gestational age | TECC/NCCHD |  | d |
| Low birth weight | Low birth weight | TECC/NCCHD |  |  |
| Late talker (24 months)  Low receptive vocabulary (48 months) |  |  | Not available |  |
| Nonsingleton pregnancy | Flag singleton and nonsingleton children | TECC/NCCHD |  | d |
| Child in Head Start programme (USA) |  |  | Not available |  |
| Internalizing problems |  |  | Not available |  |
| Air quality | Physical environment deprivation | WIMD 2014/19 reference table | No equivalent individual level record available in RD | d |
| Absenteeism | Total absence (authorised and unauthorised) | Education database |  | d |
| English as a second language |  | Education database | Not available |  |
| Subsidized Childcare |  |  | Not available |  |
| Main outcome variable - School readiness | Foundation phase/KS1 (score) | Education database |  | d |
| Ethnicity | Ethnicity - Check with Cynthia |  | Not available |  |
| Family member - Substance misuse  And/or ALCOHOL record | Any household member with substance misuse record  Any household member alcohol related hospital admission | Substance Misuse Database, PEDW, WLGP |  | Mother d |
| Evidence of partner violence | Mother with assault record in PEDW/WLGP | PEDW, WLGP |  | d |
| Maternal education |  | - | Not available |  |
| *Congenital abnormality* |  | - | Not available |  |
| Deprivation of the area | Local area deprivation overall WIMD score | Welsh Index of Multiple Deprivation (WIMD) 2014/2019 lookup table |  | d |
| Living area | Rural/urban area | 2014/2019 lookup table |  | d |

*Cohort selection (inclusion and exclusion):*

- Anyone who are born/residing in Wales and completed foundation phase in Wales (linked WDSD and Education dataset)
- In the latest version of the education data, foundation phase record (3-7) from 2012 till 2018
- Study population -

1. Completed foundation phase (age 3 - 7) between 2012 and 2018 - Born between 2005 and 2015

- They have GP record in SAIL and valid RALF record in WDSD

*Follow up period:* Till they completed foundation phase/KS1 exam

*Current datasets:* WDSD, NCCHD, PEDW, WLGP, A&E, EDUCATION, TECC, Substance misuse database (SMD), *MIDS, Lifelong learning, POLICE data*
