# Supplementary material for: Factors associated with low school readiness, a linked health and education data study in Wales, UK
Source: PLoS One. 2023 Dec 11;18(12):e0273596. doi: 10.1371/journal.pone.0273596 (PMC10712842; doi:10.1371/journal.pone.0273596)
Supplement: S1 File — (ZIP) [file pone.0273596.s001.zip › Appendix3_readcodes_ICD10codes.docx]

# Appendix 3: Read codes and ICD 10 codes of the health conditions.

**Table 1:Diabetes ICD10 codes**

| ICD10 | Description |
| --- | --- |
| E100 | Insulin-dependent diabetes mellitus with coma |
| E101 | Insulin-dependent diabetes mellitus with ketoacidosis |
| E102 | Insulin-dependent diabetes mellitus with renal complications |
| E103 | Insulin-dependent diabetes mellitus with ophthalmic comps |
| E104 | Insulin-dependent diabetes mellitus with neurological comps |
| E105 | Insulin-dependent diabetes mellitus with periph circ comps |
| E106 | Insulin-dependent diabetes mellitus with other spec comps |
| E107 | Insulin-dependent diabetes mellitus with multiple comps |
| E108 | Insulin-dependent diabetes mellitus with unspec comps |
| E109 | Insulin-dependent diabetes mellitus without complications |
| E110 | Non-insulin-dependent diabetes mellitus with coma |
| E111 | Non-insulin-dependent diabetes mellitus with ketoacidosis |
| E112 | Non-insulin-dependent diabetes mellitus with renal comps |
| E113 | Non-insulin-dependent diabetes mellitus with ophthalm comps |
| E114 | Non-insulin-dependent diabetes mellitus with neuro comps |
| E115 | Non-insulin-depend diabetes mellitus with periph circ comp |
| E116 | Non-insulin-depend diabetes mellitus with other spec comp |
| E117 | Non-insulin-dependent diabetes mellitus with multiple comps |
| E118 | Non-insulin-dependent diabetes mellitus with unspec comps |
| E119 | Non-insulin-depend diabetes mellitus without complication |
| E120 | Malnutrition-related diabetes mellitus with coma |
| E121 | Malnutrition-related diabetes mellitus with ketoacidosis |
| E122 | Malnutrition-related diabetes mellitus with renal comps |
| E123 | Malnutrition-related diabetes mellitus with ophthalmic comps |
| E124 | Malnutrition-related diabetes mellitus with neuro comps |
| E125 | Malnutrition-relat diabetes mellitus with periph circ comp |
| E126 | Malnutrition-relat diabetes mellitus with other spec comps |
| E127 | Malnutrition-related diabetes mellitus with multiple comps |
| E128 | Malnutrition-related diabetes mellitus with unspec comps |
| E129 | Malnutrition-related diabetes mellitus without complications |
| E130 | Other specified diabetes mellitus with coma |
| E131 | Other specified diabetes mellitus with ketoacidosis |
| E132 | Other specified diabetes mellitus with renal complications |
| E133 | Other specified diabetes mellitus with ophthalmic comps |
| E134 | Other specified diabetes mellitus with neurological comps |
| E135 | Other specified diabetes mellitus with periph circ comps |
| E136 | Other specified diabetes mellitus with other spec comps |
| E137 | Other specified diabetes mellitus with multiple comps |
| E138 | Other specified diabetes mellitus with unspecified comps |
| E139 | Other specified diabetes mellitus without complications |
| E140 | Unspecified diabetes mellitus with coma |
| E141 | Unspecified diabetes mellitus with ketoacidosis |
| E142 | Unspecified diabetes mellitus with renal complications |
| E143 | Unspecified diabetes mellitus with ophthalmic complications |
| E144 | Unspecified diabetes mellitus with neurological comps |
| E145 | Unspecified diabetes mellitus with periph circulatory comps |
| E146 | Unspecified diabetes mellitus with other specified comps |
| E147 | Unspecified diabetes mellitus with multiple complications |
| E148 | Unspecified diabetes mellitus with unspecified complications |
| E149 | Unspecified diabetes mellitus without complications |
| O240 | Pre-existing diabetes mellitusinsulin-dependent |
| O241 | Pre-existing diabetes mellitusnon-insulin-dependent |
| O242 | Pre-existing malnutrition-related diabetes mellitus |

**Table 2: Diabetes READ codes**

| READ | Description |
| --- | --- |
| C10.. | Diabetes mellitus |
| C109J | Insulin treated Type 2 diabetes mellitus |
| C109K | Hyperosmolar non-ketotic state in type 2 diabetes mellitus |
| C10C. | Diabetes mellitus autosomal dominant |
| C10D. | Diabetes mellitus autosomal dominant type 2 |
| C10E. | Type 1 diabetes mellitus |
| C10E0 | Type 1 diabetes mellitus with renal complications |
| C10E1 | Type 1 diabetes mellitus with ophthalmic complications |
| C10E2 | Type 1 diabetes mellitus with neurological complications |
| C10E3 | Type 1 diabetes mellitus with multiple complications |
| C10E4 | Unstable type 1 diabetes mellitus |
| C10E5 | Type 1 diabetes mellitus with ulcer |
| C10E6 | Type 1 diabetes mellitus with gangrene |
| C10E7 | Type 1 diabetes mellitus with retinopathy |
| C10E8 | Type 1 diabetes mellitus - poor control |
| C10E9 | Type 1 diabetes mellitus maturity onset |
| C10EA | Type 1 diabetes mellitus without complication |
| C10EB | Type 1 diabetes mellitus with mononeuropathy |
| C10EC | Type 1 diabetes mellitus with polyneuropathy |
| C10ED | Type 1 diabetes mellitus with nephropathy |
| C10EE | Type 1 diabetes mellitus with hypoglycaemic coma |
| C10EF | Type 1 diabetes mellitus with diabetic cataract |
| C10EG | Type 1 diabetes mellitus with peripheral angiopathy |
| C10EH | Type 1 diabetes mellitus with arthropathy |
| C10EJ | Type 1 diabetes mellitus with neuropathic arthropathy |
| C10EK | Type 1 diabetes mellitus with persistent proteinuria |
| C10EL | Type 1 diabetes mellitus with persistent microalbuminuria |
| C10EM | Type 1 diabetes mellitus with ketoacidosis |
| C10EN | Type 1 diabetes mellitus with ketoacidotic coma |
| C10EP | Type 1 diabetes mellitus with exudative maculopathy |
| C10EQ | Type 1 diabetes mellitus with gastroparesis |
| C10ER | Latent autoimmune diabetes mellitus in adult |
| C10F. | Type 2 diabetes mellitus |
| C10F0 | Type 2 diabetes mellitus with renal complications |
| C10F1 | Type 2 diabetes mellitus with ophthalmic complications |
| C10F2 | Type 2 diabetes mellitus with neurological complications |
| C10F3 | Type 2 diabetes mellitus with multiple complications |
| C10F4 | Type 2 diabetes mellitus with ulcer |
| C10F5 | Type 2 diabetes mellitus with gangrene |
| C10F6 | Type 2 diabetes mellitus with retinopathy |
| C10F7 | Type 2 diabetes mellitus - poor control |
| C10F9 | Type 2 diabetes mellitus without complication |
| C10FA | Type 2 diabetes mellitus with mononeuropathy |
| C10FB | Type 2 diabetes mellitus with polyneuropathy |
| C10FC | Type 2 diabetes mellitus with nephropathy |
| C10FD | Type 2 diabetes mellitus with hypoglycaemic coma |
| C10FE | Type 2 diabetes mellitus with diabetic cataract |
| C10FF | Type 2 diabetes mellitus with peripheral angiopathy |
| C10FG | Type 2 diabetes mellitus with arthropathy |
| C10FH | Type 2 diabetes mellitus with neuropathic arthropathy |
| C10FJ | Insulin treated Type 2 diabetes mellitus |
| C10FK | Hyperosmolar non-ketotic state in type 2 diabetes mellitus |
| C10FL | Type 2 diabetes mellitus with persistent proteinuria |
| C10FM | Type 2 diabetes mellitus with persistent microalbuminuria |
| C10FN | Type 2 diabetes mellitus with ketoacidosis |
| C10FP | Type 2 diabetes mellitus with ketoacidotic coma |
| C10FQ | Type 2 diabetes mellitus with exudative maculopathy |
| C10FR | Type 2 diabetes mellitus with gastroparesis |
| C10FS | Maternally inherited diabetes mellitus |
| C10G. | Secondary pancreatic diabetes mellitus |
| C10G0 | Secondary pancreatic diabetes mellitus without complication |
| C10H. | Diabetes mellitus induced by non-steroid drugs |
| C10H0 | DM induced by non-steroid drugs without complication |
| C10M. | Lipoatrophic diabetes mellitus |
| C10M0 | Lipoatrophic diabetes mellitus without complication |
| C10N. | Secondary diabetes mellitus |
| C10N0 | Secondary diabetes mellitus without complication |
| C10N1 | Cystic fibrosis related diabetes mellitus |

**Table 3: Cancer ICD10 codes**

| ICD10 | Descriptions |
| --- | --- |
| C00 | Malignant neoplasm of lip |
| C000 | Malignant neoplasm of external upper lip |
| C001 | Malignant neoplasm of external lower lip |
| C002 | Malignant neoplasm of external lipunspecified |
| C003 | Malignant neoplasm of upper lipinner aspect |
| C004 | Malignant neoplasm of lower lipinner aspect |
| C005 | Malignant neoplasm of lipunspecifiedinner aspect |
| C006 | Malignant neoplasm of commissure of lip |
| C008 | Malignant neoplasm of overlapping lesion of lip |
| C009 | Malignant neoplasm of lipunspecified |
| C01 | Malignant neoplasm of base of tongue |
| C01X | Malignant neoplasm of base of tongue |
| C02 | Malignant neoplasm of other and unspecified parts of tongue |
| C020 | Malignant neoplasm of dorsal surface tongue |
| C021 | Malignant neoplasm of border of tongue |
| C022 | Malignant neoplasm of ventral surface of tongue |
| C023 | Malignant neo of anterior two-thirds of tongue part unspecified |
| C024 | Malignant neoplasm of lingual tonsil |
| C028 | Malignant neoplasm of overlapping lesion of tongue |
| C029 | Malignant neoplasm of tongue unspecified |
| C03 | Malignant neoplasm of gum |
| C030 | Malignant neoplasm of upper gum |
| C031 | Malignant neoplasm of lower gum |
| C039 | Malignant neoplasm of gum unspecified |
| C04 | Malignant neoplasm of floor of mouth |
| C040 | Malignant neoplasm of floor of anterior floor of mouth |
| C041 | Malignant neoplasm of lateral floor of mouth |
| C048 | Malignant neoplasm overlapping lesion of floor of mouth |
| C049 | Malignant neoplasm of floor of mouth floor of mouth unspecified |
| C05 | Malignant neoplasm of palate |
| C050 | Malignant neoplasm of hard palate |
| C051 | Malignant neoplasm of soft palate |
| C052 | Malignant neoplasm of uvula |
| C058 | Malignant neoplasm overlapping lesion of palate |
| C059 | Malignant neoplasm of palate unspecified |
| C06 | Malignant neoplasm of other and unspecified parts of mouth |
| C060 | Malignant neoplasm cheek mucosa |
| C061 | Malignant neoplasm of vestibule of mouth |
| C062 | Malignant neoplasm of retromolar area |
| C068 | Malignant neoplasmoverlap les of oth & unsp part of mouth |
| C069 | Malignant neoplasm of part of mouthunspecified |
| C07 | Malignant neoplasm of parotid gland |
| C07X | Malignant neoplasm of parotid gland |
| C08 | Maligt neoplasm of oth and unspec major saliv glands |
| C080 | Malignant neoplasm of submandibular gland |
| C081 | Malignant neoplasm of sublingual gland |
| C088 | Malignant neoplasmoverlapping lesion of major saliv gland |
| C089 | Malignant neoplasm of major salivary glandunspecified |
| C09 | Malignant neoplasm of tonsil |
| C090 | Malignant neoplasm tonsillar fossa |
| C091 | Malig neo of tonsillar pillar (anterior)(posterior) |
| C098 | Malignant neoplasm of overlapping lesion of tonsil |
| C099 | Malignant neoplasm of tonsil unspecified |
| C10 | Malignant neoplasm of oropharynx |
| C100 | Malignant neoplasm of vallecula |
| C101 | Malignant neoplasm of anterior surface of epiglottis |
| C102 | Malignant neoplasm of lateral wall of oropharynx |
| C103 | Malignant neoplasm of posterior wall of oropharynx |
| C104 | Malignant neoplasm of branchial cleft |
| C108 | Malignant neoplasm overlapping lesion of oropharynx |
| C109 | Malignant neoplasm of oropharynx unspecified |
| C11 | Malignant neoplasm of nasopharynx |
| C110 | Malignant neoplasm of superior wall of nasopharynx |
| C111 | Malignant neoplasm of posterior wall of nasopharynx |
| C112 | Malignant neoplasm of lateral wall of nasopharynx |
| C113 | Malignant neoplasm of anterior wall of nasopharynx |
| C118 | Malignant neoplasm overlapping lesion of nasopharynx |
| C119 | Malignant neoplasm of nasopharynx unspecified |
| C12 | Malignant neoplasm of pyriform sinus |
| C12X | Malignant neoplasm of pyriform sinus |
| C13 | Malignant neoplasm of hypopharynx |
| C130 | Malignant neoplasm of hypopharynxpostcricoid region |
| C131 | Malig neoplasm aryepiglottic foldhypopharyngeal aspect |
| C132 | Malignant neoplasm posterior wall of hypopharynx |
| C138 | Malignant neoplasm overlapping lesion of hypopharynx |
| C139 | Malignant neoplasm of hypopharynx unspecified |
| C14 | Mal neo oth ill-def sites lip/oral cavity/pharynx |
| C140 | Malignant neoplasm of pharynxunsp |
| C142 | Malignant neoplasm of Waldeyer's ring |
| C148 | Malig neooverlapping lesion of liporal cavity & pharynx |
| C15 | Malignant neoplasm of oesophagus |
| C150 | Malignant neoplasm of cervical part of oesophagus |
| C151 | Malignant neoplasm of thoracic part of oesophagus |
| C152 | Malignant neo of abdominal part of oesophagus |
| C153 | Malignant neoplasm of upper third of oesophagus |
| C154 | Malignant neoplasm of middle third of oesophagus |
| C155 | Malignant neoplasm of lower third of oesophagus |
| C158 | Malignant neoplasm overlapping lesion of oesophagus |
| C159 | Malignant neoplasm of oesophagus unspecified |
| C16 | Malignant neoplasm of stomach |
| C160 | Malignant neoplasm of cardia of stomach |
| C161 | Malignant neoplasm of fundus of stomach |
| C162 | Malignant neoplasm of body of stomach |
| C163 | Malignant neoplasm of pyloric antrum |
| C164 | Malignant neoplasm of pylorus |
| C165 | Malignant neoplasm of lesser curvature of stomachunsp |
| C166 | Malignant neoplasm of greater curvature of stomachunsp |
| C168 | Malignant neoplasm overlapping lesion of stomach |
| C169 | Malignant neoplasm of stomachunspecified |
| C17 | Malignant neoplasm of small intestine |
| C170 | Malignant neoplasm of small intestineduodenum |
| C171 | Malignant neoplasm of small intestinejejunum |
| C172 | Malignant neoplasm of small intestineileum |
| C173 | Malignant neoplasm of small intestineMeckel's diverticulum |
| C178 | Malignant neoplasm overlapping lesion of small intestine |
| C179 | Malignant neoplasm of small intestineunspecified |
| C18 | Malignant neoplasm of colon |
| C180 | Malignant neoplasm of caecum |
| C181 | Malignant neoplasm of appendix |
| C182 | Malignant neoplasm of ascending colon |
| C183 | Malignant neoplasm of hepatic flexure |
| C184 | Malignant neoplasm of transverse colon |
| C185 | Malignant neoplasm of splenic flexure |
| C186 | Malignant neoplasm of descending colon |
| C187 | Malignant neoplasm of sigmoid colon |
| C188 | Malignant neoplasm overlapping lesion of colon |
| C189 | Malignant neoplasm of colonunspecified |
| C19 | Malignant neoplasm of rectosigmoid junction |
| C19X | Malignant neoplasm of rectosigmoid junction |
| C20 | Malignant neoplasm of rectum |
| C20X | Malignant neoplasm of rectum |
| C21 | Malignant neoplasm of anus and anal canal |
| C210 | Malignant neoplasm of anusunspecified |
| C211 | Malignant neoplasm of anal canal |
| C212 | Malignant neoplasm of cloacogenic zone |
| C218 | Malig neooverlapping lesion of rectumanus and anal canal |
| C22 | Malignant neoplasm of liver and intrahepatic bile ducts |
| C220 | Malignant neoplasmliver cell carcinoma |
| C221 | Malignant neoplasmintrahep bile duct carcinoma |
| C222 | Malignant neoplasmhepatoblastoma |
| C223 | Malignant neoplasmangiosarcoma of liver |
| C224 | Malignant neoplasmother sarcomas of liver |
| C227 | Malignant neoplasmoth spec carcinomas of liver |
| C229 | Malignant neoplasmliverunspecified |
| C23 | Malignant neoplasm of gallbladder |
| C23X | Malignant neoplasm of gallbladder |
| C24 | Maligt neoplasm of other and unspec parts biliary tract |
| C240 | Malignant neoplasm of extrahepatic bile duct |
| C241 | Malignant neoplasm of Ampulla of Vater |
| C248 | Malignant neoplasm overlapping lesion of biliary tract |
| C249 | Malignant neoplasm of biliary tractunspecified |
| C25 | Malignant neoplasm of pancreas |
| C250 | Malignant neoplasm of head of pancreas |
| C251 | Malignant neoplasm of body of pancreas |
| C252 | Malignant neoplasm of tail of pancreas |
| C253 | Malignant neoplasm of pancreatic duct |
| C254 | Malignant neoplasm of endocrine pancreas |
| C257 | Malignant neoplasm of other parts of pancreas |
| C258 | Malignant neoplasmoverlapping lesion of pancreas |
| C259 | Malignant neoplasm of pancreasunspecified |
| C26 | Malignant neoplasm of other and ill-defined digestive organs |
| C260 | Malignant neoplasm of intestinal tractpart unsp |
| C261 | Malignant neoplasm of spleen |
| C268 | Malignant neoplasmoverlapping lesion of digestive system |
| C269 | Malignant neoplasm of ill-def sites within digestive system |
| C30 | Malignant neoplasm of nasal cavity and middle ear |
| C300 | Malignant neoplasm of nasal cavity |
| C301 | Malignant neoplasm of middle ear |
| C31 | Malignant neoplasm of accessory sinuses |
| C310 | Malignant neoplasm of maxillary sinus |
| C311 | Malignant neoplasm of ethmoidal sinus |
| C312 | Malignant neoplasm of frontal sinus |
| C313 | Malignant neoplasm of sphenoidal sinus |
| C318 | Malignant neoplasmoverlapping lesion accessory sinuses |
| C319 | Malignant neoplasm of accessory sinusunsp |
| C32 | Malignant neoplasm of larynx |
| C320 | Malignant neoplasm of glottis |
| C321 | Malignant neoplasm of supraglottis |
| C322 | Malignant neoplasm of subglottis |
| C323 | Malignant neoplasm of laryngeal cartilage |
| C328 | Malignant neoplasmoverlapping lesion of larynx |
| C329 | Malignant neoplasm of larynxunspecified |
| C33 | Malignant neoplasm of trachea |
| C33X | Malignant neoplasm of trachea |
| C34 | Malignant neoplasm of bronchus and lung |
| C340 | Malignant neoplasm of main bronchus |
| C341 | Malignant neoplasm of upper lobebronchus or lung |
| C342 | Malignant neoplasm of middle lobebronchus or lung |
| C343 | Malignant neoplasm of lower lobebronchus or lung |
| C348 | Malignant neoplasm of overlap les of bronchus & lung |
| C349 | Malignant neoplasm of bronchus or lungunspec |
| C37 | Malignant neoplasm of thymus |
| C37X | Malignant neoplasm of thymus |
| C38 | Malignant neoplasm of heart, mediastinum and pleura |
| C380 | Malignant neoplasm of heartmediastinum & pleuraheart |
| C381 | Malignant neoplasm of anterior mediastinum |
| C382 | Malignant neoplasm of posterior mediastinum |
| C383 | Malig neo heartmediastinum & pleura |
| C384 | Malignant neoplasm of pleura |
| C388 | Malig neooverlapping lesion of heartmediastinum & pleura |
| C39 | Malig neo oth + ill-def sites resp sys + intrathorac orgs |
| C390 | Malignant neoplasm of upper respiratory tractpart unsp |
| C398 | Malignant neoplasmoverlap lesion of resp & intrathor orgs |
| C399 | Malignant neoplasm of ill-def sites within the resp sys |
| C40 | Malignant neoplasm of bone and articular cartilage of limbs |
| C400 | Malignant neoplasm of scapula and long bones of upper limb |
| C401 | Malignant neoplasm of short bones of upper limb |
| C402 | Malignant neoplasm of long bones of lower limb |
| C403 | Malignant neoplasm of short bones of lower limb |
| C408 | Malignant neoplasmoverlap les bone and artic cart of limbs |
| C409 | Malignant neoplasm of bone and artic cart of limbunsp |
| C41 | Malig neo bone + articular cartilage of oth + unspeci sites |
| C410 | Malignant neoplasm of bones of skull and face |
| C411 | Malignant neoplasm of mandible |
| C412 | Malignant neoplasm of vertebral column |
| C413 | Malignant neoplasm of ribssternum and clavicle |
| C414 | Malignant neoplasm of sacrum and coccyx |
| C418 | Malignant neoplasmoverlap lesion bon and articular cart |
| C419 | Malignant neoplasm of bone and articular cartilageunsp |
| C43 | Malignant melanoma of skin |
| C430 | Malignant melanoma of lip |
| C431 | Malignant melanoma of eyelidincluding canthus |
| C432 | Malignant melanoma of ear and ext auricular canal |
| C433 | Malignant melanoma of other and unspecified parts of face |
| C434 | Malignant melanoma of scalp and neck |
| C435 | Malignant melanoma of trunk |
| C436 | Malignant melanoma of upper limbincluding shoulder |
| C437 | Malignant melanoma of lower limbincluding hip |
| C438 | Malignant melanoma of skin |
| C439 | Malignant melanoma of skinunsp |
| C44 | Other malignant neoplasms of skin |
| C440 | Other malignant neoplasms of skin of lip |
| C441 | Other malignant neoplasms of skin of eyelidincl canthus |
| C442 | Oth malignant neoplasms of skin of ear & ext auricular canal |
| C443 | Oth malignant neoplasm of skin of oth & unsp parts of face |
| C444 | Other malignant neoplasms of skin of scalp and neck |
| C445 | Other malignant neoplasms of skin of trunk |
| C446 | Oth malignant neoplasms of skin of upper limbincl shoulder |
| C447 | Other malignant neoplasms of skin of lower limbincl hip |
| C448 | Other malignant neoplasmsoverlapping lesion of skin |
| C449 | Other malignant neoplasms of skinunspecified |
| C45 | Mesothelioma |
| C450 | Mesothelioma of pleura |
| C451 | Mesothelioma of peritoneum |
| C452 | Mesothelioma of pericardium |
| C457 | Mesothelioma of other sites |
| C459 | Mesotheliomaunspecified |
| C46 | Kaposi's sarcoma |
| C460 | Kaposi's sarcoma of skin |
| C461 | Kaposi's sarcoma of soft tissue |
| C462 | Kaposi's sarcoma of palate |
| C463 | Kaposi's sarcoma of lymph nodes |
| C467 | Kaposi's sarcoma of other sites |
| C468 | Kaposi's sarcoma of multiple organs |
| C469 | Kaposi's sarcomaunspecified |
| C47 | Malignant neo peripheral nerves and autonomic nervous syst |
| C470 | Malignant neoplasm of peripheral nerve of headface & neck |
| C471 | Malignant neoplasm of peripheral nerve |
| C472 | Malignant neoplasm of peripheral nerve of low limbincl hip |
| C473 | Malignant neoplasm of peripheral nerve of thorax |
| C474 | Malignant neoplasm of peripheral nerve of abdomen |
| C475 | Malignant neoplasm of peripheral nerve of pelvis |
| C476 | Malignant neoplasm of peripheral nerve of trunkunspec |
| C478 | Malignant neoplasm |
| C479 | Malignant neoplasm periph nerve & autonomic nsunspec |
| C48 | Malignant neoplasm of retroperitoneum and peritoneum |
| C480 | Malignant neoplasm of retroperitoneum |
| C481 | Malignant neoplasm of spec parts of peritoneum |
| C482 | Malignant neoplasm of peritoneumunsp |
| C488 | Malignant neoplasm of overlap lesion retroperit & peritoneum |
| C49 | Malignant neoplasm of other connective and soft tissue |
| C490 | Malignant neoplasm of conn and soft tiss headface & neck |
| C491 | Malignant neoplasm of conn and soft tiss upp limb |
| C492 | Malignant neoplasm of conn and soft tiss |
| C493 | Malignant neoplasm of conn and soft tiss of thorax |
| C494 | Malignant neoplasm of conn and soft tiss of abdomen |
| C495 | Malignant neoplasm of conn and soft tiss of pelvis |
| C496 | Malignant neoplasm of conn and soft tiss of trunkunsp |
| C498 | Malignant neoplasmoverlap lesion connective & soft tiss |
| C499 | Malignant neoplasm of connective and soft tissueunsp |
| C50 | Malignant neoplasm of breast |
| C500 | Malignant neoplasm of nipple and areola |
| C501 | Malignant neoplasm of central portion of breast |
| C502 | Malignant neoplasm of upper-inner quadrant of breast |
| C503 | Malignant neoplasm of lower-inner quadrant of breast |
| C504 | Malignant neoplasm of upper-outer quadrant of breast |
| C505 | Malignant neoplasm of lower-outer quadrant of breast |
| C506 | Malignant neoplasm of axillary tail of breast |
| C508 | Malignant neoplasmoverlapping lesion of breast |
| C509 | Malignant neoplasm of breastunspecified |
| C51 | Malignant neoplasm of vulva |
| C510 | Malignant neoplasm of labium majus |
| C511 | Malignant neoplasm of labium minus |
| C512 | Malignant neoplasm of clitoris |
| C518 | Malignant neoplasm of overlapping lesion of vulva |
| C519 | Malignant neoplasm of vulvaunspecified |
| C52 | Malignant neoplasm of vagina |
| C52X | Malignant neoplasm of vagina |
| C53 | Malignant neoplasm of cervix uteri |
| C530 | Malignant neoplasm of endocervix |
| C531 | Malignant neoplasm of exocervix |
| C538 | Malignant neoplasmoverlapping lesion of cervix uteri |
| C539 | Malignant neoplasm of cervix uteriunsp |
| C54 | Malignant neoplasm of corpus uteri |
| C540 | Malignant neoplasm of isthmus uteri |
| C541 | Malignant neoplasm of endometrium |
| C542 | Malignant neoplasm of myometrium |
| C543 | Malignant neoplasm of fundus uteri |
| C548 | Malignant neoplasm overlapping lesion of corpus uteri |
| C549 | Malignant neoplasm of corpus uteriunsp |
| C55 | Malignant neoplasm of uterus, part unspecified |
| C55X | Malignant neoplasm of uteruspart unspecified |
| C56 | Malignant neoplasm of ovary |
| C56X | Malignant neoplasm of ovary |
| C57 | Malignant neoplasm of other and unspec female genital orgs |
| C570 | Malignant neoplasm of fallopian tube |
| C571 | Malignant neoplasm of broad ligament |
| C572 | Malignant neoplasm of round ligament |
| C573 | Malignant neoplasm of parametrium |
| C574 | Malignant neoplasm of uterine adnexaunsp |
| C577 | Malignant neoplasm of other specified female genital organs |
| C578 | Malignant neoplasmoverlapping lesion female genital organs |
| C579 | Malignant neoplasm of female genital organunspecified |
| C58 | Malignant neoplasm of placenta |
| C58X | Malignant neoplasm of placenta |
| C60 | Malignant neoplasm of penis |
| C600 | Malignant neoplasm of prepuce |
| C601 | Malignant neoplasm of glans penis |
| C602 | Malignant neoplasm of body of penis |
| C608 | Malignant neoplasmoverlapping lesion of penis |
| C609 | Malignant neoplasm of penisunspecified |
| C61 | Malignant neoplasm of prostate |
| C61X | Malignant neoplasm of prostate |
| C62 | Malignant neoplasm of testis |
| C620 | Malignant neoplasm of undescended testis |
| C621 | Malignant neoplasm of descended testis |
| C629 | Malignant neoplasm of testisunspecified |
| C63 | Malignant neoplasm of other and unspec male genital organs |
| C630 | Malignant neoplasm of epididymis |
| C631 | Malignant neoplasm of spermatic cord |
| C632 | Malignant neoplasm of scrotum |
| C637 | Malignant neoplasm of other specified male genital orgs |
| C638 | Malignant neoplasmoverlapping lesion male genital orgs |
| C639 | Malignant neoplasm of male genital organunspecified |
| C64 | Malignant neoplasm of kidney, except renal pelvis |
| C64X | Malignant neoplasm of kidneyexcept renal pelvis |
| C65 | Malignant neoplasm of renal pelvis |
| C65X | Malignant neoplasm of renal pelvis |
| C66 | Malignant neoplasm of ureter |
| C66X | Malignant neoplasm of ureter |
| C67 | Malignant neoplasm of bladder |
| C670 | Malignant neoplasm of trigone of bladder |
| C671 | Malignant neoplasm of dome of bladder |
| C672 | Malignant neoplasm of lateral wall of bladder |
| C673 | Malignant neoplasm of anterior wall of bladder |
| C674 | Malignant neoplasm of posterior wall of bladder |
| C675 | Malignant neoplasm of bladder neck |
| C676 | Malignant neoplasm of ureteric orifice |
| C677 | Malignant neoplasm of urachus |
| C678 | Malignant neoplasmoverlapping lesion of bladder |
| C679 | Malignant neoplasm of bladderunspecified |
| C68 | Malignant neoplasm of other and unspecified urinary organs |
| C680 | Malignant neoplasm of urethra |
| C681 | Malignant neoplasm of paraurethral gland |
| C688 | Malignant neoplasm of overlapping lesion urinary organs |
| C689 | Malignant neoplasm of urinary organunspecified |
| C69 | Malignant neoplasm of eye and adnexa |
| C690 | Malignant neoplasm of conjunctiva |
| C691 | Malignant neoplasm of cornea |
| C692 | Malignant neoplasm of retina |
| C693 | Malignant neoplasm of choroid |
| C694 | Malignant neoplasm of ciliary body |
| C695 | Malignant neoplasm of lacrimal gland and duct |
| C696 | Malignant neoplasm of orbit |
| C698 | Malignant neoplasmoverlapping lesion eye and adnexa |
| C699 | Malignant neoplasm of eyeunspecified |
| C70 | Malignant neoplasm of meninges |
| C700 | Malignant neoplasm ofcerebral meninges |
| C701 | Malignant neoplasm of spinal meninges |
| C709 | Malignant neoplasm of meningesunspecified |
| C71 | Malignant neoplasm of brain |
| C710 | Malignant neoplasm of cerebrumexcept lobes & ventricles |
| C711 | Malignant neoplasm of cerebrumfrontal lobe |
| C712 | Malignant neoplasm of cerebrumtemporal lobe |
| C713 | Malignant neoplasm of cerebrumparietal lobe |
| C714 | Malignant neoplasm of cerebrumoccipital lobe |
| C715 | Malignant neoplasm of cerebrumcerebral ventricle |
| C716 | Malignant neoplasm of cerebrumcerebellum |
| C717 | Malignant neoplasm of cerebrumbrain stem |
| C718 | Malignant neoplasm of cerebrumoverlapping lesion of brain |
| C719 | Malignant neoplasm of cerebrumbrainunspecified |
| C72 | Malig neopl spinal cord, cranial nerves & oth parts of CNS |
| C720 | Malignant neoplasm of spinal cord |
| C721 | Malignant neoplasm of cauda equina |
| C722 | Malignant neoplasm of Olfactory nerve |
| C723 | Malignant neoplasm of Optic nerve |
| C724 | Malignant neoplasm of Acoustic nerve |
| C725 | Malignant neoplasm of other and unspecified cranial nerves |
| C728 | Malignant neoplasmoverlapping lesion brain&other part CNS |
| C729 | Malignant neoplasm of Central Nervous Systemunspecified |
| C73 | Malignant neoplasm of thyroid gland |
| C73X | Malignant neoplasm of thyroid gland |
| C74 | Malignant neoplasm of adrenal gland |
| C740 | Malignant neoplasm of cortex of adrenal gland |
| C741 | Malignant neoplasm of medulla of adrenal gland |
| C749 | Malignant neoplasm of adrenal glandunsp |
| C75 | Malignant neo other endocrine glands and related structures |
| C750 | Malignant neoplasm of parathyroid gland |
| C751 | Malignant neoplasm of pituitary gland |
| C752 | Malignant neoplasm of craniopharyngeal duct |
| C753 | Malignant neoplasm of pineal gland |
| C754 | Malignant neoplasm of carotid body |
| C755 | Malignant neoplasm of aortic body and other paraganglia |
| C758 | Malignant neoplasmpluriglandular involvmentunspecified |
| C759 | Malignant neoplasm of endocrine glandunspecified |
| C76 | Malignant neoplasm of other and ill-defined sites |
| C760 | Malignant neoplasm of headface & neck |
| C761 | Malignant neoplasm of thorax |
| C762 | Malignant neoplasm of abdomen |
| C763 | Malignant neoplasm of pelvis |
| C764 | Malignant neoplasm of upper limb |
| C765 | Malignant neoplasm of lower limb |
| C767 | Malignant neoplasm of other ill-defined sites |
| C768 | Malignant neoplasmoverlap lesion oth & ill-defined sites |
| C80 | Malignant neoplasm without specification of site |
| C80X | Malignant neoplasm without specification of site |
| C81 | Hodgkin's disease |
| C810 | Hodgkin's diseaselymphocytic predominance |
| C811 | Hodgkin's diseasenodular sclerosis |
| C812 | Hodgkin's diseasemixed cellularity |
| C813 | Hodgkin's diseaselymphocytic depletion |
| C817 | Hodgkin's diseaseother Hodgkin's disease |
| C819 | Hodgkin's diseaseHodgkin's diseaseunspecified |
| C82 | Follicular [nodular] non-Hodgkin's lymphoma |
| C820 | Follicular non-Hodgkin's small cleaved cell lymphoma |
| C821 | Follicular non-Hodg mixed sml cleavd & lge cell lymphoma |
| C822 | Follicular non-Hodgkin's large cell lymphoma |
| C827 | Follicular non-Hodgkin's other types of lymphoma |
| C829 | Follicular non-Hodgkin's unspecified lymphoma |
| C83 | Diffuse non-Hodgkin's lymphoma |
| C830 | Diffuse non-Hodgkin's small cell (diffuse)lymphoma |
| C831 | Diffuse non-Hodgkin's small cleaved cell (diffuse) lymphoma |
| C832 | Diffuse non-Hodgkin mixed sml & lge cell (diffuse) lymphoma |
| C833 | Diffuse non-Hodgkin's large cell (diffuse) lymphoma |
| C834 | Diffuse non-Hodgkin's immunoblastic (diffuse) lymphoma |
| C835 | Diffuse non-Hodgkin's lymphoblastic (diffuse) lymphoma |
| C836 | Diffuse non-Hodgkin's lymphoma undifferentiated (diffuse) |
| C837 | Diffuse non-Hodgkin's lymphomaBurkitt's tumour |
| C838 | Other types of diffuse non-Hodgkin's lymphoma |
| C839 | Diffuse non-Hodgkin's lymphomaunspecified |
| C84 | Peripheral and cutaneous T-cell lymphomas |
| C840 | Peripheral and cutaneous T-cell lymphomasmycosis fungoides |
| C841 | Peripheral and cutaneous T-cell lymphomasSezary's disease |
| C842 | Peripheral and cutaneous T-cell lymphomasT-zone lymphoma |
| C843 | Periph & cutan T-cell lymphomaslymphoepithelioid lymphoma |
| C844 | Periph & cutan T-cell lymphomasperipheral T-cell lymphoma |
| C845 | Periph & cutan T-cell lymphomasoth & unsp T-cell lymphomas |
| C85 | Other and unspecified types of non-Hodgkin's lymphoma |
| C850 | Oth & unspec types of non-Hodgkin's lymphomalymphosarcoma |
| C851 | Oth & unsp types non-Hodgkin's B-cell lymphomaunsp |
| C857 | Oth specified types of non-Hodgkin's lymphoma |
| C859 | Non-Hodgkin's lymphomaunspecified type |
| C880 | Waldenstrom's macroglobulinaemia |
| C881 | Alpha heavy chain disease |
| C882 | Gamma heavy chain disease |
| C883 | Malignant immunoproliferative small intestinal disease |
| C887 | Other malignant immunoproliferative diseases |
| C889 | Malignant immunoproliferative diseaseunspecified |
| C900 | Multiple myeloma |
| C901 | Plasma cell leukaemia |
| C902 | Malignant plasma cell neoplasmextramedullary plasmacytoma |
| C91 | Lymphoid leukaemia |
| C910 | Acute lymphoblastic leukaemia |
| C911 | Chronic lymphocytic leukaemia |
| C912 | Subacute lymphocytic leukaemia |
| C913 | Prolymphocytic leukaemia |
| C914 | Hairy-cell leukaemia |
| C915 | Adult T-cell leukaemia |
| C917 | Other lymphoid leukaemia |
| C919 | Lymphoid leukaemiaunspecified |
| C92 | Myeloid leukaemia |
| C920 | Acute myeloid leukaemia |
| C921 | Chronic myeloid leukaemia |
| C922 | Subacute myeloid leukaemia |
| C923 | Myeloid sarcoma |
| C924 | Acute promyelocytic leukaemia |
| C925 | Acute myelomonocytic leukaemia |
| C927 | Other myeloid leukaemia |
| C929 | Myeloid leukaemiaunspecified |
| C93 | Monocytic leukaemia |
| C930 | Acute monocytic leukaemia |
| C931 | Chronic monocytic leukaemia |
| C932 | Subacute monocytic leukaemia |
| C937 | Other monocytic leukaemia |
| C939 | Monocytic leukaemiaunspecified |
| C940 | Acute erythraemia & erythroleukaemia |
| C941 | Chronic erythraemia |
| C942 | Acute megakaryoblastic leukaemia |
| C943 | Mast cell leukaemia |
| C944 | Acute panmyelosis |
| C945 | Acute myelofibrosis |
| C947 | Other specified leukaemias |
| C95 | Leukaemia of unspecified cell type |
| C950 | Acute leukaemia of unsp cell type |
| C951 | Chronic leukaemia unsp cell type |
| C952 | Subacute leukaemia unsp cell type |
| C957 | Other leukaemia unspecified cell type |
| C959 | Leukaemiaunspecified |
| C96 | Oth & unspec malig neop lymphoid, haematapoietic & rel tiss |
| C960 | Letterer-Siwe disease |
| C961 | Malignant histiocytosis |
| C962 | Malignant mast cell tumour |
| C963 | True histiocyt lymphoma |
| C967 | Oth spec malig neop lymphoid h'poietic & related tissue |
| C969 | Malig neop lymphoid haematopoietic and related tissue unspec |
| C97 | Malignant neoplasms of independent (primary) multiple sites |
| C97X | Malignant neoplasms of independent (primary) multiple sites |

**Table 4: Cancer READ codes**

| READ | Description |
| --- | --- |
| B0... | Malig neop lip, oral, pharynx |
| B00.. | Malig neop of lip |
| B000. | Malig neop upp lip,vermil bord |
| B0000 | Malig neop upp lip, external |
| B0001 | Malig neop upp lip, lipstick |
| B000z | Malig neop upp lip, vermil NOS |
| B001. | Malig neop low lip,vermil bord |
| B0010 | Malig neop lower lip, external |
| B0011 | Malig neop lower lip, lipstick |
| B001z | Malig neop low lip, vermil NOS |
| B002. | Malig neop upp lip,inn aspect |
| B0020 | Malig neop upp lip,bucc aspect |
| B0021 | Malig neop upper lip, frenulum |
| B0022 | Malig neop upper lip, mucosa |
| B0023 | Malig neop upp lip,oral aspect |
| B002z | Malig neop upp lip, inn aspect |
| B003. | Malig neop low lip,inn aspect |
| B0030 | Malig neop low lip,bucc aspect |
| B0031 | Malig neop lower lip, frenulum |
| B0032 | Malig neop lower lip, mucosa |
| B0033 | Malig neop low lip,oral aspect |
| B003z | Malig neop low lip, inn aspect |
| B004. | Malig neop lip unsp,inn aspect |
| B0040 | Malig neop lip, unsp, bucc asp |
| B0041 | Malig neop lip, unsp, frenulum |
| B0042 | Malig neop lip, unsp, mucosa |
| B0043 | Malig neop lip, oral aspect |
| B004z | Malig neop lip inner asp NOS |
| B005. | Malig neop commissure of lip |
| B006. | Malig neop, overlap lesn lip |
| B007. | Malignant neoplasm lip, unsp |
| B00y. | Malig neop other sites of lip |
| B00z. | Malig neop lip,unsp verm bord |
| B00z0 | Malig neop lip, unsp, external |
| B00z1 | Malig neop lip, unsp, lipstick |
| B00zz | Malig neop lip,verm.border NOS |
| B01.. | Malignant neoplasm of tongue |
| B010. | Malig neop base of tongue |
| B0100 | Malig neop tongue base dorsal |
| B010z | Malig neop fixed tongue NOS |
| B011. | Malig neop dorsal tongue |
| B0110 | Malig neop ant 2/3 tong.dorsal |
| B0111 | Malig neop midline of tongue |
| B011z | Malig neop dorsum tongue NOS |
| B012. | Malig neop tongue tip/lat bord |
| B013. | Malig neop ventral tongue |
| B0130 | Mali neop ant 2/3 tongue vent |
| B0131 | Malig neop frenulum linguae |
| B013z | Malig neop ventral tongue NOS |
| B014. | Malig neop ant 2/3 tongue unsp |
| B015. | Malig neop tongue junct zone |
| B016. | Malig neop lingual tonsil |
| B017. | Malignan overlap lesion tongue |
| B01y. | Malig neop other sites tongue |
| B01z. | Malig neop tongue NOS |
| B02.. | Malig neop major saliv glands |
| B020. | Malig neop parotid gland |
| B021. | Malig neop submandibular gland |
| B022. | Malig neop sublingual gland |
| B023. | Mal neop,ovlap lesn maj sal gl |
| B02y. | Malig neop oth major sal gland |
| B02z. | Malig neop major sal gland NOS |
| B03.. | Malignant neoplasm of gum |
| B030. | Malig neop of upper gum |
| B031. | Malig neop of lower gum |
| B03y. | Malig neop other sites of gum |
| B03z. | Malig neop of gum NOS |
| B04.. | Malignant neoplasm mouth floor |
| B040. | Malig neop ant, mouth floor |
| B041. | Malig neop lat, mouth floor |
| B042. | Mal neop,ovlap les floor mouth |
| B04y. | Malig neop oth sit,mouth floor |
| B04z. | Malig neop floor of mouth NOS |
| B05.. | Malig neop oth+unsp mouth part |
| B050. | Malig neop cheek mucosa |
| B051. | Malig neop vestibule of mouth |
| B0510 | Malig neop upper buccal sulcus |
| B0511 | Malig neop lower buccal sulcus |
| B0512 | Malig neop upper labial sulcus |
| B0513 | Malig neop lower labial sulcus |
| B051z | Malig neop vestibule mouth NOS |
| B052. | Malig neop hard palate |
| B053. | Malig neop soft palate |
| B054. | Malig neop uvula |
| B055. | Malig neop palate unspecified |
| B0550 | Malig neop hard/soft palat jct |
| B0551 | Malig neop roof of mouth |
| B055z | Malig neop palate NOS |
| B056. | Malig neop retromolar area |
| B057. | Overlap les oth+unspec mouth |
| B05y. | Malig neop oth spec mouth part |
| B05z. | Malig neop mouth NOS |
| B05z0 | Kaposi's sarcoma of palate |
| B06.. | Malignant neoplasm oropharynx |
| B060. | Malig neop of tonsil |
| B0600 | Malig neop faucial tonsil |
| B0601 | Malig neop palatine tonsil |
| B0602 | Mal neopl overlap lesn tonsil |
| B060z | Malignant neoplasm tonsil NOS |
| B061. | Malig neop of tonsillar fossa |
| B062. | Malig neop of tonsillar pillar |
| B0620 | Malig neop faucial pillar |
| B0621 | Malig neop glossopalatine fold |
| B0622 | Malig neop palatoglossal arch |
| B0623 | Malig neop palatopharyng arch |
| B062z | Malig neop tonsillar fossa NOS |
| B063. | Malig neop of vallecula |
| B064. | Malig neop of ant epiglottis |
| B0640 | Malig neop epiglot free border |
| B0641 | Malig neop glossoepiglott fold |
| B064z | Malig neop ant epiglottis NOS |
| B065. | Malig neop junct reg. epiglott |
| B066. | Malig neop lat wall oropharynx |
| B067. | Malig neop post wall orophary. |
| B06y. | Malig neop oroph oth spec site |
| B06y0 | Malig neop branchial cleft |
| B06yz | Malig neop oroph oth site NOS |
| B06z. | Malig neop oropharynx NOS |
| B07.. | Malignant neoplasm nasopharynx |
| B070. | Malig neop roof of nasopharynx |
| B071. | Malig neop post wall nasophar. |
| B0710 | Malig neop adenoid |
| B0711 | Malig neop pharyngeal tonsil |
| B071z | Malig neop post nasophar NOS |
| B072. | Malig neop lat wall nasopharyn |
| B0720 | Malig neop pharyngeal recess |
| B0721 | Malig neop opening audit. tube |
| B072z | Malig neop lat wall nasoph NOS |
| B073. | Malig neop ant wall nasopharyn |
| B0730 | Malig neop floor nasopharynx |
| B0731 | Malig neop nasoph. soft palate |
| B0732 | Malig neop post septum/choanae |
| B073z | Malig neop ant wall nasoph NOS |
| B074. | Mal neop,overlap lesn nasophar |
| B07y. | Malig neop oth spec nasop site |
| B07z. | Malig neop nasopharynx NOS |
| B08.. | Malignant neoplasm hypopharynx |
| B080. | Malig neop postcricoid region |
| B081. | Malig neop pyriform sinus |
| B082. | Malig neop aryepigl fold,hypop |
| B083. | Malig neop posterior pharynx |
| B084. | Mal neop overlap lesn hypophar |
| B08y. | Malig neop oth spec hypop site |
| B08z. | Malig neop hypopharynx NOS |
| B0z.. | Malig neop oth lip,oral,pharyn |
| B0z0. | Malig neop pharynx unspecified |
| B0z1. | Malig neop Waldeyer's ring |
| B0z2. | Malig neoplasm laryngopharynx |
| B0zy. | Malig neop oth lip,oral,phary |
| B0zz. | Malig neop lip,oral,phary NOS |
| B1... | Malignant neoplasm GIT |
| B10.. | Malig neop oesophagus |
| B100. | Malig neop cervical oesophagus |
| B101. | Malig neop thoracic oesophagus |
| B102. | Malig neop abdominal oesophag |
| B103. | Malig neop upp 1/3 oesophagus |
| B104. | Malig neop mid 1/3 oesophagus |
| B105. | Malig neop low 1/3 oesophagus |
| B106. | Mal neopl overlap lesn oesoph |
| B107. | Siewert type I adenocarcinoma |
| B10y. | Malig neop oth spec part oesop |
| B10z. | Malig neop oesophagus NOS |
| B11.. | Malig neop of stomach |
| B110. | Malig neop cardia of stomach |
| B1100 | Malig neop card orific stomach |
| B1101 | Malig neop cardio-oesoph junct |
| B110z | Malig neop cardia stomach NOS |
| B111. | Malig neop pylorus of stomach |
| B1110 | Malig neop prepylorus stomach |
| B1111 | Malig neop pylor canal stomach |
| B111z | Malig neop pylorus stomach NOS |
| B112. | Malig neop pyloric ant stomach |
| B113. | Malig neop fundus of stomach |
| B114. | Malig neop body of stomach |
| B115. | Malig neop less curv stom unsp |
| B116. | Malig neop great curv stom uns |
| B117. | Malig neopl overlap lesn stom |
| B118. | Siewert type II adenocarcinoma |
| B119. | Siewert type III adenocarcino |
| B11y. | Malig neop oth spec stomach |
| B11y0 | Malig neop ant wall stom NOS |
| B11y1 | Malig neop post wall stom NEC |
| B11yz | Malig neop oth spec stom NOS |
| B11z. | Malignant neoplasm stomach NOS |
| B12.. | Malig neop small intestine |
| B120. | Malig neop of duodenum |
| B121. | Malig neop of jejunum |
| B122. | Malig neop of ileum |
| B123. | Malig neop Meckel's divertic |
| B124. | Mal neop overlap les small int |
| B12y. | Malig neop oth spec small int |
| B12z. | Malig neop small intestine NOS |
| B13.. | Malig neop of colon |
| B130. | Malig neop hepatic flex colon |
| B131. | Malig neop of transverse colon |
| B132. | Malig neop of descending colon |
| B133. | Malig neop of sigmoid colon |
| B134. | Malig neop of caecum |
| B135. | Malig neop of appendix |
| B136. | Malig neop of ascending colon |
| B137. | Malig neop splenic flex colon |
| B138. | Mal neop overlap lesn of colon |
| B139. | Heredit nonpolypos colon cancr |
| B13y. | Malig neop oth spec site colon |
| B13z. | Malig neop of colon NOS |
| B14.. | Malig neop of rectum and anus |
| B140. | Malig neop rectosigmoid junct |
| B141. | Malig neop of rectum |
| B142. | Malig neop of anal canal |
| B1420 | Mal neopl of cloacogenic zone |
| B143. | Malig neop of anus unspecified |
| B14y. | Malig neop oth rectum and anus |
| B14z. | Malig neop rectum and anus NOS |
| B15.. | Malig neop liver/intrahep duct |
| B150. | Primary malig neop of liver |
| B1500 | Primary carcinoma of liver |
| B1501 | Hepatoblastoma of liver |
| B1502 | Primary angiosarcoma of liver |
| B1503 | Hepatocellular carcinoma |
| B150z | Primary malig neop liver NOS |
| B151. | Malig neop intrahep bile ducts |
| B1510 | Malig neop interlob bile ducts |
| B1511 | Malig neop interlob bil canals |
| B1512 | Malig neop intrahep bil passag |
| B1513 | Malig neop intrahep canaliculi |
| B1514 | Malig neop intrahep gall duct |
| B151z | Malig neop intrahep ducts NOS |
| B152. | Malig neop liver unspecified |
| B153. | Secondary malig neopl of liver |
| B15z. | Malig neop liver/intrahep NOS |
| B16.. | Malig neop GB/extrahep ducts |
| B160. | Malig neop of gallbladder |
| B161. | Malig neop extrahep bile ducts |
| B1610 | Malig neop cystic duct |
| B1611 | Malig neop hepatic duct |
| B1612 | Malig neop common bile duct |
| B1613 | Malig neop sphincter of Oddi |
| B161z | Malig neop extrahep ducts NOS |
| B162. | Malig neop ampulla of Vater |
| B163. | Mal neop overlap lesn bil trac |
| B16y. | Malig neop oth GB/extrahep bil |
| B16z. | Malig neop GB/extrahep bil NOS |
| B17.. | Malignant neoplasm of pancreas |
| B170. | Malig neop head of pancreas |
| B171. | Malig neop body of pancreas |
| B172. | Malig neop tail of pancreas |
| B173. | Malig neop pancreatic duct |
| B174. | Malig neop Islets Langerhans |
| B175. | Malig neopl overlap lesn pancr |
| B176. | Somatostatinoma of pancreas |
| B17y. | Malig neop oth spec pancreas |
| B17y0 | Malig neop ectop pancreat tiss |
| B17yz | Malig neop spec site panc NOS |
| B17z. | Malig neop of pancreas NOS |
| B18.. | Malig neop retroperit/periton |
| B180. | Malig neop of retroperitoneum |
| B1800 | Malig neop periadrenal tissue |
| B1801 | Malig neop perinephric tissue |
| B1802 | Malig neop retrocaecal tissue |
| B180z | Malig neop retroperitoneum NOS |
| B181. | Mesothelioma of peritoneum |
| B182. | Overlap les retroperit+periton |
| B18y. | Malig neop spec part periton |
| B18y0 | Malig neop of mesocolon |
| B18y1 | Malig neop of mesocaecum |
| B18y2 | Malig neop of mesorectum |
| B18y3 | Malig neop of omentum |
| B18y4 | Malig neop parietal peritoneum |
| B18y5 | Malig neop pelvic peritoneum |
| B18y6 | Malig neop pouch of Douglas |
| B18y7 | Malignant neoplasm mesentery |
| B18yz | Malig neop spec perit part NOS |
| B18z. | Malig neop retroper/perit NOS |
| B1z.. | Malig neop GIT other/ill-defin |
| B1z0. | Malig neop intest tract unspec |
| B1z1. | Malig neop spleen NEC |
| B1z10 | Angiosarcoma of spleen |
| B1z11 | Fibrosarcoma of spleen |
| B1z1z | Malig neop spleen NOS |
| B1z2. | Mal neopl,overlap lesn dig sys |
| B1zy. | Malig neop oth spec GIT part |
| B1zz. | Malignant GIT neoplasm NOS |
| B2... | Malig neop resp/intrath. organ |
| B20.. | Malig neop nose,mid ear,sinus |
| B200. | Malig neop nasal cavities |
| B2000 | Malig neop cartilage of nose |
| B2001 | Malig neop nasal conchae |
| B2002 | Malig neop septum of nose |
| B2003 | Malig neop vestibule of nose |
| B200z | Malig neop nasal cavities NOS |
| B201. | Malig neop middle ear |
| B2010 | Malig neop eustachian tube |
| B2011 | Malig neop tympanic cavity |
| B2012 | Malig neop tympanic antrum |
| B2013 | Malig neop mastoid air cells |
| B201z | Malig neop middle ear NOS |
| B202. | Malig neop maxillary sinus |
| B203. | Malig neop ethmoidal sinus |
| B204. | Malig neop frontal sinus |
| B205. | Malig neop sphenoidal sinus |
| B206. | Mal neop, ovlap lesn acc sinus |
| B20y. | Malig neop oth nose,ear,sinus |
| B20z. | Malig neop accessory sinus NOS |
| B21.. | Malig neop larynx |
| B210. | Malig neop glottis |
| B211. | Malig neop supraglottis |
| B212. | Malig neop subglottis |
| B213. | Malig neop laryngeal cartilage |
| B2130 | Malig neop arytenoid cartilage |
| B2131 | Malig neop cricoid cartilage |
| B2132 | Malig neop cuneiform cartilage |
| B2133 | Malig neop thyroid cartilage |
| B213z | Malig neop laryngeal cart NOS |
| B214. | Mal neopl, overlap lesn larynx |
| B215. | Mal neoplasm of epiglottis NOS |
| B21y. | Malig neop larynx other specif |
| B21z. | Malig neop larynx NOS |
| B22.. | Malig neop trachea/bronch/lung |
| B220. | Malig neop trachea |
| B2200 | Malig neop cartilage trachea |
| B2201 | Malig neop mucosa of trachea |
| B220z | Malig neop trachea NOS |
| B221. | Malig neop main bronchus |
| B2210 | Malig neop carina of bronchus |
| B2211 | Malig neop hilus of lung |
| B221z | Malig neop main bronchus NOS |
| B222. | Malig neop upp lobe bronc/lung |
| B2220 | Malig neop upper lobe bronchus |
| B2221 | Malig neop upper lobe of lung |
| B222z | Malig neop upp bronc/lung NOS |
| B223. | Malig neop mid lobe bronc/lung |
| B2230 | Malig neop mid lobe bronchus |
| B2231 | Malig neop middle lobe of lung |
| B223z | Malig neop mid lobe bronc/lung |
| B224. | Malig neop low lobe bronc/lung |
| B2240 | Malig neop lower bronchus |
| B2241 | Malig neop lower lobe of lung |
| B224z | Malig neop low lobe bronc/lung |
| B225. | Mal neop,overlap les bron/lung |
| B226. | Mesothelioma |
| B22y. | Malig neop oth site bronc/lung |
| B22z. | Malig neop bronchus/lung NOS |
| B23.. | Malig neop pleura |
| B230. | Malig neop parietal pleura |
| B231. | Malig neop visceral pleura |
| B232. | Mesothelioma of pleura |
| B23y. | Malig neop oth specif pleura |
| B23z. | Malig neop pleura NOS |
| B24.. | Malig neop heart/thym/mediasti |
| B240. | Malig neop thymus |
| B241. | Malig neop heart |
| B2410 | Malig neop endocardium |
| B2411 | Malig neop epicardium |
| B2412 | Malig neop myocardium |
| B2413 | Malig neop pericardium |
| B2414 | Mesothelioma of pericardium |
| B241z | Malig neop heart NOS |
| B242. | Malig neop ant mediastinum |
| B243. | Malig neop post mediastinum |
| B24X. | Mal neo mediastin,part uns |
| B24y. | Malig neop oth hrt/thym/medias |
| B24z. | Malig neop hrt/thym/medias NOS |
| B25.. | Malig neop ovlap heart/med/pl |
| B26.. | Malig neop ovlap resp/thc org |
| B2z.. | Malig neop oth resp/intrathor |
| B2z0. | Malig neop up resp tract unspe |
| B2zy. | Malig neop other resp tract |
| B2zz. | Malig neop resp tract NOS |
| B3... | Malig neop bone/skin/breast |
| B30.. | Malig neop bone/articular cart |
| B300. | Malig neop bones of skull/face |
| B3000 | Malig neop ethmoid bone |
| B3001 | Malig neop frontal bone |
| B3002 | Malig neop malar bone |
| B3003 | Malig neop nasal bone |
| B3004 | Malig neop occipital bone |
| B3005 | Malig neop orbital bone |
| B3006 | Malig neop parietal bone |
| B3007 | Malig neop sphenoid bone |
| B3008 | Malig neop temporal bone |
| B3009 | Malig neop zygomatic bone |
| B300A | Malig neop maxilla |
| B300B | Malig neop turbinate |
| B300C | Malig neop vomer |
| B300z | Malig neop skull/face NOS |
| B301. | Malig neop mandible |
| B302. | Malig neop vertebral column |
| B3020 | Malig neop cervical vertebra |
| B3021 | Malig neop thoracic vertebra |
| B3022 | Malig neop lumbar vertebra |
| B302z | Malig neop vertebral col NOS |
| B303. | Malig neop rib/sternum/clavicl |
| B3030 | Malig neop rib |
| B3031 | Malig neop sternum |
| B3032 | Malig neop clavicle |
| B3033 | Malig neop costal cartilage |
| B3034 | Malig neop costo-vertebral jnt |
| B3035 | Malig neop xiphoid process |
| B303z | Malig neop rib/stern/clav NOS |
| B304. | Malig neop scap/arm long bone |
| B3040 | Malig neop scapula |
| B3041 | Malig neop acromion |
| B3042 | Malig neop humerus |
| B3043 | Malig neop radius |
| B3044 | Malig neop ulna |
| B304z | Malig neop scap/arm bone NOS |
| B305. | Malig neop hand bones |
| B3050 | Malig neop carpal - scaphoid |
| B3051 | Malig neop carpal - lunate |
| B3052 | Malig neop carpal - triquetrum |
| B3053 | Malig neop carpal - pisiform |
| B3054 | Malig neop carpal - trapezium |
| B3055 | Malig neop carpal - trapezoid |
| B3056 | Malig neop carpal - capitate |
| B3057 | Malig neop carpal - hamate |
| B3058 | Malig neop first metacarpal |
| B3059 | Malig neop second metacarpal |
| B305A | Malig neop third metacarpal |
| B305B | Malig neop fourth metacarpal |
| B305C | Malig neop fifth metacarpal |
| B305D | Malig neop phalanges of hand |
| B305z | Malig neop hand bones NOS |
| B306. | Malig neop pelvis/sacrum/coccy |
| B3060 | Malig neop ilium |
| B3061 | Malig neop ischium |
| B3062 | Malig neop pubis |
| B3063 | Malig neop sacral vertebra |
| B3064 | Malig neop coccygeal vertebra |
| B3065 | Malignant sacral teratoma |
| B306z | Malig neop pelvis/sacrum/coccy |
| B307. | Malig neop long bones of leg |
| B3070 | Malig neop femur |
| B3071 | Malig neop fibula |
| B3072 | Malig neop tibia |
| B307z | Malig neop long bone leg NOS |
| B308. | Malig neop short bones of leg |
| B3080 | Malig neop patella |
| B3081 | Malig neop talus |
| B3082 | Malig neop calcaneum |
| B3083 | Malig neop medial cuneiform |
| B3084 | Malig neop intermed cuneiform |
| B3085 | Malig neop lateral cuneiform |
| B3086 | Malig neop cuboid |
| B3087 | Malig neop navicular |
| B3088 | Malig neop first metatarsal |
| B3089 | Malig neop second metatarsal |
| B308A | Malig neop third metatarsal |
| B308B | Malig neop fourth metatarsal |
| B308C | Malig neop fifth metatarsal |
| B308D | Malig neop phalanges of foot |
| B308z | Malig neop short bone leg NOS |
| B309. | Malig neop ovlap bone/car lim |
| B30W. | Mal neo/ovlap les/bne+art c |
| B30X. | Mal neo/bne+art c/limb,unsp |
| B30z. | Malig neop bone/art cart NOS |
| B30z0 | Osteosarcoma |
| B31.. | Malig neop connect/oth tissue |
| B310. | Malig neop soft tiss head/neck |
| B3100 | Malig neop soft tissue of head |
| B3101 | Malig neop soft tissue of face |
| B3102 | Malig neop soft tissue of neck |
| B3103 | Malig neop cartilage of ear |
| B3104 | Malig neop tarsus of eyelid |
| B3105 | Malignancy, soft tiss-Cx spine |
| B310z | Malig neop tiss head/neck NOS |
| B311. | Malig neop soft tis arm/should |
| B3110 | Malig neop soft tiss shoulder |
| B3111 | Malig neop soft tiss upper arm |
| B3112 | Malig neop soft tiss fore-arm |
| B3113 | Malig neop soft tissue hand |
| B3114 | Malig neop soft tissue finger |
| B3115 | Malig neop soft tissue thumb |
| B311z | Malig neop tis should/arm NOS |
| B312. | Malig neop soft tissue hip/leg |
| B3120 | Malig neop soft tissue hip |
| B3121 | Malig neop tis thigh/upper leg |
| B3122 | Malig neop tiss poplit space |
| B3123 | Malig neop soft tiss lower leg |
| B3124 | Malig neop soft tissue foot |
| B3125 | Malig neop soft tissue toe |
| B3126 | Malig neop soft tiss great toe |
| B312z | Malig neop tissue hip/leg NOS |
| B313. | Malig neop soft tissue thorax |
| B3130 | Malig neop soft tissue axilla |
| B3131 | Malig neop diaphragm |
| B3132 | Malig neop great vessels |
| B3133 | Malignancy, soft tiss-th spine |
| B313z | Malig neop soft tis thorax NOS |
| B314. | Malig neop soft tissue abdomen |
| B3140 | Malig neop tissue abdo wall |
| B3141 | Malignancy, soft tiss-lu spine |
| B314z | Malig neop tissue abdomen NOS |
| B315. | Malig neop soft tissue pelvis |
| B3150 | Malig neop soft tissue buttock |
| B3151 | Malig neop tis inguinal region |
| B3152 | Malig neop tissue perineum |
| B3153 | Malignancy, soft tiss-sac+cocc |
| B315z | Malig neop tissue pelvis NOS |
| B316. | Malig neop tissue trunk unspec |
| B317. | Malig neop ovlap con/soft tis |
| B31y. | Malig neop oth spec soft tiss |
| B31z. | Malig neop soft tissue NOS |
| B31z0 | Kaposi's sarc of soft tissue |
| B32.. | Malignant melanoma of skin |
| B320. | Malignant melanoma of lip |
| B321. | Malignant melanoma of eyelid |
| B322. | Malignant melanoma of ear |
| B3220 | Malignant melanoma ear auricle |
| B3221 | Malig melanoma ext. ear canal |
| B322z | Malignant melanoma of ear NOS |
| B323. | Malig melanoma other part face |
| B3230 | Malignant melanoma cheek, ext |
| B3231 | Malignant melanoma of chin |
| B3232 | Malignant melanoma of eyebrow |
| B3233 | Malignant melanoma of forehead |
| B3234 | Malignant melanoma of nose,ext |
| B3235 | Malignant melanoma of temple |
| B323z | Malignant melanoma of face NOS |
| B324. | Malignant melanoma scalp/neck |
| B3240 | Malignant melanoma of scalp |
| B3241 | Malignant melanoma of neck |
| B324z | Malig melanoma scalp/neck NOS |
| B325. | Malig melan trunk ex scrotum |
| B3250 | Malignant melanoma of axilla |
| B3251 | Malignant melanoma of breast |
| B3252 | Malignant melanoma of buttock |
| B3253 | Malignant melanoma of groin |
| B3254 | Malig melan of perianal skin |
| B3255 | Malignant melanoma of perineum |
| B3256 | Malignant melanoma umbilicus |
| B3257 | Malignant melanoma of back |
| B3258 | Malignant melanoma chest wall |
| B325z | Malignant melanoma trunk NOS |
| B326. | Malig melan of arm/shoulder |
| B3260 | Malignant melanoma of shoulder |
| B3261 | Malignant melanoma upper arm |
| B3262 | Malignant melanoma of fore-arm |
| B3263 | Malignant melanoma of hand |
| B3264 | Malignant melanoma of finger |
| B3265 | Malignant melanoma of thumb |
| B326z | Malig melan arm/shoulder NOS |
| B327. | Malignant melanoma of leg/hip |
| B3270 | Malignant melanoma of hip |
| B3271 | Malignant melanoma of thigh |
| B3272 | Malignant melanoma of knee |
| B3273 | Malig melanoma-popliteal area |
| B3274 | Malignant melanoma lower leg |
| B3275 | Malignant melanoma of ankle |
| B3276 | Malignant melanoma of heel |
| B3277 | Malignant melanoma of foot |
| B3278 | Malignant melanoma of toe |
| B3279 | Malignant melanoma great toe |
| B327z | Malignant melanoma leg/hip NOS |
| B328. | Malignant melanoma stage IA |
| B329. | Malignant melanoma stage IB |
| B32A. | Malignant melanoma stage IIA |
| B32B. | Malignant melanoma stage IIB |
| B32C. | Malignant melanoma stage IIC |
| B32D. | Malignant melanoma stage IIIA |
| B32E. | Malignant melanoma stage IIIB |
| B32F. | Malignant melanoma stage IIIC |
| B32G. | Malignant melanoma stge IV M1a |
| B32H. | Malignant melanoma stge IV M1b |
| B32J. | Malignant melanoma stge IV M1c |
| B32y. | Malig melan oth spec skin site |
| B32y0 | Overlap malig melanoma of skin |
| B32z. | Malignant melanoma of skin NOS |
| B34.. | Malig neop female breast (f) |
| B340. | Malig neop nipple/areola (f) |
| B3400 | Malig neop nipple of breast(f) |
| B3401 | Malig neop areola of breast(f) |
| B340z | Malig neop nippl/areol (f) NOS |
| B341. | Malig neop cent part breast(f) |
| B342. | Malig neop upp-inner quad (f) |
| B343. | Malig neop low-inner quad (f) |
| B344. | Malig neop upp-outer quad (f) |
| B345. | Malig neop low-outer quad (f) |
| B346. | Malig neop axillary tail (f) |
| B347. | Mal neopl, overlapping breast |
| B34y. | Malig neop oth site breast (f) |
| B34y0 | Malig neop ectopic breast (f) |
| B34yz | Malig neop oth breast (f) NOS |
| B34z. | Malig neop female breast NOS |
| B35.. | Malig neop male breast (m) |
| B350. | Malig neop nipple/areola (m) |
| B3500 | Malig neop nipple male breast |
| B3501 | Malig neop areola male breast |
| B350z | Malig neop nippl/areol (m) NOS |
| B35z. | Malig neop oth site breast (m) |
| B35z0 | Malig neop ectopic breast (m) |
| B35zz | Malig neop male breast NOS |
| B36.. | Loc recur malign tumour breast |
| B3y.. | Malig neop bone/skin/breas OS |
| B3z.. | Malig neop bone/skin/breas NOS |
| B4... | Malig neop genitourinary organ |
| B40.. | Malig neop uterus, part unspec |
| B41.. | Malig neop cervix uteri |
| B410. | Malig neop endocervix |
| B4100 | Malig neop endocervical canal |
| B4101 | Malig neop endocervical gland |
| B410z | Malig neop endocervix NOS |
| B411. | Malig neop exocervix |
| B412. | Mal neop, overlap lesn cerv ut |
| B41y. | Malig neop other site cervix |
| B41y0 | Malig neop cervical stump |
| B41y1 | Malig neop squamocolum cervix |
| B41yz | Malig neop oth site cervix NOS |
| B41z. | Malig neop cervix uteri NOS |
| B42.. | Malig neop placenta |
| B420. | Choriocarcinoma |
| B43.. | Malig neop body of uterus |
| B430. | Malig neop corp uteri,ex isthm |
| B4300 | Malig neop cornu corpus uteri |
| B4301 | Malig neop fundus corpus uteri |
| B4302 | Malig neop endomet corpus uter |
| B4303 | Malig neop myomet corpus uteri |
| B430z | Malig neop corpus uteri NOS |
| B431. | Malig neop isthmus of uterus |
| B4310 | Malig neop low uterine segment |
| B431z | Malig neop isthmus uterus NOS |
| B432. | Mal neop, ovlap les corp uteri |
| B43y. | Malig neop other uterine body |
| B43z. | Malig neop body of uterus NOS |
| B44.. | Malig neop ovary/uterine adnex |
| B440. | Malig neop ovary |
| B441. | Malig neop fallopian tube |
| B442. | Malig neop broad ligament |
| B443. | Malig neop parametrium |
| B444. | Malig neop round ligament |
| B44y. | Malig neop oth uterine adenexa |
| B44z. | Malig neop uterine adnexa NOS |
| B45.. | Malig neop oth female genital |
| B450. | Malig neop vagina |
| B4500 | Malig neop Gartner's duct |
| B4501 | Malig neop vaginal vault |
| B450z | Malig neop vagina NOS |
| B451. | Malig neop labia majora |
| B4510 | Malig neop Bartholin's gland |
| B451z | Malig neop labia majora NOS |
| B452. | Malig neop labia minora |
| B453. | Malig neop clitoris |
| B454. | Malig neop vulva unspecified |
| B45X. | Mal neo/olap les/fm gen org |
| B45y. | Malig neop oth spec fem organ |
| B45y0 | Malig neopl overlap lesn vulva |
| B45z. | Malig neop female genital NOS |
| B46.. | Malig neop prostate |
| B47.. | Malig neop testis |
| B470. | Malig neop undescended testis |
| B4700 | Malig neop ectopic testis |
| B4701 | Malig neop retained testis |
| B4702 | Seminoma of undescended testis |
| B4703 | Teratoma of undescended testis |
| B470z | Malig neop undesc testis NOS |
| B471. | Mal neoplasm of descend testis |
| B4710 | Seminoma of descended testis |
| B4711 | Teratoma of descended testis |
| B471z | Mal neopl descended testis NOS |
| B47z. | Malig neop testis NOS |
| B48.. | Malig neop penis/oth male gen |
| B480. | Malig neop prepuce (foreskin) |
| B481. | Malig neop glans penis |
| B482. | Malig neop body of penis |
| B483. | Malig neop penis part unspecif |
| B484. | Malig neop epididymis |
| B485. | Malig neop spermatic cord |
| B486. | Malig neop scrotum |
| B487. | Malig neop, overlap lesn penis |
| B48y. | Malig neop oth male gen organ |
| B48y0 | Malig neop seminal vesicle |
| B48y1 | Malig neop tunica vaginalis |
| B48y2 | Mal neop,ovlap les mal gen org |
| B48yz | Malig neop oth male genit NOS |
| B48z. | Malig neop male genital NOS |
| B49.. | Malig neop of bladder |
| B490. | Malig neop trigone of bladder |
| B491. | Malig neop dome of bladder |
| B492. | Malig neop lat wall bladder |
| B493. | Malig neop ant wall bladder |
| B494. | Malig neop post wall bladder |
| B495. | Malig neop bladder neck |
| B496. | Malig neop ureteric orifice |
| B497. | Malig neop urachus |
| B498. | Loc recur malig tumour bladder |
| B49y. | Malig neop other bladder site |
| B49y0 | Mal neopl, overlap lesn bladd |
| B49z. | Malig neop bladder NOS |
| B4A.. | Malig neop kid/oth urinary org |
| B4A0. | Malig neop kidney parenchyma |
| B4A00 | Hypernephroma |
| B4A1. | Malig neop renal pelvis |
| B4A10 | Malig neop renal calyces |
| B4A11 | Malig neop ureteropelvic junct |
| B4A1z | Malig neop renal pelvis NOS |
| B4A2. | Malig neop ureter |
| B4A3. | Malig neop urethra |
| B4A4. | Malig neop paraurethral glands |
| B4Ay. | Malig neop other urinary organ |
| B4Ay0 | Mal neop overlap les urin orgs |
| B4Az. | Malig neop urinary organ NOS |
| B4y.. | Malig neop genitourinary OS |
| B4z.. | Malig neop genitourinary NOS |
| B5... | Malig neop other/unspec sites |
| B50.. | Malig neop eye |
| B500. | Malig neop eyeball ex spec pt |
| B5000 | Malig neop ciliary body |
| B5001 | Malig neop iris |
| B5002 | Malig neop crystalline lens |
| B5003 | Malig neop sclera |
| B500z | Malig neop eyeball NOS |
| B501. | Malig neop orbit |
| B5010 | Malig neop connect tiss orbit |
| B5011 | Malig neop extraoc musc orbit |
| B501z | Malig neop orbit NOS |
| B502. | Malig neop lacrimal gland |
| B503. | Malig neop conjunctiva |
| B504. | Malig neop cornea |
| B505. | Malig neop retina |
| B506. | Malig neop choroid |
| B507. | Malig neop lacrimal duct |
| B5070 | Malig neop lacrimal sac |
| B5071 | Malig neop nasolacrimal duct |
| B507z | Malig neop lacrimal duct NOS |
| B508. | Mal neop, overlap lesn eye/adn |
| B509. | Malignant melanoma of eye |
| B50y. | Malig neop oth spec site eye |
| B50z. | Malig neop eye NOS |
| B51.. | Malig neop brain |
| B510. | Malig neop cereb ex lobe/vent |
| B5100 | Malig neop basal ganglia |
| B5101 | Malig neop cerebral cortex |
| B5102 | Malig neop corpus striatum |
| B5103 | Malig neop globus pallidus |
| B5104 | Malig neop hypothalamus |
| B5105 | Malig neop thalamus |
| B510z | Malig neop cerebrum NOS |
| B511. | Malig neop frontal lobe |
| B512. | Malig neop temporal lobe |
| B5120 | Malig neop hippocampus |
| B5121 | Malig neop uncus |
| B512z | Malig neop temporal lobe NOS |
| B513. | Malig neop parietal lobe |
| B514. | Malig neop occipital lobe |
| B515. | Malig neop ventricles |
| B5150 | Malig neop choroid plexus |
| B5151 | Malig neop floor of ventricle |
| B515z | Malig neop ventricles NOS |
| B516. | Malig neop cerebellum |
| B517. | Malig neop brain stem |
| B5170 | Malig neop cerebral peduncle |
| B5171 | Malig neop medulla oblongata |
| B5172 | Malig neop midbrain |
| B5173 | Malig neop pons |
| B517z | Malig neop brain stem NOS |
| B51y. | Malig neop oth parts of brain |
| B51y0 | Malig neop corpus callosum |
| B51y1 | Malig neop tapetum |
| B51y2 | Malig neop overl lesion brain |
| B51yz | Malig neop oth part brain NOS |
| B51z. | Malig neop brain NOS |
| B52.. | Malig neop oth nervous system |
| B520. | Malig neop cranial nerves |
| B5200 | Malig neop olfactory bulb |
| B5201 | Malig neoplasm of optic nerve |
| B5202 | Malig neopl of acoustic nerve |
| B520z | Malig neop cranial nerves NOS |
| B521. | Malig neop cerebral meninges |
| B5210 | Malig neop cerebral dura mater |
| B5211 | Malig neop cereb arach mater |
| B5212 | Malig neop cerebral pia mater |
| B521z | Malig neop cereb meninges NOS |
| B522. | Malig neop spinal cord |
| B523. | Malig neop spinal meninges |
| B5230 | Malig neop spinal dura mater |
| B5231 | Malig neop spinal arach mater |
| B5232 | Malig neop spinal pia mater |
| B523z | Malig neop spinal meninges NOS |
| B524. | Mal neop per nrv/auton nrv sys |
| B5240 | Mal neo per nrv head/face/neck |
| B5241 | Mal neo per nv up limb, inc sh |
| B5242 | Mal neo per nv low lmb,inc hip |
| B5243 | Malig neopl periph nerv thorax |
| B5244 | Malig neopl periph nerve abdom |
| B5245 | Malig neopl periph nerv pelvis |
| B5246 | Ml neo,ovlap les per nv/aut ns |
| B524W | Mal neo/p nrv+auto n s,unsp |
| B524X | Mal neo/periph nrv/trnk,uns |
| B525. | Malig neoplasm of cauda equina |
| B52W. | Mal neo o'lap les br/ot CNS |
| B52X. | Mal neop meninges, unspec |
| B52y. | Malig neop oth spec nerv syst |
| B52z. | Malig neop nervous system NOS |
| B53.. | Malig neop thyroid gland |
| B54.. | Malig neop oth endocrine gland |
| B540. | Malig neop adrenal gland |
| B5400 | Malig neop adrenal cortex |
| B5401 | Malig neop adrenal medulla |
| B540z | Malig neop adrenal gland NOS |
| B541. | Malig neop parathyroid gland |
| B542. | Malig neop pit+craniophar duct |
| B5420 | Malig neop pituitary gland |
| B5421 | Malig neop craniophary duct |
| B542z | Malig neop pituitary gland NOS |
| B543. | Malig neop pineal gland |
| B544. | Malig neop carotid body |
| B545. | Malig neop aort body/paragangl |
| B5450 | Malig neop glomus jugulare |
| B5451 | Malig neop aortic body |
| B5452 | Malig neop coccygeal body |
| B545z | Malig neop aort body/parag NOS |
| B546. | Neuroblastoma |
| B54X. | Mal neo-plurigland inv,unsp |
| B54y. | Malig neop oth spec endo gland |
| B54z. | Malig neop endocrine gland NOS |
| B55.. | Malig neop oth,ill-define site |
| B550. | Malig neop head, neck and face |
| B5500 | Malig neop head NOS |
| B5501 | Malig neop cheek NOS |
| B5502 | Malig neop nose NOS |
| B5503 | Malig neop jaw NOS |
| B5504 | Malig neop neck NOS |
| B5505 | Malig neop supraclav fossa NOS |
| B550z | Malig neop head,neck,face NOS |
| B551. | Malig neop thorax |
| B5510 | Malig neop axilla NOS |
| B5511 | Malig neop chest wall NOS |
| B5512 | Malig neop intrathor site NOS |
| B551z | Malig neop thorax NOS |
| B552. | Malig neop abdomen |
| B553. | Malig neop pelvis |
| B5530 | Malig neop inguinal region NOS |
| B5531 | Malig neop presacral region |
| B5532 | Malig neop sacrococcygeal reg |
| B553z | Malig neop pelvis NOS |
| B554. | Malig neop upper limb NOS |
| B555. | Malig neop lower limb NOS |
| B55y. | Malig neop other specif site |
| B55y0 | Malig neop back NOS |
| B55y1 | Malig neop trunk NOS |
| B55y2 | Malig neop flank NOS |
| B55yz | Malig neop specified site NOS |
| B55z. | Malig neop ill define site NOS |
| B56.. | Secondar/malig neop lymph node |
| B560. | 2-malig neop LN head/face/neck |
| B5600 | 2-malig neop sup parotid LN |
| B5601 | 2-malig neop mastoid LN |
| B5602 | 2-malig neop sup cervical LN |
| B5603 | 2-malig neop occipital LN |
| B5604 | 2-malig neop deep parotid LN |
| B5605 | 2-malig neop submandib LN |
| B5606 | 2-malig neop facial LN |
| B5607 | 2-malig neop submental LN |
| B5608 | 2-malig neop ant cervical LN |
| B5609 | 2-malig neop deep cervic LN |
| B560z | 2-malig neop LN head/face NOS |
| B561. | 2-malig neop intrathoracic LN |
| B5610 | 2-malig neop intern mammary LN |
| B5611 | 2-malig neop intercostal LN |
| B5612 | 2-malig neop diaphragmatic LN |
| B5613 | 2-malig neop ant mediastin LN |
| B5614 | 2-malig neop post mediastin LN |
| B5615 | 2-malig neop paratracheal LN |
| B5616 | 2-malig neop sup tracheobro LN |
| B5617 | 2-malig neop inf tracheobro LN |
| B5618 | 2-malig neop bronchopulmon LN |
| B5619 | 2-malig neop pulmonary LN |
| B561z | 2-malig neop intrathor LN NOS |
| B562. | 2-malig neop intra-abdo LN |
| B5620 | 2-malig neop coeliac LN |
| B5621 | 2-malig neop sup mesenteric LN |
| B5622 | 2-malig neop inf mesenteric LN |
| B5623 | 2-malig neop common iliac LN |
| B5624 | 2-malig neop external iliac LN |
| B562z | 2-malig neop intra-abdo LN NOS |
| B563. | 2-malig neop axilla/arm LN |
| B5630 | 2-malig neop axillary LN |
| B5631 | 2-malig neop supratrochlear LN |
| B5632 | 2-malig neop infraclavic LN |
| B5633 | 2-malig neop pectoral LN |
| B563z | 2-malig neop axilla/arm LN NOS |
| B564. | 2-malig neop inguinal/leg LN |
| B5640 | 2-malig neop super inguinal LN |
| B5641 | 2-malig neop deep inguinal LN |
| B5642 | 2-malig neop popliteal LN |
| B564z | 2-malig neop inguin/leg LN NOS |
| B565. | 2-malig neop intrapelvic LN |
| B5650 | 2-malig neop internal iliac LN |
| B5651 | 2-malig neop inf epigastric LN |
| B5652 | 2-malig neop circumfl iliac LN |
| B5653 | 2-malig neop sacral LN |
| B5654 | 2-malig neop obturator LN |
| B565z | 2-malig neop intrapelv LN NOS |
| B56y. | 2-malig neop multiple site LN |
| B56z. | 2-malig neop lymph nodes NOS |
| B57.. | 2-malig neop resp/digest syst |
| B570. | 2-malig neop lung |
| B571. | 2-malig neop mediastinum |
| B572. | 2-malig neop pleura |
| B573. | 2-malig neop other resp organs |
| B574. | 2-malig neop small intest/duod |
| B5740 | 2-malig neop duodenum |
| B5741 | 2-malig neop jejunum |
| B5742 | 2-malig neop ileum |
| B574z | 2-malig neop small intest NOS |
| B575. | 2-malig neop colon/rectum |
| B5750 | 2-malig neop colon |
| B5751 | 2-malig neop rectum |
| B575z | 2-malig neop colon/rect NOS |
| B576. | 2-malig neop retroperit/perit |
| B5760 | 2-malig neop retroperitoneum |
| B5761 | 2-malig neop peritoneum |
| B5762 | Malignant ascites |
| B576z | 2-malig neop retrop/perit NOS |
| B577. | 2-malig neop liver |
| B5770 | Sec ma ne liv intrahep bile du |
| B57y. | 2-malig neop other GIT organs |
| B57z. | 2-malig neop resp/dig sys NOS |
| B58.. | 2-malig neop other spec sites |
| B580. | 2-malig neop kidney |
| B581. | 2-malig neop oth urinary organ |
| B5810 | 2-malig neop ureter |
| B5811 | 2-malig neop bladder |
| B5812 | 2-malig neop urethra |
| B581z | 2-malig neop oth urin org NOS |
| B582. | 2-malig neop skin |
| B5820 | 2-malig neop skin head |
| B5821 | 2-malig neop skin of face |
| B5822 | 2-malig neop skin of neck |
| B5823 | 2-malig neop skin of trunk |
| B5824 | 2-malig neop skin shoulder/arm |
| B5825 | 2-malig neop skin of hip/leg |
| B5826 | 2-malig neop skin of breast |
| B582z | 2-malig neop skin NOS |
| B583. | 2-malig neop brain/spinal cord |
| B5830 | 2-malig neop brain |
| B5831 | 2-malig neop spinal cord |
| B5832 | Cerebral metastasis |
| B583z | 2-malig neop brain/spine NOS |
| B584. | 2-malig neop oth nervous syst |
| B585. | 2-malig neop bone/bone marrow |
| B5850 | Patholog # metastic bone dis |
| B586. | 2-malig neop ovary |
| B587. | 2-malig neop adrenal gland |
| B58y. | 2-malig neop oth specif sites |
| B58y0 | 2-malig neop breast |
| B58y1 | 2-malig neop uterus |
| B58y2 | 2-malig neop cervix uteri |
| B58y3 | 2-malig neop vagina |
| B58y4 | 2-malig neop vulva |
| B58y5 | 2-malig neop prostate |
| B58y6 | 2-malig neop testis |
| B58y7 | 2-malig neop penis |
| B58y8 | 2-malig neop epididy/vas defer |
| B58y9 | 2-malig neop of tongue |
| B58yz | 2-malig neop oth spec site NOS |
| B58z. | 2-malig neop oth spec site NOS |
| B59.. | Malig neop of unspec site |
| B590. | Disseminated malignancy NOS |
| B591. | Other malig neop NOS |
| B592. | Mal neo indep (prim) mult site |
| B592X | Kaposi sarcoma/multi organs |
| B593. | Primary malig neo unknown site |
| B594. | Second malig neo unknown site |
| B595. | Malign tumour unknown origin |
| B59z. | Malig neop of unspec site NOS |
| B59zX | Kaposi's sarcoma,unspecifd |
| B5y.. | Malig neop oth and unspec OS |
| B5z.. | Malig neop oth and unspec NOS |
| B6... | Malig neop lympha/haemo tiss |
| B60.. | Lymphosarcoma/reticulosarcoma |
| B600. | Reticulosarcoma |
| B6000 | Reticulosarcoma - unspec site |
| B6001 | Reticulosarcoma-head/face/neck |
| B6002 | Reticulosarcoma -intrathoracic |
| B6003 | Reticulosarcoma - intra-abdomi |
| B6004 | Reticulosarcoma - axilla / arm |
| B6005 | Reticulosarcoma - inguinal/leg |
| B6006 | Reticulosarcoma - intrapelvic |
| B6007 | Reticulosarcoma - spleen |
| B6008 | Reticulosarcoma-multiple sites |
| B600z | Reticulosarcoma NOS |
| B601. | Lymphosarcoma |
| B6010 | Lymphosarcoma - unspecif. site |
| B6011 | Lymphosarcoma - head/face/neck |
| B6012 | Lymphosarcoma - intrathoracic |
| B6013 | Lymphosarcoma-intra-abdominal |
| B6014 | Lymphosarcoma - axilla or arm |
| B6015 | Lymphosarcoma - inguinal / leg |
| B6016 | Lymphosarcoma - intrapelvic |
| B6017 | Lymphosarcoma - spleen |
| B6018 | Lymphosarcoma - multiple sites |
| B601z | Lymphosarcoma NOS |
| B602. | Burkitt's lymphoma |
| B6020 | Burkitt's lymph - unspec. site |
| B6021 | Burkitt's lymph-head/face/neck |
| B6022 | Burkitt's lymph-intrathoracic |
| B6023 | Burkitt's lymph-intra-abdomen |
| B6024 | Burkitt's lymphoma-axilla/arm |
| B6025 | Burkitt's lymph-inguinal/leg |
| B6026 | Burkitt's lymphoma-intrapelvic |
| B6027 | Burkitt's lymphoma - spleen |
| B6028 | Burkitt's lymphoma-multip.site |
| B602z | Burkitt's lymphoma NOS |
| B60y. | Other reticulo / lymphosarcoma |
| B60z. | Reticulo/lymphosarcoma NOS |
| B61.. | Hodgkin's disease |
| B610. | Hodgkin's paragranuloma |
| B6100 | Hodgkin's paragran-unspec site |
| B6101 | Hodgkin's paragran - head/neck |
| B6102 | Hodgkin's paragran-intrathorax |
| B6103 | Hodgkin's paragran-intra-abd. |
| B6104 | Hodgkin's paragran-axilla/arm |
| B6105 | Hodgkin's paragran-inguin./leg |
| B6106 | Hodgkin's paragran-intrapelvic |
| B6107 | Hodgkin's paragranuloma-spleen |
| B6108 | Hodgkin's paragran.-mult.sites |
| B610z | Hodgkin's paragranuloma NOS |
| B611. | Hodgkin's granuloma |
| B6110 | Hodgkin's granuloma-unsp. site |
| B6111 | Hodgkin's granuloma-head/neck |
| B6112 | Hodgkin's granuloma-intrathor. |
| B6113 | Hodgkin's granuloma - intra-ab |
| B6114 | Hodgkin's granuloma-axilla/arm |
| B6115 | Hodgkin's granuloma-inguin/leg |
| B6116 | Hodgkin's granuloma-intrapelv. |
| B6117 | Hodgkin's granuloma - spleen |
| B6118 | Hodgkin's granuloma-mult.sites |
| B611z | Hodgkin's granuloma NOS |
| B612. | Hodgkin's sarcoma |
| B6120 | Hodgkin's sarcoma-unspec. site |
| B6121 | Hodgkin's sarcoma - head/neck |
| B6122 | Hodgkin's sarcoma-intrathorax |
| B6123 | Hodgkin's sarcoma-intra-abdom. |
| B6124 | Hodgkin's sarcoma - axilla/arm |
| B6125 | Hodgkin's sarcoma-inguinal/leg |
| B6126 | Hodgkin's sarcoma-intrapelvic |
| B6127 | Hodgkin's sarcoma - spleen |
| B6128 | Hodgkin's sarcoma-multip.sites |
| B612z | Hodgkin's sarcoma NOS |
| B613. | Hodgkin's lympho-histio predom |
| B6130 | Lymph-histio-Hodgkin,unsp.site |
| B6131 | Lymph-histio-Hodgkin,head/neck |
| B6132 | Lymph-histio-Hodgkin,intrathor |
| B6133 | Lymph-histio-Hodgkin,intra-abd |
| B6134 | Lymph-histio-Hodgkin,axil./arm |
| B6135 | Lymph-histio-Hodgkin,ing./leg |
| B6136 | Lymph-histio-Hodgkin,intrapelv |
| B6137 | Lymph-histio-Hodgkin, spleen |
| B6138 | Lymph-histio-Hodgkin,mult.site |
| B613z | Lympho-histiocytic Hodgkin NOS |
| B614. | Hodgkin's nodular sclerosis |
| B6140 | Hodgkin's nod.scl. unspec site |
| B6141 | Hodgkin's nod.scl.-head/neck |
| B6142 | Hodgkin's nod.scl.-intrathorax |
| B6143 | Hodgkin's nod.scl.-intra-abdom |
| B6144 | Hodgkin's nod.scl.-axilla/arm |
| B6145 | Hodgkin's nod.scl.- inguin/leg |
| B6146 | Hodgkin's nod.scl.-intrapelvic |
| B6147 | Hodgkin's nod.scl. - spleen |
| B6148 | Hodgkin's nod.scl.-multip.site |
| B614z | Hodgkin's nodular scler. NOS |
| B615. | Hodgkin's - mixed cellularity |
| B6150 | Hodgkin's mix cell-unspec site |
| B6151 | Hodgkin's mix cell-head/neck |
| B6152 | Hodgkin's mix cell-intrathorax |
| B6153 | Hodgkin's mix cell-intra-abdom |
| B6154 | Hodgkin's mix cell-axilla/arm |
| B6155 | Hodgkin's mix cell-inguin/leg |
| B6156 | Hodgkin's mix cell-intrapelvic |
| B6157 | Hodgkin's mix cell - spleen |
| B6158 | Hodgkin's mix cell-multip.site |
| B615z | Hodgkin's mixed cellular. NOS |
| B616. | Hodgkin's lymphocyt. depletion |
| B6160 | Hodgkin's lymp.dep.-unsp. site |
| B6161 | Hodgkin's lymp.dep.-head/neck |
| B6162 | Hodgkin's lymp.dep.-intrathor. |
| B6163 | Hodgkin's lymp.dep.-intra-abd. |
| B6164 | Hodgkin's lymp.dep.-axilla/arm |
| B6165 | Hodgkin's lymp.dep.-inguin/leg |
| B6166 | Hodgkin's lymp.dep.-intrapelv. |
| B6167 | Hodgkin's lymp.dep. - spleen |
| B6168 | Hodgkin's lymp.dep.-mult.sites |
| B616z | Hodgkin's lymphocyt. depl. NOS |
| B617. | Nod lympho predom Hodgk lymph |
| B618. | Nod scler classic Hodgk lymph |
| B619. | Mixed cellul class hodgk lymph |
| B61A. | Lymphoc depl class Hodgk lymph |
| B61B. | Lymphoc-rich class Hodgk lymph |
| B61C. | Oth classical Hodgkin lymphoma |
| B61z. | Hodgkin's disease NOS |
| B61z0 | Hodgkin's dis.NOS-unspec. site |
| B61z1 | Hodgkin's dis.NOS-head/neck |
| B61z2 | Hodgkin's dis.NOS-intrathorax |
| B61z3 | Hodgkin's dis.NOS-intra-abdom. |
| B61z4 | Hodgkin's dis.NOS-axilla/arm |
| B61z5 | Hodgkin's dis.NOS-inguinal/leg |
| B61z6 | Hodgkin's dis.NOS-intrapelvic |
| B61z7 | Hodgkin's dis.NOS - spleen |
| B61z8 | Hodgkin's dis.NOS-multip.sites |
| B61zz | Hodgkin's disease NOS |
| B62.. | Other malig neop lymph/histioc |
| B620. | Nodular lymphoma (Brill-Symm.) |
| B6200 | Nodular lymphoma-unspec. site |
| B6201 | Nodular lymphoma-head/neck |
| B6202 | Nodular lymphoma-intrathoracic |
| B6203 | Nodular lymphoma-intra-abdomen |
| B6204 | Nodular lymphoma-axilla/arm |
| B6205 | Nodular lymphoma-inguinal/leg |
| B6206 | Nodular lymphoma-intrapelvic |
| B6207 | Nodular lymphoma - spleen |
| B6208 | Nodular lymphoma-multiple site |
| B620z | Nodular lymphoma NOS |
| B621. | Mycosis fungoides |
| B6210 | Mycosis fungoides-unspec. site |
| B6211 | Mycosis fungoides-head/neck |
| B6212 | Mycosis fungoides-intrathorax |
| B6213 | Mycosis fungoides-intra-abdom. |
| B6214 | Mycosis fungoides-axilla/arm |
| B6215 | Mycosis fungoides-inguinal/leg |
| B6216 | Mycosis fungoides-intrapelvic |
| B6217 | Mycosis fungoides - spleen |
| B6218 | Mycosis fungoides-multip.sites |
| B621z | Mycosis fungoides NOS |
| B622. | Sezary's disease |
| B6220 | Sezary's disease-unspec. site |
| B6221 | Sezary's disease-head/neck |
| B6222 | Sezary's disease-intrathoracic |
| B6223 | Sezary's disease-intra-abdomen |
| B6224 | Sezary's disease-axilla/arm |
| B6225 | Sezary's disease-inguinal/leg |
| B6226 | Sezary's disease - intrapelvic |
| B6227 | Sezary's disease - spleen |
| B6228 | Sezary's disease-multiple site |
| B622z | Sezary's disease NOS |
| B623. | Malignant histiocytosis |
| B6230 | Malig.histiocytosis-unsp.site |
| B6231 | Malig.histiocytosis-head/neck |
| B6232 | Malig.histiocytosis-intrathor. |
| B6233 | Malig.histiocytosis-intra-abd. |
| B6234 | Malig.histiocytosis-axilla/arm |
| B6235 | Malig.histiocytosis-inguin/leg |
| B6236 | Malig.histiocytosis-intrapelv. |
| B6237 | Malig.histiocytosis - spleen |
| B6238 | Malig.histiocytosis-mult.sites |
| B623z | Malignant histiocytosis NOS |
| B624. | Leukaemic reticuloendothelios. |
| B6240 | Leukaem.reticuloend.-unsp.site |
| B6241 | Leukaem.reticuloend.-head/neck |
| B6242 | Leukaem.reticuloend.-intrathor |
| B6243 | Leukaem.reticuloend.-intra-abd |
| B6244 | Leukaem.reticuloend.-axill/arm |
| B6245 | Leukaem.reticuloend.-ingu./leg |
| B6246 | Leukaem.reticuloend.-intrapelv |
| B6247 | Leukaem.reticuloend. - spleen |
| B6248 | Leukaem.reticuloend.-mult.site |
| B624z | Leukaemic reticuloendoth. NOS |
| B625. | Letterer-Siwe disease |
| B6250 | Letterer-Siwe dis.-unspec.site |
| B6251 | Letterer-Siwe dis.-head/neck |
| B6252 | Letterer-Siwe dis.-intrathorax |
| B6253 | Letterer-Siwe dis.-intra-abdom |
| B6254 | Letterer-Siwe dis.-axilla/arm |
| B6255 | Letterer-Siwe dis.-inguin./leg |
| B6256 | Letterer-Siwe dis.-intrapelvic |
| B6257 | Letterer-Siwe dis. - spleen |
| B6258 | Letterer-Siwe dis.-multip.site |
| B625z | Letterer-Siwe disease NOS |
| B626. | Malignant mast cell tumours |
| B6260 | Mast cell malignancy ? site |
| B6261 | Mast cell malignancy-head/neck |
| B6262 | Mast cell malignancy-intrathor |
| B6263 | Mast cell malignancy-intra-abd |
| B6264 | Mast cell malignancy-axill/arm |
| B6265 | Mast cell malignancy-ingui/leg |
| B6266 | Mast cell malignancy-intrapelv |
| B6267 | Mast cell malignancy - spleen |
| B6268 | Mast cell malignancy-mult.site |
| B626z | Malignant mast cell tumour NOS |
| B627. | Non - Hodgkin's lymphoma |
| B6270 | Follic non-Hodgkins small cell |
| B6271 | Follic non-Hodgkins mixed cell |
| B6272 | Follic non-Hodgkins large cell |
| B6273 | Diffus non-Hodgkins small cell |
| B6274 | Diffus non-Hodg small clv cell |
| B6275 | Diffus non-Hodgkin mixed cell |
| B6276 | Diff non-Hodgkin immunoblastic |
| B6277 | Diff non-Hodgkin lymphoblastic |
| B6278 | Diffuse non-Hodgkin undiff |
| B6279 | Mucosa-associated lymphoma |
| B627A | Diff non-Hodg large cell lymph |
| B627B | Oth type foll non-Hodg lymphom |
| B627C | Follicular non-Hodgkin's lymph |
| B627D | Diff non-Hodg centro lymph |
| B627E | Diffuse large B-cell lymphoma |
| B627F | Extra ma zone B ly mu lymph ti |
| B627G | Mediast thym large B-cell lymp |
| B627W | Unsp B-cell non-Hodg lympha |
| B627X | Diff non-Hodg lympha, unsp |
| B628. | Follicular lymphoma |
| B6280 | Follicular lymphoma grade 1 |
| B6281 | Follicular lymphoma grade 2 |
| B6282 | Follicular lymphoma grade 3 |
| B6283 | Follicular lymphoma grade 3a |
| B6284 | Follicular lymphoma grade 3b |
| B6285 | Diffuse follicle cent lymphoma |
| B6286 | Cutaneous follicle cent lymph |
| B6287 | Other types follicular lymphom |
| B629. | Multif multis diss Lang-c hist |
| B62A. | Sarcoma of dendritic cells |
| B62B. | Multif unisyst Lang-cell histi |
| B62C. | Unifocal Lang-cell histiocytos |
| B62D. | Histiocytic sarcoma |
| B62E. | T/NK-cell lymphoma |
| B62E0 | Mature T/NK-cell lymphoma |
| B62E1 | Anapl large cell lymph ALK-pos |
| B62E2 | Anapl large cell lymph ALK-neg |
| B62E3 | Cutaneous T-cell lymphoma |
| B62E4 | Extranod NK/T-cell lymph na ty |
| B62E5 | Hepatosplenic T-cell lymphoma |
| B62E6 | Enterop-assoc T-cell lymphoma |
| B62E7 | Subcutaneo panniculit T lymph |
| B62E8 | Blastic NK-cell lymphoma |
| B62E9 | Angioimmunoblast T-cell lymph |
| B62EA | Prim cut CD30-pos T-cel prolif |
| B62Ew | Other mature T/NK-cell lymphom |
| B62F. | Nonfollicular lymphoma |
| B62F0 | Small cell B-cell lymphoma |
| B62F1 | Mantle cell lymphoma |
| B62F2 | Lymphoblast (diffuse) lymphoma |
| B62Fy | Other non-follicular lymphoma |
| B62x. | Mal lymphoma otherwise specif |
| B62x0 | T-zone lymphoma |
| B62x1 | Lymphoepithelioid lymphoma |
| B62x2 | Peripheral T-cell lymphoma |
| B62x3 | Malig reticuloendotheliosis |
| B62x4 | Malignant reticulosis |
| B62x5 | Mal immunoprolif sm intest dis |
| B62x6 | True histiocytic lymphoma |
| B62xX | Oth/un per/cut T-cel lympha |
| B62y. | Malignant lymphoma NOS |
| B62y0 | Malig.lymphoma NOS-unspec site |
| B62y1 | Malig.lymphoma NOS-head/neck |
| B62y2 | Malig.lymphoma NOS-intrathorax |
| B62y3 | Malig.lymphoma NOS-intra-abdom |
| B62y4 | Malig.lymphoma NOS-axilla/arm |
| B62y5 | Malig.lymphoma NOS-inguin./leg |
| B62y6 | Malig.lymphoma NOS-intrapelvic |
| B62y7 | Malig.lymphoma NOS - spleen |
| B62y8 | Malig.lymphoma NOS-multip.site |
| B62yz | Malignant lymphoma NOS |
| B62z. | Lymphoid/histiocytic malig NOS |
| B62z0 | Lymph/histio malig-unspec site |
| B62z1 | Lymph/histio malig-head/neck |
| B62z2 | Lymph/histio malig-intrathorax |
| B62z3 | Lymph/histio malig-intra-abdom |
| B62z4 | Lymph/histio malig-axilla/arm |
| B62z5 | Lymph/histio malig-inguin./leg |
| B62z6 | Lymph/histio malig-intrapelvic |
| B62z7 | Lymph/histio malig - spleen |
| B62z8 | Lymph/histio malig-multip.site |
| B62zz | Lymph/histio malignancy NOS |
| B63.. | Multiple myeloma+immunopr.neop |
| B630. | Multiple myeloma |
| B6300 | M plsma cel neo,extram p'cytom |
| B6301 | Solitary myeloma |
| B6302 | Plasmacytoma NOS |
| B6303 | Lambda light chain myeloma |
| B6304 | Solitary plasmacytoma |
| B631. | Plasma cell leukaemia |
| B63y. | Other immunoproliferative neop |
| B63z. | Immunoproliferative neopl.NOS |
| B64.. | Lymphoid leukaemia |
| B640. | Acute lymphoid leukaemia |
| B6400 | B-cell acute lymphob leukaemia |
| B641. | Chronic lymphoid leukaemia |
| B6410 | B-cell chron lymphocyt leukaem |
| B6411 | Clin stage A chron lymph leuk |
| B6412 | Clin stage B chron lymph leuk |
| B6413 | Clin stage C chron lymph leuk |
| B642. | Subacute lymphoid leukaemia |
| B64y. | Other lymphoid leukaemia |
| B64y0 | Aleukaemic lymphoid leukaemia |
| B64y1 | Prolymphocytic leukaemia |
| B64y2 | Adult T-cell leukaemia |
| B64y3 | B-cell prolymphocyt leukaemia |
| B64y4 | T-cell prolymphocyt leukaemia |
| B64y5 | Ad T-c lymph/leuk HTLV-1-assoc |
| B64yz | Other lymphoid leukaemia NOS |
| B64z. | Lymphoid leukaemia NOS |
| B65.. | Myeloid leukaemia |
| B650. | Acute myeloid leukaemia |
| B6500 | Acut myeloid leuk 11q23 abnorm |
| B6501 | Ac myel leuk multilineag dyspl |
| B651. | Chronic myeloid leukaemia |
| B6510 | Eosinophilic leukaemia |
| B6511 | Chron myeloid leuk BCR/ABL pos |
| B6512 | Neutrophilic leukaemia |
| B6513 | At chron myel leuk BCR/ABL neg |
| B651z | Chronic myeloid leukaemia NOS |
| B652. | Subacute myeloid leukaemia |
| B653. | Myeloid sarcoma |
| B6530 | Chloroma |
| B6531 | Granulocytic sarcoma |
| B653z | Myeloid sarcoma NOS |
| B654. | Acute myeloblastic leukaemia |
| B65y. | Other myeloid leukaemia |
| B65y0 | Aleukaemic myeloid leukaemia |
| B65y1 | Acute promyelocytic leukaemia |
| B65yz | Other myeloid leukaemia NOS |
| B65z. | Myeloid leukaemia NOS |
| B66.. | Monocytic leukaemia |
| B660. | Acute monocytic leukaemia |
| B661. | Chronic monocytic leukaemia |
| B662. | Subacute monocytic leukaemia |
| B663. | Acute monoblastic leukaemia |
| B66y. | Other monocytic leukaemia |
| B66y0 | Aleukaemic monocytic leukaemia |
| B66yz | Other monocytic leukaemia NOS |
| B66z. | Monocytic leukaemia NOS |
| B67.. | Other specified leukaemia |
| B670. | Acute erythraemia/erythroleuk. |
| B671. | Chronic erythraemia |
| B672. | Megakaryocytic leukaemia |
| B673. | Mast cell leukaemia |
| B674. | Acute panmyelosis |
| B675. | Acute myelofibrosis |
| B676. | Acute erythroid leukaemia |
| B67y. | Other and unspec leukaemia |
| B67y0 | Lymphosarcoma cell leukaemia |
| B67yz | Other and unspec leukaemia NOS |
| B67z. | Other specified leukaemia NOS |
| B68.. | Unspecif. cell type leukaemia |
| B680. | Acute leukaemia NOS |
| B681. | Chronic leukaemia NOS |
| B682. | Subacute leukaemia NOS |
| B68y. | Other leukaemia unsp.cell type |
| B68z. | Leukaemia NOS |
| B69.. | Myelomonocytic leukaemia |
| B690. | Acute myelomonocytic leukaemia |
| B691. | Chron myelomonocytic leukaemia |
| B692. | Subacut myelomonocytic leukaem |
| B693. | Juvenile myelomonocyt leukaem |
| B6y.. | Lymph/haemopoietic malig. OS |
| B6y0. | Myeloproliferative disorder |
| B6y1. | Myeloscler with myeoid metapls |
| B6z.. | Lymph/haemopoietic malig. NOS |
| B6z0. | Kaposi's sarc of lymph nodes |
| Byu.. | [X]Addtnl neoplsm classfn term |
| Byu0. | [X]Mal neopl/lp,oral cav+phary |
| Byu1. | [X]Mal neoplasm/digestiv organ |
| Byu10 | [X]Other sarcomas of the liver |
| Byu11 | [X]Other spcf carcinomas/liver |
| Byu12 | [X]Mal neo/intest trct/pt unsp |
| Byu13 | [X]Mal neo/il-df site in dig s |
| Byu2. | [X]Malignant neoplasm of resp |
| Byu20 | [X]Mal neop/bronchus,lung,unsp |
| Byu21 | [X]Mal neo/o les/hrt,medstn+pl |
| Byu22 | [X]Mal neo/up resp trct,p unsp |
| Byu23 | [X]Mal neo/o les/rsp+i'thor og |
| Byu24 | [X]Mal neo/il-def site/resp sy |
| Byu25 | [X]Mal neo mediastin,part uns |
| Byu3. | [X]Mal neoplasm/bone+art cartg |
| Byu30 | [X]Mal neo/o l/bone+art c/limb |
| Byu31 | [X]Mal neo/bne+art c/limb,unsp |
| Byu32 | [X]Mal neo/ovlap les/bne+art c |
| Byu33 | [X]Mal neo/bne+art cartlg,unsp |
| Byu4. | [X]Melanoma+oth mal neopl/skin |
| Byu40 | [X]Mal melanoma/o+unsp pt/face |
| Byu41 | [X]Mal melanoma/skin,unspecfd |
| Byu5. | [X]Mal neo/mesothel+soft tissu |
| Byu50 | [X]Mesothelioma of other sites |
| Byu51 | [X]Mesothelioma, unspecified |
| Byu52 | [X]Kaposi sarcoma/multi organs |
| Byu53 | [X]Kaposi's sarcoma,unspecifd |
| Byu54 | [X]Mal neo/periph nrv/trnk,uns |
| Byu55 | [X]Mal neo/o ls/p nrv+auto n s |
| Byu56 | [X]Mal neo/p nrv+auto n s,unsp |
| Byu57 | [X]Mal neoplm/peritoneum,unspc |
| Byu58 | [X]Mal neo/cn+sf tis/trnk,unsp |
| Byu59 | [X]Mal neo/con+sft tis,unspcf |
| Byu5A | [X]Mal neop overlap lesn skin |
| Byu5B | [X]Kaposi's sarc other sites |
| Byu6. | [X]Malignant neoplasm/breast |
| Byu7. | [X]Mal neoplasm/fem gen organs |
| Byu70 | [X]Mal neopl/uterin adnex,unsp |
| Byu71 | [X]Mal neo/o spcf fem gen orgn |
| Byu72 | [X]Mal neo/olap les/fm gen org |
| Byu73 | [X]Mal neo/fem gen organ,unspc |
| Byu8. | [X]Mal neoplasm/male gen organ |
| Byu80 | [X]Mal neo/o spcf male gen org |
| Byu81 | [X]Mal neo/olap l/male gen org |
| Byu82 | [X]Mal neo/male gen organ,unsp |
| Byu9. | [X]Mal neoplasm/urinary tract |
| Byu90 | [X]Mal neo/urinary organ,unsp |
| ByuA. | [X]Malignant neoplasm of eye, |
| ByuA0 | [X]Mal neo/o+unsp cranial nerv |
| ByuA1 | [X]Malignant neoplasm/CNS unsp |
| ByuA2 | [X]Mal neop meninges, unspec |
| ByuA3 | [X]Mal neo o'lap les br/ot CNS |
| ByuB. | [X]Mal neo/thyroid/o endocr gl |
| ByuB0 | [X]Mal neo-plurigland inv,unsp |
| ByuB1 | [X]Mal neo/endocrin gland,unsp |
| ByuC. | [X]Malignant neoplasm of ill- |
| ByuC0 | [X]Mal neo/oth specified sites |
| ByuC1 | [X]Mal neo/olap les/o+il-de st |
| ByuC2 | [X]2nd+unsp m neo lym n/mlt rg |
| ByuC3 | [X]2ndy mal neo/o+unsp rsp org |
| ByuC4 | [X]2ndy mal neo/o+unsp dig org |
| ByuC5 | [X]2ndy mal neo/blad/o+u ur og |
| ByuC6 | [X]2ndy mal neo/o+unsp pt/n sy |
| ByuC7 | [X]2ndry mal neo/oth spec site |
| ByuC8 | [X]Mal neo w'out specfctn/site |
| ByuD. | [X]Malignant neoplasms of lym |
| ByuD0 | [X]Other Hodgkin's disease |
| ByuD1 | [X]O type/follcl non-Hdgk lymp |
| ByuD2 | [X]O typ/difus non-Hdgkn lymph |
| ByuD3 | [X]Oth spc typ/non-Hdgkn lymph |
| ByuD4 | [X]Oth mal immunoprolfrtiv dis |
| ByuD5 | [X]Other lymphoid leukaemia |
| ByuD6 | [X]Other myeloid leukaemia |
| ByuD7 | [X]Other monocytic leukaemia |
| ByuD8 | [X]Other specified leukaemias |
| ByuD9 | [X]Oth leukaemia/unsp cell typ |
| ByuDA | [X]O spc m neo/lym,h'm rlt tis |
| ByuDB | [X]Mal neo/lym,h'm+rlt tis,uns |
| ByuDC | [X]Diff non-Hodg lympha, unsp |
| ByuDD | [X]Oth/un per/cut T-cel lympha |
| ByuDE | [X]Unsp B-cell non-Hodg lympha |
| ByuDF | [X]Non-Hod lympho, unspec type |
| ByuE. | [X]Mal neo/indep(prim)mult sit |
| ByuE0 | [X]Mal neo/indep(prim)mult sit |
| C184. | Multiple endoc neoplas type 1 |
| K01w1 | Drash syndrome |
| K1323 | Acq renal cyst + neopl change |
| 68W24 | Bwl sco (flxi) scr: Ca detectd |

**Table 5: Anaemia ICD10 codes**

| ICD10 | Description |
| --- | --- |
| D50 | Iron deficiency anaemia |
| D500 | Iron deficiency anaemia secondary to blood loss (chronic) |
| D501 | Sideropenic dysphagia |
| D508 | Other iron deficiency anaemias |
| D509 | Iron deficiency anaemia unspecified |
| D51 | Vitamin B12 deficiency anaemia |
| D510 | Vitamin B12 deficit anaemia due to intrinsic factor deficiency |
| D511 | Vit B12 def anaemia select vit B12 malabsorption with proteinuria |
| D513 | Other dietary vitamin B12 deficiency anaemia |
| D518 | Other vitamin B12 deficiency anaemias |
| D519 | Vitamin B12 deficiency anaemia unspecified |
| D52 | Folate deficiency anaemia |
| D520 | Dietary folate deficiency anaemia |
| D521 | Drug-induced folate deficiency anaemia |
| D528 | Other folate deficiency anaemias |
| D529 | Folate deficiency anaemia unspecified |
| D53 | Other nutritional anaemias |
| D530 | Protein deficiency anaemia |
| D531 | Other megaloblastic anaemias not elsewhere classified |
| D532 | Scorbutic anaemia |
| D538 | Other specified nutritional anaemias |
| D539 | Nutritional anaemia unspecified |
| D55 | Anaemia due to enzyme disorders |
| D550 | Anaemia due to glucose-6-phosphate dehydrogenase deficiency |
| D551 | Anaemia due to other disorders of glutathione metabolism |
| D552 | Anaemia due to disorders of glycolytic enzymes |
| D553 | Anaemia due to disorders of nucleotide metabolism |
| D558 | Other anaemias due to enzyme disorders |
| D559 | Anaemia due to enzyme disorder unspecified |
| D56 | Thalassaemia |
| D560 | Alpha thalassaemia |
| D561 | Beta thalassaemia |
| D562 | Delta-beta thalassaemia |
| D563 | Thalassaemia trait |
| D564 | Hereditary persistence of fetal haemoglobin [HPFH] |
| D568 | Other thalassaemias |
| D569 | Thalassaemiaunspecified |
| D570 | Sickle-cell anaemia with crisis |
| D571 | Sickle-cell anaemia without crisis |
| D572 | Double heterozygous sickling disorders |
| D573 | Sickle-cell trait |
| D578 | Other sickle-cell disorders |
| D58 | Other hereditary haemolytic anaemias |
| D580 | Hereditary spherocytosis |
| D581 | Hereditary elliptocytosis |
| D582 | Other haemoglobinopathies |
| D588 | Other specified hereditary haemolytic anaemias |
| D589 | Hereditary haemolytic anaemiaunspecified |
| D59 | Acquired haemolytic anaemia |
| D590 | Drug-induced autoimmune haemolytic anaemia |
| D591 | Other autoimmune haemolytic anaemias |
| D592 | Drug-induced nonautoimmune haemolytic anaemia |
| D593 | Haemolytic-uraemic syndrome |
| D594 | Other nonautoimmune haemolytic anaemias |
| D595 | Paroxysmal nocturnal haemoglobinuria [Marchiafava-Micheli] |
| D596 | Haemoglobinuria due to haemolysis from other external causes |
| D598 | Other acquired haemolytic anaemias |
| D599 | Acquired haemolytic anaemiaunspecified |
| D60 | Acquired pure red cell aplasia [erythroblastopenia] |
| D600 | Chronic acquired pure red cell aplasia |
| D601 | Transient acquired pure red cell aplasia |
| D608 | Other acquired pure red cell aplasias |
| D609 | Acquired pure red cell aplasiaunspecified |
| D61 | Other aplastic anaemias |
| D610 | Constitutional aplastic anaemia |
| D611 | Drug-induced aplastic anaemia |
| D612 | Aplastic anaemia due to other external agents |
| D613 | Idiopathic aplastic anaemia |
| D618 | Other specified aplastic anaemias |
| D619 | Aplastic anaemiaunspecified |
| D62 | Acute posthaemorrhagic anaemia |
| D62X | Acute posthaemorrhagic anaemia |
| D63 | Anaemia in chronic diseases classified elsewhere |
| D638 | Anaemia in other chronic diseases classified elsewhere |
| D64 | Other anaemias |
| D640 | Hereditary sideroblastic anaemia |
| D641 | Secondary sideroblastic anaemia due to disease |
| D642 | Secondary sideroblastic anaemia due to drugs and toxins |
| D643 | Other sideroblastic anaemias |
| D648 | Other specified anaemias |
| D649 | Anaemia unspecified |
| D70 | Agranulocytosis |
| O990 | Anaemia comp pregnancy childbirth and the puerperium |

**Table 6: Anaemia READ codes**

| READ | Description |
| --- | --- |
| 13A1. | Vegetarian diet - no meat |
| 13A2. | Vegan diet - no dairy produce |
| 1F7.. | Restricted diet pattern |
| 1F71. | Vegan / strict vegetarian |
| 1F72. | Ovo-lacto-vegetarian |
| 1F7Z. | Dietary restriction NOS |
| ByuHC | [X]Refractory anaemia, unspecified |
| D0... | Deficiency anaemias |
| D00.. | Iron deficiency anaemias |
| D000. | Iron deficiency anaemia due to chronic blood loss |
| D001. | Iron deficiency anaemia due to dietary causes |
| D00y. | Other specified iron deficiency anaemia |
| D00y0 | Sideropenic dysphagia |
| D00y1 | Microcytic hypochromic anaemia |
| D00yz | Other specified iron deficiency anaemia NOS |
| D00z. | Unspecified iron deficiency anaemia |
| D00z2 | Idiopathic hypochromic anaemia |
| D00zz | Iron deficiency anaemia NOS |
| D01.. | Other deficiency anaemias |
| D010. | Pernicious anaemia |
| D011. | Other vitamin B12 deficiency anaemias |
| D0110 | Vitamin B12 deficiency anaemia due to dietary causes |
| D0111 | Vit B12 defic anaemia due to malabsorption with proteinuria |
| D011X | Vitamin B12 deficiency anaemia, unspecified |
| D011z | Other vitamin B12 deficiency anaemia NOS |
| D012. | Folate-deficiency anaemia |
| D0121 | Folate-deficiency anaemia due to dietary causes |
| D0122 | Folate-deficiency anaemia, drug induced |
| D0123 | Folate-deficiency anaemia due to malabsorption |
| D0124 | Folate-deficiency anaemia due to liver disorders |
| D0125 | Macrocytic anaemia unspecified cause |
| D012z | Folate-deficiency anaemia NOS |
| D013. | Other specified megaloblastic anaemia NEC |
| D0130 | Combined B12 and folate deficiency anaemia |
| D013z | Other specified megaloblastic anaemia NEC NOS |
| D014. | Protein-deficiency anaemia |
| D0140 | Amino-acid deficiency anaemia |
| D014z | Protein-deficiency anaemia NOS |
| D01y. | Other specified nutritional deficiency anaemia |
| D01yy | Other specified other nutritional deficiency anaemia |
| D01yz | Other specified nutritional deficiency anaemia NOS |
| D01z. | Other deficiency anaemias NOS |
| D01z0 | [X]Megaloblastic anaemia NOS |
| D0y.. | Other specified deficiency anaemias |
| D0z.. | Deficiency anaemias NOS |
| D1... | Haemolytic anaemias |
| D10.. | Hereditary haemolytic anaemias |
| D100. | Hereditary spherocytosis |
| D101. | Hereditary elliptocytosis |
| D102. | Haemolytic anaemia due to glutathione metabolism disorder |
| D1020 | Glucose-6-phosphate dehydrogenase deficiency anaemia |
| D1021 | Favism |
| D1022 | Drug-induced enzyme deficiency anaemia |
| D102y | Haemolytic anaemia due to glutathione metabolism disorder OS |
| D102z | Haemolytic anaemia due glutathione metabolism disorder NOS |
| D103. | Other enzyme deficiency haemolytic anaemia |
| D1031 | Haemolytic anaemia due to pyruvate kinase deficiency |
| D1033 | Anaemia due to disorders of nucleotide metabolism |
| D103z | Enzyme deficiency haemolytic anaemia NOS |
| D104. | Thalassaemia |
| D1040 | Thalassaemia major NEC |
| D1041 | Thalassaemia minor NEC |
| D1042 | Thalassaemia with haemoglobin S disease |
| D1043 | Alpha thalassaemia |
| D1044 | Alpha trait thalassaemia |
| D1045 | Beta trait thalassaemia |
| D1046 | Beta intermedia thalassaemia |
| D1047 | Beta major thalassaemia |
| D1048 | Beta minor thalassaemia |
| D1049 | Delta-beta thalassaemia |
| D104A | Hereditary leptocytosis |
| D104z | Thalassaemia NOS |
| D105. | Sickle-cell trait |
| D106. | Sickle-cell anaemia |
| D1060 | Sickle-cell anaemia of unspecified type |
| D1061 | Sickle-cell anaemia with no crisis |
| D1062 | Sickle-cell anaemia with crisis |
| D1063 | Sickle-cell anaemia with haemoglobin C disease |
| D1064 | Sickle-cell anaemia with haemoglobin D disease |
| D1065 | Sickle-cell anaemia with haemoglobin E disease |
| D106z | Sickle-cell anaemia NOS |
| D107. | Other haemoglobinopathies |
| D1070 | Congenital Heinz-body anaemia |
| D1071 | Hereditary persistence of fetal haemoglobin [HPFH] |
| D1072 | Haemoglobin Bart's disease |
| D1073 | Haemoglobin-C disease |
| D1074 | Haemoglobin-D disease |
| D1075 | Haemoglobin-E disease |
| D1076 | Haemoglobin Zurich disease |
| D1077 | Haemoglobin-H disease |
| D1078 | Haemoglobin C trait |
| D1079 | Haemoglobin D trait |
| D107A | Haemoglobin E trait |
| D107y | Other specified other haemoglobinopathy |
| D107z | Other haemoglobinopathy NOS |
| D10y. | Other specified hereditary haemolytic anaemias |
| D10y0 | Stomatocytosis |
| D10yz | Other specified hereditary haemolytic anaemia NOS |
| D10z. | Hereditary haemolytic anaemia NOS |
| D11.. | Acquired haemolytic anaemias |
| D110. | Autoimmune haemolytic anaemias |
| D1100 | Primary cold-type haemolytic anaemia |
| D1101 | Primary warm-type haemolytic anaemia |
| D1102 | Secondary cold-type haemolytic anaemia |
| D1103 | Secondary warm-type haemolytic anaemia |
| D1104 | Drug-induced autoimmune haemolytic anaemia |
| D110z | Autoimmune haemolytic anaemia NOS |
| D111. | Non-autoimmune haemolytic anaemia |
| D1110 | Mechanical haemolytic anaemia |
| D1111 | Microangiopathic haemolytic anaemia |
| D1112 | Toxic haemolytic anaemia |
| D1113 | Haemolytic-uraemic syndrome |
| D1114 | Drug-induced haemolytic anaemia |
| D1115 | Infective haemolytic anaemia |
| D111y | Other specified non-autoimmune haemolytic anaemia |
| D111z | Non-autoimmune haemolytic anaemia NOS |
| D112. | Haemoglobinuria due to haemolysis from external causes |
| D1120 | Haemoglobinuria from exertion |
| D1121 | Paroxysmal nocturnal haemoglobinuria |
| D1122 | Paroxysmal cold haemoglobinuria |
| D112y | Haemoglobinuria due to haemolysis from external cause OS |
| D112z | Haemoglobinuria due to haemolysis from external cause NOS |
| D11z. | Acquired haemolytic anaemia NOS |
| D11z0 | Acquired spherocytosis |
| D1y.. | Other specified haemolytic anaemias |
| D1z.. | Haemolytic anaemias NOS |
| D2... | Aplastic and other anaemias |
| D20.. | Aplastic anaemia |
| D200. | Constitutional aplastic anaemia |
| D2000 | Congenital hypoplastic anaemia |
| D2001 | Fanconi's familial refractory anaemia |
| D2002 | Constitutional aplastic anaemia with malformation |
| D2003 | Constitutional red cell aplasia and hypoplasia |
| D200y | Other specified constitutional aplastic anaemia |
| D200z | Constitutional aplastic anaemia NOS |
| D201. | Acquired aplastic anaemia |
| D2010 | Aplastic anaemia due to chronic disease |
| D2011 | Aplastic anaemia due to drugs |
| D2012 | Aplastic anaemia due to infection |
| D2013 | Aplastic anaemia due to radiation |
| D2014 | Aplastic anaemia due to toxic cause |
| D2015 | Pancytopenia - acquired |
| D2016 | Pancytopenia NOS |
| D2017 | Transient hypoplastic anaemia |
| D2018 | [X]Pure red cell aplasia |
| D201z | Acquired aplastic anaemia NOS |
| D202. | Chronic acquired pure red cell aplasia |
| D203. | Transient acquired pure red cell aplasia |
| D2030 | Transient erythroblastopenia of childhood |
| D204. | Idiopathic aplastic anaemia |
| D20X. | Acquired pure red cell aplasia, unspecified |
| D20z. | Aplastic anaemia NOS |
| D21.. | Other and unspecified anaemias |
| D210. | Sideroblastic anaemia |
| D2100 | Congenital sideroblastic anaemia |
| D2101 | Acquired sideroblastic anaemia |
| D2102 | Pyridoxine-responsive sideroblastic anaemia |
| D2103 | Secondary sideroblastic anaemia due to disease |
| D2104 | Secondary sideroblastic anaemia due to drugs and toxins |
| D210z | Sideroblastic anaemia NOS |
| D211. | Acute posthaemorrhagic anaemia |
| D212. | Anaemia in neoplastic disease |
| D2120 | Anaemia in ovarian carcinoma |
| D213. | Refractory Anaemia |
| D214. | Chronic anaemia |
| D215. | Anaemia secondary to renal failure |
| D2150 | Anaemia secondary to chronic renal failure |
| D21y. | Other specified anaemias |
| D21y0 | Congenital dyshaematopoietic anaemia |
| D21y2 | Leukoerythroblastic anaemia |
| D21y3 | Hereditary erythroblast multinuclearity + positive acid test |
| D21yy | Other specified other anaemia |
| D21yz | Other specified anaemia NOS |
| D21z. | Anaemia unspecified |
| D2y.. | Other specified anaemias |
| D2z.. | Other anaemias NOS |
| Dyu0. | [X]Nutritional anaemias |
| Dyu00 | [X]Other iron deficiency anaemias |
| Dyu01 | [X]Other dietary vitamin B12 deficiency anaemia |
| Dyu02 | [X]Other vitamin B12 deficiency anaemias |
| Dyu03 | [X]Other folate deficiency anaemias |
| Dyu04 | [X]Other megaloblastic anaemias, not elsewhere classified |
| Dyu05 | [X]Anaem(nonmegaloblast)assoc+oth specfd nutrition deficiens |
| Dyu06 | [X]Vitamin B12 deficiency anaemia, unspecified |
| Dyu1. | [X]Haemolytic anaemias |
| Dyu10 | [X]Other anaemias due to enzyme disorders |
| Dyu11 | [X]Other thalassaemias |
| Dyu12 | [X]Other sickle-cell disorders |
| Dyu13 | [X]Other haemoglobinopathies |
| Dyu14 | [X]Other specified hereditary haemolytic anaemias |
| Dyu15 | [X]Other autoimmune haemolytic anaemias |
| Dyu16 | [X]Other nonautoimmune haemolytic anaemias |
| Dyu17 | [X]Other acquired haemolytic anaemias |
| Dyu2. | [X]Aplastic and other anaemias |
| Dyu20 | [X]Other acquired pure red cell aplasias |
| Dyu21 | [X]Other specified aplastic anaemias |
| Dyu22 | [X]Anaemia in other chronic diseases classified elsewhere |
| Dyu23 | [X]Other sideroblastic anaemias |
| Dyu24 | [X]Other specified anaemias |
| Dyu25 | [X]Acquired pure red cell aplasia, unspecified |
| L10.. | Haemorrhage in early pregnancy |
| L11.. | Antepartum haemorrhage, abruptio placentae, placenta praevia |
| L182. | Anaemia during pregnancy, childbirth and the puerperium |
| L1820 | Anaemia - unspecified whether in pregnancy or the puerperium |
| L1821 | Anaemia during pregnancy - baby delivered |
| L1822 | Anaemia in the puerperium - baby delivered |
| L1823 | Anaemia during pregnancy - baby not yet delivered |
| L1824 | Anaemia in the puerperium - baby previously delivered |
| L1825 | Iron deficiency anaemia of pregnancy |
| L182z | Anaemia during pregnancy/childbirth/puerperium NOS |
| L2A0. | Abnormal haematologic find on antenatal screening of mother |
| L2AX. | Abnormal finding on antenatal screening of mother |
| L2C.. | Malnutrition in pregnancy |
| Lyu20 | [X]Other haemorrhage in early pregnancy |

**Table 7: Hypertension READ codes**

| READ | Description |
| --- | --- |
| G2... | Hypertensive disease |
| G20.. | Essential hypertension |
| G200. | Malignant essential hypertension |
| G201. | Benign essential hypertension |
| G202. | Systolic hypertension |
| G203. | Diastolic hypertension |
| G20z. | Essential hypertension NOS |
| G24.. | Secondary hypertension |
| G240. | Secondary malignant hypertension |
| G240z | Secondary malignant hypertension NOS |
| G241. | Secondary benign hypertension |
| G241z | Secondary benign hypertension NOS |
| G244. | Hypertension secondary to endocrine disorders |
| G24z. | Secondary hypertension NOS |
| G24z0 | Secondary renovascular hypertension NOS |
| G24zz | Secondary hypertension NOS |
| G2y.. | Other specified hypertensive disease |
| G2z.. | Hypertensive disease NOS |
| Gyu2. | [X]Hypertensive diseases |
| Gyu20 | [X]Other secondary hypertension |

**Table 8: Depression ICD10 codes**

| ICD10 | Description |
| --- | --- |
| F32 | Depressive episode |
| F320 | Mild depressive episode |
| F321 | Moderate depressive episode |
| F322 | Severe depressive episode without psychotic symptoms |
| F323 | Severe depressive episode with psychotic symptoms |
| F328 | Other depressive episodes |
| F329 | Depressive episode unspecified |
| F33 | Recurrent depressive disorder |
| F330 | Recurrent depressive disorder current episode mild |
| F331 | Recurrent depressive disorder current episode moderate |
| F332 | Recurrent depress disorder cur epi severe without psyc symp |
| F333 | Recurrent depress disorder cur epi severe with psyc symp |
| F334 | Recurrent depressive disorder currently in remission |
| F338 | Other recurrent depressive disorders |
| F339 | Recurrent depressive disorder unspecified |

**Table 9: Depression READ codes**

| READ | Description |
| --- | --- |
| E0013 | Presenile dementia with depression |
| E0021 | Senile dementia with depression |
| E112. | Single major depressive episode |
| E1120 | Single major depressive episode, unspecified |
| E1121 | Single major depressive episode, mild |
| E1122 | Single major depressive episode, moderate |
| E1123 | Single major depressive episode, severe, without psychosis |
| E1124 | Single major depressive episode, severe, with psychosis |
| E1125 | Single major depressive episode, partial or unspec remission |
| E1126 | Single major depressive episode, in full remission |
| E112z | Single major depressive episode NOS |
| E113. | Recurrent major depressive episode |
| E1130 | Recurrent major depressive episodes, unspecified |
| E1131 | Recurrent major depressive episodes, mild |
| E1132 | Recurrent major depressive episodes, moderate |
| E1133 | Recurrent major depressive episodes, severe, no psychosis |
| E1134 | Recurrent major depressive episodes, severe, with psychosis |
| E1135 | Recurrent major depressive episodes, partial/unspec remission |
| E1136 | Recurrent major depressive episodes, in full remission |
| E1137 | Recurrent depression |
| E113z | Recurrent major depressive episode NOS |
| E118. | Seasonal affective disorder |
| E11y2 | Atypical depressive disorder |
| E11z2 | Masked depression |
| E130. | Reactive depressive psychosis |
| E135. | Agitated depression |
| E2003 | Anxiety with depression |
| E291. | Prolonged depressive reaction |
| E2B.. | Depressive disorder NEC |
| E2B1. | Chronic depression |
| Eu204 | [X]Post-schizophrenic depression |
| Eu251 | [X]Schizoaffective disorder, depressive type |
| Eu32. | [X]Depressive episode |
| Eu320 | [X]Mild depressive episode |
| Eu321 | [X]Moderate depressive episode |
| Eu322 | [X]Severe depressive episode without psychotic symptoms |
| Eu323 | [X]Severe depressive episode with psychotic symptoms |
| Eu324 | [X]Mild depression |
| Eu325 | [X]Major depression, mild |
| Eu326 | [X]Major depression, moderately severe |
| Eu327 | [X]Major depression, severe without psychotic symptoms |
| Eu328 | [X]Major depression, severe with psychotic symptoms |
| Eu32y | [X]Other depressive episodes |
| Eu32z | [X]Depressive episode, unspecified |
| Eu33. | [X]Recurrent depressive disorder |
| Eu330 | [X]Recurrent depressive disorder, current episode mild |
| Eu331 | [X]Recurrent depressive disorder, current episode moderate |
| Eu332 | [X]Recurr depress disorder cur epi severe without psyc sympt |
| Eu333 | [X]Recurrent depress disorder cur epi severe with psyc symp |
| Eu334 | [X]Recurrent depressive disorder, currently in remission |
| Eu33y | [X]Other recurrent depressive disorders |
| Eu33z | [X]Recurrent depressive disorder, unspecified |
| Eu341 | [X]Dysthymia |
| Eu412 | [X]Mixed anxiety and depressive disorder |

**Table 10: Anxiety ICD10 codes**

| ICD10 | Description |
| --- | --- |
| F064 | Organic anxiety disorder |
| F40 | Phobic anxiety disorders |
| F400 | Agoraphobia |
| F401 | Social phobias |
| F402 | Specific (isolated) phobias |
| F408 | Other phobic anxiety disorders |
| F409 | Phobic anxiety disorder unspecified |
| F41 | Other anxiety disorders |
| F410 | Panic disorder [episodic paroxysmal anxiety] |
| F411 | Generalized anxiety disorder |
| F412 | Mixed anxiety and depressive disorder |
| F413 | Other mixed anxiety disorders |
| F418 | Other specified anxiety disorders |
| F419 | Anxiety disorder unspecified |
| F930 | Separation anxiety disorder of childhood |
| F931 | Phobic anxiety disorder of childhood |
| F932 | Social anxiety disorder of childhood |

**Table 11: Anxiety READ codes**

| READ | Description |
| --- | --- |
| 1B12. | Nerves - nervousness |
| 1B13. | Anxiousness |
| 2258. | O/E - anxious |
| 2259. | O/E - nervous |
| E200. | Anxiety states |
| E2000 | Anxiety state unspecified |
| E2001 | Panic disorder |
| E2002 | Generalised anxiety disorder |
| E2003 | Anxiety with depression |
| E2004 | Chronic anxiety |
| E2005 | Recurrent anxiety |
| E200z | Anxiety state NOS |
| E2D0. | Disturbance of anxiety and fearfulness childhood/adolescent |
| E2D00 | Childhood and adolescent over anxiousness disturbance |
| E2D0z | Disturbance anxiety and fearfulness childhood/adolescent NOS |
| Eu41. | [X]Other anxiety disorders |
| Eu410 | [X]Panic disorder [episodic paroxysmal anxiety] |
| Eu411 | [X]Generalized anxiety disorder |
| Eu412 | [X]Mixed anxiety and depressive disorder |
| Eu413 | [X]Other mixed anxiety disorders |
| Eu41y | [X]Other specified anxiety disorders |
| Eu41z | [X]Anxiety disorder, unspecified |
| Eu930 | [X]Separation anxiety disorder of childhood |
| Eu931 | [X]Phobic anxiety disorder of childhood |
| Eu932 | [X]Social anxiety disorder of childhood |
| R2y2. | [D]Nervousness |

**Table 12: Anxiety and Depression medication READ codes**

| READ | Description |
| --- | --- |
| d11.. | CHLORAL HYDRATE |
| d12.. | CLOMETHIAZOLE EDISYLATE [HYPNOTIC] |
| d13.. | *DICHLORALPHENAZONE |
| d14.. | *FLUNITRAZEPAM |
| d15.. | FLURAZEPAM |
| d16.. | LOPRAZOLAM |
| d17.. | LORMETAZEPAM |
| d18.. | NITRAZEPAM |
| d1a.. | TEMAZEPAM [HYPNOTIC] |
| d1b.. | *TRIAZOLAM |
| d1c.. | TRICLOFOS SODIUM |
| d1d.. | ZOPICLONE |
| d1f.. | ZOLPIDEM |
| d1g.. | ZALEPLON |
| d1h.. | MELATONIN |
| d21.. | DIAZEPAM [ANXIOLYTIC] |
| d22.. | ALPRAZOLAM |
| d23.. | BROMAZEPAM |
| d24.. | CHLORDIAZEPOXIDE |
| d25.. | CHLORMEZANONE |
| d26.. | CLOBAZAM |
| d27.. | CLORAZEPATE DIPOTASSIUM |
| d28.. | HYDROXYZINE HCL [ANXIOLYTIC] |
| d29.. | *KETAZOLAM |
| d2a.. | LORAZEPAM [ANXIOLYTIC] |
| d2b.. | *MEDAZEPAM |
| d2c.. | MEPROBAMATE |
| d2d.. | OXAZEPAM |
| d2e.. | *PRAZEPAM |
| d2f.. | BUSPIRONE HYDROCHLORIDE |
| d2g.. | FLUMAZENIL |
| d71.. | AMITRIPTYLINE HYDROCHLORIDE [ANTIDEPRESSANT] |
| d72.. | *BUTRIPTYLINE |
| d73.. | CLOMIPRAMINE HYDROCHLORIDE |
| d74.. | DESIPRAMINE HYDROCHLORIDE |
| d75.. | DOSULEPIN HYDROCHLORIDE |
| d76.. | DOXEPIN |
| d77.. | IMIPRAMINE HYDROCHLORIDE [ANTIDEPRESSANT] |
| d78.. | IPRINDOLE |
| d79.. | LOFEPRAMINE |
| d7a.. | MAPROTILINE HYDROCHLORIDE |
| d7b.. | MIANSERIN HYDROCHLORIDE |
| d7c.. | NORTRIPTYLINE |
| d7d.. | PROTRIPTYLINE HYDROCHLORIDE |
| d7e.. | TRAZODONE HYDROCHLORIDE |
| d7f.. | TRIMIPRAMINE |
| d7g.. | VILOXAZINE HYDROCHLORIDE |
| d7h.. | AMOXAPINE |
| d81.. | PHENELZINE |
| d83.. | ISOCARBOXAZID |
| d84.. | TRANYLCYPROMINE |
| d85.. | MOCLOBEMIDE |
| d91.. | COMPOUND ANTIDEPRESSANTS A-Z |
| da1.. | FLUPENTIXOL [ANTIDEPRESSANT] |
| da2.. | TRYPTOPHAN |
| da3.. | FLUVOXAMINE MALEATE |
| da4.. | FLUOXETINE HYDROCHLORIDE |
| da5.. | SERTRALINE HYDROCHLORIDE |
| da6.. | PAROXETINE HYDROCHLORIDE |
| da7.. | VENLAFAXINE |
| da9.. | CITALOPRAM |
| daA.. | REBOXETINE |
| daB.. | MIRTAZAPINE |
| daC.. | ESCITALOPRAM |
| daD.. | AGOMELATINE |
| gde.. | DULOXETINE |

**Table 13: Serious mental illness ICD10 codes**

| ICD10 | Description |
| --- | --- |
| F200 | Paranoid schizophrenia |
| F201 | Hebephrenic schizophrenia |
| F202 | Catatonic schizophrenia |
| F203 | Undifferentiated schizophrenia |
| F204 | Post-schizophrenic depression |
| F205 | Residual schizophrenia |
| F206 | Simple schizophrenia |
| F208 | Other schizophrenia |
| F209 | Schizophrenia unspecified |
| F21X | Schizotypal disorder |
| F220 | Delusional disorder |
| F228 | Other persistent delusional disorders |
| F229 | Persistent delusional disorderunspecified |
| F230 | Acute polymorphic psychot disord without symp of schizoph'a |
| F231 | Acute polymorphic psychot disord with symp of schizophrenia |
| F232 | Acute schizophrenia-like psychotic disorder |
| F233 | Other acute predominantly delusional psychotic disorders |
| F238 | Other acute and transient psychotic disorders |
| F239 | Acute and transient psychotic disorderunspecified |
| F24X | Induced delusional disorder |
| F250 | Schizoaffective disordermanic type |
| F251 | Schizoaffective disorderdepressive type |
| F252 | Schizoaffective disordermixed type |
| F258 | Other schizoaffective disorders |
| F259 | Schizoaffective disorderunspecified |
| F28X | Other nonorganic psychotic disorders |
| F29X | Unspecified nonorganic psychosis |
| F300 | Hypomania |
| F301 | Mania without psychotic symptoms |
| F302 | Mania with psychotic symptoms |
| F308 | Other manic episodes |
| F309 | Manic episode unspecified |
| F310 | Bipolar affective disorder current episode hypomanic |
| F311 | Bipolar affect disorder cur epi manic without psychotic symp |
| F312 | Bipolar affect disorder cur epi manic with psychotic symp |
| F313 | Bipolar affect disorder cur epi mild or moderate depression |
| F314 | Bipolar affect disorder cur epi sev depres without psyc symp |
| F315 | Bipolar affect disorder cur epi severe depres with psyc symp |
| F316 | Bipolar affective disorder current episode mixed |
| F317 | Bipolar affective disorder currently in remission |
| F318 | Other bipolar affective disorders |
| F319 | Bipolar affective disorder unspecified |
| F323 | Severe depressive episode with psychotic symptoms |
| F333 | Recurrent depress disorder cur epi severe with psyc symp |
| F39X | Unspecified mood [affective] disorder |

**Table 14: Serious mental illness READ codes**

| READ | Description |
| --- | --- |
| E10.. | Schizophrenic disorders |
| E100. | Simple schizophrenia |
| E1000 | Unspecified schizophrenia |
| E1001 | Subchronic schizophrenia |
| E1002 | Chronic schizophrenic |
| E1003 | Acute exacerbation of subchronic schizophrenia |
| E1004 | Acute exacerbation of chronic schizophrenia |
| E1005 | Schizophrenia in remission |
| E100z | Simple schizophrenia NOS |
| E101. | Hebephrenic schizophrenia |
| E1010 | Unspecified hebephrenic schizophrenia |
| E1011 | Subchronic hebephrenic schizophrenia |
| E1012 | Chronic hebephrenic schizophrenia |
| E1013 | Acute exacerbation of subchronic hebephrenic schizophrenia |
| E1014 | Acute exacerbation of chronic hebephrenic schizophrenia |
| E1015 | Hebephrenic schizophrenia in remission |
| E101z | Hebephrenic schizophrenia NOS |
| E102. | Catatonic schizophrenia |
| E1020 | Unspecified catatonic schizophrenia |
| E1021 | Subchronic catatonic schizophrenia |
| E1022 | Chronic catatonic schizophrenia |
| E1023 | Acute exacerbation of subchronic catatonic schizophrenia |
| E1024 | Acute exacerbation of chronic catatonic schizophrenia |
| E1025 | Catatonic schizophrenia in remission |
| E102z | Catatonic schizophrenia NOS |
| E103. | Paranoid schizophrenia |
| E1030 | Unspecified paranoid schizophrenia |
| E1031 | Subchronic paranoid schizophrenia |
| E1032 | Chronic paranoid schizophrenia |
| E1033 | Acute exacerbation of subchronic paranoid schizophrenia |
| E1034 | Acute exacerbation of chronic paranoid schizophrenia |
| E1035 | Paranoid schizophrenia in remission |
| E103z | Paranoid schizophrenia NOS |
| E104. | Acute schizophrenic episode |
| E105. | Latent schizophrenia |
| E1050 | Unspecified latent schizophrenia |
| E1051 | Subchronic latent schizophrenia |
| E1052 | Chronic latent schizophrenia |
| E1053 | Acute exacerbation of subchronic latent schizophrenia |
| E1054 | Acute exacerbation of chronic latent schizophrenia |
| E1055 | Latent schizophrenia in remission |
| E105z | Latent schizophrenia NOS |
| E106. | Residual schizophrenia |
| E107. | Schizo-affective schizophrenia |
| E1070 | Unspecified schizo-affective schizophrenia |
| E1071 | Subchronic schizo-affective schizophrenia |
| E1072 | Chronic schizo-affective schizophrenia |
| E1073 | Acute exacerbation subchronic schizo-affective schizophrenia |
| E1074 | Acute exacerbation of chronic schizo-affective schizophrenia |
| E1075 | Schizo-affective schizophrenia in remission |
| E107z | Schizo-affective schizophrenia NOS |
| E10y. | Other schizophrenia |
| E10y0 | Atypical schizophrenia |
| E10y1 | Coenesthopathic schizophrenia |
| E10yz | Other schizophrenia NOS |
| E10z. | Schizophrenia NOS |
| E110. | Manic disorder, single episode |
| E1100 | Single manic episode, unspecified |
| E1101 | Single manic episode, mild |
| E1102 | Single manic episode, moderate |
| E1103 | Single manic episode, severe without mention of psychosis |
| E1104 | Single manic episode, severe, with psychosis |
| E1105 | Single manic episode in partial or unspecified remission |
| E1106 | Single manic episode in full remission |
| E110z | Manic disorder, single episode NOS |
| E111. | Recurrent manic episodes |
| E1110 | Recurrent manic episodes, unspecified |
| E1111 | Recurrent manic episodes, mild |
| E1112 | Recurrent manic episodes, moderate |
| E1113 | Recurrent manic episodes, severe without mention psychosis |
| E1114 | Recurrent manic episodes, severe, with psychosis |
| E1115 | Recurrent manic episodes, partial or unspecified remission |
| E1116 | Recurrent manic episodes, in full remission |
| E111z | Recurrent manic episode NOS |
| E1124 | Single major depressive episode, severe, with psychosis |
| E1134 | Recurrent major depressive episodes, severe, with psychosis |
| E114. | Bipolar affective disorder, currently manic |
| E1140 | Bipolar affective disorder, currently manic, unspecified |
| E1141 | Bipolar affective disorder, currently manic, mild |
| E1142 | Bipolar affective disorder, currently manic, moderate |
| E1143 | Bipolar affect disord, currently manic, severe, no psychosis |
| E1144 | Bipolar affect disord, currently manic,severe with psychosis |
| E1145 | Bipolar affect disord,currently manic, part/unspec remission |
| E1146 | Bipolar affective disorder, currently manic, full remission |
| E114z | Bipolar affective disorder, currently manic, NOS |
| E115. | Bipolar affective disorder, currently depressed |
| E1150 | Bipolar affective disorder, currently depressed, unspecified |
| E1151 | Bipolar affective disorder, currently depressed, mild |
| E1152 | Bipolar affective disorder, currently depressed, moderate |
| E1153 | Bipolar affect disord, now depressed, severe, no psychosis |
| E1154 | Bipolar affect disord, now depressed, severe with psychosis |
| E1155 | Bipolar affect disord, now depressed, part/unspec remission |
| E1156 | Bipolar affective disorder, now depressed, in full remission |
| E115z | Bipolar affective disorder, currently depressed, NOS |
| E116. | Mixed bipolar affective disorder |
| E1160 | Mixed bipolar affective disorder, unspecified |
| E1161 | Mixed bipolar affective disorder, mild |
| E1162 | Mixed bipolar affective disorder, moderate |
| E1163 | Mixed bipolar affective disorder, severe, without psychosis |
| E1164 | Mixed bipolar affective disorder, severe, with psychosis |
| E1165 | Mixed bipolar affective disorder, partial/unspec remission |
| E1166 | Mixed bipolar affective disorder, in full remission |
| E116z | Mixed bipolar affective disorder, NOS |
| E117. | Unspecified bipolar affective disorder |
| E1170 | Unspecified bipolar affective disorder, unspecified |
| E1171 | Unspecified bipolar affective disorder, mild |
| E1172 | Unspecified bipolar affective disorder, moderate |
| E1173 | Unspecified bipolar affective disorder, severe, no psychosis |
| E1174 | Unspecified bipolar affective disorder,severe with psychosis |
| E1175 | Unspecified bipolar affect disord, partial/unspec remission |
| E1176 | Unspecified bipolar affective disorder, in full remission |
| E117z | Unspecified bipolar affective disorder, NOS |
| E11y. | Other and unspecified manic-depressive psychoses |
| E11y0 | Unspecified manic-depressive psychoses |
| E11y1 | Atypical manic disorder |
| E11y3 | Other mixed manic-depressive psychoses |
| E11yz | Other and unspecified manic-depressive psychoses NOS |
| E11z. | Other and unspecified affective psychoses |
| E11z0 | Unspecified affective psychoses NOS |
| E11zz | Other affective psychosis NOS |
| E12.. | Paranoid states |
| E120. | Simple paranoid state |
| E121. | Chronic paranoid psychosis |
| E122. | Paraphrenia |
| E123. | Shared paranoid disorder |
| E12y. | Other paranoid states |
| E12y0 | Paranoia querulans |
| E12yz | Other paranoid states NOS |
| E12z. | Paranoid psychosis NOS |
| E13.. | Other nonorganic psychoses |
| E130. | Reactive depressive psychosis |
| E131. | Acute hysterical psychosis |
| E132. | Reactive confusion |
| E133. | Acute paranoid reaction |
| E134. | Psychogenic paranoid psychosis |
| E13y. | Other reactive psychoses |
| E13y0 | Psychogenic stupor |
| E13y1 | Brief reactive psychosis |
| E13yz | Other reactive psychoses NOS |
| E13z. | Nonorganic psychosis NOS |
| E2122 | Schizotypal personality |
| Eu2.. | [X]Schizophrenia, schizotypal and delusional disorders |
| Eu20. | [X]Schizophrenia |
| Eu200 | [X]Paranoid schizophrenia |
| Eu201 | [X]Hebephrenic schizophrenia |
| Eu202 | [X]Catatonic schizophrenia |
| Eu203 | [X]Undifferentiated schizophrenia |
| Eu204 | [X]Post-schizophrenic depression |
| Eu205 | [X]Residual schizophrenia |
| Eu206 | [X]Simple schizophrenia |
| Eu20y | [X]Other schizophrenia |
| Eu20z | [X]Schizophrenia, unspecified |
| Eu21. | [X]Schizotypal disorder |
| Eu22. | [X]Persistent delusional disorders |
| Eu220 | [X]Delusional disorder |
| Eu221 | [X]Delusional misidentification syndrome |
| Eu222 | [X]Cotard syndrome |
| Eu223 | [X]Paranoid state in remission |
| Eu22y | [X]Other persistent delusional disorders |
| Eu22z | [X]Persistent delusional disorder, unspecified |
| Eu23. | [X]Acute and transient psychotic disorders |
| Eu230 | [X]Acute polymorphic psychot disord without symp of schizoph |
| Eu231 | [X]Acute polymorphic psychot disord with symp of schizophren |
| Eu232 | [X]Acute schizophrenia-like psychotic disorder |
| Eu233 | [X]Other acute predominantly delusional psychotic disorders |
| Eu23y | [X]Other acute and transient psychotic disorders |
| Eu23z | [X]Acute and transient psychotic disorder, unspecified |
| Eu24. | [X]Induced delusional disorder |
| Eu25. | [X]Schizoaffective disorders |
| Eu250 | [X]Schizoaffective disorder, manic type |
| Eu251 | [X]Schizoaffective disorder, depressive type |
| Eu252 | [X]Schizoaffective disorder, mixed type |
| Eu25y | [X]Other schizoaffective disorders |
| Eu25z | [X]Schizoaffective disorder, unspecified |
| Eu26. | [X]Nonorganic psychosis in remission |
| Eu2y. | [X]Other nonorganic psychotic disorders |
| Eu2z. | [X]Unspecified nonorganic psychosis |
| Eu30. | [X]Manic episode |
| Eu300 | [X]Hypomania |
| Eu301 | [X]Mania without psychotic symptoms |
| Eu302 | [X]Mania with psychotic symptoms |
| Eu30y | [X]Other manic episodes |
| Eu30z | [X]Manic episode, unspecified |
| Eu31. | [X]Bipolar affective disorder |
| Eu310 | [X]Bipolar affective disorder, current episode hypomanic |
| Eu311 | [X]Bipolar affect disorder cur epi manic wout psychotic symp |
| Eu312 | [X]Bipolar affect disorder cur epi manic with psychotic symp |
| Eu313 | [X]Bipolar affect disorder cur epi mild or moderate depressn |
| Eu314 | [X]Bipol aff disord, curr epis sev depress, no psychot symp |
| Eu315 | [X]Bipolar affect dis cur epi severe depres with psyc symp |
| Eu316 | [X]Bipolar affective disorder, current episode mixed |
| Eu317 | [X]Bipolar affective disorder, currently in remission |
| Eu318 | [X]Bipolar affective disorder type I |
| Eu319 | [X]Bipolar affective disorder type II |
| Eu31y | [X]Other bipolar affective disorders |
| Eu31z | [X]Bipolar affective disorder, unspecified |
| Eu323 | [X]Severe depressive episode with psychotic symptoms |
| Eu333 | [X]Recurrent depress disorder cur epi severe with psyc symp |

**Table 15: Assault ICD10 codes**

| ICD10 | Description |
| --- | --- |
| X90 | Assault by unspecified chemical or noxious substance |
| X900 | Occurrence at home |
| X901 | Occurrence in residential institution |
| X902 | Occurrence at school other instit'n / pub admin area |
| X903 | Occurrence at sports / athletics area |
| X904 | Occurrence on street / highway |
| X905 | Occurrence at trade / service area |
| X906 | Occurrence at industrial / construction area |
| X907 | Occurrence on farm |
| X908 | Occurrence at other specified place |
| X909 | Occurrence at unspecified place |
| X91 | Assault by hanging strangulation and suffocation |
| X910 | Occurrence at home |
| X911 | Occurrence in residential institution |
| X912 | Occurrence at school other instit'n / pub admin area |
| X913 | Occurrence at sports / athletics area |
| X914 | Occurrence on street / highway |
| X915 | Occurrence at trade / service area |
| X916 | Occurrence at industrial / construction area |
| X917 | Occurrence on farm |
| X918 | Occurrence at other specified place |
| X919 | Occurrence at unspecified place |
| X92 | Assault by drowning and submersion |
| X920 | Occurrence at home |
| X921 | Occurrence in residential institution |
| X922 | Occurrence at school other instit'n / pub admin area |
| X923 | Occurrence at sports / athletics area |
| X924 | Occurrence on street / highway |
| X925 | Occurrence at trade / service area |
| X926 | Occurrence at industrial / construction area |
| X927 | Occurrence on farm |
| X928 | Occurrence at other specified place |
| X929 | Occurrence at unspecified place |
| X93 | Assault by handgun discharge |
| X930 | Occurrence at home |
| X931 | Occurrence in residential institution |
| X932 | Occurrence at school other instit'n / pub admin area |
| X933 | Occurrence at sports / athletics area |
| X934 | Occurrence on street / highway |
| X935 | Occurrence at trade / service area |
| X936 | Occurrence at industrial / construction area |
| X937 | Occurrence on farm |
| X938 | Occurrence at other specified place |
| X939 | Occurrence at unspecified place |
| X94 | Assault by rifle shotgun and larger firearm discharge |
| X940 | Occurrence at home |
| X941 | Occurrence in residential institution |
| X942 | Occurrence at school other instit'n/pub admin area |
| X943 | Occurrence at sports / athletics area |
| X944 | Occurrence on street / highway |
| X945 | Occurrence at trade / service area |
| X946 | Occurrence at industrial / construction area |
| X947 | Occurrence on farm |
| X948 | Occurrence at other specified place |
| X949 | Occurrence at unspecified place |
| X95 | Assault by other and unspecified firearm discharge |
| X950 | Occurrence at home |
| X951 | Occurrence in residential institution |
| X952 | Occurrence at school other instit'n / pub admin area |
| X953 | Occurrence at sports / athletics area |
| X954 | Occurrence on street / highway |
| X955 | Occurrence at trade / service area |
| X956 | Occurrence at industrial / construction area |
| X957 | Occurrence on farm |
| X958 | Occurrence at other specified place |
| X959 | Occurrence at unspecified place |
| X96 | Assault by explosive material |
| X960 | Occurrence at home |
| X961 | Occurrence in residential institution |
| X962 | Occurrence at school other instit'n / pub admin area |
| X963 | Occurrence at sports / athletics area |
| X964 | Occurrence on street / highway |
| X965 | Occurrence at trade / service area |
| X966 | Occurrence at industrial / construction area |
| X967 | Occurrence on farm |
| X968 | Occurrence at other specified place |
| X969 | Occurrence at unspecified place |
| X97 | Assault by smoke fire and flames |
| X970 | Occurrence at home |
| X971 | Occurrence in residential institution |
| X972 | Occurrence at school other instit'n / pub admin area |
| X973 | Occurrence at sports / athletics area |
| X974 | Occurrence on street / highway |
| X975 | Occurrence at trade / service area |
| X976 | Occurrence at industrial / construction area |
| X977 | Occurrence on farm |
| X978 | Occurrence at other specified place |
| X979 | Occurrence at unspecified place |
| X98 | Assault by steam hot vapours and hot objects |
| X980 | Occurrence at home |
| X981 | Occurrence in residential institution |
| X982 | Occurrence at school other instit'n / pub admin area |
| X983 | Occurrence at sports / athletics area |
| X984 | Occurrence on street / highway |
| X985 | Occurrence at trade / service area |
| X986 | Occurrence at industrial / construction area |
| X987 | Occurrence on farm |
| X988 | Occurrence at other specified place |
| X989 | Occurrence at unspecified place |
| X99 | Assault by sharp object |
| X990 | Occurrence at home |
| X991 | Occurrence in residential institution |
| X992 | Occurrence at school other instit'n / pub admin area |
| X993 | Occurrence at sports / athletics area |
| X994 | Occurrence on street / highway |
| X995 | Occurrence at trade / service area |
| X996 | Occurrence at industrial / construction area |
| X997 | Occurrence on farm |
| X998 | Occurrence at other specified place |
| X999 | Occurrence at unspecified place |
| Y00 | Assault by blunt object |
| Y000 | Occurrence at home |
| Y001 | Occurrence in residential institution |
| Y002 | Occurrence at school other instit'n / pub admin area |
| Y003 | Occurrence at sports / athletics area |
| Y004 | Occurrence on street / highway |
| Y005 | Occurrence at trade / service area |
| Y006 | Occurrence at industrial / construction area |
| Y007 | Occurrence on farm |
| Y008 | Occurrence at other specified place |
| Y009 | Occurrence at unspecified place |
| Y01 | Assault by pushing from high place |
| Y010 | Occurrence at home |
| Y011 | Occurrence in residential institution |
| Y012 | Occurrence at school other instit'n / pub admin area |
| Y013 | Occurrence at sports / athletics area |
| Y014 | Occurrence on street / highway |
| Y015 | Occurrence at trade / service area |
| Y016 | Occurrence at industrial / construction area |
| Y017 | Occurrence on farm |
| Y018 | Occurrence at other specified place |
| Y019 | Occurrence at unspecified place |
| Y02 | Assault by pushing or placing victim before moving object |
| Y020 | Occurrence at home |
| Y021 | Occurrence in residential institution |
| Y022 | Occurrence at school other instit'n / pub admin area |
| Y023 | Occurrence at sports / athletics area |
| Y024 | Occurrence on street / highway |
| Y025 | Occurrence at trade / service area |
| Y026 | Occurrence at industrial / construction area |
| Y027 | Occurrence on farm |
| Y028 | Occurrence at other specified place |
| Y029 | Occurrence at unspecified place |
| Y03 | Assault by crashing of motor vehicle |
| Y030 | Occurrence at home |
| Y031 | Occurrence in residential institution |
| Y032 | Occurrence at school other instit'n / pub admin area |
| Y033 | Occurrence at sports / athletics area |
| Y034 | Occurrence on street / highway |
| Y035 | Occurrence at trade / service area |
| Y036 | Occurrence at industrial / construction area |
| Y037 | Occurrence on farm |
| Y038 | Occurrence at other specified place |
| Y039 | Occurrence at unspecified place |
| Y04 | Assault by bodily force |
| Y040 | Occurrence at home |
| Y041 | Occurrence in residential institution |
| Y042 | Occurrence at school other instit'n / pub admin area |
| Y043 | Occurrence at sports / athletics area |
| Y044 | Occurrence on street / highway |
| Y045 | Occurrence at trade / service area |
| Y046 | Occurrence at industrial / construction area |
| Y047 | Occurrence on farm |
| Y048 | Occurrence at other specified place |
| Y049 | Occurrence at unspecified place |
| Y05 | Sexual assault by bodily force |
| Y050 | Occurrence at home |
| Y051 | Occurrence in residential institution |
| Y052 | Occurrence at school other instit'n / pub admin area |
| Y053 | Occurrence at sports / athletics area |
| Y054 | Occurrence on street / highway |
| Y055 | Occurrence at trade / service area |
| Y056 | Occurrence at industrial / construction area |
| Y057 | Occurrence on farm |
| Y058 | Occurrence at other specified place |
| Y059 | Occurrence at unspecified place |
| Y06 | Neglect and abandonment |
| Y060 | By spouse or partner |
| Y061 | By parent |
| Y062 | By acquaintance or friend |
| Y068 | By other specified persons |
| Y069 | By unspecified person |
| Y07 | Other maltreatment syndromes |
| Y070 | By spouse or partner |
| Y071 | By parent |
| Y072 | By acquaintance or friend |
| Y073 | By official authorities |
| Y078 | By other specified persons |
| Y079 | By unspecified person |
| Y08 | Assault by other specified means |
| Y080 | Occurrence at home |
| Y081 | Occurrence in residential institution |
| Y082 | Occurrence at school other instit'n / pub admin area |
| Y083 | Occurrence at sports / athletics area |
| Y084 | Occurrence on street / highway |
| Y085 | Occurrence at trade / service area |
| Y086 | Occurrence at industrial / construction area |
| Y087 | Occurrence on farm |
| Y088 | Occurrence at other specified place |
| Y089 | Occurrence at unspecified place |
| Y09 | Assault by unspecified means |
| Y090 | Occurrence at home |
| Y091 | Occurrence in residential institution |
| Y092 | Occurrence at school other instit'n / pub admin area |
| Y093 | Occurrence at sports / athletics area |
| Y094 | Occurrence on street / highway |
| Y095 | Occurrence at trade / service area |
| Y096 | Occurrence at industrial / construction area |
| Y097 | Occurrence on farm |
| Y098 | Occurrence at other specified place |
| Y099 | Occurrence at unspecified place |

**Table 16: Learning difficulty READ codes**

| READ | Description |
| --- | --- |
| 1J9.. | Suspected autism |
| 69DB. | Learning disability health examination |
| 8Ce6. | Preferred place of care - learning disability unit |
| 8H4f. | Referral to learning disabilities psychiatrist |
| 8HHP. | Referral to learning disability team |
| 8Hg2. | Discharge from learning disability team |
| 918e. | On learning disability register |
| 94Z9. | Preferred place of death: learning disability unit |
| 9HB.. | Learning disabilities administration status |
| 9HB0. | Learning disabilities health action plan declined |
| 9HB1. | Learning disabilities health action plan offered |
| 9HB2. | Learning disabilities health action plan reviewed |
| 9HB3. | Learning disabilities health assessment |
| 9HB4. | Learning disabilities health action plan completed |
| 9HB5. | Learning disabilities annual health assessment |
| 9HB6. | Learning disabilities annual health assessment declined |
| 9HB7. | Did not attend learning disabilities annual health assessmnt |
| 9N0y. | Seen in learning disabilities clinic |
| 9hL.. | Exception reporting: learning disability quality indicators |
| 9mA.. | Learning disability annual health check invitation |
| 9mA0. | Learning disability annual health check verbal invitation |
| 9mA1. | Learning disability annual health check telephone invitation |
| 9mA2. | Learning disability annual health check letter invitation |
| 9mA20 | Learning disability annual health check invtation 1st letter |
| 9mA21 | Learning disability annual health check invtation 2nd letter |
| 9mA22 | Learning disability annual health check invtation 3rd letter |
| E140. | Infantile autism |
| E1400 | Active infantile autism |
| E1401 | Residual infantile autism |
| E140z | Infantile autism NOS |
| E3... | Mental retardation |
| E30.. | Mild mental retardation, IQ in range 50-70 |
| E31.. | Other specified mental retardation |
| E310. | Moderate mental retardation, IQ in range 35-49 |
| E311. | Severe mental retardation, IQ in range 20-34 |
| E312. | Profound mental retardation with IQ less than 20 |
| E31z. | Other specified mental retardation NOS |
| E3y.. | Other specified mental retardation |
| E3z.. | Mental retardation NOS |
| Eu7.. | [X]Mental retardation |
| Eu70. | [X]Mild mental retardation |
| Eu700 | [X]Mld mental retard with statement no min impairm behav |
| Eu701 | [X]Mld mental retard sig impairment behav req attent/treatmt |
| Eu70y | [X]Mild mental retardation, other impairments of behaviour |
| Eu70z | [X]Mild mental retardation without mention impairment behav |
| Eu71. | [X]Moderate mental retardation |
| Eu710 | [X]Mod mental retard with statement no min impairm behav |
| Eu711 | [X]Mod mental retard sig impairment behav req attent/treatmt |
| Eu71y | [X]Mod retard oth behav impair |
| Eu71z | [X]Mod mental retardation without mention impairment behav |
| Eu72. | [X]Severe mental retardation |
| Eu720 | [X]Sev mental retard with statement no min impairm behav |
| Eu721 | [X]Sev mental retard sig impairment behav req attent/treatmt |
| Eu72y | [X]Severe mental retardation, other impairments of behaviour |
| Eu72z | [X]Sev mental retardation without mention impairment behav |
| Eu73. | [X]Profound mental retardation |
| Eu730 | [X]Profound ment retrd wth statement no min impairm behav |
| Eu731 | [X]Profound ment retard sig impairmnt behav req attent/treat |
| Eu73y | [X]Profound mental retardation, other impairments of behavr |
| Eu73z | [X]Prfnd mental retardation without mention impairment behav |
| Eu7y. | [X]Other mental retardation |
| Eu7y0 | [X]Oth mental retard with statement no min impairm behav |
| Eu7y1 | [X]Oth mental retard sig impairment behav req attent/treatmt |
| Eu7yy | [X]Other mental retardation, other impairments of behaviour |
| Eu7yz | [X]Other mental retardation without mention impairment behav |
| Eu7z. | [X]Unspecified mental retardation |
| Eu7z0 | [X]Unsp mental retard with statement no min impairm behav |
| Eu7z1 | [X]Unsp mentl retard sig impairment behav req attent/treatmt |
| Eu7zy | [X]Unspecified mental retardatn, other impairments of behav |
| Eu7zz | [X]Unsp mental retardation without mention impairment behav |
| Eu814 | [X]Moderate learning disability |
| Eu815 | [X]Severe learning disability |
| Eu816 | [X]Mild learning disability |
| Eu817 | [X]Profound learning disability |
| Eu818 | [X]Specific learning disability |
| Eu81z | [X]Developmental disorder of scholastic skills, unspecified |
| Eu840 | [X]Childhood autism |
| Eu841 | [X]Atypical autism |
| PJ0.. | Down's syndrome - trisomy 21 |
| PJ00. | Trisomy 21, meiotic nondisjunction |
| PJ01. | Trisomy 21, mosaicism |
| PJ02. | Trisomy 21, translocation |
| PJ0z. | Down's syndrome NOS |

**Table 17: Alcohol related health conditions ICD10 codes**

| ICD10 | Description |
| --- | --- |
| E244 | Alcohol-induced pseudo-Cushing's syndrome |
| E512 | Wernicke's encephalopathy |
| F10 | Mental and behavioural disorders due to use of alcohol |
| F100 | Mental and behavioural disorders due to use of alcohol |
| F101 | Mental and behavioural disorders due to use of alcohol |
| F102 | Mental and behavioural disorders due to use of alcohol |
| F103 | Mental and behavioural disorders due to use of alcohol |
| F104 | Mental and behavioural disorders due to use of alcohol |
| F105 | Mental and behavioural disorders due to use of alcohol |
| F106 | Mental and behavioural disorders due to use of alcohol |
| F107 | Mental and behavioural disorders due to use of alcohol |
| F108 | Mental and behavioural disorders due to use of alcohol |
| F109 | Mental and behavioural disorders due to use of alcohol |
| G312 | Degeneration of nervous system due to alcohol |
| G621 | Alcoholic polyneuropathy |
| G721 | Alcoholic myopathy |
| I426 | Alcoholic cardiomyopathy |
| K292 | Alcoholic gastritis |
| K70 | Alcoholic liver disease |
| K700 | Alcoholic fatty liver |
| K701 | Alcoholic hepatitis |
| K702 | Alcoholic fibrosis and sclerosis of liver |
| K703 | Alcoholic cirrhosis of liver |
| K704 | Alcoholic hepatic failure |
| K709 | Alcoholic liver disease, unspecified |
| K852 | Alcohol-induced acute pancreatitis |
| K860 | Alcohol-induced chronic pancreatitis |
| O354 | Maternal care for (suspected) damage to fetus from alcohol |
| R780 | Finding of alcohol in blood |
| T51 | Toxic effect of alcohol |
| T510 | Toxic effect: Ethanol |
| T511 | Toxic effect: Methanol |
| T512 | Toxic effect: 2-Propanol |
| T513 | Toxic effect: Fusel oil |
| T518 | Toxic effect: Other alcohols |
| T519 | Toxic effect: Alcohol, unspecified |
| X45 | Accidental poisoning by and exposure to alcohol |
| X450 | Accidental poisoning by and exposure to alcohol |
| X451 | Accidental poisoning by and exposure to alcohol |
| X452 | Accidental poisoning by and exposure to alcohol |
| X453 | Accidental poisoning by and exposure to alcohol |
| X454 | Accidental poisoning by and exposure to alcohol |
| X455 | Accidental poisoning by and exposure to alcohol |
| X456 | Accidental poisoning by and exposure to alcohol |
| X457 | Accidental poisoning by and exposure to alcohol |
| X458 | Accidental poisoning by and exposure to alcohol |
| X459 | Accidental poisoning by and exposure to alcohol |
| X65 | Intentional self-poisoning by and exposure to alcohol |
| X650 | Intentional self-poisoning by and exposure to alcohol |
| X651 | Intentional self-poisoning by and exposure to alcohol |
| X652 | Intentional self-poisoning by and exposure to alcohol |
| X653 | Intentional self-poisoning by and exposure to alcohol |
| X654 | Intentional self-poisoning by and exposure to alcohol |
| X655 | Intentional self-poisoning by and exposure to alcohol |
| X656 | Intentional self-poisoning by and exposure to alcohol |
| X657 | Intentional self-poisoning by and exposure to alcohol |
| X658 | Intentional self-poisoning by and exposure to alcohol |
| X659 | Intentional self-poisoning by and exposure to alcohol |
| Y15 | Poisoning by and exposure to alcohol, undetermined intent |
| Y150 | Poisoning by and exposure to alcohol, undetermined intent |
| Y151 | Poisoning by and exposure to alcohol, undetermined intent |
| Y152 | Poisoning by and exposure to alcohol, undetermined intent |
| Y153 | Poisoning by and exposure to alcohol, undetermined intent |
| Y154 | Poisoning by and exposure to alcohol, undetermined intent |
| Y155 | Poisoning by and exposure to alcohol, undetermined intent |
| Y156 | Poisoning by and exposure to alcohol, undetermined intent |
| Y157 | Poisoning by and exposure to alcohol, undetermined intent |
| Y158 | Poisoning by and exposure to alcohol, undetermined intent |
| Y159 | Poisoning by and exposure to alcohol, undetermined intent |
| Y573 | Alcohol deterrents |
| Y900 | Blood alcohol level of less than 20 mg/100 ml |
| Y901 | Blood alcohol level of 20-39 mg/100 ml |
| Y902 | Blood alcohol level of 40-59 mg/100 ml |
| Y903 | Blood alcohol level of 60-79 mg/100 ml |
| Y904 | Blood alcohol level of 80-99 mg/100 ml |
| Y905 | Blood alcohol level of 100-119 mg/100 ml |
| Y906 | Blood alcohol level of 120-199 mg/100 ml |
| Y907 | Blood alcohol level of 200-239 mg/100 ml |
| Y908 | Blood alcohol level of 240 mg/100 ml or more |
| Y909 | Presence of alcohol in blood, level not specified |
| Y910 | Mild alcohol intoxication |
| Y911 | Moderate alcohol intoxication |
| Y912 | Severe alcohol intoxication |
| Y913 | Very severe alcohol intoxication |
| Y919 | Alcohol involvement, not otherwise specified |
| Z502 | Alcohol rehabilitation |
| Z714 | Alcohol abuse counselling and surveillance |
| Z721 | Alcohol use |

**Table 18:** **Substance misuse related health condition ICD10 codes**

| ICD10 | Description |
| --- | --- |
| F11 | Mental and behavioural disorders due to use of opioids |
| F110 | Mental & behav dis due to use of opiods; acute intoxication |
| F111 | Mental & behav dis due to use of opiods; harmful use |
| F112 | Mental & behav dis due to use of opiods; dependence syndrome |
| F113 | Mental & behav dis due to use of opiods; withdrawal state |
| F114 | Men & behav dis due opiods; withdrawl state with delirium |
| F115 | Mental & behav dis due to use of opiods; psychotic disorder |
| F116 | Mental & behav dis due to use of opiods; amnesic syndrome |
| F117 | Men& behav dis due use opiods; resid & late-ons psychot dis |
| F118 | Men & behav dis due to use of opiods; other men & behav dis |
| F119 | Mental & behav dis due use opiods; unsp mental & behav dis |
| F12 | Mental and behavioural disorders due to use of cannabinoids |
| F120 | Mental & behav dis due use cannabinoids; acute intoxication |
| F121 | Mental & behav dis due use cannabinoids; harmful use |
| F122 | Mental & behav dis due use cannabinoids; dependence syndrome |
| F123 | Mental & behav dis due use cannabinoids; withdrawal state |
| F124 | Men & beh dis due use cannabis; withdraw state with delirium |
| F125 | Mental & behav dis due use cannabinoids; psychotic disorder |
| F126 | Mental & behav dis due use cannabinoids; amnesic syndrome |
| F127 | Men & beh dis due cannabis; resid & late-onset psychot dis |
| F128 | Men & behav dis due use cannabinoids; oth men and behav dis |
| F129 | Mental & behav dis due cannabinoids; unsp mental & behav dis |
| F13 | Mental & behavioural disorders due use sedatives/hypnotics |
| F130 | Men & behav dis due use seds/hypnotics: acute intoxication |
| F131 | Men & behav dis due use seds/hypnotics: harmful use |
| F132 | Men & behav dis due use seds/hypnotics: dependence syndrome |
| F133 | Men & behav dis due use seds/hypnotics: withdrawal state |
| F134 | Men & beh dis due seds/hypns: withdrawl state with delirium |
| F135 | Men & behav dis due use seds/hypnotics: psychotic disorder |
| F136 | Men & behav dis due use seds/hypnotics: amnesic syndrome |
| F137 | Men & behav dis due seds/hypns: resid & late-ons psychot dis |
| F138 | Men & behav dis due seds/hypnotics: other men & behav dis |
| F139 | Men & behav dis due seds/hypnotics: unsp men & behav dis |
| F14 | Mental and behavioural disorders due to use of cocaine |
| F140 | Mental & behav dis due use cocaine: acute intoxication |
| F141 | Mental & behav dis due use cocaine: harmful use |
| F142 | Mental & behav dis due use cocaine: dependence syndrome |
| F143 | Mental & behav dis due use cocaine: withdrawal state |
| F144 | Men & behav dis due cocaine: withdrawl state with delirium |
| F145 | Mental & behav dis due use cocaine: psychotic disorder |
| F146 | Mental & behav dis due use cocaine: amnesic syndrome |
| F147 | Men & behav dis due use cocain: resid & late-ons psychot dis |
| F148 | Mental & behav dis due use cocaine: other mental & behav dis |
| F149 | Mental & behav dis due use cocaine: unsp mental & behav dis |
| F15 | Men & behav dis due use oth stims inc caffeine |
| F150 | Men & behav dis due oth stims inc cafein: acute intoxication |
| F151 | Men & behav dis due use oth stims inc caffeine: harmful use |
| F152 | Men & behav dis due oth stims inc caffeine: dependence synd |
| F153 | Men & behav dis due oth stims inc caffeine: withdrawal state |
| F154 | Men & beh dis due oth stims inc caff: withdraw stat + delir |
| F155 | Men & behav dis due oth stims inc caffeine: psychotic dis |
| F156 | Men & behav dis due oth stims inc caffeine: amnesic syndrome |
| F157 | Men&beh dis due oth stims inc caff:res & late-ons psych dis |
| F158 | Men & beh dis due oth stims inc caffeine:other men & beh dis |
| F159 | Men & beh dis due oth stims inc caffeine: unsp men & beh dis |
| F16 | Mental and behavioural disorders due to use of hallucinogens |
| F160 | Men & behav dis due use hallucinogens: acute intoxication |
| F161 | Men & behav dis due use hallucinogens: harmful use |
| F162 | Men & behav dis due use hallucinogens: dependence syndrome |
| F163 | Men & behav dis due use hallucinogens: withdrawal state |
| F164 | Men & behav dis due use hallucinogens: withdraw stat + delir |
| F165 | Men & behav dis due use hallucinogens: psychotic disorder |
| F166 | Men & behav dis due use hallucinogens: amnesic syndrome |
| F167 | Men & behav dis due use hallucins: res & late-ons psych dis |
| F168 | Men & behav dis due use hallucinogens: oth men & behav dis |
| F169 | Men & behav dis due use hallucinogens: unsp men & behav dis |
| F18 | Mental & behavioural disorders due use volatile solvents |
| F180 | Men & behav dis due use volatil solvs: acute intoxication |
| F181 | Men & behav dis due use volatil solvs: harmful use |
| F182 | Men & behav dis due use volatil solvs: dependence syndrome |
| F183 | Men & behav dis due use volatil solvs: withdrawl state |
| F184 | Men & behav dis due use volatil solvs: withdrawl stat + deli |
| F185 | Men & behav dis due use volatil solvs: psychotic disorder |
| F186 | Men & behav dis due use volatil solvs: amnesic syndrome |
| F187 | Men & behav dis due use volatil solvs: res & late-ons psych |
| F188 | Men & behav dis due use volatil solvs: oth men & behav dis |
| F189 | Men & behav dis due use volatil solvs: unsp men & behav dis |
| F19 | Mental & behav'l disorders due multiple/psychoact drug use |
| F190 | Men & behav dis multiple/psychoact drug: acute intox |
| F191 | Men & behav dis multiple/psychoact drug: harmful use |
| F192 | Men & behav dis multiple/psychoact drug: dependence syndrome |
| F193 | Men & behav dis multiple/psychoact drug: withdrawl state |
| F194 | Men & behav dis multiple/psychoact drug: withdrawl stat + de |
| F195 | Men & behav dis multiple/psychoact drug: psychotic disorder |
| F196 | Men & behav dis multiple/psychoact drug: amnesic syndrome |
| F197 | Men & behav dis multiple/psychoact drug: res & late-ons psy |
| F198 | Men & behav dis multiple/psychoact drug: oth men & behav dis |
| F199 | Men & behav dis multiple/psychoact drug: unsp men & behav di |
| O355 | Maternal care for (suspected) damage to fetus by drugs |
| R781 | Finding of opiate drug in blood |
| R782 | Finding of cocaine in blood |
| R783 | Finding of hallucinogen in blood |
| R784 | Finding of other drugs of addictive potential in blood |
| R785 | Finding of psychotropic drug in blood |
| T40 | Poisoning by narcotics and psychodysleptics [hallucinogens] |
| T400 | Poisoning by Opium |
| T401 | Poisoning by Heroin |
| T402 | Poisoning by other opioids |
| T403 | Poisoning by Methadone |
| T404 | Poisoning by other synthetic narcotics |
| T405 | Poisoning by Cocaine |
| T406 | Poisoning by other and unspecified narcotics |
| T407 | Poisoning by Cannabis (derivatives) |
| T408 | Poisoning by Lysergide [LSD] |
| T409 | Poisoning by other & unspec psychodysleptics [hallucinogens] |
| T436 | Poisoning by psychostimulants with abuse potential |
| X42 | Acc pois/expos narcotics & psychodysleptics [halluc'ns] NEC |
| X420 | Occurrence at home |
| X421 | Occurrence in residential institution |
| X422 | Occurrence at school other instit'n / pub admin area |
| X423 | Occurrence at sports / athletics area |
| X424 | Occurrence on street / highway |
| X425 | Occurrence at trade / service area |
| X426 | Occurrence at industrial / construction area |
| X427 | Occurrence on farm |
| X428 | Occurrence at other specified place |
| X429 | Occurrence at unspecified place |
| X62 | Inten sf pois/expos narcots & psy'dysleptics [halluc'ns]NEC |
| X620 | Occurrence at home |
| X621 | Occurrence in residential institution |
| X622 | Occurrence at school other instit'n / pub admin area |
| X623 | Occurrence at sports / athletics area |
| X624 | Occurrence on street / highway |
| X625 | Occurrence at trade / service area |
| X626 | Occurrence at industrial / construction area |
| X627 | Occurrence on farm |
| X628 | Occurrence at other specified place |
| X629 | Occurrence at unspecified place |
| Y12 | Pois/expos narcotics & psy'dys'tics [halluc'ns] undet intent |
| Y120 | Occurrence at home |
| Y121 | Occurrence in residential institution |
| Y122 | Occurrence at school other instit'n / pub admin area |
| Y123 | Occurrence at sports / athletics area |
| Y124 | Occurrence on street / highway |
| Y125 | Occurrence at trade / service area |
| Y126 | Occurrence at industrial / construction area |
| Y127 | Occurrence on farm |
| Y128 | Occurrence at other specified place |
| Y129 | Occurrence at unspecified place |
| Z503 | Drug rehabilitation |
| Z715 | Drug abuse counselling and surveillance |
| Z722 | Drug use |

**Table 19: Substance misuse related health condition READ codes**

| READ | Description |
| --- | --- |
| 13c.. | Drug user |
| 13c0. | Injecting drug user |
| 13c1. | Intravenous drug user |
| 13c2. | Never injecting drug user |
| 13c3. | Intramuscular drug user |
| 13c4. | Intranasal drug user |
| 13c5. | Substance misuse increased |
| 13c6. | Substance misuse decreased |
| 13c7. | Current drug user |
| 13c8. | Reduced drugs misuse |
| 13c9. | Subcutaneous drug user |
| 13cA. | Smokes drugs |
| 13cB. | Misuses drugs orally |
| 13cC. | Continuous use of drugs |
| 13cD. | Episodic use of drugs |
| 13cE. | Prolong high dose use cannabis |
| 13cF. | Preoccup with substance misuse |
| 13cG. | Drug tolerance |
| 13cG0 | Opioid tolerant |
| 13cG1 | Opioid naive |
| 13cH. | Persistent substance misuse |
| 13cJ. | Previously injecting drug user |
| 13cK. | Current non recreat drug user |
| 13cL. | Has never injected drugs |
| 13cM. | Substance misuse |
| 13cM0 | Novl psychactve sbstnce misuse |
| 13cM1 | Opioid analgesic dependence |
| 13cN. | Has nvr shrd drg injctn equipt |
| 13cQ. | Behavioural tolerance to drug |
| 13cR. | Physical tolerance to drug |
| 13cS. | Psychological drug tolerance |
| 13cT. | Reverse tolerance to drug |
| 1463 | H/O: drug dependency |
| 146C. | Failed heroin detoxification |
| 146E. | H/O: recreational drug use |
| 146F. | H/O: drug abuse |
| 1P30. | Compul uncontrollable drug tak |
| 1P31. | Compulsive drug taking |
| 1T... | History of substance misuse |
| 1T0.. | H/O heroin misuse |
| 1T00. | H/O daily heroin misuse |
| 1T01. | H/O weekly heroin misuse |
| 1T02. | Prev history of heroin misuse |
| 1T03. | H/O infrequent heroin misuse |
| 1T1.. | H/O methadone misuse |
| 1T10. | H/O daily methadone misuse |
| 1T11. | H/O weekly methadone misuse |
| 1T12. | H/O infrequent methadone misus |
| 1T13. | Prev history methadone misuse |
| 1T2.. | H/O ecstasy misuse |
| 1T20. | H/O daily ecstasy misuse |
| 1T21. | H/O weekly ecstasy misuse |
| 1T22. | H/O infrequent ecstasy misuse |
| 1T23. | Prev history of ecstasy misuse |
| 1T3.. | H/O benzodiazepine misuse |
| 1T30. | H/O daily benzodiazepin misuse |
| 1T31. | H/O weekly benzodiazep misuse |
| 1T32. | H/O infreq benzodiazep misuse |
| 1T33. | Prev H/O benzodiazepine misuse |
| 1T4.. | H/O amphetamine misuse |
| 1T40. | H/O daily amphetamine misuse |
| 1T41. | H/O weekly amphetamine misuse |
| 1T42. | H/O infrequent amphetam misuse |
| 1T43. | Prev H/O amphetamine misuse |
| 1T5.. | H/O cocaine misuse |
| 1T50. | H/O daily cocaine misuse |
| 1T51. | H/O weekly cocaine misuse |
| 1T52. | H/O infrequent cocaine misuse |
| 1T53. | Prev H/O cocaine misuse |
| 1T6.. | H/O crack cocaine misuse |
| 1T60. | H/O daily crack cocaine misuse |
| 1T61. | H/O weekly crack cocain misuse |
| 1T62. | H/O infrequ crack cocain misus |
| 1T63. | Prev H/O crack cocaine misuse |
| 1T7.. | H/O hallucinogen misuse |
| 1T70. | H/O daily hallucinogen misuse |
| 1T71. | H/O weekly hallucinogen misuse |
| 1T72. | H/O infrequ hallucinog misuse |
| 1T73. | Prev H/O hallucinogen misuse |
| 1T8.. | H/O cannabis misuse |
| 1T80. | H/O daily cannabis misuse |
| 1T81. | H/O weekly cannabis misuse |
| 1T82. | H/O infrequent cannabis misuse |
| 1T83. | Prev H/O cannabis misuse |
| 1T9.. | H/O solvent misuse |
| 1T90. | H/O daily solvent misuse |
| 1T91. | H/O weekly solvent misuse |
| 1T92. | H/O infrequent solvent misuse |
| 1T93. | Prev history of solvent misuse |
| 1TA.. | H/O barbiturate misuse |
| 1TA0. | H/O daily barbiturate misuse |
| 1TA1. | H/O weekly barbiturate misuse |
| 1TA2. | H/O infrequ barbiturate misuse |
| 1TA3. | Prev H/O barbiturate misuse |
| 1TB.. | H/O major tranquilliser misuse |
| 1TB0. | H/O daily maj tranquilli misus |
| 1TB1. | H/O weekl maj tranquilli misus |
| 1TB2. | H/O infreq maj trnquillis miss |
| 1TB3. | Prev H/O major tranq misuse |
| 1TC.. | H/O anti-depressant misuse |
| 1TC0. | H/O daily anti-depress misuse |
| 1TC1. | H/O weekly anti-depress misuse |
| 1TC2. | H/O infreq anti-depress misuse |
| 1TC3. | Prev H/O anti-depressnt misuse |
| 1TD.. | H/O opiate misuse |
| 1TD0. | H/O daily opiate misuse |
| 1TD1. | H/O weekly opiate misuse |
| 1TD2. | H/O infrequent opiate misuse |
| 1TD3. | Prev history of opiate misuse |
| 1TE.. | Uses heroin on top subst ther |
| 1TF.. | Dsnt use heroin top subst ther |
| 1TG.. | H/O nov psychoact subst misuse |
| 1V... | Drug misuse behaviour |
| 1V0.. | Misuses drugs |
| 1V00. | Occasional drug user |
| 1V01. | Long-term drug misuser |
| 1V02. | Poly-drug misuser |
| 1V03. | Misuses drugs sublingually |
| 1V04. | Misuses drugs rectally |
| 1V05. | Misuses drugs vaginally |
| 1V06. | Uses drug paraphernalia |
| 1V07. | Notified addict |
| 1V08. | Smokes drugs in cigarette form |
| 1V09. | Smokes drugs through a pipe |
| 1V0A. | Chases the dragon |
| 1V0B. | Sniffs drugs |
| 1V0C. | Drug addict |
| 1V0D. | Am spent per day on drug habit |
| 1V0E. | Health prob sec to drug misuse |
| 1V1.. | Time devotd drug rel activties |
| 1V10. | Time spent obtaining drugs |
| 1V11. | Time spent taking drugs |
| 1V12. | Time spent recover from drugs |
| 1V2.. | Frequency of drug misuse |
| 1V22. | Age at starting drug misuse |
| 1V23. | Time since stopped drug misuse |
| 1V24. | Total time drugs misused |
| 1V26. | Misused drugs in past |
| 1V3.. | Drug injection behaviour |
| 1V30. | Injects drugs subcutaneously |
| 1V31. | Injects drugs intramuscularly |
| 1V32. | Neck injector |
| 1V33. | Groin injector |
| 1V34. | Does not inject drugs |
| 1V35. | Shares drug equipment |
| 1V36. | Frontloading |
| 1V37. | Drug inject equipment hygiene |
| 1V38. | Sharing drug inject equipment |
| 1V3A. | Not share drug inject equipmen |
| 1V3B. | Shares syringes |
| 1V3C. | Shares needles |
| 1V3D. | Cleaning of needles |
| 1V3E. | Cleans own needles |
| 1V3F. | Cleans needles with bleach |
| 1V3G. | Does not clean needles |
| 1V3H. | Obtains clean needles |
| 1V3J. | Uses needle exchange scheme |
| 1V3K. | Obtains clean syringes |
| 1V3L. | Needle syringe exch scheme use |
| 1V3M. | Needle + syringe exch not used |
| 1V3N. | Needle and syringe exch used |
| 1V4.. | Priority of drug activity |
| 1V40. | No priority to drug activities |
| 1V41. | Priority to drug activities |
| 1V42. | Drug priority ov social obligs |
| 1V43. | Drug priority over family |
| 1V44. | Drug priority ov finance oblig |
| 1V5.. | Routine drug-related activity |
| 1V50. | No routine of drug activities |
| 1V51. | Has routine of drug activities |
| 1V52. | Same drug routine every day |
| 1V53. | Drug-related rituals |
| 1V54. | Follows drug-related rituals |
| 1V55. | Not follow drug-relate rituals |
| 1V6.. | Drug-relat offending behaviour |
| 1V64. | Illicit drug use |
| 1V65. | Heroin misuse |
| 1V66. | Ecstasy misuse |
| 677T. | Subst misuse structurd counsel |
| 7P220 | Delivery rehab drug addiction |
| 8AA.. | Drug abuse monitoring |
| 8B23. | Drug addiction therapy |
| 8B230 | Drug add maint ther naltrexone |
| 8B231 | Drug add maint ther lofexidine |
| 8B2M. | Buprenorphine maintenance ther |
| 8B2N. | Drug add detox ther methadone |
| 8B2P. | Drug add maint ther methadone |
| 8B2Q. | Drug add maint ther buprenorph |
| 8B2R. | Drug add detox ther buprenorph |
| 8B2S. | Opioid agonist substitut thera |
| 8B2T. | Opioid antagonist therapy |
| 8BA9. | Detoxification dependence drug |
| 8BAW. | Drug depen self detoxification |
| 8BAX. | Drug depen home detoxification |
| 8BAc. | Subs mis mgt stop - self withd |
| 8BAd. | Opiate dependence detoxificatn |
| 8BAt. | Drug relapse prevention |
| 8BAv. | Drug harm reduction programme |
| 8BAx. | Drug twelve step programme |
| 8BE.. | Maintenance therapy |
| 8BE0. | Reinduct methadone maint thera |
| 8BE1. | Reinduct buprenorph maint ther |
| 8CR9. | Benzodiazepi clinical mgt plan |
| 8FB.. | Drug rehabilitation |
| 8FB0. | Drug detox programme completed |
| 8H7x. | Refer to drug abuse counsellor |
| 8HHL. | Ref to comm drug dependen team |
| 8HHd. | Referral to drug treatment cen |
| 8HHe. | Referral to com drug alco team |
| 8Hh1. | Self refer substanc misus serv |
| 8HkF. | Refer substance misuse service |
| 8Hl5. | Referral to drugs therapist |
| 8Hl6. | Referral to drugs worker |
| 8Hq.. | Admsn substnc misuse detox cnt |
| 8I2N. | Drug depend home detox contra |
| 8IE7. | Substance misuse assess declin |
| 9G2.. | Drug addiction notification |
| 9G21. | Drug addict notific to CMO |
| 9G22. | Drug addict re-notific due |
| 9G23. | Drug addict re-notif to CMO |
| 9G2Z. | Drug addiction notif NOS |
| 9HC.. | Substance misuse monitoring |
| 9HC0. | Initial substance misuse asses |
| 9HC1. | Follow up substa misuse assess |
| 9HC2. | Subst mis clin man plan agreed |
| 9HC3. | Subst mis clin man plan review |
| 9HC4. | Sub misuse treatment withdrawn |
| 9HC5. | Sub misus treat prog completed |
| 9HC6. | Substance misuse treatm declin |
| 9HC7. | Subst misuse treat not availbl |
| 9HC8. | Decl to give subst misuse hist |
| 9HC9. | Snc mse tmnt gvn othr hcr prdr |
| 9HCA. | Sbstnce misuse mntr 6 mnth rvw |
| 9HCB. | Substance misuse mntr annl rvw |
| 9HCC. | On substance misuse programme |
| 9N0Z. | Seen in drug rehab centre |
| 9N1yJ | Seen in drug misuse clinic |
| 9N6a. | Refer by drug statutor service |
| 9N6b. | Ref by drug non-statutory serv |
| 9N6g. | Refer by syringe excha service |
| 9NN1. | Under care community drug team |
| 9NX2. | In-house subs misuse treatment |
| 9NdN. | Declnd consnt notif drug misus |
| 9No5. | Seen in substance misuse clinc |
| 9k5.. | Drug misuse - enhan serv admin |
| 9k50. | Drug misuse - enh serv complet |
| 9k51. | Share care drug misu trt - ESA |
| 9k52. | Drug misus trt prim care - ESA |
| 9k53. | Phrmcy attend drug misus - ESA |
| 9kS.. | Drug mis asse decl - enha serv |
| E02.. | Drug psychoses |
| E020. | Drug withdrawal syndrome |
| E021. | Drug-induced paranoia/hallucin |
| E0210 | Drug-induced paranoid state |
| E0211 | Drug-induced hallucinosis |
| E021z | Drug-induc.paranoia/halluc NOS |
| E022. | Pathological drug intoxication |
| E02y. | Other drug psychoses |
| E02y0 | Drug-induced delirium |
| E02y1 | Drug-induced dementia |
| E02y2 | Drug-induced amnestic syndrome |
| E02y3 | Drug-induced depressive state |
| E02y4 | Drug-induced personality dis. |
| E02yz | Other drug psychoses NOS |
| E02z. | Drug psychosis NOS |
| E24.. | Drug dependence |
| E240. | Opioid type drug dependence |
| E2400 | Opioid dependence-unspecified |
| E2401 | Opioid dependence-continuous |
| E2402 | Opioid dependence - episodic |
| E2403 | Opioid dependence-in remission |
| E240z | Opioid drug dependence NOS |
| E241. | Hypnotic/anxiolytic dependence |
| E2410 | Hypnotic/anxiol.depend.-unspec |
| E2411 | Hypnot/anxiol.dep.-continuous |
| E2412 | Hypnot/anxiol.dep.-episodic |
| E2413 | Hypnot/anxiol.dep-in remission |
| E241z | Hypnotic/anxiolytic depend.NOS |
| E242. | Cocaine type drug dependence |
| E2420 | Cocaine dependence-unspecified |
| E2421 | Cocaine dependence-continuous |
| E2422 | Cocaine dependence-episodic |
| E2423 | Cocaine depend. - in remission |
| E242z | Cocaine drug dependence NOS |
| E243. | Cannabis type drug dependence |
| E2430 | Cannabis dependence-unspecif. |
| E2431 | Cannabis dependence-continuous |
| E2432 | Cannabis dependence-episodic |
| E2433 | Cannabis depend.- in remission |
| E243z | Cannabis drug dependence NOS |
| E244. | Amphetamine/psychostim.depend. |
| E2440 | Amphetamine depend.-unspecif. |
| E2441 | Amphetamine depend.-continuous |
| E2442 | Amphetamine depend.-episodic |
| E2443 | Amphetamine dep.-in remission |
| E244z | Amphetamine dependence NOS |
| E245. | Hallucinogen dependence |
| E2450 | Hallucinogen depend.-unspecif. |
| E2451 | Hallucinogen depend-continuous |
| E2452 | Hallucinogen depend.-episodic |
| E2453 | Hallucinogen dep.-in remission |
| E245z | Hallucinogen dependence NOS |
| E246. | Glue sniffing dependence |
| E2460 | Glue sniffing - unspecified |
| E2461 | Glue sniffing - continuous |
| E2462 | Glue sniffing - episodic |
| E2463 | Glue sniffing - in remission |
| E246z | Glue sniffing dependence NOS |
| E247. | Other specified drug dependen. |
| E2470 | Other drug dependence unspecif |
| E2471 | Other drug depend.-continuous |
| E2472 | Other drug depend.-episodic |
| E2473 | Other drug dep.-in remission |
| E247z | Other drug dependence NOS |
| E248. | Combined opioid+other drug dep |
| E2480 | Opioid+other drug dep. unspec. |
| E2481 | Continuous opioid+other depen. |
| E2482 | Episodic opioid+other depend. |
| E2483 | In remission-opioid+other dep. |
| E248z | Opioid+other drug depend. NOS |
| E249. | Combined drug dep. excl.opioid |
| E2490 | Comb.drug dep ex opioid-unspec |
| E2491 | Comb.drug dep ex opioid-contin |
| E2492 | Comb.drug dep ex opioid-episod |
| E2493 | Comb.drug dep ex opioid-in rem |
| E249z | Comb.drug dep ex opioid NOS |
| E24A. | Ecstasy type drug dependence |
| E24z. | Drug dependence NOS |
| E25.. | Nondependent abuse of drugs |
| E252. | Nondependent cannabis abuse |
| E2520 | Nondep cannabis abuse - unspec |
| E2521 | Nondep cannabis abuse - contin |
| E2522 | Nondep cannabis abuse - episod |
| E2523 | Nondep cannabis abuse in remis |
| E252z | Nondep cannabis abuse NOS |
| E253. | Nondependen hallucinogen abuse |
| E2530 | Nondep hallucinogen abuse-unsp |
| E2531 | Nondep hallucinogen abuse-cont |
| E2532 | Nondep hallucinogen abuse-epis |
| E2533 | Nondep hallucin abuse-in remis |
| E253z | Nondep hallucinogen abuse NOS |
| E254. | Nondep hypnot/anxiolytic abuse |
| E2540 | Nondep hypnot/anxio.abuse-unsp |
| E2541 | Nondep hypnot/anxio.abuse-cont |
| E2542 | Nondep hypnot/anxio.abuse-epis |
| E2543 | Nondep hypn/anxio.abuse-in rem |
| E254z | Nondep hypnot/anxiol abuse NOS |
| E255. | Nondependent opioid abuse |
| E2550 | Nondep opioid abuse - unspecif |
| E2551 | Nondep opioid abuse - continuo |
| E2552 | Nondep opioid abuse - episodic |
| E2553 | Nondep opioid abuse - in remis |
| E255z | Nondependent opioid abuse NOS |
| E256. | Nondependent cocaine abuse |
| E2560 | Nondep cocaine abuse - unspec. |
| E2561 | Nondep cocaine abuse - contin. |
| E2562 | Nondep cocaine abuse - episod. |
| E2563 | Nondep cocaine abuse -in remis |
| E256z | Nondependent cocaine abuse NOS |
| E257. | Nondep amphetamine type abuse |
| E2570 | Nondep amphet type abuse -unsp |
| E2571 | Nondep amphet type abuse -cont |
| E2572 | Nondep amphet type abuse -epis |
| E2573 | Nondep amph. type abuse-in rem |
| E257z | Nondep amphet. type abuse NOS |
| E258. | Nondep antidepress type abuse |
| E2580 | Nondep antidep type abuse-unsp |
| E2581 | Nondep antidep type abuse-cont |
| E2582 | Nondep antidep type abuse-epis |
| E2583 | Nondep antidep tp abuse-in rem |
| E258z | Nondep antidep type abuse NOS |
| E259. | Nondependent mixed drug abuse |
| E2590 | Nondep mixed drug abuse-unspec |
| E2591 | Nondep mixed drug abuse-contin |
| E2592 | Nondep mixed drug abuse-episod |
| E2593 | Nondep mixed drug abuse-in rem |
| E2594 | Misuse of prescription drugs |
| E259z | Nondep mixed drug abuse NOS |
| E25y. | Nondependent other drug abuse |
| E25y0 | Nondep other drug abuse-unspec |
| E25y1 | Nondep other drug abuse-contin |
| E25y2 | Nondep other drug abuse-episod |
| E25y3 | Nondep other drug abuse-in rem |
| E25yz | Nondep other drug abuse NOS |
| E25z. | Misuse of drugs NOS |
| Eu1.. | [X]Mental dis, psychoact subst |
| Eu11. | [X]Mental dis due to opioids |
| Eu110 | [X]Acute opioid intoxication |
| Eu111 | [X]Harmful use of opioids |
| Eu112 | [X]Opioid dependence syndrome |
| Eu113 | [X]Opioid withthdrawal state |
| Eu114 | [X]Opioid withdrawal delirium |
| Eu115 | [X]Psychot dis due to opioids |
| Eu116 | [X]Amnesic synd due to opioids |
| Eu117 | [X]Resid psychotic due opioid |
| Eu11y | [X]Oth ment/beh dis due opioid |
| Eu11z | [X]Uns ment/beh dis due opioid |
| Eu12. | [X]Mental dis due cannabinoids |
| Eu120 | [X]Acute cannabis intoxication |
| Eu121 | [X]Harmful use of cannabis |
| Eu122 | [X]Cannabis dependence syndrom |
| Eu123 | [X]Cannabis withdrawal state |
| Eu124 | [X]Cannabis withdrawl delirium |
| Eu125 | [X]Psychot dis due to cannabis |
| Eu126 | [X]Amnesic synd due cannabis |
| Eu127 | [X]Resid psychot due cannabis |
| Eu12y | [X]Oth ment/beh dis cannabinds |
| Eu12z | [X]Unsp mnt/beh dis cannabinds |
| Eu13. | [X]Mental dis due sedat/hypnot |
| Eu130 | [X]Acute sedat/hypnotic intox |
| Eu131 | [X]Harmful use sedat/hypnotic |
| Eu132 | [X]Sedat/hypnotic depend syndr |
| Eu133 | [X]Sedat/hypnot withdraw state |
| Eu134 | [X]Sed/hypn withdraw delirium |
| Eu135 | [X]Psychot dis due sedat/hypn |
| Eu136 | [X]Amnesic synd due sedat/hypn |
| Eu137 | [X]Resid psychot due sed/hypn |
| Eu13y | [X]Oth ment/beh dis sed/hypnot |
| Eu13z | [X]Uns ment/beh dis sed/hypnot |
| Eu14. | [X]Mental dis due use cocaine |
| Eu140 | [X]Acute cocaine intoxication |
| Eu141 | [X]Harmful use of cocaine |
| Eu142 | [X]Cocaine dependence syndrome |
| Eu143 | [X]Cocaine withdrawal state |
| Eu144 | [X]Cocaine withdrawal delirium |
| Eu145 | [X]Psychot dis due to cocaine |
| Eu146 | [X]Amnesic synd due to cocaine |
| Eu147 | [X]Resid psychot due cocaine |
| Eu14y | [X]Ot ment/beh dis due cocaine |
| Eu14z | [X]Uns ment/bh dis due cocaine |
| Eu15. | [X]Ment dis oth stimul/caffein |
| Eu150 | [X]Acute intoxic oth stimulant |
| Eu151 | [X]Harmful use other stimulant |
| Eu152 | [X]Oth stimulant dependen synd |
| Eu153 | [X]Oth stimulant withdr state |
| Eu154 | [X]Oth stimulant withdr delir |
| Eu155 | [X]Psychotic dis oth stimulant |
| Eu156 | [X]Amnesic syndr oth stimulant |
| Eu157 | [X]Resid psychot oth stimulant |
| Eu15y | [X]Oth ment/beh dis stimulant |
| Eu15z | [X]Uns ment/beh dis stimulant |
| Eu16. | [X]Mental disord hallucinogens |
| Eu160 | [X]Acute hallucinogen intoxic |
| Eu161 | [X]Harmful use hallucinogens |
| Eu162 | [X]Hallucinogen depend synd |
| Eu163 | [X]Hallucinogen withdraw state |
| Eu164 | [X]Hallucin withdraw delirium |
| Eu165 | [X]Psychotic due hallucinogen |
| Eu166 | [X]Amnesic synd due hallucinog |
| Eu167 | [X]Resid psychot hallucinogen |
| Eu16y | [X]Oth ment/beh dis hallucinog |
| Eu16z | [X]Uns ment/beh dis hallucinog |
| Eu18. | [X]Ment dis volatile solvents |
| Eu180 | [X]Acute solvent intoxication |
| Eu181 | [X]Harmful use of solvents |
| Eu182 | [X]Solvent dependence syndrome |
| Eu183 | [X]Solvent withdrawal state |
| Eu184 | [X]Solvent withdrawal delirium |
| Eu185 | [X]Psychotic dis due solvent |
| Eu186 | [X]Amnesic syndr due solvent |
| Eu187 | [X]Resid psychotic due solvent |
| Eu18y | [X]Ot ment/beh dis due solvent |
| Eu18z | [X]Uns ment/beh due solvent |
| Eu19. | [X]Ment disord multi drug use |
| Eu190 | [X]Acute intox multi drug use |
| Eu191 | [X]Harmful use multiple drugs |
| Eu192 | [X]Multiple drug dependence |
| Eu193 | [X]Multiple drug withdrawal |
| Eu194 | [X]Multi drug withdr delirium |
| Eu195 | [X]Psychotic due multi drugs |
| Eu196 | [X]Amnesic syn due multi drugs |
| Eu197 | [X]Resid psychotic multi drugs |
| Eu19y | [X]Ot ment/beh due multi drugs |
| Eu19z | [X]Un ment/beh due multi drugs |
| Eu1A. | [X]Men behav dis due crack coc |
| Eu1A0 | [X]Acute crack cocaine intoxic |
| Eu1A1 | [X]Harmful use crack cocaine |
| Eu1A2 | [X]Crack cocaine depend synd |
| Eu1A3 | [X]Crack cocaine withdraw stat |
| Eu1A4 | [X]Crack coc withdraw stat del |
| Eu1A5 | [X]Crack cocaine psychotic dis |
| Eu1A6 | [X]Crack cocaine amnesic synd |
| Eu1A7 | [X]Cra coc res late-on psy dis |
| Eu1Ay | [X]Crac coc other ment beh dis |
| Eu1Az | [X]Crack coc unsp men beh dis |
| L183. | Pregnancy+drug dependence |
| L1830 | Preg.+drug dependence unspecif |
| L1831 | Preg.+drug dependence-deliver. |
| L1832 | Preg.+drug depend-del+p/n comp |
| L1833 | Preg.+drug depend-not deliver. |
| L1834 | Preg.+drug depend.+p/n complic |
| L183z | Preg.+drug dependence NOS |
| L255. | Fetus+drug damage |
| L2550 | Fetus+drug damage unspecified |
| L2551 | Fetus+drug damage-delivered |
| L2552 | Fetus+drug damage+a/n problem |
| L255z | Fetus+drug damage NOS |
| R10B0 | [D]Finding of cocain in blood |
| R10B1 | [D]Find hallucinogen in blood |
| R10B2 | [D]Find psychotrop drug blood |
| R10B4 | [D]Finding, opiate drug in bld |
| Ryu86 | [X]Find ot drg addic poten,bld |
| SL50. | Opiate/narcotic poisoning |
| SL500 | Unspecified opium poisoning |
| SL501 | Heroin poisoning |
| SL502 | Methadone poisoning |
| SL50z | Opiate/narcotic poisoning NOS |
| SL850 | Cocaine poisoning |
| SL96. | Hallucinogen poisoning |
| SL960 | Cannabis poisoning |
| SL961 | Lysergide (LSD) poisoning |
| SL963 | Mescaline poisoning |
| SL964 | Psilocybin poisoning |
| SL96z | Hallucinogen poisoning NOS |
| SL97. | Psychostimulant poisoning |
| SL970 | Amfetamine poisoning |
| SL972 | Ecstasy poisoning |
| SL97z | Psychostimulant poisoning NOS |
| SyuFB | [X]Poisoning by other opioids |
| SyuFC | [X]Poisoning by oth synth narc |
| SyuFD | [X]Poisoning by oth/unsp narc |
| SyuFE | [X]Pois,oth/un psychodysl/hall |
| T800. | Accid.pois.- heroin |
| T801. | Accid.pois.- methadone |
| T8023 | Accid.pois.- opium |
| T841. | Accid.pois.- hallucinogens |
| T8410 | Accid.pois.- cannabis derivat. |
| T8413 | Accid.pois.- mescaline |
| T8414 | Accid.pois.- psilocin |
| T8415 | Accid.pois.- psilocybin |
| T842. | Accid.pois.- psychostimulants |
| T8420 | Accid.pois.- amphetamine |
| T8520 | Accid.pois.- cocaine |
| U1A5. | [X]Accident poisoning narcotic |
| U1A50 | [X]Acc poison narcotic home |
| U1A51 | [X]Ac pois narcotic res ins |
| U1A52 | [X]Ac pois narcotic pub ins |
| U1A53 | [X]Ac pois narcotic sport ar |
| U1A54 | [X]Ac pois narcotic on hway |
| U1A55 | [X]Ac pois narcotic trade ar |
| U1A56 | [X]Ac pois narcotic indus ar |
| U1A57 | [X]Ac pois narcotic on farm |
| U1A5y | [X]Ac pois narcotic OS place |
| U1A5z | [X]Ac pois narcotic unsp pl |
| U1A6. | [X]Acc poisoning hallucinogens |
| U1A60 | [X]Acc poison hallucinog home |
| U1A61 | [X]Ac pois hallucinog res ins |
| U1A62 | [X]Ac pois hallucinog pub ins |
| U1A63 | [X]Ac pois hallucinog sport ar |
| U1A64 | [X]Ac pois hallucinog on hway |
| U1A65 | [X]Ac pois hallucinog trade ar |
| U1A66 | [X]Ac pois hallucinog indus ar |
| U1A67 | [X]Ac pois hallucinog on farm |
| U1A6y | [X]Ac pois hallucinog OS place |
| U1A6z | [X]Ac pois hallucinog unsp pl |
| U205. | [X]Intent self poison narcotic |
| U2050 | [X]Self pois narcotic home |
| U2051 | [X]S/pois narcotic res ins |
| U2052 | [X]S/pois narcotic pub ins |
| U2053 | [X]S/pois narcotic sport ar |
| U2054 | [X]S/pois narcotic on hway |
| U2055 | [X]S/pois narcotic trade ar |
| U2056 | [X]S/pois narcotic indus ar |
| U2057 | [X]S/pois narcotic on farm |
| U205y | [X]S/pois narcotic OS place |
| U205z | [X]S/pois narcotic unsp pl |
| U206. | [X]Int s/poising hallucinogens |
| U2060 | [X]Self pois hallucinog home |
| U2061 | [X]S/pois hallucinog res ins |
| U2062 | [X]S/pois hallucinog pub ins |
| U2063 | [X]S/pois hallucinog sport ar |
| U2064 | [X]S/pois hallucinog on hway |
| U2065 | [X]S/pois hallucinog trade ar |
| U2066 | [X]S/pois hallucinog indus ar |
| U2067 | [X]S/pois hallucinog on farm |
| U206y | [X]S/pois hallucinog OS place |
| U206z | [X]S/pois hallucinog unsp pl |
| U405. | [X]Poisoning ?intent narcotic |
| U4050 | [X]Pois ?intent narcotic home |
| U4051 | [X]Pois ?int narcotic resid |
| U4052 | [X]Pois ?int narcotic pub ins |
| U4053 | [X]Pois ?int narcotic sport ar |
| U4054 | [X]Pois ?int narcotic on hway |
| U4055 | [X]Pois ?int narcotic trade ar |
| U4056 | [X]Pois ?int narcotic indus ar |
| U4057 | [X]Pois ?int narcotic on farm |
| U405y | [X]Pois ?int narcotic OS place |
| U405z | [X]Pois ?int narcotic unsp pl |
| U406. | [X]Poison ?intent hallucinogen |
| U4060 | [X]Pois ?intent hallucin home |
| U4061 | [X]Pois ?int hallucinog resid |
| U4062 | [X]Pois ?int hallucin pub ins |
| U4063 | [X]Pois ?int hallucin sport ar |
| U4064 | [X]Pois ?int hallucin on hway |
| U4065 | [X]Pois ?int hallucin trade ar |
| U4066 | [X]Pois ?int hallucin indus ar |
| U4067 | [X]Pois ?int hallucin on farm |
| U406y | [X]Pois ?int hallucin OS place |
| U406z | [X]Pois ?int hallucin unsp pl |
| ZV114 | [V]Pers hist subst abuse |
| ZV4K1 | [V]Drug use |
| ZV6D7 | [V]Drug abuse counsel+surveiln |
| dj36. | SUBUTEX 400micrograms s/l tabs |
| dj37. | SUBUTEX 2mg sublingual tablets |
| dj38. | SUBUTEX 8mg sublingual tablets |
| dj3D. | BUPRNRPHNE+NALOXN 2/0.5mg tabs |
| dj3E. | SUBOXONE 2mg/0.5mg s/l tabs |
| dj3F. | BUPRNRPHNE+NALOXN 8mg/2mg tabs |
| dj3G. | SUBOXONE 8mg/2mg s/l tabs |
| dj3K. | NATZON 400micrograms s/l tabs |
| dj3L. | NATZON 2mg sublingual tablets |
| dj3M. | NATZON 8mg sublingual tablets |
| dj3N. | GABUP 400micrograms s/l tabs |
| dj3O. | GABUP 1mg sublingual tablets |
| dj3P. | GABUP 2mg sublingual tablets |
| dj3Q. | GABUP 4mg sublingual tablets |
| dj3R. | GABUP 6mg sublingual tablets |
| dj3S. | GABUP 8mg sublingual tablets |
| dj3T. | BUPRENORPHINE 1mg s/l tabs |
| dj3U. | BUPRENORPHINE 4mg s/l tabs |
| dj3V. | BUPRENORPHINE 6mg s/l tabs |
| dj3c. | PREFIBIN 400mcg sublingual tab |
| dj3d. | PREFIBIN 2mg sublingual tabs |
| dj3e. | PREFIBIN 8mg sublingual tabs |
| dj3u. | BUPRENORPHINE 2mg s/l tabs |
| dj3v. | BUPRENORPHINE 8mg s/l tabs |
| djc.. | METHADONE HCL [ANALGESIC] |
| djc1. | PHYSEPTONE 5mg tablets |
| djc2. | PHYSEPTONE 10mg/1mL injection |
| djc3. | METHADONE 1mg/1mL mixture |
| djc4. | METHADONE HCL 50mg/5mL s/f liq |
| djc5. | MARTINDALE METHADONE DTF mixt |
| djc6. | METHODEX 1mg/1mL mixture |
| djc7. | METHADOSE 10mg/mL s/f liq |
| djc8. | METHADONE HCL 20mg/mL s/f liq |
| djc9. | METHADOSE 20mg/mL s/f liq |
| djcA. | METHADONE DILUENT liquid |
| djcB. | METHADOSE DILUENT liquid |
| djcC. | METHADONE 1mg/1mL s/f mixt |
| djcD. | METHAROSE 1mg/1mL s/f soln |
| djcE. | *PINADONE 1mg/1mL mixture |
| djcF. | *PINADONE 1mg/1mL s/f mixt |
| djcG. | PHYSEPTONE 20mg/2mL injection |
| djcH. | PHYSEPTONE 35mg/3.5mL inj |
| djcJ. | PHYSEPTONE 50mg/5mL injection |
| djcK. | PHYSEPTONE 1mg/1mL s/f mixture |
| djcL. | PHYSEPTONE 1mg/1mL mixture |
| djcM. | SYNASTONE 10mg/1mL injection |
| djcN. | SYNASTONE 20mg/2mL injection |
| djcO. | SYNASTONE 35mg/3.5mL injection |
| djcP. | SYNASTONE 50mg/5mL injection |
| djcQ. | SYNASTONE 50mg/2mL injection |
| djcR. | SYNASTONE 50mg/1mL injection |
| djcS. | PHYSEPTONE 50mg/2mL injection |
| djcT. | PHYSEPTONE 50mg/1mL injection |
| djcU. | EPTADONE 1mg/mL oral solution |
| djcV. | EPTADONE 5mg/mL oral solution |
| djcW. | EPTADONE 20mg/20mL oral soln |
| djcX. | EPTADONE 40mg/40mL oral soln |
| djcY. | EPTADONE 60mg/60mL oral soln |
| djcZ. | EPTADONE 100mg/20mL oral soln |
| djco. | METHADONE 20mg/20mL oral soln |
| djcp. | METHADONE 40mg/40mL oral soln |
| djcq. | METHADONE 60mg/60mL oral soln |
| djcr. | METHADONE 100mg/20mL oral soln |
| djcs. | METHADONE 5mg/mL oral solution |
| djct. | METHADONE HCL 50mg/2mL inj |
| djcu. | METHADONE HCL 50mg/1mL inj |
| djcv. | METHADONE HCL 20mg/2mL inj |
| djcw. | METHADONE HCL 35mg/3.5mL inj |
| djcx. | METHADONE HCL 50mg/5mL inj |
| djcy. | METHADONE HCL 5mg tablets |
| djcz. | METHADONE HCL 10mg/1mL inj |
| du2.. | NALTREXONE HYDROCHLORIDE |
| du21. | NALTREXONE HCL 50mg tablets |
| du22. | NALOREX 50mg tablets |
| du23. | OPIZONE 50mg tablets |
| du24. | ADEPEND 50mg tablets |
| du4.. | LOFEXIDINE HYDROCHLORIDE |
| du41. | BRITLOFEX 200mcg tablets |
| du42. | LOFEXIDINE HCL 200mcg tablets |

**Table 20: Smoking READ codes**

| READ | Description |
| --- | --- |
| 137.. | Tobacco consumption |
| 1372. | Trivial smoker - < 1 cig/day |
| 1373. | Light smoker - 1-9 cigs/day |
| 1374. | Moderate smoker - 10-19 cigs/d |
| 1375. | Heavy smoker - 20-39 cigs/day |
| 1376. | Very heavy smoker - 40+cigs/d |
| 137C. | Keeps trying to stop smoking |
| 137D. | Admitted tobacco cons untrue ? |
| 137E. | Tobacco consumption unknown |
| 137G. | Trying to give up smoking |
| 137H. | Pipe smoker |
| 137J. | Cigar smoker |
| 137M. | Rolls own cigarettes |
| 137P. | Cigarette smoker |
| 137Q. | Smoking started |
| 137R. | Current smoker |
| 137V. | Smoking reduced |
| 137W. | Chews tobacco |
| 137X. | Cigarette consumption |
| 137Y. | Cigar consumption |
| 137Z. | Tobacco consumption NOS |
| 137a. | Pipe tobacco consumption |
| 137b. | Ready to stop smoking |
| 137c. | Thinking about stopping smoking |
| 137d. | Not interested in stopping smoking |
| 137e. | Smoking restarted |
| 137f. | Reason for restarting smoking |
| 137g. | Cigarette pack-years |
| 137h. | Minutes from waking to first tobacco consumption |
| 137m. | Failed attempt to stop smoking |
| 13p.. | Smoking cessation milestones |
| 13p0. | Negotiated date for cessation of smoking |
| 13p5. | Smoking cessation programme start date |
| 13p8. | Lost to smoking cessation follow-up |
| 38DH. | Fagerstrom test for nicotine dependence |
| 6791.. | Health ed. - smoking |
| 67910. | Health education - parental smoking |
| 67A3. | Pregnancy smoking advice |
| 67H1. | Lifestyle advice regarding smoking |
| 67H6. | Brief intervention for smoking cessation |
| 745H. | Smoking cessation therapy |
| 745H0 | Nicotine replacement therapy using nicotine patches |
| 745H1 | Nicotine replacement therapy using nicotine gum |
| 745H2 | Nicotine replacement therapy using nicotine inhalator |
| 745H3 | Nicotine replacement therapy using nicotine lozenges |
| 745H4 | Smoking cessation drug therapy |
| 745Hy | Other specified smoking cessation therapy |
| 745Hz | Smoking cessation therapy NOS |
| 8B2B. | Nicotine replacement therapy |
| 8B3Y. | Over the counter nicotine replacement therapy |
| 8B3f. | Nicotine replacement therapy provided free |
| 8BP3. | Nicotine replacement therapy provided by community pharmacis |
| 8H7i. | Referral to smoking cessation advisor |
| 8HBM. | Stop smoking face to face follow-up |
| 8HTK. | Referral to stop-smoking clinic |
| 8HkQ. | Referral to NHS stop smoking service |
| 8I2I. | Nicotine replacement therapy contraindicated |
| 8I2J. | Bupropion contraindicated |
| 8I39. | Nicotine replacement therapy refused |
| 8I3M. | Bupropion refused |
| 8I6H. | Smoking review not indicated |
| 8IAj. | Smoking cessation advice declined |
| 8IEK. | Smoking cessation programme declined |
| 8IEM. | Smoking cessation drug therapy declined |
| 9N2k. | Seen by smoking cessation advisor |
| 9N4M. | DNA - Did not attend smoking cessation clinic |
| 9NS02 | Referral for smoking cessation service offered |
| 9NdV. | Consent given follow-up after smoking cessation intervention |
| 9NdW. | Consent given for smoking cessation data sharing |
| 9NdY. | Decline cons follow-up evaluation after smoking cess interven |
| 9NdZ. | Declined consent for smoking cessation data sharing |
| 9Ndf. | Consent given for follow-up by smoking cessation team |
| 9Ndg. | Declined consent for follow-up by smoking cessation team |
| 9OO.. | Anti-smoking monitoring admin. |
| 9OO1. | Attends stop smoking monitor. |
| 9OO2. | Refuses stop smoking monitor |
| 9OO3. | Stop smoking monitor default |
| 9OO4. | Stop smoking monitor 1st lettr |
| 9OO5. | Stop smoking monitor 2nd lettr |
| 9OO6. | Stop smoking monitor 3rd lettr |
| 9OO7. | Stop smoking monitor verb.inv. |
| 9OO8. | Stop smoking monitor phone inv |
| 9OO9. | Stop smoking monitoring delete |
| 9OOA. | Stop smoking monitor.chck done |
| 9OOB. | Stop smoking invitation short message service text message |
| 9OOB0 | Stop smoking invitation first SMS text message |
| 9OOB1 | Stop smoking invitation second SMS text message |
| 9OOB2 | Stop smoking invitation third SMS text message |
| 9OOZ. | Stop smoking monitor admin.NOS |
| 9hG.. | Exception reporting: smoking quality indicators |
| 9hG0. | Excepted from smoking quality indicators: Patient unsuitable |
| 9hG1. | Excepted from smoking quality indicators: Informed dissent |
| 9kc.. | Smoking cessation - enhanced services administration |
| 9kc0. | Smoking cessatn monitor template complet - enhanc serv admin |
| 9ko.. | Current smoker annual review - enhanced services admin |
| E023. | Nicotine withdrawal |
| E251. | Tobacco dependence |
| E2510 | Tobacco dependence, unspecified |
| E2511 | Tobacco dependence, continuous |
| E2512 | Tobacco dependence, episodic |
| E2513 | Tobacco dependence in remission |
| E251z | Tobacco dependence NOS |
| TJHy2 | Adverse reaction to nicotine |
| ZV4K0 | [V]Tobacco use |
| ZV6D8 | [V]Tobacco abuse counselling |

**Table 21: Epilepsy ICD10 codes**

| ICD10 | Description |
| --- | --- |
| G40 | Epilepsy |
| G400 | Localization-related (focal)(partial) idiopathic epilepsy and epileptic syndromes with seizures of localized onset |
| G401 | Localization-related (focal)(partial) symptomatic epilepsy and epileptic syndromes with simple partial seizures |
| G402 | Localization-related (focal)(partial) symptomatic epilepsy and epileptic syndromes with complex partial seizures |
| G403 | Generalized idiopathic epilepsy and epileptic syndromes |
| G404 | Other generalized epilepsy and epileptic syndromes |
| G405 | Special epileptic syndromes |
| G406 | Grand mal seizures, unspecified (with or without petit mal) |
| G407 | Petit mal, unspecified, without grand mal seizures |
| G408 | Other epilepsy |
| G409 | Epilepsy, unspecified |
| G41 | Status epilepticus |
| G410 | Grand mal status epilepticus |
| G411 | Petit mal status epilepticus |
| G412 | Complex partial status epilepticus |
| G418 | Other status epilepticus |
| G419 | Status epilepticus, unspecified |

**Table 22: Epilepsy READ codes**

| READ | Description |
| --- | --- |
| 1O30. | Epilepsy confirmed |
| F25.. | Epilepsy |
| F250. | Generalised nonconvuls.epilep. |
| F2500 | Petit mal (minor) epilepsy |
| F2501 | Pykno-epilepsy |
| F2502 | Epileptic seizures - atonic |
| F2503 | Epileptic seizures - akinetic |
| F2504 | Juvenile absence epilepsy |
| F2505 | Lennox-Gastaut syndrome |
| F250y | General.nonconvuls.epilep.OS |
| F250z | General.nonconvuls.epilep.NOS |
| F251. | Generalised convulsive epilep. |
| F2510 | Grand mal (major) epilepsy |
| F2511 | Neonatal myoclonic epilepsy |
| F2512 | Epileptic seizures - clonic |
| F2513 | Epileptic seizures - myoclonic |
| F2514 | Epileptic seizures - tonic |
| F2515 | Tonic-clonic epilepsy |
| F2516 | Grand mal seizure |
| F251y | General.convuls.epilepsy OS |
| F251z | General.convuls.epilepsy NOS |
| F252. | Petit mal status |
| F253. | Grand mal status |
| F254. | Partial epilep.-consc.impaired |
| F2540 | Temporal lobe epilepsy |
| F2541 | Psychomotor epilepsy |
| F2542 | Psychosensory epilepsy |
| F2543 | Limbic system epilepsy |
| F2544 | Epileptic automatism |
| F2545 | Complex partial epilep seizure |
| F254z | Partial epil.-consc.impair.NOS |
| F255. | Partial epilepsy-conscious OK |
| F2550 | Jacksonian/focal/motor epilep. |
| F2551 | Sensory induced epilepsy |
| F2552 | Somatosensory epilepsy |
| F2553 | Visceral reflex epilepsy |
| F2554 | Visual reflex epilepsy |
| F2555 | Unilateral epilepsy |
| F2556 | Simple part epileptic seizure |
| F255y | Partial epilep.-consc.OK OS |
| F255z | Partial epilep.-consc.OK NOS |
| F256. | Infantile spasms |
| F2560 | Hypsarrhythmia |
| F2561 | Salaam attacks |
| F256z | Infantile spasms NOS |
| F257. | Kojevnikov's epilepsy |
| F258. | Post-ictal state |
| F259. | Erly inf ep enceph wth sup bur |
| F25A. | Juvenile myoclonic epilepsy |
| F25B. | Alcohol-induced epilepsy |
| F25C. | Drug-induced epilepsy |
| F25D. | Menstrual epilepsy |
| F25E. | Stress-induced epilepsy |
| F25F. | Photosensitive epilepsy |
| F25G. | Seve myoclon epilepsy in infan |
| F25H. | Generalised seizure |
| F25X. | Status epilepticus, unspec |
| F25y. | Other forms of epilepsy |
| F25y0 | Cursive (running) epilepsy |
| F25y1 | Gelastic epilepsy |
| F25y2 | L-r(f)(p)idio ep&ep s+seiz l o |
| F25y3 | Complx partl status epileptcus |
| F25y4 | Benign Rolandic epilepsy |
| F25y5 | Panayiotopoulos syndrome |
| F25yz | Other forms of epilepsy NOS |
| F25z. | Epilepsy NOS |
| dn... | CONTROL OF EPILEPSY |
| dn1.. | ACETAZOLAMIDE [EPILEPSY] |
| dn11. | *DIAMOX [EP] 500mg m/r caps |
| dn12. | DIAMOX [EP] 250mg tablets |
| dn13. | DIAMOX [EP] 500mg injection |
| dn1x. | *ACETAZOLAMIDE 500mg m/r caps |
| dn1y. | ACETAZOLAMIDE [EP] 250mg tabs |
| dn1z. | ACETAZOLAMIDE [EP] 500mg inj |
| dn2.. | *BECLAMIDE |
| dn21. | *NYDRANE 500mg tablets |
| dn2z. | *BECLAMIDE 500mg tablets |
| dn3.. | CARBAMAZEPINE |
| dn31. | CARBAMAZEPINE 100mg tablets |
| dn32. | CARBAMAZEPINE 200mg tablets |
| dn33. | CARBAMAZEPINE 400mg tablets |
| dn34. | TEGRETOL 100mg tablets |
| dn35. | TEGRETOL 200mg tablets |
| dn36. | TEGRETOL 400mg tablets |
| dn37. | TEGRETOL 100mg/5mL s/f liquid |
| dn38. | TEGRETOL RETARD 200mg m/r tabs |
| dn39. | TEGRETOL RETARD 400mg m/r tabs |
| dn3A. | *EPIMAZ 100mg tablets |
| dn3B. | *EPIMAZ 200mg tablets |
| dn3C. | *EPIMAZ 400mg tablets |
| dn3D. | TEGRETOL 125mg suppositories |
| dn3E. | TEGRETOL 250mg suppositories |
| dn3F. | *TIMONIL RETARD 200mg m/r tabs |
| dn3G. | *TIMONIL RETARD 400mg m/r tabs |
| dn3H. | *TERIL CR 200mg m/r tablets |
| dn3I. | *TERIL CR 400mg m/r tablets |
| dn3J. | CARBAGEN SR 200mg m/r tablets |
| dn3K. | CARBAGEN SR 400mg m/r tablets |
| dn3a. | CARBAMAZEPINE 200mg m/r tabs |
| dn3b. | CARBAMAZEPINE 400mg m/r tabs |
| dn3c. | *TEGRETOL 100mg chew tabs |
| dn3c. | TEGRETOL 100mg chew tabs |
| dn3d. | *TEGRETOL 200mg chew tabs |
| dn3d. | TEGRETOL 200mg chew tabs |
| dn3e. | *ARBIL MR 200mg m/r tablets |
| dn3f. | *ARBIL MR 400mg m/r tablets |
| dn3v. | CARBAMAZEPINE 125mg supps |
| dn3w. | CARBAMAZEPINE 250mg supps |
| dn3x. | CARBAMAZEPINE 200mg chew tabs |
| dn3x. | *CARBAMAZEPINE 200mg chew tabs |
| dn3y. | CARBAMAZEPINE 100mg chew tabs |
| dn3y. | *CARBAMAZEPINE 100mg chew tabs |
| dn3z. | CARBAMAZEPINE 100mg/5mL sf liq |
| dn4.. | CLONAZEPAM [EPILEPSY CONTROL] |
| dn41. | RIVOTRIL 500micrograms tablets |
| dn41. | *RIVOTRIL 500mcg tablets |
| dn42. | RIVOTRIL 2mg tablets |
| dn42. | *RIVOTRIL 2mg tablets |
| dn4w. | CLONAZEPAM 0.5mg/5mL s/f soln |
| dn4x. | CLONAZEPAM 2mg/5mL s/f soln |
| dn4y. | CLONAZEPAM 500mcg tablets |
| dn4z. | CLONAZEPAM 2mg tablets |
| dn5.. | ETHOSUXIMIDE |
| dn51. | *ETHOSUXIMIDE 250mg capsules |
| dn52. | *ETHOSUXIMIDE 250mg/5mL elixir |
| dn53. | *EMESIDE 250mg capsules |
| dn54. | EMESIDE 250mg/5mL syrup |
| dn55. | *ZARONTIN 250mg capsules |
| dn56. | ZARONTIN 250mg/5mL syrup |
| dn5x. | ETHOSUXIMIDE 250mg capsules |
| dn5y. | *ETHOSUXIMIDE 250mg/5mL elixir |
| dn5z. | ETHOSUXIMIDE 250mg/5mL syrup |
| dn6.. | METHYLPHENOBARBITAL |
| dn61. | *PROMINAL 30mg tablets |
| dn62. | *PROMINAL 60mg tablets |
| dn63. | *PROMINAL 200mg tablets |
| dn6x. | *METHYLPHENOBARBITONE 30mg tab |
| dn6y. | *METHYLPHENOBARBITONE 60mg tab |
| dn6z. | *METHYLPHENOBARB 200mg tabs |
| dn7.. | PHENOBARBITAL |
| dn71. | PHENOBARBITAL 15mg tablets |
| dn72. | PHENOBARBITAL 30mg tablets |
| dn73. | PHENOBARBITAL 60mg tablets |
| dn74. | PHENOBARBITAL 100mg tablets |
| dn75. | *PHENOBARB SODIUM 30mg tablets |
| dn76. | *PHENOBARB SODIUM 60mg tablets |
| dn77. | *PHENOBARBITONE 15mg/10mL elix |
| dn78. | PHENOBARBITAL 200mg/1mL inj |
| dn79. | *GARDENAL 200mg/1mL injection |
| dn7a. | *LUMINAL 15mg tablets |
| dn7b. | *LUMINAL 30mg tablets |
| dn7c. | *LUMINAL 60mg tablets |
| dn7d. | PHENOBARBITAL 15mg/5mL elixir |
| dn8.. | PHENYTOIN |
| dn81. | EPANUTIN 30mg/5mL suspension |
| dn82. | EPANUTIN 50mg Infatabs |
| dn83. | PHENYTOIN 50mg chew tabs |
| dn8y. | PHENYTOIN 30mg/5mL suspension |
| dn8z. | PHENYTOIN 90mg/5mL s/f susp |
| dn9.. | PHENYTOIN SODIUM |
| dn91. | PHENYTOIN 50mg tablets |
| dn92. | PHENYTOIN 100mg tablets |
| dn93. | *EPANUTIN 25mg capsules |
| dn93. | EPANUTIN 25mg capsules |
| dn94. | *EPANUTIN 50mg capsules |
| dn94. | EPANUTIN 50mg capsules |
| dn95. | EPANUTIN 100mg capsules |
| dn95. | *EPANUTIN 100mg capsules |
| dn96. | EPANUTIN 300mg capsules |
| dn96. | *EPANUTIN 300mg capsules |
| dn97. | *PENTRAN 50mg tablets |
| dn98. | *PENTRAN 100mg tablets |
| dn9w. | PHENYTOIN SODIUM 300mg caps |
| dn9x. | PHENYTOIN SODIUM 25mg caps |
| dn9y. | PHENYTOIN SODIUM 50mg capsules |
| dn9z. | PHENYTOIN SODIUM 100mg caps |
| dna.. | PRIMIDONE |
| dna1. | MYSOLINE 250mg tablets |
| dna1. | *MYSOLINE 250mg tablets |
| dna2. | *MYSOLINE 250mg/5mL suspension |
| dna3. | *MYSOLINE 50mg tablets |
| dna3. | MYSOLINE 50mg tablets |
| dnax. | PRIMIDONE 50mg tablets |
| dnay. | PRIMIDONE 250mg tablets |
| dnaz. | *PRIMIDONE 250mg/5mL susp |
| dnb.. | SODIUM VALPROATE |
| dnb1. | EPILIM 100mg crushable tablets |
| dnb2. | EPILIM 200mg e/c tablets |
| dnb3. | EPILIM 500mg e/c tablets |
| dnb4. | EPILIM 200mg/5mL s/f liq |
| dnb5. | EPILIM 200mg/5mL syrup |
| dnb6. | EPILIM IV 400mg/4mL injection |
| dnb7. | SOD. VALPROATE 200mg e/c tabs |
| dnb8. | SOD. VALPROATE 500mg e/c tabs |
| dnb9. | *ORLEPT 200mg e/c tabs x10 |
| dnbA. | *ORLEPT 200mg/5mL s/f liquid |
| dnbB. | EPIVAL CR 300mg m/r tablets |
| dnbC. | EPIVAL CR 500mg m/r tablets |
| dnbD. | EPISENTA 300mg/3mL injection |
| dnbE. | SODIUM VALPROATE 300mg/3mL inj |
| dnbF. | EPISENTA 150mg m/r capsules |
| dnbG. | EPISENTA 300mg m/r capsules |
| dnbH. | EPISENTA 500mg/sach m/r grans |
| dnbI. | EPISENTA 1g/sachet m/r grans |
| dnbJ. | SODUM VALPROATE 150mg m/r caps |
| dnbK. | SODUM VALPROATE 300mg m/r caps |
| dnbL. | SOD VALPROATE 500mg m/r grans |
| dnbM. | SOD VALPROTE 1g/sach m/r grans |
| dnbN. | SODIUM VALPROATE 1g/10mL inj |
| dnbO. | EPISENTA 1g/10mL soln for inj |
| dnbP. | EPILIM CHRONO 50mg m/r grans |
| dnbQ. | EPILIM CHRONO 100mg m/r grans |
| dnbR. | EPILIM CHRONO 250mg m/r grans |
| dnbS. | EPILIM CHRONO 500mg m/r grans |
| dnbT. | EPILIM CHRONO 750mg m/r grans |
| dnbU. | EPILIM CHRONO 1g m/r granules |
| dnba. | *ORLEPT 200mg e/c tablets |
| dnbb. | *ORLEPT 500mg e/c tablets |
| dnbc. | EPILIM CHRONO 200 m/r tablets |
| dnbd. | EPILIM CHRONO 300 m/r tablets |
| dnbe. | EPILIM CHRONO 500 m/r tablets |
| dnbn. | SOD VALPROATE 50mg m/r grans |
| dnbo. | SOD VALPROATE 100mg m/r grans |
| dnbp. | SOD VALPROATE 250mg m/r grans |
| dnbq. | SOD VALPROATE 750mg m/r grans |
| dnbr. | SOD VALPROATE 200mg m/r tabs |
| dnbs. | SOD VALPROATE 300mg m/r tabs |
| dnbt. | SOD VALPROATE 500mg m/r tabs |
| dnbu. | SODIUM VALPROATE 400mg/4mL inj |
| dnbv. | SOD VALPROATE 100mg crush tabs |
| dnbw. | *SODIUM VALPROATE 200mg tabs |
| dnbx. | *SODIUM VALPROATE 500mg tabs |
| dnby. | SOD. VALPROATE 200mg/5mL sfliq |
| dnbz. | SODIUM VALPROATE 200mg/5mL syr |
| dnc.. | CLOBAZAM [EPILEPSY ONLY] |
| dnc1. | *CLOBAZAM SLS 10mg capsules |
| dne.. | VIGABATRIN |
| dne1. | VIGABATRIN 500mg tablets |
| dne2. | SABRIL 500mg tablets |
| dne3. | VIGABATRIN 500mg pdr sachets |
| dne4. | SABRIL 500mg powder sachets |
| dnf.. | LAMOTRIGINE |
| dnf1. | LAMOTRIGINE 50mg tablets |
| dnf2. | LAMOTRIGINE 100mg tablets |
| dnf3. | LAMICTAL 50mg tablets |
| dnf4. | LAMICTAL 100mg tablets |
| dnf5. | LAMOTRIGINE 25mg tablets |
| dnf6. | LAMICTAL 25mg tablets |
| dnf7. | LAMICTAL 5mg disp tablets |
| dnf8. | LAMICTAL 25mg disp tablets |
| dnf9. | LAMICTAL 100mg disp tablets |
| dnfA. | LAMOTRIGINE 5mg disp tablets |
| dnfB. | LAMOTRIGINE 25mg disp tablets |
| dnfC. | LAMOTRIGINE 100mg disp tablets |
| dnfD. | LAMICTAL 200mg tablets |
| dnfE. | LAMOTRIGINE 200mg tablets |
| dnfF. | *LAMICTAL MONOTHERAPY pack |
| dnfG. | *LAMICTAL VALPROATE ADD-ON pck |
| dnfH. | *LAMICTAL NON-VALPROATE pack |
| dnfJ. | LAMICTAL 2mg dispersible tabs |
| dnfz. | LAMOTRIGINE 2mg disp tablets |
| dng.. | PIRACETAM |
| dng1. | NOOTROPIL 800mg tablets |
| dng2. | NOOTROPIL 1.2g tablets |
| dng3. | NOOTROPIL 33% oral solution |
| dng4. | PIRACETAM 800mg tablets |
| dng5. | PIRACETAM 1.2g tablets |
| dng6. | PIRACETAM 333.3mg/mL solution |
| dnh.. | VALPROIC ACID |
| dnh1. | CONVULEX 150mg e/c capsules |
| dnh2. | CONVULEX 300mg e/c capsules |
| dnh3. | CONVULEX 500mg e/c capsules |
| dnh4. | VALPROIC ACID 150mg e/c caps |
| dnh5. | VALPROIC ACID 300mg e/c caps |
| dnh6. | VALPROIC ACID 500mg e/c caps |
| dnh7. | DEPAKOTE 250mg e/c tablets |
| dnh8. | DEPAKOTE 500mg e/c tablets |
| dnhy. | VALPROIC ACID 500mg e/c tabs |
| dnhz. | VALPROIC ACID 250mg e/c tabs |
| dni.. | FOSPHENYTOIN SODIUM |
| dni1. | FOSPHENYTOIN NA 750mg/10mL inj |
| dni2. | PRO-EPANUTIN 750mg/10mL inj |
| dnj.. | GABAPENTIN |
| dnj1. | GABAPENTIN 100mg capsules |
| dnj2. | GABAPENTIN 300mg capsules |
| dnj3. | GABAPENTIN 400mg capsules |
| dnj4. | NEURONTIN 100mg capsules |
| dnj5. | NEURONTIN 300mg capsules |
| dnj6. | NEURONTIN 400mg capsules |
| dnj7. | NEURONTIN 600mg tablets |
| dnj8. | NEURONTIN 800mg tablets |
| dnj9. | *NEURONTIN 300mg/600mg pack |
| dnjA. | GABAPENTIN 50mg/mL oral soln |
| dnjx. | *GABAPENTN 300mg cap/600mg tab |
| dnjy. | GABAPENTIN 600mg tablets |
| dnjz. | GABAPENTIN 800mg tablets |
| dnk.. | TOPIRAMATE |
| dnk1. | TOPIRAMATE 50mg tablets |
| dnk2. | TOPIRAMATE 100mg tablets |
| dnk3. | TOPIRAMATE 200mg tablets |
| dnk4. | TOPAMAX 50mg tablets |
| dnk5. | TOPAMAX 100mg tablets |
| dnk6. | TOPAMAX 200mg tablets |
| dnk7. | TOPIRAMATE 25mg tablets |
| dnk8. | TOPAMAX 25mg tablets |
| dnk9. | TOPIRAMATE 15mg beads in caps |
| dnkA. | TOPIRAMATE 25mg beads in caps |
| dnkB. | TOPAMAX SPRINKLE 15mg capsules |
| dnkC. | TOPAMAX SPRINKLE 25mg capsules |
| dnkD. | TOPIRAMATE 50mg beads in caps |
| dnkE. | TOPAMAX SPRINKLE 50mg capsules |
| dnl.. | TIAGABINE |
| dnl1. | TIAGABINE 5mg tablets |
| dnl2. | TIAGABINE 10mg tablets |
| dnl3. | TIAGABINE 15mg tablets |
| dnl4. | GABITRIL 5mg tablets |
| dnl5. | GABITRIL 10mg tablets |
| dnl6. | GABITRIL 15mg tablets |
| dnm.. | OXCARBAZEPINE |
| dnm1. | TRILEPTAL 150 tablets |
| dnm2. | TRILEPTAL 300 tablets |
| dnm3. | TRILEPTAL 600 tablets |
| dnm4. | TRILEPTAL 60mg/mL s/f susp |
| dnmw. | OXCARBAZEPINE 60mg/mL s/f susp |
| dnmx. | OXCARBAZEPINE 150mg tablets |
| dnmy. | OXCARBAZEPINE 300mg tablets |
| dnmz. | OXCARBAZEPINE 600mg tablets |
| dno.. | LEVETIRACETAM |
| dno1. | KEPPRA 250mg tablets |
| dno2. | KEPPRA 500mg tablets |
| dno3. | KEPPRA 1g tablets |
| dno4. | KEPPRA 750mg tablets |
| dno5. | KEPPRA 100mg/mL s/f oral soln |
| dno6. | KEPPRA 500mg/5mL soln for inj |
| dno7. | DESITREND 250mg/sach granules |
| dno8. | DESITREND 500mg/sach granules |
| dno9. | DESITREND 1g/sachet granules |
| dnoA. | DESITREND 100mg/mL oral soln |
| dnoB. | DESITREND 500mg/5mL soln inf |
| dnor. | LEVETIRACETAM 1g/sach granules |
| dnos. | LEVETIRACETAM 500mg/sach grans |
| dnot. | LEVETIRACETAM 250mg/sach grans |
| dnou. | LEVETIRACETAM 500mg/5mL inj |
| dnov. | LEVETIRACTAM 100mg/mL s/f soln |
| dnow. | LEVETIRACETAM 750mg tablets |
| dnox. | LEVETIRACETAM 1g tablets |
| dnoy. | LEVETIRACETAM 500mg tablets |
| dnoz. | LEVETIRACETAM 250mg tablets |
| dnp.. | PREGABALIN |
| dnp1. | LYRICA 25mg capsules |
| dnp2. | LYRICA 50mg capsules |
| dnp3. | LYRICA 75mg capsules |
| dnp4. | LYRICA 100mg capsules |
| dnp5. | LYRICA 150mg capsules |
| dnp6. | LYRICA 200mg capsules |
| dnp7. | LYRICA 300mg capsules |
| dnp8. | LYRICA 225mg capsules |
| dnp9. | LYRICA 20mg/1mL oral solution |
| dnpA. | LECAENT 25mg capsules |
| dnpB. | LECAENT 50mg capsules |
| dnpC. | LECAENT 75mg capsules |
| dnpD. | LECAENT 100mg capsules |
| dnpE. | LECAENT 150mg capsules |
| dnpF. | LECAENT 200mg capsules |
| dnpG. | LECAENT 225mg capsules |
| dnpH. | LECAENT 300mg capsules |
| dnpI. | REWISCA 25mg capsules |
| dnpJ. | REWISCA 50mg capsules |
| dnpK. | REWISCA 75mg capsules |
| dnpL. | REWISCA 100mg capsules |
| dnpM. | REWISCA 150mg capsules |
| dnpN. | REWISCA 200mg capsules |
| dnpO. | REWISCA 225mg capsules |
| dnpP. | REWISCA 300mg capsules |
| dnpr. | PREGABALIN 20mg/1mL solution |
| dnps. | PREGABALIN 225mg capsules |
| dnpt. | PREGABALIN 300mg capsules |
| dnpu. | PREGABALIN 200mg capsules |
| dnpv. | PREGABALIN 100mg capsules |
| dnpw. | PREGABALIN 150mg capsules |
| dnpx. | PREGABALIN 75mg capsules |
| dnpy. | PREGABALIN 50mg capsules |
| dnpz. | PREGABALIN 25mg capsules |
| dnq.. | ZONISAMIDE |
| dnq1. | ZONISAMIDE 25mg capsules |
| dnq2. | ZONISAMIDE 50mg capsules |
| dnq3. | ZONISAMIDE 100mg capsules |
| dnq4. | ZONEGRAN 25mg capsules |
| dnq5. | ZONEGRAN 50mg capsules |
| dnq6. | ZONEGRAN 100mg capsules |
| dnr.. | RUFINAMIDE |
| dnr1. | INOVELON 100mg tablets |
| dnr2. | INOVELON 200mg tablets |
| dnr3. | INOVELON 400mg tablets |
| dnr4. | INOVELON 40mg/mL oral susp |
| dnrw. | RUFINAMIDE 40mg/mL oral susp |
| dnrx. | RUFINAMIDE 400mg tablets |
| dnry. | RUFINAMIDE 200mg tablets |
| dnrz. | RUFINAMIDE 100mg tablets |
| dns.. | STIRIPENTOL |
| dns1. | DIACOMIT 250mg capsules |
| dns2. | DIACOMIT 500mg capsules |
| dns3. | DIACOMIT 250mg/sachet pdr |
| dns4. | DIACOMIT 500mg/sachet pdr |
| dnsw. | STIRIPENTOL 500mg/sachet pdr |
| dnsx. | STIRIPENTOL 250mg/sachet pdr |
| dnsy. | STIRIPENTOL 500mg capsules |
| dnsz. | STIRIPENTOL 250mg capsules |
| dnt.. | LACOSAMIDE |
| dnt1. | VIMPAT 200mg/20mL soln for inj |
| dnt2. | *VIMPAT 15mg/1mL s/f liquid |
| dnt3. | VIMPAT 50mg tablets |
| dnt4. | VIMPAT 100mg tablets |
| dnt5. | VIMPAT 150mg tablets |
| dnt6. | VIMPAT 200mg tablets |
| dnt7. | LACOSAMIDE 200mg/20mL inj |
| dnt8. | *LACOSAMIDE 15mg/1mL s/f liq |
| dnt9. | LACOSAMIDE 50mg tablets |
| dntA. | LACOSAMIDE 100mg tablets |
| dntB. | LACOSAMIDE 150mg tablets |
| dntC. | LACOSAMIDE 200mg tablets |
| dntD. | VIMPAT 10mg/1mL syrup |
| dntE. | LACOSAMIDE 10mg/1mL s/f liquid |
| dnu.. | ESLICARBAZEPINE |
| dnu1. | ZEBINIX 800mg tablets |
| dnu2. | ESLICARBAZPN ACETAT 800mg tabs |
| dnv.. | RETIGABINE |
| dnv1. | TROBALT 50mg tablets |
| dnv2. | TROBALT 100mg tablets |
| dnv3. | TROBALT 200mg tablets |
| dnv4. | TROBALT 300mg tablets |
| dnv5. | TROBALT 400mg tablets |
| dnv6. | TROBALT tabs initiation pack |
| dnv7. | RETIGABINE 50mg tablets |
| dnv8. | RETIGABINE 100mg tablets |
| dnv9. | RETIGABINE 200mg tablets |
| dnvA. | RETIGABINE 300mg tablets |
| dnvB. | RETIGABINE 400mg tablets |
| dnvC. | RETIGABIN 50mg+100mg tabs pack |
| dnw.. | PERAMPANEL |
| dnw1. | FYCOMPA 2mg tablets |
| dnw2. | FYCOMPA 4mg tablets |
| dnw3. | FYCOMPA 6mg tablets |
| dnw4. | FYCOMPA 8mg tablets |
| dnw5. | FYCOMPA 10mg tablets |
| dnw6. | FYCOMPA 12mg tablets |
| dnwu. | PERAMPANEL 12mg tablets |
| dnwv. | PERAMPANEL 10mg tablets |
| dnww. | PERAMPANEL 8mg tablets |
| dnwx. | PERAMPANEL 6mg tablets |
| dnwy. | PERAMPANEL 4mg tablets |
| dnwz. | PERAMPANEL 2mg tablets |
| do... | STATUS EPILEPTICUS DRUGS |
| do1.. | DIAZEPAM [EPILEPSY USE] |
| do11. | DIAZEMULS [EP] 10mg/2mL inj |
| do12. | STESOLID [EP] 10mg/2mL inj |
| do13. | *STESOLID 20mg/4mL injection |
| do14. | STESOLID 5mg/2.5mL rect.soln |
| do15. | STESOLID 10mg/2.5mL rect.soln |
| do16. | VALIUM [EP] 10mg/2mL injection |
| do17. | *VALIUM [EP] 20mg/4mL inj |
| do18. | DIAZEPAM 5mg/2.5mL RecTubes |
| do19. | DIAZEPAM 10mg/2.5mL RecTubes |
| do1A. | DIAZEPAM 2.5mg/1.25mL RecTubes |
| do1B. | *DIAZEPAM 20mg/5mL RecTubes |
| do1t. | DIAZEPAM 2.5mg rectal solution |
| do1u. | *DIAZEPAM 20mg/5mL rect.soln |
| do1v. | DIAZEPAM 10mg/2mL emulsion inj |
| do1w. | DIAZEPAM 10mg/2mL injection |
| do1x. | DIAZEPAM 5mg/2.5mL rect.soln |
| do1y. | DIAZEPAM 10mg/2.5mL rect.soln |
| do1z. | *DIAZEPAM 20mg/4mL injection |
| do2.. | CLONAZEPAM [STATUS EPILEPSY] |
| do21. | RIVOTRIL 1mg/1mL injection |
| do21. | *RIVOTRIL 1mg/1mL injection |
| do2z. | *CLONAZEPAM 1mg/1mL injection |
| do2z. | CLONAZEPAM 1mg/1mL injection |
| do3.. | CLOMETHIAZOLE EDISYL. [CNS] |
| do31. | *HEMINEVRIN 8mg/mL i-v inf |
| do3z. | *CLOMETHIAZOLE EDI 8mg/mL inf |
| do4.. | LORAZEPAM [EPILEPSY] |
| do41. | ATIVAN [EP] 4mg/mL injection |
| do5.. | PARALDEHYDE |
| do51. | *PARALDEHYDE injection 5mL |
| do52. | *PARALDEHYDE injection 10mL |
| do6.. | PHENYTOIN SODIUM [STATUS EP] |
| do61. | EPANUTIN [EP] 250mg/5mL inj |
| do6z. | PHENYTOIN SODIUM 250mg/5mL inj |

**Table 23: Asthma ICD10 codes**

| ICD10 | Description |
| --- | --- |
| J45 | Asthma |
| J450 | Predominantly allergic asthma |
| J451 | Nonallergic asthma |
| J458 | Mixed asthma |
| J459 | Asthma, unspecified |
| J46X | Status asthmaticus |

**Table 24: Asthma READ codes**

| READ_CD | Description |
| --- | --- |
| 173A. | Exercise induced asthma |
| H3120 | Chronic asthmatic bronchitis |
| H33.. | Asthma |
| H330. | Extrinsic (atopic) asthma |
| H3300 | Extrinsic asthma - no status |
| H3301 | Extrinsic asthma + status |
| H330z | Extrinsic asthma NOS |
| H331. | Intrinsic asthma |
| H3310 | Intrinsic asthma - no status |
| H3311 | Intrinsic asthma + status |
| H331z | Intrinsic asthma NOS |
| H332. | Mixed asthma |
| H334. | Brittle asthma |
| H335. | Chron asthm w fix airflw obstr |
| H33z. | Asthma unspecified |
| H33z0 | Status asthmaticus NOS |
| H33z1 | Asthma attack |
| H33z2 | Late-onset asthma |
| H33zz | Asthma NOS |
| H3B.. | Asthma-COPD overlap syndrome |
| c11.. | SALBUTAMOL [ORAL PREPARATIONS] |
| c111. | *ASMAVEN 2mg tablets |
| c112. | *ASMAVEN 4mg tablets |
| c113. | *COBUTOLIN 2mg tablets |
| c114. | *COBUTOLIN 4mg tablets |
| c115. | *SALBULIN 2mg tablets |
| c116. | *SALBULIN 4mg tablets |
| c117. | *SALBULIN 2mg/2mL liquid |
| c118. | *VENTOLIN 2mg tablets |
| c119. | *VENTOLIN 4mg tablets |
| c11A. | *VENTOLIN CR 4mg m/r tablets |
| c11B. | *SALBUTAMOL 4mg m/r tablets |
| c11C. | *VENTOLIN CR 8mg m/r tablets |
| c11D. | SALAPIN 2mg/5mL s/f syrup |
| c11a. | *VENTOLIN 8mg m/r tablets |
| c11b. | VENTOLIN 2mg/5mL syrup |
| c11c. | *VOLMAX 4mg m/r tablets |
| c11d. | *VOLMAX 8mg m/r tablets |
| c11e. | *SALBUVENT 2mg tablets |
| c11f. | *SALBUVENT 4mg tablets |
| c11g. | *SALBUVENT 2mg/5mL syrup |
| c11h. | *SALBUVENT 2mg/5mL syr 2litre |
| c11i. | *VENTOLIN CR 4mg m/r tablets |
| c11j. | *SALBUTAMOL 4mg m/r tablets |
| c11k. | *VENTOLIN CR 8mg m/r tablets |
| c11m. | *LIBETIST 2mg/5mL s/f syrup |
| c11n. | *SALBUTAMOL 4mg m/r capsules |
| c11n. | SALBUTAMOL 4mg m/r capsules |
| c11o. | *SALBUTAMOL 8mg m/r capsules |
| c11o. | SALBUTAMOL 8mg m/r capsules |
| c11p. | VENTMAX SR 4mg m/r capsules |
| c11p. | *VENTMAX SR 4mg m/r capsules |
| c11q. | VENTMAX SR 8mg m/r capsules |
| c11q. | *VENTMAX SR 8mg m/r capsules |
| c11v. | SALBUTAMOL 4mg tablets |
| c11w. | *SALBUTAMOL 2mg/2mL liquid |
| c11x. | SALBUTAMOL 2mg tablets |
| c11y. | *SALBUTAMOL 8mg m/r tablets |
| c11z. | SALBUTAMOL 2mg/5mL s/f syrup |
| c12.. | SALBUTAMOL [PARENTERAL PREPS] |
| c121. | *VENTOLIN 250mcg/5mL injection |
| c122. | VENTOLIN 500mcg/1mL injection |
| c123. | VENTOLIN 5mg/5mL i-v infusion |
| c124. | *SALBUVENT 250mcg/5mL inj |
| c125. | *SALBUVENT 500mcg/1mL inj |
| c126. | *SALBUVENT 5mg/5mL i-v inf |
| c12w. | *SALBUTAMOL 5mg/50mL injection |
| c12x. | *SALBUTAMOL 250mcg/5mL inj |
| c12y. | SALBUTAMOL 500mcg/1mL inj |
| c12z. | SALBUTAMOL 5mg/5mL i-v inf |
| c13.. | SALBUTAMOL [INHALATION PREPS] |
| c131. | *ASMAVEN 100micrograms inhaler |
| c132. | *COBUTOLIN 100mcg inhaler |
| c133. | *SALBULIN 100mcg inhaler |
| c134. | *VENTOLIN 100mcg inhaler |
| c135. | VENTOLIN 2.5mg/2.5mL Nebules |
| c136. | *VENTOLIN 200mcg rotacaps |
| c137. | *VENTOLIN 400mcg rotacaps |
| c138. | ROTAHALER DEVICE |
| c139. | VENTOLIN 100mg/20mL resp soln |
| c13A. | STERI-NEB SALAMOL 2.5mg nebs |
| c13B. | STERI-NEB SALAMOL 5mg nebs |
| c13C. | *SALBUTAMOL 200mcg disks+inh |
| c13D. | *SALBUTAMOL 400mcg disks+inh |
| c13E. | *SALBUTAMOL 200mcg disk refill |
| c13F. | *SALBUTAMOL 400mcg disk refill |
| c13G. | *SALBUT 100mcg bth-ac aero inh |
| c13H. | *SALAMOL 100micrograms inhaler |
| c13I. | AIROMIR 100mcg CFC-free inh |
| c13J. | SALBUTAMOL 100mcg CFC-free inh |
| c13K. | *SALAMOL EASI-BRETH 100mcg inh |
| c13L. | SALBUT 200mcg bth-act pdr inh |
| c13M. | VENTOLIN 200mcg Accuhaler |
| c13N. | *SALBUTAMOL 100mcg vortex inh |
| c13O. | *VENTOLIN EASI-BRTH 100mcg inh |
| c13P. | *SALBUTAMOL 100mcg Spacehaler |
| c13Q. | ASMASAL 95mcg Clickhaler |
| c13R. | SALBUT 100mcg bth-act pdr inh |
| c13S. | SALBUTAM 95mcg bth-act pdr inh |
| c13T. | VENTOLIN 100mcg Evohaler |
| c13U. | SAL 100 CFC-fr br-act aero inh |
| c13V. | AIROMIR 100 CFC-free Autohaler |
| c13W. | *MAXIVENT 2.5mg/2.5mL Steriple |
| c13X. | *MAXIVENT 5mg/2.5mL Steripoule |
| c13Y. | *SALBULIN 100mcg CFC-fr inh |
| c13Z. | SALAMOL 100mcg CFC-free inh |
| c13a. | *AEROLIN-400 100mcg inhaler |
| c13b. | *ROTAHALER DEVICE |
| c13c. | *AEROLIN AUTO 100mcg inhaler |
| c13d. | *VENTODISKS 200mcg dskhal 14x8 |
| c13e. | *VENTODISKS 400mcg dskhal 14x8 |
| c13f. | *VENTODISKS 200mcg refill 14x8 |
| c13g. | *VENTODISKS 400mcg refill 14x8 |
| c13h. | *SALBUVENT 100mcg inhaler |
| c13i. | *SALBUVENT RONDO 100mcg inh |
| c13j. | *SALBUVENT 5mg/mL resp soln |
| c13k. | *SALBUVENT RONDO spacer x1 |
| c13l. | *AEROLIN 100mcg Autohaler 200d |
| c13m. | VENTOLIN 5mg/2.5mL Nebules |
| c13n. | *AEROLIN 100mcg Autohaler 100d |
| c13o. | SALBUTAMOL 5mg/2.5mL neb units |
| c13p. | *MAXIVENT 100microgram inhaler |
| c13q. | *SALBUTAMOL 200 cyclocaps |
| c13r. | *SALBUTAMOL 400 cyclocaps |
| c13s. | *VENTOLIN rotahaler device |
| c13v. | SALBUTAMOL 100mcg inhaler |
| c13w. | SALBUTAMOL 2.5mg/2.5mL nebs |
| c13x. | *SALBUTAMOL 200mcg inhal caps |
| c13y. | *SALBUTAMOL 400mcg inhal caps |
| c13z. | SALBUTAMOL 100mg/20mL resp sol |
| c14.. | TERBUTALINE SULFATE [RESP] |
| c14.. | TERBUTALINE SULPHATE [RESP] |
| c141. | BRICANYL 5mg tablets |
| c142. | BRICANYL 1.5mg/5mL syrup |
| c143. | BRICANYL 500mcg/1mL injection |
| c144. | *BRICANYL 250mcg inhaler |
| c145. | *BRICANYL 250micrograms refill |
| c146. | *BRICANYL 250mcg spacer inh |
| c147. | BRICANYL RESPULES 5mg/2mL soln |
| c147. | *BRICANYL RESPULES 5mg/2mL |
| c148. | *BRICANYL 100mg/10mL resp soln |
| c149. | *BRICANYL SA 7.5mg m/r tablets |
| c14a. | *MONOVENT 5mg tablets |
| c14b. | *MONOVENT 1.5mg/5mL syrup |
| c14c. | *MONOVENT SA 7.5mg m/r tablets |
| c14d. | *NEBUHALER spacer device |
| c14e. | BRICANYL 2.5mg/5mL injection |
| c14f. | BRICANYL 500mcg Turbohaler |
| c14g. | *TERBUTALINE 500mcg inhaler |
| c14h. | TERBUTALINE 2.5mg/5mL inj |
| c14i. | TERBUTAL 200mg/20mL neb.soln |
| c14i. | *TERBUTAL 200mg/20mL neb.soln |
| c14j. | TERBUTALINE 500mcg bth-act inh |
| c14k. | *BRICANYL 200mg/20mL resp soln |
| c14k. | BRICANYL 200mg/20mL resp soln |
| c14r. | TERBUTALINE 5mg tablets |
| c14s. | TERBUTALINE 500mcg/1mL inj |
| c14t. | *TERBUTALINE 250mcg inhaler |
| c14u. | *TERBUTALINE 250mcg refill |
| c14v. | *TERBUTALINE 250mcg spacer |
| c14w. | *TERBUTALINE 5mg/2mL neb.soln |
| c14x. | *TERBUTAL 100mg/10mL resp soln |
| c14y. | *TERBUTALINE 7.5mg m/r tablets |
| c14z. | TERBUTALINE 1.5mg/5mL syrup |
| c15.. | FENOTEROL HYDROBROMIDE |
| c151. | *BEROTEC 200micrograms inhaler |
| c152. | *BEROTEC 100mg/20mL resp soln |
| c153. | *BEROTEC 100micrograms inhaler |
| c154. | *FENOTEROL 100mcg inhaler |
| c15y. | *FENOTEROL 200mcg inhaler |
| c15z. | *FENOTEROL 100mg/20mL resp sol |
| c19.. | SALMETEROL XINAFOATE |
| c191. | *SALMETEROL 25mcg inhaler |
| c192. | *SEREVENT 25microgram inhaler |
| c193. | SEREVENT 50microgram diskhaler |
| c193. | *SEREVENT 50mcg diskhaler |
| c194. | *SEREVENT 50mcg disk refill |
| c194. | SEREVENT 50mcg disk refill |
| c195. | *SALMETEROL 50mcg disks+inh |
| c195. | SALMETEROL 50mcg disks+inh |
| c196. | SALMETEROL 50mcg disk refill |
| c196. | *SALMETEROL 50mcg disk refill |
| c197. | SALMETEROL 50mcg bth-act inh |
| c198. | SEREVENT 50mcg Accuhaler |
| c199. | SEREVENT 25micrograms Evohaler |
| c19A. | NEOVENT 25mcg CFC-free inhaler |
| c19B. | VERTINE 25mcg CFC-free inhaler |
| c19z. | SALMETEROL 25mcg CFC-free inh |
| c1B.. | BAMBUTEROL HYDROCHLORIDE |
| c1B1. | BAMBEC 10mg tablets |
| c1B2. | BAMBEC 20mg tablets |
| c1B3. | BAMBUTEROL HCL 10mg tablets |
| c1B4. | BAMBUTEROL HCL 20mg tablets |
| c1C.. | FORMOTEROL |
| c1C1. | FORMOTEROL 12mcg caps+inhaler |
| c1C2. | FORADIL 12mcg inh caps+inhaler |
| c1C3. | FORMOTEROL 6mcg bth-act inh |
| c1C4. | FORMOTEROL 12mcg bth-act inh |
| c1C5. | OXIS 6micrograms Turbohaler |
| c1C6. | OXIS 12micrograms Turbohaler |
| c1C7. | ATIMOS MODULITE 12mcg inhaler |
| c1C8. | FORMOTEROL EASYHALER 12mcg inh |
| c1Cy. | FRMOTERL FUM DHYDRTE 12mcg inh |
| c1Cz. | FORMOTEROL 12mcg inhaler |
| c1D.. | SALMETEROL+FLUTICASONE PROPION |
| c1D1. | SERETIDE 100 Accuhaler |
| c1D2. | SERETIDE 250 Accuhaler |
| c1D3. | SERETIDE 500 Accuhaler |
| c1D4. | SERETIDE 50 Evohaler |
| c1D5. | SERETIDE 125 Evohaler |
| c1D6. | SERETIDE 250 Evohaler |
| c1D7. | SIRDUPLA 25mcg/125mcg inhaler |
| c1D8. | SIRDUPLA 25mcg/250mcg inhaler |
| c1Du. | SALM+FLUT 25/50 CFC-free inh |
| c1Dv. | SALM+FLUT 25/125 CFC-free inh |
| c1Dw. | SALM+FLUT 25/250 CFC-free inh |
| c1Dx. | SALM+FLUT 50/100 b-act pdr inh |
| c1Dy. | SALM+FLUT 50/250 b-act pdr inh |
| c1Dz. | SALM+FLUT 50/500 b-act pdr inh |
| c1E.. | SALBUTAMOL [INHALATION PREP 2] |
| c1b1. | ONBREZ BREZHAL 150mcg inh caps |
| c1b2. | INDACATEROL 150mcg inh caps |
| c1b3. | ONBREZ BREZHAL 300mcg inh caps |
| c1b4. | INDACATEROL 300mcg inh caps |
| c1c.. | FLUTICASONE+FORMOTEROL |
| c1c1. | FLUTIFORM 50mcg/5mcg inhaler |
| c1c2. | FLUTIFORM 125mcg/5mcg inhaler |
| c1c3. | FLUTIFORM 250mcg/10mcg inhaler |
| c1cx. | FLUTIC+FORMOT 250mcg/10mcg inh |
| c1cy. | FLUTICA+FORMOT 125mcg/5mcg inh |
| c1cz. | FLUTICA+FORMOT 50mcg/5mcg inh |
| c1d.. | OLODATEROL |
| c1d1. | STRIVERDI RESP 2.5mcg inhaler |
| c1d2. | OLODATEROL 2.5mcg inhaler |
| c1e.. | INDACATEROL+GLYCOPYRRONIUM |
| c1… | SELECTIVE BETA-ADRENOCEPT.STIM |
| c2... | OTHER ADRENOCEPTOR STIMULANTS |
| c21.. | ADRENALINE [RESP] |
| c211. | *ADRENALINE 500mcg/0.5mL inj |
| c212. | *ADRENALINE 1mg/1mL injection |
| c213. | *MEDIHALER-EPI 280mcg inhaler |
| c214. | *MIN-I-JET ADREN 500mcg/0.5mL |
| c215. | *MIN-I-JET ADREN 1mg/1mL inj |
| c216. | *ADRENALINE 280mcg inhaler |
| c22.. | EPHEDRINE HYDROCHLORIDE [RESP] |
| c221. | EPHEDRINE HCL 15mg tablets |
| c222. | EPHEDRINE HCL 30mg tablets |
| c223. | *EPHEDRINE HCL 60mg tablets |
| c224. | EPHEDRINE HCL 15mg/5mL elixir |
| c225. | *CAM SF 15mg/5mL mixture |
| c226. | CAM 4mg/5mL sugar free mixture |
| c227. | EPHEDRINE HCL 4mg/5mL s/f mixt |
| c25.. | ORCIPRENALINE SULPHATE [RESP] |
| c25.. | ORCIPRENALINE SULFATE [RESP] |
| c251. | *ALUPENT 20mg tablets |
| c252. | *ALUPENT 10mg/5mL syrup |
| c253. | *ALUPENT 500mcg/1mL injection |
| c254. | *ALUPENT 750micrograms inhaler |
| c255. | *ALUPENT 750mcg inhaler refill |
| c25v. | *ORCIPRENALINE 20mg tablets |
| c25w. | *ORCIPRENALINE 10mg/5mL syrup |
| c25x. | *ORCIPRENALINE 500mcg/1mL inj |
| c25y. | *ORCIPRENALINE 750mcg inhaler |
| c25z. | *ORCIPREN 750mcg inh refill |
| c3... | ANTICHOLINERGIC BRONCHODILAT. |
| c31.. | IPRATROPIUM BROMIDE [1] |
| c311. | *ATROVENT 20micrograms inhaler |
| c312. | ATROVENT 500mcg/2mL neb.soln |
| c313. | *ATROVENT FORTE 40mcg inhaler |
| c314. | ATROVENT 250mcg/1mL neb.soln |
| c315. | *ATROVENT 20mcg Autohaler |
| c316. | STERI-NEB IPRATROPIUM 250mcg |
| c317. | STERI-NEB IPRATROPIUM 500mcg |
| c318. | *ATROVENT 40mcg Aerocaps refil |
| c319. | *ATROVENT AEROCAP 40mcg+device |
| c31A. | *IPRATROPIUM 40mcg inhal.caps |
| c31B. | *IPRATROPIUM 40mcg caps+device |
| c31C. | RESPONTIN 250mcg/1mL Nebules |
| c31D. | RESPONTIN 500mcg/2mL Nebules |
| c31E. | *TROPIOVENT 250mcg/1mL Steripl |
| c31F. | *TROPIOVENT 500mcg/2mL Steripl |
| c31G. | ATROVENT 20mcg CFC-free inh |
| c31t. | IPRATROPIUM 20mcg CFC-free inh |
| c31u. | *IPRATROPIUM 20mcg bth-act inh |
| c31v. | IPRATROPIUM 250mcg/1mL neb.sol |
| c31w. | IPRATROPIUM 500mcg/2mL neb.sol |
| c31x. | *IPRATROPIUM 20mcg inhaler |
| c31y. | *IPRATROPIUM 250mcg/mL neb.sol |
| c31z. | *IPRATROPIUM 40mcg inhaler |
| c33.. | TIOTROPIUM |
| c331. | TIOTROPIUM 18mcg inhal caps |
| c332. | TIOTROPIUM 18mcg caps+device |
| c333. | TIOTROPIUM 2.5mcg carts+device |
| c33x. | SPIRIVA RESPIMAT 2.5mcg carts |
| c33y. | SPIRIVA COMBOPACK 18mcg caps |
| c33z. | SPIRIVA 18mcg inhalation caps |
| c34.. | ACLIDINIUM |
| c341. | EKLIRA GENUAIR 322mcg/dose inh |
| c342. | ACLIDINIUM 322mcg/dose inhaler |
| c35.. | UMECLIDINIUM |
| c351. | INCRUSE ELLIPTA 55mcg/dose inh |
| c352. | UMECLIDINIUM 55mcg/dose inh |
| c4... | XANTHINE BRONCHODILATORS |
| c41.. | AMINOPHYLLINE |
| c411. | AMINOPHYLLINE 100mg tablets |
| c412. | AMINOPHYLLINE 250mg/10mL inj |
| c413. | *AMINOPHYLLINE 500mg/2mL inj |
| c414. | *AMINOPHYLLINE 50mg supps |
| c415. | *AMINOPHYLLINE 100mg supps |
| c416. | *AMINOPHYLLINE 150mg supps |
| c417. | *AMINOPHYLLINE 180mg supps |
| c418. | *AMINOPHYLLINE 360mg supps |
| c419. | *THEODROX tablets |
| c41A. | *NORPHYLLIN 100mg tablets |
| c41B. | NORPHYLLIN SR 225mg m/r tabs |
| c41C. | *NORPHYLLIN SR 350mg m/r tabs |
| c41a. | PHYLLOCONTIN CONT 225mg tabs |
| c41b. | PHYLLOCONTIN FORTE 350mg tabs |
| c41c. | *PHYLLOCONTIN PAED 100mg tab |
| c41d. | AMINOPHYLLINE 225mg m/r tabs |
| c41e. | *PECRAM 225mg m/r tablets |
| c41f. | AMINOPHYLLINE 350mg m/r tabs |
| c41g. | *AMINOPHYLLINE 100mg m/r tabs |
| c41h. | *AMNIVENT 225mg m/r tablets |
| c41i. | *AMNIVENT 350mg m/r tablets |
| c41j. | MIN-I-JET AMINOPHYLLINE 250mg |
| c41k. | AMINOPHYLLINE 250mg/10mL syrng |
| c41m. | *AMINOPHYLL HYD 225mg m/r tabs |
| c43.. | THEOPHYLLINE |
| c431. | *BIOPHYLLINE 125mg/5mL syrup |
| c432. | *NUELIN 125mg tablets |
| c433. | *NUELIN 60mg/5mL liquid |
| c434. | *LASMA 300mg m/r tablets |
| c435. | NUELIN SA 175mg m/r tablets |
| c436. | NUELIN SA-250 250mg m/r tabs |
| c437. | *PRO-VENT 300mg m/r capsules |
| c438. | SLO-PHYLLIN 60mg m/r capsules |
| c439. | SLO-PHYLLIN 125mg m/r capsules |
| c43A. | *THEOPHYLLINE 200mg/10mL inj |
| c43B. | THEOPHYLLINE 10mg/5mL s/f soln |
| c43a. | SLO-PHYLLIN 250mg m/r capsules |
| c43b. | *THEO-DUR 200mg m/r tablets |
| c43c. | *THEO-DUR 300mg m/r tablets |
| c43d. | *THEOGRAD 350mg m/r tablets |
| c43e. | UNIPHYLLIN CONTINUS 400mg tabs |
| c43f. | UNIPHYLLIN CONT 200mg m/r tabs |
| c43g. | *LABOPHYLLINE 200mg/10mL inj |
| c43h. | UNIPHYLLIN CONTINUS 300mg tabs |
| c43i. | *BIOPHYLLINE 350mg m/r tablets |
| c43j. | *BIOPHYLLINE 500mg m/r tablets |
| c43k. | THEOPHYLLINE 500mg m/r tablets |
| c43m. | *THEOPHYLLINE 125mg/5mL syrup |
| c43n. | *THEOPHYLLINE 125mg tablets |
| c43o. | *THEOPHYLLINE 60mg/5mL liquid |
| c43p. | THEOPHYLLINE 175mg m/r tablets |
| c43q. | THEOPHYLLINE 250mg m/r tablets |
| c43r. | *THEOPHYLLINE 300mg m/r caps |
| c43s. | THEOPHYLLINE 60mg m/r capsules |
| c43t. | THEOPHYLLINE 125mg m/r caps |
| c43u. | THEOPHYLLINE 250mg m/r caps |
| c43v. | THEOPHYLLINE 200mg m/r tabs |
| c43w. | THEOPHYLLINE 300mg m/r tabs |
| c43x. | *THEOPHYLLINE 350mg m/r tabs |
| c43y. | THEOPHYLLINE 400mg m/r tablets |
| c43z. | *THEOPHYLLINE 200mg tablets |
| c5... | COMPOUND BRONCHODILATORS |
| c51.. | COMPOUND BRONCHODILATORS A-Z |
| c511. | *ADRENALINE+ATROPINE CO. spray |
| c512. | *ALUPENT EXPECTORANT 20mg tabs |
| c513. | *ALUPENT EXPECTORANT mixture |
| c514. | *ASMA-VYDRIN spray |
| c515. | *ASMA-VYDRIN spray 120mL |
| c516. | *BRICANYL COMPOUND tablets |
| c517. | *BRICANYL EXPECTORANT elixir |
| c518. | *BRONCHILATOR inhaler |
| c519. | *BROVON spray 20mL |
| c51A. | *FENOT+IPRAT 100/40mcg inhaler |
| c51B. | *FENOT+IPRA 100/40 bth-act inh |
| c51C. | *SALBUT+IPRATR 100/20mcg inh |
| c51D. | *COMBIVENT inhaler |
| c51E. | COMBIVENT Unit Dose Vials |
| c51F. | SALBUTAMOL+IPRATROPIUM udv |
| c51G. | SALIPRANEB nebulisr soln 2.5mL |
| c51H. | IPRATROPIUM+SALBUT neb soln |
| c51I. | ANORO ELLIPTA 55mcg/22mcg inh |
| c51J. | UMECLIDIN+VILANT 55/22mcg inh |
| c51K. | DUAKLIR GENUAIR 340/12mcg inh |
| c51L. | ACLIDIN+FORMOTER 340/12mcg inh |
| c51a. | *BROVON spray 50mL |
| c51b. | *BROVON MIDGET inhaler |
| c51c. | BROVON RESERVOIR+CLOSURE |
| c51d. | BROVON RUBBER BULB |
| c51e. | *BROVON pressurised inhaler |
| c51f. | *CAM mixture |
| c51g. | *DUO-AUTOHALER inhaler |
| c51h. | *DUO-AUTOHALER refill cannist |
| c51i. | *DUOVENT inhaler |
| c51j. | *ISO-BROVON pressurised inh |
| c51k. | *ISO-BROVON PLUS inhaler |
| c51l. | *MEDIHALER DUO inhaler |
| c51m. | *NETHAPRIN DOSPAN m/r tablets |
| c51n. | RYBARVIN INHALANT soln 30mL |
| c51o. | *RYBAR NO-1 inhaler |
| c51p. | *RYBAR NO-2 inhaler |
| c51q. | *TAUMASTHMAN tablets |
| c51r. | *TEDRAL tablets |
| c51s. | *TEDRAL elixir |
| c51t. | *FRANOL NEW 11mg/120mg tablets |
| c51u. | *FRANOL PLUS NEW 15/120mg tabs |
| c51v. | *DUOVENT UDV neb solution 4mL |
| c51w. | *IPRATROP+FENOTER neb soln 4mL |
| c51x. | *DUOVENT Autohaler |
| c51y. | *AMBIGUOUS CODE-DUPLICAT ISSUE |
| c53.. | COMPOUND BRONCHODILATORS [1] |
| c531. | IPRAMOL STERI-NEB soln 2.5mL |
| c61.. | BECLOMETASONE DIPROPIONATE [RESPIRATORY USE] |
| c611. | BECLOFORTE 250microgram inhaler |
| c612. | BECOTIDE-50 50microgram inhaler |
| c613. | BECOTIDE 100micrograms rotacaps |
| c614. | BECOTIDE 200micrograms rotacaps |
| c615. | *BECOTIDE rotahaler device |
| c616. | BECOTIDE 50micrograms/mL nebuliser solution |
| c617. | BECOTIDE-100 100microgram inhaler |
| c618. | *VOLUMATIC spacer device |
| c619. | BECODISK 100micrograms diskhaler 14x8 |
| c61A. | BECLOMETASONE DIPROPIONATE 400micrograms disks+disk inhaler |
| c61B. | BECLOMETASONE DIPROPIONATE 400micrograms disk refill |
| c61C. | BECLOMETHASONE DIPROPIONATE 250mcg inhaler+spacer device |
| c61E. | BECLOMETASONE DIPROP 250mcg breath-actuated aerosol inhaler |
| c61F. | BECLOMETASONE DIPROP 100mcg breath-actuated aerosol inhaler |
| c61G. | *FILAIR 50micrograms inhaler |
| c61H. | *FILAIR 100micrograms inhaler |
| c61J. | FILAIR FORTE 250micrograms inhaler |
| c61K. | BECLAZONE 50micrograms inhaler |
| c61L. | BECLAZONE 100micrograms inhaler |
| c61M. | BECLAZONE 250micrograms inhaler |
| c61N. | BECLAZONE 50 EASI-BREATHE inhaler |
| c61O. | BECLAZONE 100 EASI-BREATHE inhaler |
| c61P. | BECLAZONE 250 EASI-BREATHE inhaler |
| c61Q. | BECLOFORTE INTEGRA 250micrograms inhaler+compact spacer |
| c61R. | BECLOFORTE INTEGRA 250micrograms refill |
| c61S. | BECLOMETHASONE DIPROPIONATE 250mcg inhaler+compact spacer |
| c61T. | BECLOMETHASONE DIPROPIONATE 250mcg compact spacer refill |
| c61U. | BECLOMETHASONE rotahaler device |
| c61V. | BECLOMETHASONE DIPROPIONATE 50mcg vortex metered dose inh |
| c61W. | *BDP 50micrograms Spacehaler |
| c61X. | BECLOMETHASONE DIPROPIONATE 100mcg vortex metered dose inh |
| c61Y. | *BDP 100micrograms Spacehaler |
| c61Z. | BECLOMETHASONE DIPROPIONATE 250mcg vortex metered dose inh |
| c61a. | BECODISK 200micrograms diskhaler 14x8 |
| c61b. | BECOTIDE 400micrograms rotacaps |
| c61c. | BECODISK 100micrograms disk refill 14x8 |
| c61d. | BECODISK 200micrograms disk refill 14x8 |
| c61e. | BECODISK 400micrograms diskhaler 7x8 |
| c61f. | BECODISK 400micrograms disk refill 7x8 |
| c61g. | BECLOFORTE VM 250micrograms inhaler+volumatic |
| c61h. | BECLOMETASONE DIPROPIONATE 400micrograms inhalation capsules |
| c61i. | BECOTIDE-200 200microgram inhaler |
| c61j. | *AEROBEC 50microgram Autohaler |
| c61k. | AEROBEC FORTE 250micrograms Autohaler |
| c61l. | AEROBEC 100microgram Autohaler |
| c61m. | BECLOFORTE DISKHALER 400micrograms 14x8 |
| c61n. | BECLOFORTE DISKS 400micrograms disk refill 14x8 |
| c61p. | BECLOMETASONE DIPROPIONATE 100micrograms disks+disk inhaler |
| c61q. | BECLOMETASONE DIPROPIONATE 200micrograms disks+disk inhaler |
| c61r. | BECLOMETASONE DIPROPIONATE 100micrograms disk refill |
| c61s. | BECLOMETASONE DIPROPIONATE 200micrograms disk refill |
| c61u. | BECLOMETASONE DIPROPIONATE 200micrograms inhaler |
| c61v. | BECLOMETASONE DIPROPIONATE 50micrograms inhaler |
| c61w. | BECLOMETASONE DIPROPIONATE 100micrograms inhalation capsules |
| c61x. | BECLOMETASONE DIPROPIONATE 200micrograms inhalation capsules |
| c61y. | BECLOMETHASONE DIPROPIONATE 50mcg/mL nebuliser solution |
| c61z. | BECLOMETASONE DIPROPIONATE 100micrograms inhaler |
| c62.. | BECLOMETASONE COMPOUNDS |
| c621. | *VENTIDE inhaler |
| c622. | *VENTIDE Rotacaps |
| c623. | *VENTIDE paediatric Rotacaps |
| c624. | *VENTIDE Rotahaler device |
| c63.. | *BETAMETHASONE VALERATE |
| c631. | *BEXTASOL 100microgram inhaler |
| c63z. | BETAMETHASONE 100micrograms inhaler |
| c64.. | BUDESONIDE [RESPIRATORY USE] |
| c641. | PULMICORT 200micrograms inhaler 200dose |
| c642. | PULMICORT 200micrograms refill 100dose |
| c643. | PULMICORT 200micrograms refill 200dose |
| c644. | PULMICORT LS 50micrograms inhaler |
| c645. | PULMICORT LS 50micrograms refill |
| c646. | *NEBUHALER spacer device |
| c647. | PULMICORT 200microgram inhaler 100dose |
| c649. | PULMICORT 400microgram Turbohaler 50dose |
| c64A. | BUDESONIDE 200micrograms refill cannister |
| c64B. | BUDESONIDE 50micrograms spacer inhaler |
| c64C. | PULMICORT 200micrograms spacer inhaler |
| c64D. | PULMICORT LS 50micrograms spacer inhaler |
| c64E. | PULMICORT 200micrograms inhaler with NebuChamber |
| c64F. | BUDESONIDE 200micrograms/dose dry powder cartridge refill |
| c64G. | NOVOLIZER BUDESONIDE 200micrograms/dose cartridge refill |
| c64H. | EASYHALER BUDESONIDE 100mcg breath-actuated dry powder inh |
| c64I. | EASYHALER BUDESONIDE 200mcg breath-actuated dry powder inh |
| c64J. | EASYHALER BUDESONIDE 400mcg breath-actuated dry powder inh |
| c64K. | PULMICORT 100micrograms CFC-free inhaler |
| c64a. | PULMICORT 500micrograms Respules 2mL unit |
| c64b. | PULMICORT 1mg Respules 2mL unit |
| c64c. | PULMICORT 100microgram Turbohaler 200dose |
| c64d. | BUDESONIDE 100micrograms breath-actuated dry powder inhaler |
| c64e. | BUDESONIDE 50micrograms refill cannister |
| c64g. | BUDESONIDE 200micrograms breath-actuated dry powder inhaler |
| c64h. | BUDESONIDE 400micrograms breath-actuated dry powder inhaler |
| c64i. | BUDESONIDE 500micrograms/2mL nebuliser solution |
| c64j. | BUDESONIDE 1mg/2mL nebuliser solution |
| c64k. | *BUDESONIDE 200 Cyclocaps |
| c64l. | *BUDESONIDE 400 Cyclocaps |
| c64m. | BUDESONIDE 200micrograms inhalation capsules |
| c64n. | BUDESONIDE 400micrograms inhalation capsules |
| c64o. | BUDESONIDE 200micrograms inhaler with spacer device |
| c64p. | NOVOLIZER BUDESONIDE 200micrograms/dose cartridge+inhaler |
| c64u. | BUDESONIDE 200micrograms/dose dry powder cartridge+inhaler |
| c64v. | BUDESONIDE 200micrograms inhaler |
| c64x. | *BUDESONIDE refill 200dose |
| c64y. | BUDESONIDE 50micrograms inhaler |
| c64z. | BUDESONIDE 200micrograms spacer inhaler |
| c65.. | FLUTICASONE PROPIONATE [RESPIRATORY USE] |
| c651. | FLIXOTIDE 50micrograms diskhaler |
| c652. | FLIXOTIDE 100micrograms diskhaler |
| c653. | FLIXOTIDE 250micrograms diskhaler |
| c654. | FLUTICASONE PROPIONATE 50micrograms disks+disk inhaler |
| c655. | FLUTICASONE PROPIONATE 100micrograms disks+disk inhaler |
| c656. | FLUTICASONE PROPIONATE 250micrograms disks+disk inhaler |
| c657. | FLIXOTIDE 50micrograms disk refill |
| c658. | FLIXOTIDE 100micrograms disk refill |
| c659. | FLIXOTIDE 250micrograms disk refill |
| c65A. | FLUTICASONE PROPIONATE 50micrograms disk refill |
| c65B. | FLUTICASONE PROPIONATE 100micrograms disk refill |
| c65C. | FLUTICASONE PROPIONATE 250micrograms disk refill |
| c65D. | FLIXOTIDE 25micrograms inhaler |
| c65E. | FLIXOTIDE 50micrograms inhaler |
| c65F. | FLIXOTIDE 125micrograms inhaler |
| c65G. | FLUTICASONE PROPIONATE 25micrograms inhaler |
| c65H. | FLUTICASONE PROPIONATE 50micrograms inhaler |
| c65I. | FLUTICASONE PROPIONATE 125micrograms inhaler |
| c65K. | FLIXOTIDE 250micrograms inhaler |
| c65L. | FLIXOTIDE 500micrograms diskhaler |
| c65M. | FLIXOTIDE 500micrograms disk refill |
| c65N. | FLUTICASONE PROPIONATE 500micrograms disks+disk inhaler |
| c65O. | FLUTICASONE PROPIONATE 500micrograms disk refill |
| c65P. | FLUTICASONE PROPIONATE 50mcg breath-actuated dry powder inh |
| c65Q. | FLUTICASONE PROPIONATE 100mcg breath-actuated dry powder inh |
| c65R. | FLUTICASONE PROPIONATE 250mcg breath-actuated dry powder inh |
| c65S. | FLUTICASONE PROPIONATE 500mcg breath-actuated dry powder inh |
| c65T. | FLIXOTIDE 50micrograms Accuhaler |
| c65U. | FLIXOTIDE 100micrograms Accuhaler |
| c65V. | FLIXOTIDE 250micrograms Accuhaler |
| c65W. | FLIXOTIDE 500micrograms Accuhaler |
| c65X. | FLUTICASONE PROPIONATE 0.5mg/2mL nebulisation units |
| c65Y. | FLUTICASONE PROPIONATE 2mg/2mL nebulisation units |
| c65Z. | FLIXOTIDE 0.5mg/2mL Nebules |
| c65a. | FLIXOTIDE 2mg/2mL Nebules |
| c65b. | FLUTICASONE PROPIONATE 125micrograms CFC-free inhaler |
| c65c. | FLUTICASONE PROPIONATE 250micrograms CFC-free inhaler |
| c65d. | FLIXOTIDE 125micrograms Evohaler |
| c65e. | FLIXOTIDE 250micrograms Evohaler |
| c65f. | FLUTICASONE PROPIONATE 50micrograms CFC-free inhaler |
| c65g. | FLIXOTIDE 50micrograms Evohaler |
| c66.. | BECLOMETASONE DIPROPIONATE [RESPIRATORY USE 2] |
| c661. | *BDP 250micrograms Spacehaler |
| c662. | BECOTIDE 50 EASI-BREATHE inhaler |
| c663. | BECOTIDE 100 EASI-BREATHE inhaler |
| c664. | BECLOFORTE EASI-BREATHE 250micrograms inhaler |
| c665. | QVAR 50 inhaler |
| c666. | QVAR 100 inhaler |
| c667. | QVAR 50 Autohaler |
| c668. | QVAR 100 Autohaler |
| c669. | *BECLAZONE 200 inhaler |
| c66A. | BECLOMETASONE DIPROP 50mcg breath-act dry powder inhaler |
| c66B. | BECLOMETASONE DIPROP 100mcg breath-act dry powder inhaler |
| c66C. | BECLOMETASONE DIPROP 250mcg breath-act dry powder inhaler |
| c66D. | ASMABEC 50micrograms Clickhaler |
| c66E. | ASMABEC 100micrograms Clickhaler |
| c66F. | ASMABEC 250micrograms Clickhaler |
| c66G. | BECLOMETASONE DIPROP 400mcg breath-act dry powder inhaler |
| c66H. | BECLOMETASONE DIPROP 200mcg breath-act dry powder inhaler |
| c66I. | PULVINAL BECLOMETHASONE DIPROP 100mcg breath-act dry pdr inh |
| c66J. | PULVINAL BECLOMETHASONE DIPROP 200mcg breath-act dry pdr inh |
| c66K. | PULVINAL BECLOMETHASONE DIPROP 400mcg breath-act dry pdr inh |
| c66L. | BECLOMETASONE 100 Cyclocaps |
| c66M. | BECLOMETASONE 200 Cyclocaps |
| c66N. | BECLOMETASONE 400 Cyclocaps |
| c66P. | BECODISK 100micrograms diskhaler 15x8 |
| c66Q. | BECODISK 200micrograms diskhaler 15x8 |
| c66R. | BECODISK 400micrograms diskhaler 15x8 |
| c66S. | BECODISK 100micrograms disk refill 15x8 |
| c66T. | BECODISK 200micrograms disk refill 15x8 |
| c66U. | BECODISK 400micrograms disk refill 15x8 |
| c66V. | BECLOMETASONE DIPROPIONATE 50micrograms CFC-free inhaler |
| c66W. | BECLOMETASONE DIPROPIONATE 100micrograms CFC-free inhaler |
| c66X. | BECLOMETASONE DIPROPIONATE 50mcg CFC-free br-act inhaler |
| c66Y. | BECLOMETASONE DIPROPIONATE 100mcg CFC-free br-act inhaler |
| c66Z. | QVAR EASI-BREATHE 50mcg CFC-free breath-act dry pdr inhaler |
| c66a. | QVAR EASI-BREATHE 100mcg CFC-free breath-act dry pdr inhaler |
| c66c. | CLENIL MODULITE 50micrograms CFC-free inhaler |
| c66d. | CLENIL MODULITE 100micrograms CFC-free inhaler |
| c66e. | CLENIL MODULITE 200micrograms CFC-free inhaler |
| c66f. | CLENIL MODULITE 250micrograms CFC-free inhaler |
| c66g. | BECLOMETASONE DIPROPIONATE 200micrograms CFC-free inhaler |
| c66h. | BECLOMETASONE DIPROPIONATE 250micrograms CFC-free inhaler |
| c67.. | BUDESONIDE+FORMOTEROL |
| c671. | SYMBICORT 100/6 Turbohaler |
| c672. | SYMBICORT 200/6 Turbohaler |
| c673. | SYMBICORT 400/12 Turbohaler |
| c674. | DUORESP SPIROMAX 160mcg/4.5mcg breath-act dry powder inhaler |
| c675. | DUORESP SPIROMAX 320mcg/9mcg breath-act dry powder inhaler |
| c67x. | BUDESONIDE+FORMOTEROL FUMARATE 400/12mcg b-act dry pdr inh |
| c67y. | BUDESONIDE+FORMOTEROL FUMARATE 200/6mcg bth-act dry pdr inh |
| c67z. | BUDESONIDE+FORMOTEROL FUMARATE 100/6mcg bth-act dry pdr inh |
| c68.. | MOMETASONE [RESPIRATORY USE] |
| c681. | MOMETASONE FUROATE 200mcg breath-act dry powder inhaler |
| c682. | MOMETASONE FUROATE 400mcg breath-act dry powder inhaler |
| c683. | ASMANEX TWISTHALER 200mcg breath-act dry powder inhaler |
| c684. | ASMANEX TWISTHALER 400mcg breath-act dry powder inhaler |
| c69.. | CICLESONIDE |
| c691. | ALVESCO 160micrograms inhaler |
| c692. | ALVESCO 80micrograms inhaler |
| c69y. | CICLESONIDE 80micrograms inhaler |
| c69z. | CICLESONIDE 160micrograms inhaler |
| c6A.. | BECLOMETASONE+FORMOTEROL |
| c6A1. | FOSTAIR 100micrograms/6micrograms inhaler |
| c6A2. | FOSTAIR NEXTHALER 100micrograms/6micrograms powder inhaler |
| c6Ay. | BECLOMET DIPROP+FORMOTERL FUMARATE DIHYD 100mcg/6mcg pdr inh |
| c6Az. | BECLOMETASONE+FORMOTEROL 100micrograms/6micrograms inhaler |
| c6B.. | FLUTICASONE+VILANTEROL |
| c6B1. | RELVAR ELLIPTA 184micrograms/22micrograms inhaler |
| c6B2. | FLUTICASONE FUROATE+VILANTEROL 184mcg/22mcg dry pdr inhaler |
| c6B3. | RELVAR ELLIPTA 92micrograms/22micrograms inhaler |
| c6B4. | FLUTICASONE FUROATE+VILANTEROL 92mcg/22mcg dry pdr inhaler |
| c6… | CORTICOSTEROIDS [RESPIRATORY USE] |
| c71.. | SODIUM CROMOGLICATE [ASTHMA] |
| c711. | *INTAL 1mg inhaler |
| c712. | *INTAL HALERMATIC insufflator |
| c713. | *INTAL 20mg spincaps |
| c714. | *INTAL SPINHALER insufflator |
| c715. | INTAL 20mg/2mL nebuliser solution |
| c716. | *INTAL 5mg inhaler |
| c717. | SODIUM CROMOGLICATE 20mg inhalation capsules |
| c718. | SODIUM CROMOGLICATE 20mg/2mL nebuliser solution |
| c719. | SODIUM CROMOGLICATE 5mg inhaler |
| c71a. | *INTAL 5mg Autohaler |
| c71b. | STERI-NEB CROMOGEN 20mg nebulisation units |
| c71c. | *CROMOGEN 5mg inhaler |
| c71d. | INTAL FISONAIR 5mg inhaler + spacer device |
| c71e. | SODIUM CROMOGLICATE 5mg inhaler + spacer device |
| c71f. | SODIUM CROMOGLYCATE 5mg auto inhaler |
| c71g. | INTAL SYNCRONER 5mg inhaler + spacer device 2x112dose |
| c71h. | SODIUM CROMOGLICATE 5mg breath-actuated aerosol inhaler |
| c71i. | INTAL 5mg CFC-free inhaler |
| c71j. | CROMOGEN EASI-BREATHE 5mg breath-actuated aerosol inhaler |
| c71k. | SODIUM CROMOGLICATE 5mg CFC-free inhaler |
| c72.. | SODIUM CROMOGLICATE COMPOUNDS |
| c721. | *INTAL COMPOUND spincaps |
| c722. | *AEROCROM inhaler |
| c723. | AEROCROM SYNCRONER inhaler + spacer device |
| c72y. | SODIUM CROMOGLICATE+SALBUTAMOL 1mg/100mcg inhaler + spacer |
| c72z. | SODIUM CROMOGLICATE+SALBUTAMOL 1mg/100micrograms inhaler |
| c73.. | KETOTIFEN [ASTHMA PROPHYLAXIS] |
| c731. | *ZADITEN 1mg capsules |
| c732. | ZADITEN 1mg tablets |
| c733. | ZADITEN 1mg/5mL elixir |
| c734. | *KETOTIFEN 1mg capsules |
| c735. | KETOTIFEN 1mg tablets |
| c736. | KETOTIFEN 1mg/5mL elixir |
| c73x. | *KETOTIFEN 1mg capsules |
| c73y. | *KETOTIFEN 1mg tablets |
| c73z. | *KETOTIFEN 1mg/5mL elixir |
| c74.. | NEDOCROMIL SODIUM [ASTHMA] |
| c741. | *TILADE MINT 2mg inhaler |
| c742. | *NEDOCROMIL SODIUM 2mg inhaler |
| c743. | *TILADE MINT 2mg inhaler |
| c744. | TILADE MINT SYNCRONER 2mg inhaler |
| c745. | NEDOCROMIL SODIUM 2mg inhaler + spacer |
| c746. | NEDOCROMIL SODIUM 2mg CFC-free inhaler |
| c747. | TILADE 2mg CFC-free inhaler |
| c7… | Asthma prophylaxis |
| cA1.. | MONTELUKAST |
| cA11. | MONTELUKAST 10mg tablets |
| cA12. | MONTELUKAST 5mg chewable tablets |
| cA13. | SINGULAIR 10mg tablets |
| cA14. | SINGULAIR PAEDIATRIC 5mg chewable tablets |
| cA15. | SINGULAIR PAEDIATRIC 4mg chewable tablets |
| cA16. | SINGULAIR PAEDIATRIC 4mg/sachet granules |
| cA1y. | MONTELUKAST 4mg/sachet granules |
| cA1z. | MONTELUKAST 4mg chewable tablets |
| cA2.. | ZAFIRLUKAST |
| cA21. | ZAFIRLUKAST 20mg tablets |
| cA22. | ACCOLATE 20mg tablets |
| cA… | Leukotriene receptor antagonist |
| ck1.. | OMALIZUMAB |
| ck11. | OMALIZUMAB 150mg injection(pdr for recon)+solvent |
| ck12. | XOLAIR 150mg injection(pdr for recon)+solvent |
| ck13. | OMALIZUMAB 75mg/0.5mL soln for injection prefilled syringe |
| ck14. | XOLAIR 75mg/0.5mL solution for injection prefilled syringe |
| ck15. | OMALIZUMAB 150mg/1mL soln for injection prefilled syringe |
| ck16. | XOLAIR 150mg/1mL solution for injection prefilled syringe |
| ck… | Monoclonal IgE antibody |

**Table 25: Ear ICD10 codes**

| ICD10 | Description | |
| --- | --- | --- |
| H60 | Otitis externa | |
| H600 | Abscess of external ear | |
| H601 | Cellulitis of external ear | |
| H602 | Malignant otitis externa | |
| H603 | Other infective otitis externa | |
| H604 | Cholesteatoma of external ear | |
| H605 | Acute otitis externa, noninfective | |
| H608 | Other otitis externa | |
| H609 | Otitis externa, unspecified | |
| H61 | Other disorders of external ear | |
| H610 | Perichondritis of external ear | |
| H611 | Noninfective disorders of pinna | |
| H612 | Impacted cerumen | |
| H613 | Acquired stenosis of external ear canal | |
| H618 | Other specified disorders of external ear | |
| H619 | Disorder of external ear, unspecified | |
| H62 | Disorders of external ear in diseases classified elsewhere | |
| H620 | Otitis externa in bacterial diseases classified elsewhere | |
| H621 | Otitis externa in viral diseases classified elsewhere | |
| H622 | Otitis externa in mycoses | |
| H623 | Otitis externa in other infectious and parasitic diseases classified elsewhere | |
| H624 | Otitis externa in other diseases classified elsewhere | |
| H628 | Other disorders of external ear in diseases classified elsewhere | |
| H65 | Nonsuppurative otitis media | |
| H650 | Acute serous otitis media | |
| H651 | Other acute nonsuppurative otitis media | |
| H652 | Chronic serous otitis media | |
| H653 | Chronic mucoid otitis media | |
| H654 | Other chronic nonsuppurative otitis media | |
| H659 | Nonsuppurative otitis media, unspecified | |
| H66 | Suppurative and unspecified otitis media | |
| H660 | Acute suppurative otitis media | |
| H661 | Chronic tubotympanic suppurative otitis media | |
| H662 | Chronic atticoantral suppurative otitis media | |
| H663 | Other chronic suppurative otitis media | |
| H664 | Suppurative otitis media, unspecified | |
| H669 | Otitis media, unspecified | |
| H67 | Otitis media in diseases classified elsewhere | |
| H670 | Otitis media in bacterial diseases classified elsewhere | |
| H671 | Otitis media in viral diseases classified elsewhere | |
| H678 | Otitis media in other diseases classified elsewhere | |
| H68 | Eustachian salpingitis and obstruction | |
| H680 | Eustachian salpingitis | |
| H681 | Obstruction of Eustachian tube | |
| H69 | Other disorders of Eustachian tube | |
| H690 | Patulous Eustachian tube | |
| H698 | Other specified disorders of Eustachian tube | |
| H699 | Eustachian tube disorder, unspecified | |
| H70 | Mastoiditis and related conditions | |
| H700 | Acute mastoiditis | |
| H701 | Chronic mastoiditis | |
| H702 | Petrositis |  |
| H708 | Other mastoiditis and related conditions | |
| H709 | Mastoiditis, unspecified | |
| H71X | Cholesteatoma of middle ear | |
| H72 | Perforation of tympanic membrane | |
| H720 | Central perforation of tympanic membrane | |
| H721 | Attic perforation of tympanic membrane | |
| H722 | Other marginal perforations of tympanic membrane | |
| H728 | Other perforations of tympanic membrane | |
| H729 | Perforation of tympanic membrane, unspecified | |
| H73 | Other disorders of tympanic membrane | |
| H730 | Acute myringitis | |
| H731 | Chronic myringitis | |
| H738 | Other specified disorders of tympanic membrane | |
| H739 | Disorder of tympanic membrane, unspecified | |
| H74 | Other disorders of middle ear and mastoid | |
| H740 | Tympanosclerosis | |
| H741 | Adhesive middle ear disease | |
| H742 | Discontinuity and dislocation of ear ossicles | |
| H743 | Other acquired abnormalities of ear ossicles | |
| H744 | Polyp of middle ear | |
| H748 | Other specified disorders of middle ear and mastoid | |
| H749 | Disorder of middle ear and mastoid, unspecified | |
| H75 | Other disorders of middle ear and mastoid in diseases classified elsewhere | |
| H750 | Mastoiditis in infectious and parasitic diseases classified elsewhere | |
| H758 | Other specified disorders of middle ear and mastoid in diseases classified elsewhere | |
| H80 | Otosclerosis | |
| H800 | Otosclerosis involving oval window, nonobliterative | |
| H801 | Otosclerosis involving oval window, obliterative | |
| H802 | Cochlear otosclerosis | |
| H808 | Other otosclerosis | |
| H809 | Otosclerosis, unspecified | |
| H81 | Disorders of vestibular function | |
| H810 | MâniÅ re's disease | |
| H811 | Benign paroxysmal vertigo | |
| H812 | Vestibular neuronitis | |
| H813 | Other peripheral vertigo | |
| H814 | Vertigo of central origin | |
| H818 | Other disorders of vestibular function | |
| H819 | Disorder of vestibular function, unspecified | |
| H82X | Vertiginous syndromes in diseases classified elsewhere | |
| H83 | Other diseases of inner ear | |
| H830 | Labyrinthitis | |
| H831 | Labyrinthine fistula | |
| H832 | Labyrinthine dysfunction | |
| H833 | Noise effects on inner ear | |
| H838 | Other specified diseases of inner ear | |
| H839 | Disease of inner ear, unspecified | |
| H90 | Conductive and sensorineural hearing loss | |
| H900 | Conductive hearing loss, bilateral | |
| H901 | Conductive hearing loss, unilateral with unrestricted hearing on the contralateral side | |
| H902 | Conductive hearing loss, unspecified | |
| H903 | Sensorineural hearing loss, bilateral | |
| H904 | Sensorineural hearing loss, unilateral with unrestricted hearing on the contralateral side | |
| H905 | Sensorineural hearing loss, unspecified | |
| H906 | Mixed conductive and sensorineural hearing loss, bilateral | |
| H907 | Mixed conductive and sensorineural hearing loss, unilateral with unrestricted hearing on the contralateral side | |
| H908 | Mixed conductive and sensorineural hearing loss, unspecified | |
| H91 | Other hearing loss | |
| H910 | Ototoxic hearing loss | |
| H911 | Presbycusis | |
| H912 | Sudden idiopathic hearing loss | |
| H913 | Deaf mutism, not elsewhere classified | |
| H918 | Other specified hearing loss | |
| H919 | Hearing loss, unspecified | |
| H92 | Otalgia and effusion of ear | |
| H920 | Otalgia |  |
| H921 | Otorrhoea | |
| H922 | Otorrhagia | |
| H93 | Other disorders of ear, not elsewhere classified | |
| H930 | Degenerative and vascular disorders of ear | |
| H931 | Tinnitus |  |
| H932 | Other abnormal auditory perceptions | |
| H933 | Disorders of acoustic nerve | |
| H938 | Other specified disorders of ear | |
| H939 | Disorder of ear, unspecified | |
| H94 | Other disorders of ear in diseases classified elsewhere | |
| H940 | Acoustic neuritis in infectious and parasitic diseases classified elsewhere | |
| H948 | Other specified disorders of ear in diseases classified elsewhere | |
| H95 | Postprocedural disorders of ear and mastoid process, not elsewhere classified | |
| H950 | Recurrent cholesteatoma of post mastoidectomy cavity | |
| H951 | Other disorders following mastoidectomy | |
| H958 | Other postprocedural disorders of ear and mastoid process | |
| H959 | Postprocedural disorder of ear and mastoid process, unspecified | |

**Table 26: Eye ICD10 codes**

| ICD10 | Description |
| --- | --- |
| H00 | Hordeolum and chalazion |
| H000 | Hordeolum and other deep inflammation of eyelid |
| H001 | Chalazion |
| H01 | Other inflammation of eyelid |
| H010 | Blepharitis |
| H011 | Noninfectious dermatoses of eyelid |
| H018 | Other specified inflammation of eyelid |
| H019 | Inflammation of eyelid, unspecified |
| H02 | Other disorders of eyelid |
| H020 | Entropion and trichiasis of eyelid |
| H021 | Ectropion of eyelid |
| H022 | Lagophthalmos |
| H023 | Blepharochalasis |
| H024 | Ptosis of eyelid |
| H025 | Other disorders affecting eyelid function |
| H026 | Xanthelasma of eyelid |
| H027 | Other degenerative disorders of eyelid and periocular area |
| H028 | Other specified disorders of eyelid |
| H029 | Disorder of eyelid, unspecified |
| H03 | Disorders of eyelid in diseases classified elsewhere |
| H030 | Parasitic infestation of eyelid in diseases classified elsewhere |
| H031 | Involvement of eyelid in other infectious diseases classified elsewhere |
| H038 | Involvement of eyelid in other diseases classified elsewhere |
| H04 | Disorders of lacrimal system |
| H040 | Dacryoadenitis |
| H041 | Other disorders of lacrimal gland |
| H042 | Epiphora |
| H043 | Acute and unspecified inflammation of lacrimal passages |
| H044 | Chronic inflammation of lacrimal passages |
| H045 | Stenosis and insufficiency of lacrimal passages |
| H046 | Other changes in lacrimal passages |
| H048 | Other disorders of lacrimal system |
| H049 | Disorder of lacrimal system, unspecified |
| H05 | Disorders of orbit |
| H050 | Acute inflammation of orbit |
| H051 | Chronic inflammatory disorders of orbit |
| H052 | Exophthalmic conditions |
| H053 | Deformity of orbit |
| H054 | Enophthalmos |
| H055 | Retained (old) foreign body following penetrating wound of orbit |
| H058 | Other disorders of orbit |
| H059 | Disorder of orbit, unspecified |
| H06 | Disorders of lacrimal system and orbit in diseases classified elsewhere |
| H060 | Disorders of lacrimal system in diseases classified elsewhere |
| H061 | Parasitic infestation of orbit in diseases classified elsewhere |
| H062 | Dysthyroid exophthalmos |
| H063 | Other disorders of orbit in diseases classified elsewhere |
| H10 | Conjunctivitis |
| H100 | Mucopurulent conjunctivitis |
| H101 | Acute atopic conjunctivitis |
| H102 | Other acute conjunctivitis |
| H103 | Acute conjunctivitis, unspecified |
| H104 | Chronic conjunctivitis |
| H105 | Blepharoconjunctivitis |
| H108 | Other conjunctivitis |
| H109 | Conjunctivitis, unspecified |
| H11 | Other disorders of conjunctiva |
| H110 | Pterygium |
| H111 | Conjunctival degenerations and deposits |
| H112 | Conjunctival scars |
| H113 | Conjunctival haemorrhage |
| H114 | Other conjunctival vascular disorders and cysts |
| H118 | Other specified disorders of conjunctiva |
| H119 | Disorder of conjunctiva, unspecified |
| H13 | Disorders of conjunctiva in diseases classified elsewhere |
| H130 | Filarial infection of conjunctiva |
| H131 | Conjunctivitis in infectious and parasitic diseases classified elsewhere |
| H132 | Conjunctivitis in other diseases classified elsewhere |
| H133 | Ocular pemphigoid |
| H138 | Other disorders of conjunctiva in diseases classified elsewhere |
| H15 | Disorders of sclera |
| H150 | Scleritis |
| H151 | Episcleritis |
| H158 | Other disorders of sclera |
| H159 | Disorder of sclera, unspecified |
| H16 | Keratitis |
| H160 | Corneal ulcer |
| H161 | Other superficial keratitis without conjunctivitis |
| H162 | Keratoconjunctivitis |
| H163 | Interstitial and deep keratitis |
| H164 | Corneal neovascularization |
| H168 | Other keratitis |
| H169 | Keratitis, unspecified |
| H17 | Corneal scars and opacities |
| H170 | Adherent leukoma |
| H171 | Other central corneal opacity |
| H178 | Other corneal scars and opacities |
| H179 | Corneal scar and opacity, unspecified |
| H18 | Other disorders of cornea |
| H180 | Corneal pigmentations and deposits |
| H181 | Bullous keratopathy |
| H182 | Other corneal oedema |
| H183 | Changes in corneal membranes |
| H184 | Corneal degeneration |
| H185 | Hereditary corneal dystrophies |
| H186 | Keratoconus |
| H187 | Other corneal deformities |
| H188 | Other specified disorders of cornea |
| H189 | Disorder of cornea, unspecified |
| H19 | Disorders of sclera and cornea in diseases classified elsewhere |
| H190 | Scleritis and episcleritis in diseases classified elsewhere |
| H191 | Herpesviral keratitis and keratoconjunctivitis |
| H192 | Keratitis and keratoconjunctivitis in other infectious and parasitic diseases classified elsewhere |
| H193 | Keratitis and keratoconjunctivitis in other diseases classified elsewhere |
| H198 | Other disorders of sclera and cornea in diseases classified elsewhere |
| H20 | Iridocyclitis |
| H200 | Acute and subacute iridocyclitis |
| H201 | Chronic iridocyclitis |
| H202 | Lens-induced iridocyclitis |
| H208 | Other iridocyclitis |
| H209 | Iridocyclitis, unspecified |
| H21 | Other disorders of iris and ciliary body |
| H210 | Hyphaema |
| H211 | Other vascular disorders of iris and ciliary body |
| H212 | Degeneration of iris and ciliary body |
| H213 | Cyst of iris, ciliary body and anterior chamber |
| H214 | Pupillary membranes |
| H215 | Other adhesions and disruptions of iris and ciliary body |
| H218 | Other specified disorders of iris and ciliary body |
| H219 | Disorder of iris and ciliary body, unspecified |
| H22 | Disorders of iris and ciliary body in diseases classified elsewhere |
| H220 | Iridocyclitis in infectious and parasitic diseases classified elsewhere |
| H221 | Iridocyclitis in other diseases classified elsewhere |
| H228 | Other disorders of iris and ciliary body in diseases classified elsewhere |
| H25 | Senile cataract |
| H250 | Senile incipient cataract |
| H251 | Senile nuclear cataract |
| H252 | Senile cataract, morgagnian type |
| H258 | Other senile cataract |
| H259 | Senile cataract, unspecified |
| H26 | Other cataract |
| H260 | Infantile, juvenile and presenile cataract |
| H261 | Traumatic cataract |
| H262 | Complicated cataract |
| H263 | Drug-induced cataract |
| H264 | After-cataract |
| H268 | Other specified cataract |
| H269 | Cataract, unspecified |
| H27 | Other disorders of lens |
| H270 | Aphakia |
| H271 | Dislocation of lens |
| H278 | Other specified disorders of lens |
| H279 | Disorder of lens, unspecified |
| H28 | Cataract and other disorders of lens in diseases classified elsewhere |
| H280 | Diabetic cataract |
| H281 | Cataract in other endocrine, nutritional and metabolic diseases |
| H282 | Cataract in other diseases classified elsewhere |
| H288 | Other disorders of lens in diseases classified elsewhere |
| H30 | Chorioretinal inflammation |
| H300 | Focal chorioretinal inflammation |
| H301 | Disseminated chorioretinal inflammation |
| H302 | Posterior cyclitis |
| H308 | Other chorioretinal inflammations |
| H309 | Chorioretinal inflammation, unspecified |
| H31 | Other disorders of choroid |
| H310 | Chorioretinal scars |
| H311 | Choroidal degeneration |
| H312 | Hereditary choroidal dystrophy |
| H313 | Choroidal haemorrhage and rupture |
| H314 | Choroidal detachment |
| H318 | Other specified disorders of choroid |
| H319 | Disorder of choroid, unspecified |
| H32 | Chorioretinal disorders in diseases classified elsewhere |
| H320 | Chorioretinal inflammation in infectious and parasitic diseases classified elsewhere |
| H328 | Other chorioretinal disorders in diseases classified elsewhere |
| H33 | Retinal detachments and breaks |
| H330 | Retinal detachment with retinal break |
| H331 | Retinoschisis and retinal cysts |
| H332 | Serous retinal detachment |
| H333 | Retinal breaks without detachment |
| H334 | Traction detachment of retina |
| H335 | Other retinal detachments |
| H34 | Retinal vascular occlusions |
| H340 | Transient retinal artery occlusion |
| H341 | Central retinal artery occlusion |
| H342 | Other retinal artery occlusions |
| H348 | Other retinal vascular occlusions |
| H349 | Retinal vascular occlusion, unspecified |
| H35 | Other retinal disorders |
| H350 | Background retinopathy and retinal vascular changes |
| H351 | Retinopathy of prematurity |
| H352 | Other proliferative retinopathy |
| H353 | Degeneration of macula and posterior pole |
| H354 | Peripheral retinal degeneration |
| H355 | Hereditary retinal dystrophy |
| H356 | Retinal haemorrhage |
| H357 | Separation of retinal layers |
| H358 | Other specified retinal disorders |
| H359 | Retinal disorder, unspecified |
| H36 | Retinal disorders in diseases classified elsewhere |
| H360 | Diabetic retinopathy |
| H368 | Other retinal disorders in diseases classified elsewhere |
| H40 | Glaucoma |
| H400 | Glaucoma suspect |
| H401 | Primary open-angle glaucoma |
| H402 | Primary angle-closure glaucoma |
| H403 | Glaucoma secondary to eye trauma |
| H404 | Glaucoma secondary to eye inflammation |
| H405 | Glaucoma secondary to other eye disorders |
| H406 | Glaucoma secondary to drugs |
| H408 | Other glaucoma |
| H409 | Glaucoma, unspecified |
| H42 | Glaucoma in diseases classified elsewhere |
| H420 | Glaucoma in endocrine, nutritional and metabolic diseases |
| H428 | Glaucoma in other diseases classified elsewhere |
| H43 | Disorders of vitreous body |
| H430 | Vitreous prolapse |
| H431 | Vitreous haemorrhage |
| H432 | Crystalline deposits in vitreous body |
| H433 | Other vitreous opacities |
| H438 | Other disorders of vitreous body |
| H439 | Disorder of vitreous body, unspecified |
| H44 | Disorders of globe |
| H440 | Purulent endophthalmitis |
| H441 | Other endophthalmitis |
| H442 | Degenerative myopia |
| H443 | Other degenerative disorders of globe |
| H444 | Hypotony of eye |
| H445 | Degenerated conditions of globe |
| H446 | Retained (old) intraocular foreign body, magnetic |
| H447 | Retained (old) intraocular foreign body, nonmagnetic |
| H448 | Other disorders of globe |
| H449 | Disorder of globe, unspecified |
| H45 | Disorders of vitreous body and globe in diseases classified elsewhere |
| H450 | Vitreous haemorrhage in diseases classified elsewhere |
| H451 | Endophthalmitis in diseases classified elsewhere |
| H458 | Other disorders of vitreous body and globe in diseases classified elsewhere |
| H46X | Optic neuritis |
| H47 | Other disorders of optic [2nd] nerve and visual pathways |
| H470 | Disorders of optic nerve, not elsewhere classified |
| H471 | Papilloedema, unspecified |
| H472 | Optic atrophy |
| H473 | Other disorders of optic disc |
| H474 | Disorders of optic chiasm |
| H475 | Disorders of other visual pathways |
| H476 | Disorders of visual cortex |
| H477 | Disorder of visual pathways, unspecified |
| H48 | Disorders of optic [2nd] nerve and visual pathways in diseases classified elsewhere |
| H480 | Optic atrophy in diseases classified elsewhere |
| H481 | Retrobulbar neuritis in diseases classified elsewhere |
| H488 | Other disorders of optic nerve and visual pathways in diseases classified elsewhere |
| H49 | Paralytic strabismus |
| H490 | Third [oculomotor] nerve palsy |
| H491 | Fourth [trochlear] nerve palsy |
| H492 | Sixth [abducent] nerve palsy |
| H493 | Total (external) ophthalmoplegia |
| H494 | Progressive external ophthalmoplegia |
| H498 | Other paralytic strabismus |
| H499 | Paralytic strabismus, unspecified |
| H50 | Other strabismus |
| H500 | Convergent concomitant strabismus |
| H501 | Divergent concomitant strabismus |
| H502 | Vertical strabismus |
| H503 | Intermittent heterotropia |
| H504 | Other and unspecified heterotropia |
| H505 | Heterophoria |
| H506 | Mechanical strabismus |
| H508 | Other specified strabismus |
| H509 | Strabismus, unspecified |
| H51 | Other disorders of binocular movement |
| H510 | Palsy of conjugate gaze |
| H511 | Convergence insufficiency and excess |
| H512 | Internuclear ophthalmoplegia |
| H518 | Other specified disorders of binocular movement |
| H519 | Disorder of binocular movement, unspecified |
| H52 | Disorders of refraction and accommodation |
| H520 | Hypermetropia |
| H521 | Myopia |
| H522 | Astigmatism |
| H523 | Anisometropia and aniseikonia |
| H524 | Presbyopia |
| H525 | Disorders of accommodation |
| H526 | Other disorders of refraction |
| H527 | Disorder of refraction, unspecified |
| H53 | Visual disturbances |
| H530 | Amblyopia ex anopsia |
| H531 | Subjective visual disturbances |
| H532 | Diplopia |
| H533 | Other disorders of binocular vision |
| H534 | Visual field defects |
| H535 | Colour vision deficiencies |
| H536 | Night blindness |
| H538 | Other visual disturbances |
| H539 | Visual disturbance, unspecified |
| H54 | Visual impairment including blindness (binocular or monocular) |
| H540 | Blindness, binocular |
| H541 | Severe visual impairment, binocular |
| H542 | Moderate visual impairment, binocular |
| H543 | Mild or no visual impairment, binocular |
| H544 | Blindness, monocular |
| H545 | Severe visual impairment, monocular |
| H546 | Moderate visual impairment, monocular |
| H547 | Unspecified visual loss |
| H549 | Unspecified visual impairment (binocular) |
| H55X | Nystagmus and other irregular eye movements |
| H57 | Other disorders of eye and adnexa |
| H570 | Anomalies of pupillary function |
| H571 | Ocular pain |
| H578 | Other specified disorders of eye and adnexa |
| H579 | Disorder of eye and adnexa, unspecified |
| H58 | Other disorders of eye and adnexa in diseases classified elsewhere |
| H580 | Anomalies of pupillary function in diseases classified elsewhere |
| H581 | Visual disturbances in diseases classified elsewhere |
| H588 | Other specified disorders of eye and adnexa in diseases classified elsewhere |
| H59 | Postprocedural disorders of eye and adnexa, not elsewhere classified |
| H590 | Keratopathy (bullous aphakic) following cataract surgery |
| H598 | Other postprocedural disorders of eye and adnexa |
| H599 | Postprocedural disorder of eye and adnexa, unspecified |
