# Supplementary material for: Factors associated with low school readiness, a linked health and education data study in Wales, UK
Source: PLoS One. 2023 Dec 11;18(12):e0273596. doi: 10.1371/journal.pone.0273596 (PMC10712842; doi:10.1371/journal.pone.0273596)
Supplement: S1 File — (ZIP) [file pone.0273596.s001.zip › Appendix4_supplementaryfile_studypopchar_stratified.docx]

# Appendix 4: Demographic table stratified by school readiness.

**Table 1: Characteristics of the study population stratified by school readiness.**

| Variables | School ready (n =122,487) | | Not school ready (n= 20,468) | |
| --- | --- | --- | --- | --- |
| Gender |  |  |  |  |
| Girl | 62,713 | 51.20% | 6,990 | 34.15% |
| Boy | 59,774 | 48.80% | 13,478 | 65.85% |
| Gestational age (between 22 and 45) |  |  |  |  |
| Extremely pre-term: <28 weeks | 219 | 0.18% | 139 | 0.68% |
| Very pre-term: 28-31 | 862 | 0.70% | 311 | 1.52% |
| Pre-term: 32-36 | 6,740 | 5.50% | 1,694 | 8.28% |
| Term: 37-42 | 113,189 | 92.41% | 18,060 | 88.24% |
| Late term: 43-45 | 746 | 0.61% | 153 | 0.75% |
| Unknown/NULL | 731 | 0.60% | 111 | 0.54% |
| Birth weight |  |  |  |  |
| Very low: <1500g | 1,001 | 0.82% | 453 | 2.21% |
| Low: 1500-<2500g | 6,303 | 5.15% | 1882 | 9.19% |
| Normal: 2500-<4000g | 99,778 | 81.46% | 16,066 | 78.49% |
| High: 4000-5000g | 14,820 | 12.10% | 1,982 | 9.68% |
| Unknown | 585 | 0.48% | 85 | 0.42% |
| Breastfeeding |  |  |  |  |
| No | 50,371 | 41.12% | 10,916 | 53.33% |
| Yes | 65,678 | 53.62% | 8,310 | 40.60% |
| Unknown | 6,438 | 5.26% | 1242 | 6.07% |
| C- section birth |  |  |  |  |
|  | 30,432 | 24.85% | 5,057 | 24.71% |
|  |  |  |  |  |
| Multiple birth flag |  |  |  |  |
| Non-singleton | 3,282 | 2.68% | 640 | 3.13% |
| Maternal age (between 10 and 65) |  |  |  |  |
| Less than 19 | 9,289 | 7.58% | 2,621 | 12.81% |
| 20-24 | 26,631 | 21.74% | 5,753 | 28.11% |
| 25-29 | 34,022 | 27.78% | 5,334 | 26.06% |
| 30-34 | 31,895 | 26.04% | 3,945 | 19.27% |
| 35 and above | 20,645 | 16.85% | 2,813 | 13.74% |
| Unknown | <10 |  | <10 |  |
| Death of mother before Foundation Phase |  |  |  |  |
|  | 258 | 0.21% | 69 | 0.34% |
| Diabetes PEDW (mother) |  |  |  |  |
|  | 1,173 | 0.96% | 289 | 1.41% |
| Diabetes GP (mother) |  |  |  |  |
|  | 1074 | 0.88% | 293 | 1.43% |
| Cancer PEDW (mother) |  |  |  |  |
|  | 1,046 | 0.85% | 146 | 0.71% |
| Cancer GP (mother) |  |  |  |  |
|  | 916 | 0.75% | 121 | 0.59% |
| Anaemia PEDW (mother) |  |  |  |  |
|  | 6,196 | 5.06% | 1121 | 5.48% |
| Anaemia GP (mother) |  |  |  |  |
|  | 13,324 | 10.88% | 2,356 | 11.51% |
| Hypertension GP (mother) |  |  |  |  |
|  | 2,192 | 1.79% | 407 | 1.99% |
| Learning Difficulty GP (mother) |  |  |  |  |
|  | 90 | 0.07% | 115 | 0.56% |
| Depression PEDW (mother) |  |  |  |  |
|  | 4,016 | 3.28% | 1163 | 5.68% |
| Depression GP (mother) |  |  |  |  |
|  | 23,766 | 19.40% | 5,566 | 27.19% |
| Anxiety PEDW (mother) |  |  |  |  |
|  | 2,301 | 1.88% | 612 | 2.99% |
| Anxiety GP (mother) |  |  |  |  |
|  | 25,054 | 20.45% | 5,224 | 25.52% |
| Anti-Depression/anxiety medication (mother) |  |  |  |  |
|  | 325 | 0.27% | 71 | 0.35% |
| Serious Mental Illness PEDW (mother) |  |  |  |  |
|  | 538 | 0.44% | 172 | 0.84% |
| Serious Mental Illness GP (mother) |  |  |  |  |
|  | 599 | 0.49% | 177 | 0.86% |
| Alcohol PEDW during pregnancy (mother) |  |  |  |  |
| During pregnancy | 91 | 0.07% | 46 | 0.22% |
| After pregnancy | 1,046 | 0.85% | 316 | 1.54% |
| Smoking GP (mother) |  |  |  |  |
| During pregnancy | 16,469 | 13.45% | 4,444 | 21.71% |
| After pregnancy | 30,302 | 24.74% | 6,840 | 33.42% |
| Substance misuse (any) SMD (mother) |  |  |  |  |
| During pregnancy | 123 | 0.10% | 44 | 0.21% |
| after pregnancy | 1,540 | 1.26% | 544 | 2.66% |
| Substance misuse (other drug) PEDW (mother) |  |  |  |  |
| During pregnancy | 175 | 0.14% | 97 | 0.47% |
| After pregnancy | 997 | 0.81% | 358 | 1.75% |
| Substance misuse (other drug) GP (mother) |  |  |  |  |
| During pregnancy | 349 | 0.28% | 162 | 0.79% |
| after pregnancy | 1,420 | 1.16% | 497 | 2.43% |
| Assault PEDW (mother) |  |  |  |  |
|  | 430 | 0.35% | 142 | 0.69% |
| Diabetes PEDW (child) |  |  |  |  |
|  | 179 | 0.15% | 33 | 0.16% |
| Diabetes GP (child) |  |  |  |  |
|  | 168 | 0.14% | 31 | 0.15% |
| Epilepsy PEDW (child) |  |  |  |  |
|  | 263 | 0.21% | 389 | 1.90% |
| Epilepsy GP (child) |  |  |  |  |
|  | 423 | 0.35% | 493 | 2.41% |
| Asthma PEDW (child) |  |  |  |  |
|  | 3,681 | 3.01% | 1,038 | 5.07% |
| Asthma GP (child) |  |  |  |  |
|  | 49,468 | 40.39% | 8,505 | 41.55% |
| Ear PEDW (child) |  |  |  |  |
|  | 5,048 | 4.12% | 1,445 | 7.06% |
| Eye PEDW (child) |  |  |  |  |
|  | 2,925 | 2.39% | 911 | 4.45% |
| Any emergency hospital admission (child) |  |  |  |  |
|  | 67,512 | 55.12% | 13,076 | 63.89% |
| Any A&E attendance (child) |  |  |  |  |
|  | 80,475 | 65.70% | 14,449 | 70.59% |
| Learning Difficulty (child) |  |  |  |  |
|  | 215 | 0.18% | 1,075 | 5.25% |
| Free school meal |  |  |  |  |
|  | 20,230 | 16.52% | 7,741 | 37.82% |
| WIMD 2014 - overall |  |  |  |  |
| 1 (most deprived) | 29,360 | 23.97% | 7,322 | 35.77% |
| 2 | 25,737 | 21.01% | 4,910 | 23.99% |
| 3 | 22,992 | 18.77% | 3,494 | 17.07% |
| 4 | 19,705 | 16.09% | 2,578 | 12.60% |
| 5 (least deprived) | 24,693 | 20.16% | 2,164 | 10.57% |
| Local area- urban/rural |  |  |  |  |
| Rural town and fringe | 16,323 | 13.33% | 2,711 | 13.25% |
| Rural town and fringe in a sparse setting | 3,019 | 2.46% | 525 | 2.56% |
| Rural village and dispersed | 5,495 | 4.49% | 791 | 3.86% |
| Rural village and dispersed in a sparse setting | 6,280 | 5.13% | 928 | 4.53% |
| Urban city and town | 88,864 | 72.55% | 15,081 | 73.68% |
| Urban city and town in a sparse setting | 2,506 | 2.05% | 432 | 2.11% |
| No of adult in the household |  |  |  |  |
| 1 | 25824 | 21.08% | 5700 | 27.85% |
| 2 | 73447 | 59.96% | 10251 | 50.08% |
| 3 | 14542 | 11.87% | 2818 | 13.77% |
| 4 or above | 8674 | 7.08% | 1,699 | 8.30% |
| No of children in the household (excluding the cohort member) |  |  |  |  |
| 0 | 20,178 | 16.47% | 3,528 | 17.24% |
| 1 | 59,700 | 48.74% | 7,993 | 39.05% |
| 2 | 28,761 | 23.48% | 5,228 | 25.54% |
| 3 | 9,404 | 7.68% | 2,310 | 11.29% |
| 4 or above | 4,444 | 3.63% | 1,409 | 6.88% |
