# Supplementary material for: Factors associated with low school readiness, a linked health and education data study in Wales, UK
Source: PLoS One. 2023 Dec 11;18(12):e0273596. doi: 10.1371/journal.pone.0273596 (PMC10712842; doi:10.1371/journal.pone.0273596)
Supplement: S1 File — (ZIP) [file pone.0273596.s001.zip › Appendix5_supplementayfile_missingdata_selection_bias.docx]

# Appendix 5: Comparing the demography between final study population and the missing group.

**Table 1: Characteristics of the missing group**

|  | Final population (142,955) | | Missing group (89,063) | |
| --- | --- | --- | --- | --- |
| Gender | | | | |
| Boy | 73,252 | 51% | 45,253 | 51% |
| Girl | 69,703 | 49% | 43,810 | 49% |
|  |  |  |  |  |
| Free School meal | | | | |
| No | 114,984 | 80% | 71,346 | 80% |
| Yes | 27,971 | 20% | 17,717 | 20% |
